# *Panax ginseng* genome examination for ginsenoside biosynthesis

**DOI:** 10.1093/gigascience/gix093

**Published:** 2017-10-05

**Authors:** Jiang Xu, Yang Chu, Baosheng Liao, Shuiming Xiao, Qinggang Yin, Rui Bai, He Su, Linlin Dong, Xiwen Li, Jun Qian, Jingjing Zhang, Yujun Zhang, Xiaoyan Zhang, Mingli Wu, Jie Zhang, Guozheng Li, Lei Zhang, Zhenzhan Chang, Yuebin Zhang, Zhengwei Jia, Zhixiang Liu, Daniel Afreh, Ruth Nahurira, Lianjuan Zhang, Ruiyang Cheng, Yingjie Zhu, Guangwei Zhu, Wei Rao, Chao Zhou, Lirui Qiao, Zhihai Huang, Yung-Chi Cheng, Shilin Chen

**Affiliations:** 1 Institute of Chinese Materia Medica, China Academy of Chinese Medical Sciences, Beijing 100700, China; 2 Guangdong Provincial Hospital of Chinese Medicine, Guangzhou 510006, China; 3 National Data Center of Traditional Chinese Medicine, China Academy of Chinese Medical Sciences, Beijing 100700, China; 4 Institute of Basic Research in Clinical Medicine, China Academy of Chinese Medical Sciences, Beijing 100700, China; 5 Department of Biophysics, School of Basic Medical Sciences, Peking University Health Science Center, Beijing 100191, China; 6 State Key Laboratory of Molecular Reaction Dynamics, Dalian Institute of Chemical Physics, Chinese Academy of Sciences, Dalian 116023, China; 7 Waters Corporation Shanghai Science & Technology Co Ltd, Shanghai 201206, China; 8 Institute of Crop Science, Chinese Academy of Agricultural Sciences/Key Laboratory of Crop Physiology and Ecology, Ministry of Agriculture, Beijing 100081, China; 9 Department of Pharmacology, School of Medicine, Yale University, New Haven, CT 06510, USA

**Keywords:** *Panax ginseng*, ginsenosides, genome, mass spectrometry imaging

## Abstract

Ginseng, which contains ginsenosides as bioactive compounds, has been regarded as an important traditional medicine for several millennia. However, the genetic background of ginseng remains poorly understood, partly because of the plant's large and complex genome composition. We report the entire genome sequence of *Panax ginseng* using next-generation sequencing. The 3.5-Gb nucleotide sequence contains more than 60% repeats and encodes 42 006 predicted genes. Twenty-two transcriptome datasets and mass spectrometry images of ginseng roots were adopted to precisely quantify the functional genes. Thirty-one genes were identified to be involved in the mevalonic acid pathway. Eight of these genes were annotated as 3-hydroxy-3-methylglutaryl-CoA reductases, which displayed diverse structures and expression characteristics. A total of 225 UDP-glycosyltransferases (UGTs) were identified, and these UGTs accounted for one of the largest gene families of ginseng. Tandem repeats contributed to the duplication and divergence of UGTs. Molecular modeling of UGTs in the 71st, 74th, and 94th families revealed a regiospecific conserved motif located at the N-terminus. Molecular docking predicted that this motif captures ginsenoside precursors. The ginseng genome represents a valuable resource for understanding and improving the breeding, cultivation, and synthesis biology of this key herb.

## Background

*Panax ginseng* C. A. Mey, a deciduous perennial plant belonging to the Araliaceae family, has been clinically used as a precious herbal medicine for several millennia in East Asia [1]. The name ginseng was translated from the pronunciation of the Chinese words “Ren shen” [2]. Modern pharmacological research has focused on the ginsenosides, the major bioactive compound of *P. ginseng*, looking to see if they exhibit multiple therapeutic activities. These activities include antitumor, antihypertensive, antivirus, and immune modulatory activities [3]. Because of this, *P. ginseng* has been adopted as a general tonic or adaptogen to promote longevity, particularly in China, Korea, and Japan [4].

Different ginseng tissues, such as the root and rhizome used in clinical practice, show significant differences in quality evaluation, commercial application, and clinical efficacy because of variations in ginsenosides [5]. Ginsenosides are frequently allocated and accumulated in specific tissues through transport systems for storage or defense. Chemical analysis, immunological staining, and microscopic imaging have all demonstrated that the ginseng cortex and periderm contain higher amounts of protopanaxadiol (PPD)-type ginsenoside (Rb1, Rb2, or Rc) and protopanaxatriol (PPT)-type ginsenoside (Rf) than those of the root medulla [6, 7]. Histochemical staining also confirmed that ginsenosides are mainly located in the oil canals of the periderm and outer cortex regions of the root but not in the xylem or pith [8, 9]. Considering their potential physiological role [10], ginsenoside enrichment in the periderm is consistent with the plant's biological function as phytoanticipins, which protects plants against pathogens.

Although the pharmacological importance of ginsenosides has been well established, their biosynthetic enzymes and regulatory mode of action remain unknown [11, 12]. Ginsenosides are biosynthesized through the cytosolic mevalonic acid (MVA) pathway, which is initiated by acetyl coenzyme A and ends with the terpene precursor isopentenyl diphosphate (IPP). After a series of condensation reactions, a linear C30 molecule, squalene, is generated [13] and converted into (S)-2,3-oxidosqualene [14] through cyclization [15]. Subsequently, after multiple oxidation events (e.g., mediated by cytochrome P450-dependent monooxygenases) [16, 17], various types of ginsenoside precursors, including oleanolic acid and PPD/PPT, are formed. The precursors are then further decorated through glycosylation reactions [11, 18, 12].

The glycosylation reaction, namely the transfer of a sugar moiety to a specific acceptor, is performed by glycosyltransferases (GTs), a group of multigene superfamilies. The GTs that utilize uridine diphosphate (UDP) and activate sugar molecules as donors are referred to as UDP-glycosyltransferases (UGTs). The diversity of the UGTs has been demonstrated by comparing genomic and complementary DNA (cDNA) sequences. In our previous work, 129 potential UGT sequences were predicted on the basis of annotation results from the transcriptome data of *P. ginseng* roots, stems, leaves, and flowers. Some of the sequences may encode enzymes responsible for ginsenoside backbone modification [19]. However, a limited number of UGTs that glycosylate triterpenoid aglycones have been described in plants, such as *Medicago truncatula* [20], *Saponaria vaccaria* [21], *Barbarea vulgaris* [22], *Glycine max* [23], and *P. ginseng* [24–25]. Yan et al. [26] reported that the UGTPg1 from *P. ginseng* glycosylates the C20-OH of PPD and its derived ginsenosides in a regiospecific manner. Two recently identified UGTs from *P. ginseng* (PgUGT74AE2 and PgUGT94Q2) catalyze the glycosylation of the C3–OH of PPD to obtain Rh2 and elongate the glucose moiety of Rh2 to generate Rg3 [25]. Wei et al. [27] found that UGT1 and its homologous genes from *P. ginseng* can glycosylate PPT to produce PPT-derived ginsenosides, which contain several key amino acids that determine their activities and substrate regiospecificities.

The functional genomic analysis of ginseng has progressed significantly but still requires improvement. First, the analysis of gene and transcript expression has mainly focused on ginseng organs, but the ginsenoside content and types vary among different tissues within the same organ. Hence, the screening of potential key genes responsible for synthesizing and modifying ginsenosides by association analysis of the transcriptome and chemical composition is not comprehensive. Second, gene duplication often leads to functional divergence. Even paralogous genes that execute the same function are usually regulated in different modes. In ginseng, the ubiquitous duplicated genes are difficult to fully elucidate using current datasets. Therefore, the analysis of the whole-genome sequence and transcriptomes by the accurate location of ginsenosides may promote the precise mining of genes associated with ginsenoside synthesis. Herein, we present the genome sequence of *P. ginseng* and comprehensively characterize the genes responsible for ginsenoside biosynthesis and modification in the plant.

## Data Description

Genomic DNA was extracted from the 4-year old *P. ginseng* line IR826, a high-quality strain with low heterozygosity. This strain is cultivated by the Institute of Chinese Materia Medica in the Jilin province of China. Five libraries with insert sizes ranging from 250 bp to 10 kb were constructed. Paired-end sequencing was performed using the HiSeq X-Ten platform (Illumina) and 391.46 Gb of raw data were produced (Supplementary Table S1). The raw reads were trimmed using skewer pipeline to remove low-quality or duplicated reads. After trimming, 315.93 Gb of data were used for genome assembly. The final assembly was checked using Benchmarking Universal Single-Copy Orthologs (BUSCOs). The frozen transverse sections of the ginseng main root with 20 μm thickness were prepared using a microcryotome for DESI-MS imaging. The ginsenoside distribution was evaluated on a Xevo G2-XS Tof mass spectrometer with the DESI source. The image creation was performed using high-definition imaging (HDI) software (Waters Corporation, MA, USA) with the following parameters: X and Y pixel size 100 μm; raster speed 400 μm/s; spray solvent 90% MeOH, 10% H_2_O, 0.1 mM NH_4_Cl, and 0.1 mM leucine enkephalin delivered at 1.5 μL/min; MS at negative polarity, 4.5 kV capillary voltage, 80 V cone voltage, and mass range m/z 100–1200. Total RNA were isolated from the periderm, cortex, and stele to construct RNA-seq libraries, each in triplicates. The RNA-Seq transcriptome libraries were prepared following the TruSeq^TM^ RNA sample preparation kit (Illumina, Santiago, CA, USA). After quantification, the paired-end libraries were sequenced by HiSeq X-Ten (Illumina) (Supplementary Table S2). Except for the 9 RNA-seq data generated in this study, 13 published ginseng RNA-seq data were re-used. Further details about sample collection, DNA/RNA extraction, library construction, sequencing, and mass spectrometry imaging can be found in the “Methods” section. Genome and DESI-MS imaging data have been uploaded to *Giga*DB (*Giga*DB, RRID:SCR_004002) [28], and raw sequencing reads can be found at NCBI under the project number PRJNA385956.

## Analyses

### Characteristics of the *P. ginseng* genome

Genomic DNA was extracted from the 4-year-old *P. ginseng* line IR826, a strain cultivated by the Institute of Chinese Materia Medica. This strain contains an estimated genome size of 3.5 Gb based on the k-mer prediction and flow cytometry analysis (Supplementary Figure S1; Supplementary Table S3). Approximately ×112 coverage of the raw sequence was generated using the Illumina HiSeq X-Ten platform (Supplementary Table S1). After filtering, ×91 high-quality reads were adopted for assembly (Supplementary Table S1). The results provided a 3.43-Gb draft assembly with a contig N50 of 21.98 kb and a scaffold N50 of 108.71 kb (Table 1). Shotgun libraries with an insert size of 250 bp and 500 bp were mapped to the assembly, which had read mapping rates of 99.77% and 99.95%, respectively. The Poisson-like distribution of the sequence depth per base represents a nonbiased sequencing and assembly (Supplementary Figure S2). To confirm the accuracy, the 75 878 transcripts assembled from RNA-Seq data using Trinity (Trinity, RRID:SCR_013048) [29] with default parameters were mapped back to the assembly, with a mapping rate of 97.76%. Furthermore, BUSCOs [30] were used for quality assessment. A total of 1323 (91.88%) CEG proteins, of which 24 BUSCOs were fragments, were determined in this assembly; 98.19% of the proteins were fully annotated, indicating the accuracy of the assembly.

**Table 1:** Statistical analysis of the *P*. *ginseng* draft genome

	Size, bp	Number
Contig		
N90	4516	150 620
N80	8639	103 388
N70	12 833	75 040
N50	21 977	39 481
Longest	574 183	–
Total size	2 999 700 459	337 439
Scaffold		
N90	24 143	33 423
N80	45 718	23 391
N70	65 171	17 168
N50	108 708	9072
Longest	1 303 414	–
Total size	3 414 349 854	83 074
Gap ratio	12.15%^a^	–

^a^Among these gaps, 368 679 gaps are single-N.

More than 62% of the ginseng genome was predicted to be repeats; about 83.5% of the repeats were annotated as long terminal repeats (LTRs) (Supplementary Table S4 and S5). Ty3/Gypsy is the most abundant retro-element superfamily and accounts for 42.8% of the genome (Supplementary Table S6), which was higher than previously reported [31]. Moreover, the amount of Ty1/Copia comprised approximately 8.3% of the whole genome and exceeded previous predictions (Supplementary Table S6) [31]. For the DNA transposon class, CMC was the most abundant repeat type and comprised 43 Mb of approximately 1.3% of the genome (Supplementary Table S6).

A total of 42 006 protein-coding gene models were predicted on the basis of *ab initio* and comparison methods using the MAKER pipeline. That is, 88% of these models were supported by the assembled RNA-Seq transcripts. More than 95.6% of the gene models contained homologs in the GenBank nonredundant database (E-value = 1e^−5^). About 73.47% of annotations could be assigned to gene ontology (GO) catalogs, and 68.39% could be assigned to Kyoto Encyclopedia of Genes and Genomes (KEGG, RRID:SCR_012773) pathways (Supplementary Figure S3). Among these annotations, the following genes were obtained: 488 cytochrome P450 genes, including the PPD-ginsenosides synthase (PPDS) CYP716A47, PPT-ginsenosides synthase (PPTS) CYP716A53, and oleanolic acid synthase CYP716A52; 2556 transcription factors; and 3745 transporters (Supplementary Table S7 and S8).

**Figure 1:**
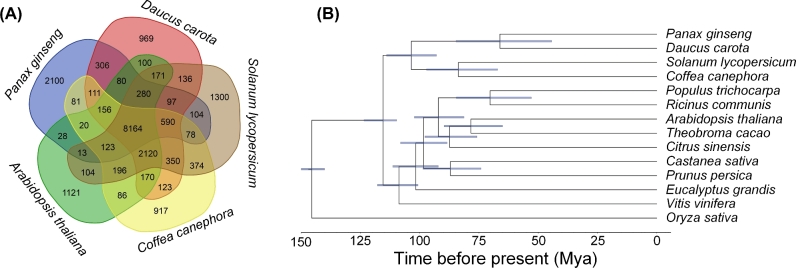
*P. ginseng* genome assembly and functional gene annotations. **(a)** Phylogenetic tree and divergence data of 14 species, including *P. ginseng*, based on the proteins of 383 single-copy genes annotated to the genome sequence of each species. **(b)** Distribution of orthologous gene families in *P. ginseng* and 4 sequenced species: carrot (*Daucus carota*), coffee (*Coffea canephora*), *Arabidopsis* (*Arabidopsis thaliana*), and tomato (*Solanum lycopersicum*).

Ortholog analysis of *P. ginseng* was conducted using 13 other plants (Supplementary Table S9). More than 75% of the gene models in *P. ginseng* were classified into 12 231 gene families, with 1648 unique gene families for *P. ginseng* itself (Fig. 1a). The average gene number per gene family was 2.59, which was the highest among all 14 plants. This finding indicates the occurrence of duplication events during the evolution of *P. ginseng*. A total of 383 single-copy genes were identified by ortholog analysis. We constructed a phylogenetic tree using the maximum-likelihood method. *Daucus carota* from Umbelliferae was found to be the closest relative of *P. ginseng* among all the compared species, diverging approximately 66 million years ago (Fig. 1b), which further supports the relative evolutionary relationships between *Daucus carota* and *Panax ginseng* [32], as well as the prevailing hypothesis of seed plants’ phylogeny [33].

### Metabolism and transcriptome of the ginseng root

**Figure 2:**
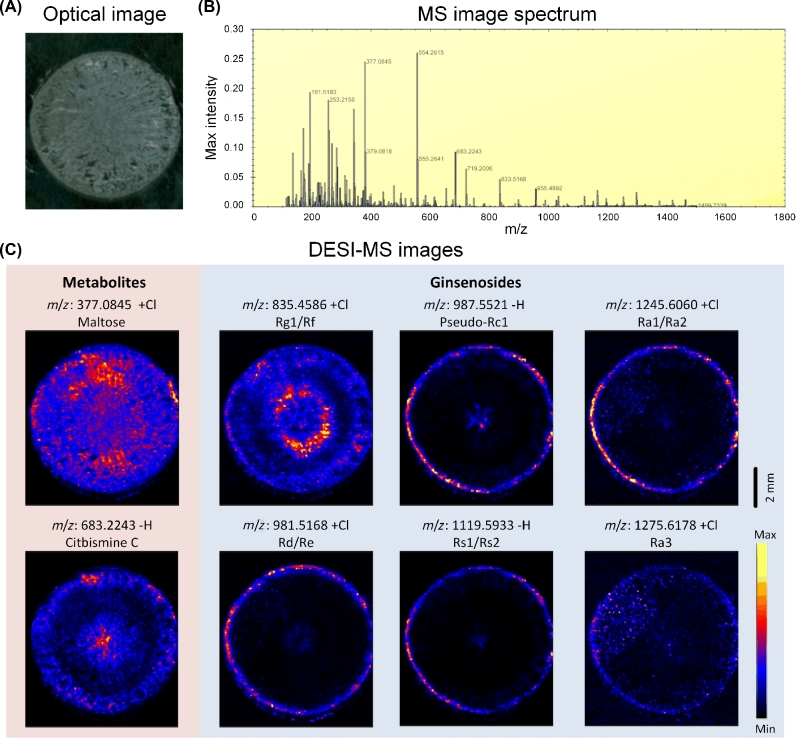
Ginsenoside distribution in the *P. ginseng* root cross-sections that were obtained through mass spectrometric imaging based on the DESI–MS. **(a)** Optical image of the main root. **(b)** Tetramethylsilane (TMS) image spectrum. **(c)** DESI–MS image of metabolites and ginsenosides: maltose, citbismine C, Rg1/Rf, pseudo-Rc1, Ra1/Ra2, Rd/Re, Rs1/Rs2, and Ra3. Scale bar = 2 mm.

Desorption electrospray ionization mass spectrometry (DESI-MS) imaging was used to elucidate the spatial distribution of ginsenosides within the ginseng root sections. Ginsenosides Rg1/Rf, pseudo Rc1, Ra1/Ra2, Rd/Re, Rs1/Rs2, and Ra3 were identified and summarized (Fig. 2b; Supplementary Table S10). Ginsenosides Rg1/Rf were highly concentrated within the outer bark and inner core areas of the root. Rd/Re Rs1/Rs2, Ra1/Ra2, and pseudoginsenoside Rc1 were distributed at high concentrations in the bark and at low concentrations in the center (Fig. 2c). Ginsenoside Ra3 exhibited a diffuse distribution within the cross-section and a high concentration around the bark (Fig. 2c). These isomers were distinguished by DESI-tandem mass spectrometry (MS/MS). For Rf/Rg1, fragmentation of the monosaccharide group C_6_H_10_O_5_ (162.05 Da) and disaccharide group C_12_H_22_O_11_ (342.12 Da) produced fragments at *m/z* 637.46 and 457.15, which corresponded to different spatial distributions (Supplementary Figure S4). The characteristic MS/MS transitions were *m/z* 603.08 for Rd and *m/z* 799.52 for Re (Supplementary Figure S5). The enrichment of Rb1 around the bark was also confirmed through DESI-MS/MS (Supplementary Figure S6).

**Figure 3:**
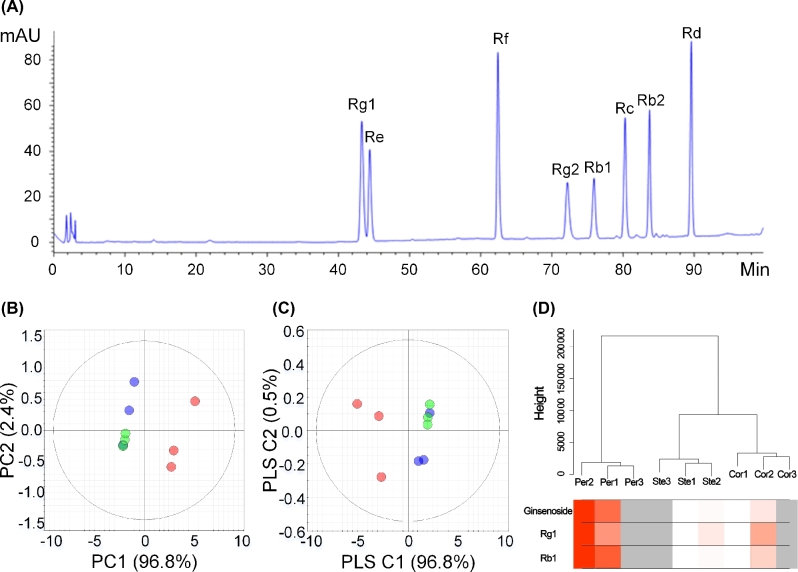
Metabolism and transcriptome analysis of *P. ginseng* root. **(a)** HPLC chromatograms of the ginsenosides Rg1, Re, Rf, Rg2, Rb1, Rc, Rb2, and Rd standards. **(b)** PCA score plots based on the HPLC dataset (red circles indicate periderm, black circles indicate cortex, and green circles indicate stele). **(c)** PLS-DA score plots based on the HPLC dataset. **(d)** Cluster tree of the ginseng samples based on the expression pattern of 42 006 genes. The leaves of the tree correspond to the different ginseng tissue samples (Per: periderm; Cor: cortex; Ste: stele). The color bands beneath the tree represent the relative content of the total ginsenosides, Rb1 and Rg1 (red indicates high values).

On the basis of anatomical characteristics, we categorized the ginseng main root into periderm, cortex, and stele for further quantitative analysis (Supplementary Figure S7). High-performance liquid chromatography (HPLC) results showed that the contents of ginsenosides Rg1, Re, Rf, Rg2, Rb1, Rc, Rb2, and Rd were significantly higher in the periderm (*P* < .001) than in the cortex and stele (Fig. 3a; Supplementary Table S11). The principal component analysis (PCA) and partial least squares discrimination analysis (PLS-DA) plots showed the distinct clustering among the periderm, cortex, and stele groups (Fig. 3b and c). The findings suggest the different distribution of ginsenosides.

More than 34 000 predicted genes were detected from the transcriptome data. Among these genes, 27 450 were expressed in the 3 sections, and 7456 genes were not detected in any section. The samples were clustered into 3 distinct groups by expression profile. The expression pattern of genes in the cortex was closer to the stele than to the periderm (Fig. 3d). A total of 2530, 2688, and 711 differentially expressed genes were found between the periderm and cortex, the periderm and stele, and the cortex and stele, respectively. GO enrichment analysis showed that differential genes between the periderm and cortex, as well as the periderm and stele, were mainly associated with metabolic processes and response to stimuli (Supplementary Figure S8). All genes were grouped into 64 modules through weighted gene coexpression network analysis (WGCNA). The total ginsenoside content was considered the weighted factor, and 3 of the modules were positively correlated with ginsenosides. The most correlated module contained 15 762 genes, indicating the complex mechanisms involved in ginsenoside synthesis and regulation (Supplementary Figure S9).

### Conserved biosynthesis pathway of ginsenosides

**Figure 4:**
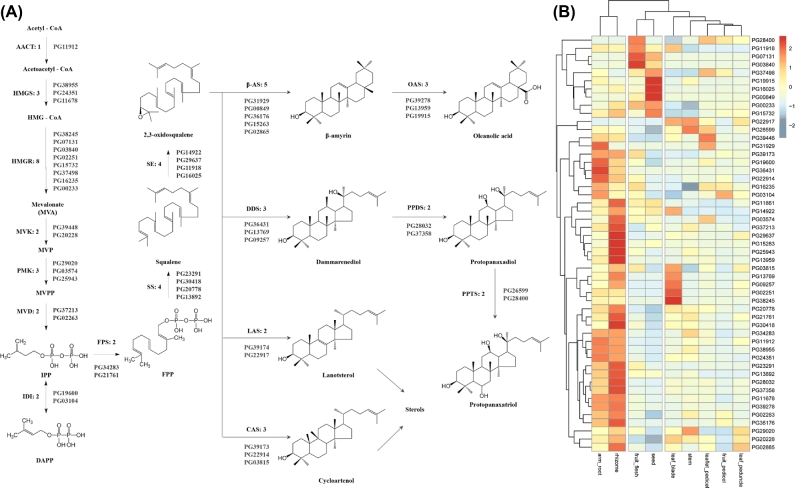
Gene expression in the MVA pathway for ginsenosides in *P. ginseng*. **(a)** Possible biosynthesis pathway for ginsenosides with the designated candidate genes. AACT: acetyl-CoA C-acetyltransferase; HMGS: 3-hydroxy-3-methylglutaryl-CoA synthase; HMGCoA: 3-hydroxy-3-methylglutaryl-CoA; HMGR: 3-hydroxy-3-methylglutaryl-CoA reductase; MVK: mevalonate kinase; MVP: mevalonate phosphate; PMK: phosphomevalonate kinase; MVPP: diphosphomevalonate; MVD: mevalonate diphosphate decarboxylase; IPP: isopentenyl diphosphate; DMAPP: dimethylallyl diphosphate; IDI: isopentenyl-diphosphate delta-isomerase; FPS: farnesyl diphosphate synthase; FPP: farnesyl diphosphate; SS: squalene synthase; SE: squalene epoxidase; β-AS: β-amyrin synthase; DDS: dammarenediol synthase; LAS: lanosterol synthase; CAS: cycloartenol synthase; OAS: oleanolic acid synthase; PPDS: protopanaxadiol synthase; PPTS: protopanaxatriol synthase. **(b)** Heatmap of the candidate biosynthesis pathway gene expression patterns in 9 organs from *P. ginseng*.

As triterpenoid saponins, ginsenosides are mainly biosynthesized using the precursor IPP produced through the MVA pathway, which includes conserved enzymes in eukaryotes [11, 34, 12]. In this study, 31 genes encoding 10 upstream enzymes were identified by BLAST search and motif finding (Fig. 4a). Except for acetyl-CoA C-acetyltransferase (AACT), all of these 10 enzymes displayed multiple copies and isoforms; 5 enzymes (8 in 3-hydroxy-3-methylglutaryl-CoA reductase [HMGR], 4 each in squalene synthase [SS] and squalene epoxidase [SE], and 3 each in phosphomevalonate kinase [PMK] and 3-hydroxy-3-methylglutaryl-CoA synthase [HMGS]) had multiple copies and isoforms. One of the PMKs may be a potential pseudogene, with several termination codons dividing the coding regions. The 4 other enzymes (mevalonate kinase [MVK], mevalonate diphosphate decarboxylase [MVD], isopentenyl-diphosphate delta-isomerase [IDI], and farnesyl diphosphate synthase [FPS]) possessed 2 copies each. Such common occurrence of the multicopy phenomenon in ginseng MVA enzymes may be associated with the diverse regulatory control of triterpenoid or steroid biosynthesis in the plant. After the formation of 2 3-oxidosqualenes, different ginsenoside precursors are cyclized and hydroxylated by various enzymes. In this assembly, 5 beta-amyrin synthases (beta-ASs), 3 oleanolic acid synthases (OASs), 3 dammarendiol synthases (DDSs), 3 PPDSs, and 3 PPTSs were identified. In addition, 100 terpenoid synthases were annotated, including 1 lanosterol synthase (LAS) and 1 cycloarstenol synthase (CAS) for ginseng sterol precursor cyclization.

The transcriptomes of 9 released RNA-Seq data (arm root, rhizome, stem, leaf blade, leaflet pedicel, leaflet peduncle, fruit pedicel, seed, and fruit flesh) [35] were used for the expression analysis of ginsenoside biosynthesis upstream genes. Two organs from the subterranean part were grouped into 1 clan. By contrast, the aerial parts were grouped into another clan (Fig. 4b). The samples, fruit flesh and seed, were relatively privileged, possibly because of their singleness as reproductive organs. Some genes were coexpressed in different organs. For example, PG07131 (HMGR), PG03840 (HMGR), PG11918 (SE), and PG28400 (PPTS) were particularly expressed in the fruit flesh sample but not in the other tissues. Meanwhile, PG19915 (OAS), PG16025 (SE), PG00849 (beta-AS), and PG37498 (HMGR) were coexpressed in the seed. In leaf blade, PG02251 (HMGR), PG38245 (HMGR), PG13769 (DDS), PG09257 (DDS), and PG03815 (CAS) were higher expressed. On the basis of hierarchical cluster analysis, the upstream genes were clustered into different groups with specific expression patterns. This pattern may be related to the organ-specific chemical distribution of ginseng (Fig. 4b).

### Sequence analysis of the *P. ginseng* HMGR (PgHMGR) family

**Figure 5:**
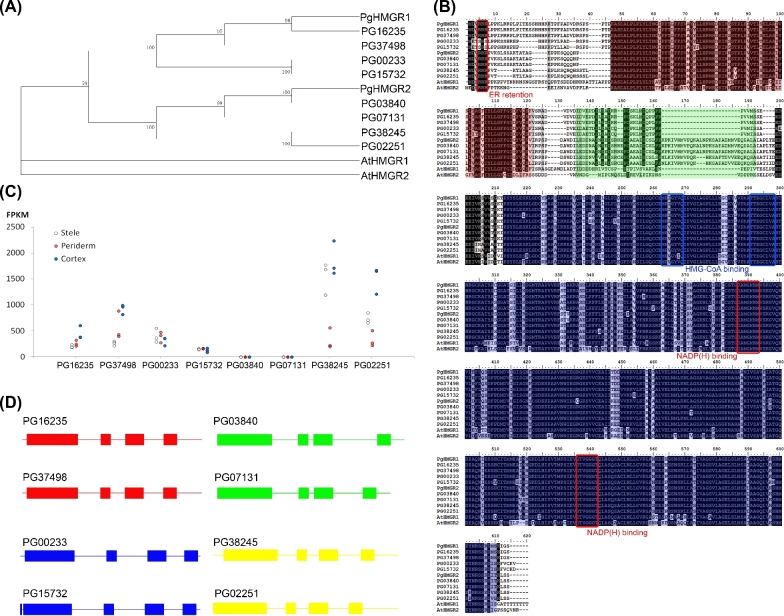
Sequence analysis and transcript levels of the HMGR gene family. **(a)** Phylogenetic analysis of PgHMGRs and characterized HMGRs from other plants. **(b)** Multiple alignments of the amino acid sequences of PgHMGRs with homologous HMGRs from *Arabidopsis*. The black boxes indicate identical residues, and the gray boxes represent identical residues for at least 2 of the sequences. Functional domains are highlighted in colored boxes (red, membrane domain; green, linker domain; and blue, catalytic domain). The 2 putative HMGR-CoA-binding sites, 2 NADP(H)-binding sites, and ER retention motifs are denoted by square boxes. **(c)** Tissue-specific PgHMGR expression patterns in 4-year-old roots. The data represent the mean ± SD of the 3 independent samples. **(d)** Genomic DNA structure of PgHMGRs. The exons are represented by the green-filled square boxes. The lines between the boxes correspond to the introns. The numbers above the exons indicate the length in bp.

HMGRs catalyze the conversion of HMG-COA into MVA, which has been considered the first committed step of ginsenoside synthesis. Eight HMGR-encoding genes were annotated. The full length of these genes was achieved by manual curation. Four of these genes showed high similarity to previously reported PgHMGR1 (with an average similarity of 94.25%), and the other 4 genes showed similarity to PgHMGR2 (with an average similarity of 93.26%) (Supplementary Table S12). Given the primary structure of putative peptide sequences, the 8 PgHMGRs were further grouped into 4 subfamilies, namely, PgHMGR1.1 (PG16235, PG37498), PgHMGR1.2 (PG00233, PG15732), PgHMGR2.1 (PG03840, PG07131), and PgHMGR2.2 (PG38245, PG02251) (Fig. 5a). The PgHMGR1 family attained relatively shorter lengths, with 573 amino acids (aa) for HMGR1.1 and 565 aa for HMGR1.2. By contrast, the PgHMGR2 family revealed relatively long lengths, with 594 aa for HMGR2.1 and 589 aa for HMGR2.2 (Fig. 5b). Most of the PgHMGR-encoding genes (except PG15732) contain 4 exons and share the same exon phase pattern with the combination “0–2-1–0.” The PgHMGR2 family was 63 bp longer than PgHMGR1 in the first exon region, but both families were roughly the same in size as the 3 other exons. The introns among the PgHMGR-coding genes fluctuated more than did the exons. Among the introns, the second intron varied the most, with a standard variation of 187 bp (Fig. 5d).

The deduced PgHMGRs were highly conserved at the C-terminal for MVA catalysis but were divergent at the N-terminal for membrane anchoring. Similar to most plants, all of the PgHMGRs contained a membrane anchor domain with a typical helix–loop–helix structure, a linker region for connection, 2 HMG-COA-binding motifs (MP(I/V)GY(I/V)QIP and TTEGCLVA), and 2 NADPH-binding motifs (DAMGMNM and GTVGGGT) (Fig. 5b). Therefore, the functional sites of all HMGRs were composed of similar residues, especially in the core region containing catalytic domains. Differences mainly located at the N-terminal were responsible for HMGR subcellular localization (Supplementary Table S13). All the deduced proteins, except HMGR1.2 (PG00233 and PG15732), attained a triple consecutive arginine region. This characteristic was implicated for endoplasmic reticulum retention. The expression patterns of different HMGR types differed among various organs (Figs 4b and 5c). Base on the calculation of fragments per kilobase of exon model per million mapped reads (FPKM), the HMGR1 family expressed more stably with an average FPKM CV of 81.97% and an average extreme deviation of 1107.81. Meanwhile, HMGR2 attained an average CV of 162.57% and an average extreme deviation of 5652.10, which was about 5 times higher than that of the HMGR1s (Supplementary Table S14). The HMGR2s were distinct among the tissues. Similar to PG07131 and PG03840, HMGR2.1 was highly expressed in fruit flesh and seed, but rarely in all other tissues (Supplementary Table S14). The excessive deviation of PG07131 reached 13 976.63, showing extreme tissue specificity (Supplementary Table S14). The 2 members of the HMGR2.2 family were prevalently expressed in leaf blades and highly expressed in the roots (Supplementary Table S14). Analysis of the expression patterns of HMGRs indicated that they may perform different task assignments in ginseng development.

### UGTs of *P. ginseng*

**Figure 6:**
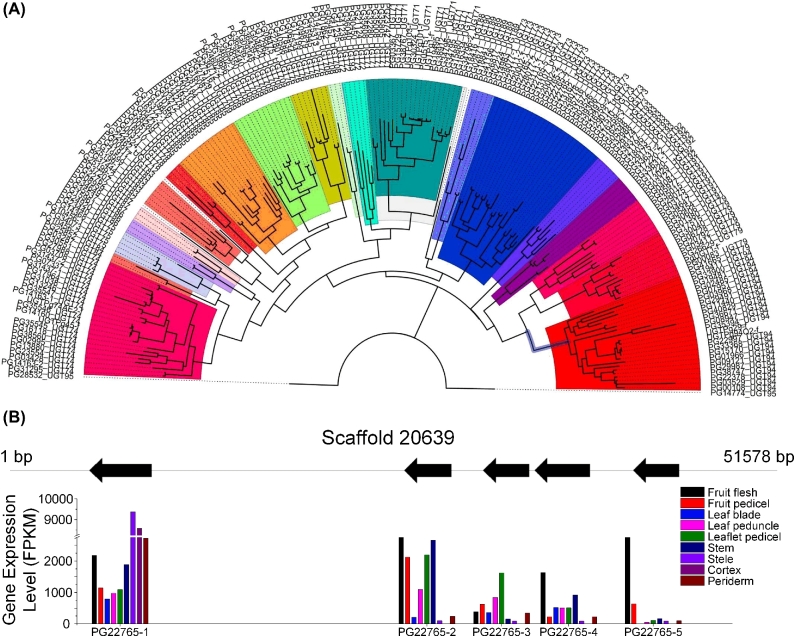
Analysis of UGTs from *P. ginseng*. **(a)** All the identified UGTs, which were newly classified according to the standardization of the UGT Nomenclature Committee, were assigned to 24 subfamilies. **(b)** The expression (lower) of UGT gene copies (PG22765) from the same scaffold (upper) in the different tissues of *P. ginseng*.

UGTs are in charge of transferring glycosyl moieties to acceptor molecules, including ginsenosides. The ginseng genome encodes a large, diverse set of UGTs. A total of 225 UGTs were identified, accounting for one of the largest gene families in ginseng. The length of these putative UGTs ranged from 74 aa to 575 aa. Moreover, the predicted isoelectric point ranged from 4.45 to 9.54. The identified UGTs were newly classified according to the standardization of the UGT Nomenclature Committee. As a result, all the UGTs were assigned to 24 subfamilies (Fig. 6a). UGT73 was the most abundant group (with 30 members), followed by UGT74 and UGT94 (with 25 and 24 members, respectively). Compared with *D. carota*, UGT74 and UGT71 notably expanded, whereas UGT93 largely shrank. Seventy-eight UGTs were found to be physically clustered into 30 groups, and the largest group contained 5 members. The PgUGTs were clustered similarly to tandem repeats and generally belonged to the same subfamily. Similar to the largest cluster, all the members originated from an ancestral UGT73, with similarity ranging from 48% to 92%. The high similarity indicated that these genes may have evolved from recent genome duplications or newly unequal recombination events.

The expression module of UGTs also showed high tissue specificity. Similar to the mentioned gene cluster, the expression patterns of these UGTs considerably differed, although all of them originated from the same gene family (UGT73) (Fig. 6b; Supplementary Figure S10). PG22765–1 was the most highly expressed member, with an average FPKM of 3089, and was the only highly expressed gene in the root, followed by PG22765–2 with an average FPKM of 1957. Meanwhile, PG22765–5 was the most fluctuant gene, with a CV of 186.72. This UGT was rarely expressed in the organ root, stem, or leaf but highly expressed in the fruit. Hence, even UGTs that belong to the same family or located closely showed a distinctly regulated gene expression.

For functional analysis, 18 UGTs from families 71, 74, or 94 were chosen for molecular modeling and docking. The models of PPD and PPT were selected as docking substrates, and UGT-Glc was selected as a sugar donor. The N-terminal I/V-G/S-H motif, the C-terminal W-N-S-X-L-E motif, and the C-terminal Y-G/A-E-Q motif of the UGT71 family; the N-terminal motif Q-G-H-X-N/S and the C-terminal H-C/S-G-W-N-S-T-X-E motif of the UGT74 family; and the N-terminal H/Q/Y-G-H motif and the C-terminal D-Q motif of the UGT94 family were predicted to bind specifically to the sugar acceptors (Supplementary Figure S11). The results showed that the key residues in the N-terminal may have been subject to selection pressure during evolution for a particular substrate binding.

## Discussion

Herb genomics has been proposed as a global platform for securing the synthesis pathways of bioactive compounds [36, 37]. This manuscript presents the genome of *P. ginseng*, which is a commercially important representative of these herbs. The assembly confirmed the previous per-haploid-genome estimation of *P. ginseng* at approximately 3.5 Gb. Second only to *Ginkgo biloba*, ginseng harbors the largest genome among the sequenced medicinal plants [38]. Detailed structural analysis revealed that more than 62% of the genome consisted of repeats. This value is the highest among those of all sequenced angiosperms, similar to orchid (61%) and higher than sorghum (58%), grape (49%), and rice (35%) [39, 40]. LTRs are a key factor in genome expansion. In *P. ginseng*, LTRs accounted for 52% of the genome, which is 1.5-fold higher than a previous estimation using bacterial artificial chromosomes (BACs) [31]. As whole-genome sequences possess more information than BACs, this divergence may be ascribed to the methodological differences. The result further emphasized the importance of whole-genome sequencing in the analysis of repeats and species evolution.

Compared with traditional chromatography methods, DESI-MS enables the exploration of secondary metabolite distribution in tissues and even in cells. The resolution of DESI-MS typically reaches 100 μm or higher [41]. The spatial distribution images can show the continuous changes of ginsenosides in the ginseng root cross-sections. These findings are expected to contribute to the screening of the physiological role, transport process, and accumulation of ginsenosides during ginseng growth and development, as well as in defense reactions, as responses to environmental challenges. DESI-MS can directly analyze isomeric compounds *in situ* [42]. Imaging ginsenosides by mass spectrometry confirmed the spatial maldistribution of ginsenosides. The data, hence, provided evidence for further gene expression analysis. Furthermore, some ginsenosides accumulated in the root center, suggesting multiple sources of ginsenoside supply (Fig. 2). Schramek et al. found by ^13^C-label tracing that the precursor units of ginsenosides are transferred from the leaves to the roots [43]. However, the mechanism underlying this long-distance transport and allocation remains unknown. Kim et al. speculated that ATP-binding cassette transporters or multidrug and toxic compound extrusion transporters may be involved in the transport process [44]. In this present research, more than 4000 transporters, including 331 ABC superfamily transporters and 71 MATE transporters, were identified. This newly obtained sequence information should facilitate future biochemical studies on ginsenoside transport.

The IPP for ginsenoside biosynthesis is generally produced via the MVA route. However, inhibition assays indicated that the methylerythritol phosphate (MEP) pathway compensated for IPP production when MVA was blocked. The MEP pathway is initiated by condensation between D-glyceraldehyde-3-phosphate and pyruvate by 1-deoxy-D-xylulose 5-phosphate synthase (DXP synthase, DXS). The pathway then terminates with the conversion of 4-hydroxy-3-methyl-butenyl 1-diphosphate (HMBPP) into IPP or dimethylallyl diphosphate (DMAPP) by isoprenoid synthase-containing protein H (IspH). In ginseng, the putative proteins involved in the MEP pathway were found to include 9 DXSs, 4 DXRs (DXP reductoisomerase), 2 IspDs, 4 IspEs, 5 IspFs, 4 IspGs, and 5 IspHs (Supplementary Figure S12). Similar to that in the MVA pathway, the members of the MEP route share a common multicopy phenomenon. Gene duplication was usually followed by functional divergence and metabolite diversity. As a result, certain ginsenosides or genes accumulated in different organs or tissues. Hitherto, this correlation has been largely unappreciated. Kim et al. cloned 3 SQSs based on ginseng expression sequence tags (ESTs) and reported their expression preferences [45]. Kim et al. found 2 copies of HMGR in ginseng and speculated that PgHMGR1 plays a general role in secondary metabolite production, whereas PgHMGR2 may be related to age-dependent ginsenoside accumulation in the root [46]. In the present study, up to 8 PgHMGRs were encoded by ginseng genomes. Of these PgHMGRs, 4 belong to the HMGR1 family and 4 belong to HMGR2 family. Each family can be grouped into 2 subfamilies. The expression of PgHMGR2s fluctuated more than PgHMGR1s among organs or tissues in ginseng. This result suggests that PgHMGR2 may conduct regulatory roles in terpene/phytosterol production during ginseng development. These results imply that the presence of multiple isoforms in the MVA/MEP route may contribute to flexible production or regulation of triterpene biosynthesis.

The glycosylation of triterpenes may increase their water solubility and modify their biological activities. In ginseng, UGTs are necessary to ginsenoside biosynthesis because they transfer monosaccharides to triterpene aglycones at C-3, C-6, or C-20 for the PPD- or PPT-type ginsenosides [44]. UGTs belong to a large and diverse gene family and can recognize a wide range of natural compounds as acceptor molecules. Triterpene glucosyltransferases belong to the UGT families 71, 73, 74, and 94 [20, 26]. These families are the most abundant UGT families in ginseng. Compared with other plants, triterpene glucosyltransferases were enriched in the ginseng genome during its evolution. This enrichment can partially account for the diversification of ginsenosides. Eighteen UGTs from UGT 71, 74, and 94 were selected for molecular modeling and docking. The results indicated that these UGTs were conserved in a 3D structure and that they displayed a general regiospecificity but not tight substrate specificity. This finding can be further confirmed by the report of Wei et al., which found that certain UGTs can modify both PPD- and PPT-type ginsenosides *in vitro* [27]. We have cloned and prokaryotically expressed a putative UGT gene of ginseng with only 1 synonymous mutation to previously reported PgUGT94Q2 [25]. Normally, the functional assay of this gene is the same as reported; this gene can catalyze the conversion of ginsenoside Rh2 into ginsenoside Rg3 and that of ginsenoside Rf2 to ginsenoside Rd (Supplementary Figure S13). Further biochemical experiments are required for other candidate tetracyclic triterpene UGTs.

This research has provided the genome sequence of *P. ginseng*, and the pathways for the synthesis of ginsenosides have been examined and described. Multiple copies of the MVA pathway and the fully described UGTs demonstrate the importance of whole-genome sequencing, while the knowledge of the specific expression of the isoform of MVA enzymes and the expansion of particular members of UGTs has expanded the understanding of the regulation of ginsenoside biosynthesis. This research will contribute to ginseng breeding, cultivation, and synthesis biology, and it provides an effective resource for plant functional genomic analysis with increased throughput, precision, and sensitivity [47].

## Methods

### Genome sequencing and assembly

Genomic DNA was extracted from the 4-year-old *P. ginseng* line IR826, a strain cultivated by the Institute of Chinese Materia Medica. Five libraries with insert sizes ranging from 250 bp to 10 kb were constructed. Except for the 2-kb mate-paired library, all the libraries were constructed using the commercial library prep kits (Vazyme Biotech, Nanjing, China). The 2-kb mate-paired library was constructed usingthe Cre/loxP recombination system, and the adapter was changed to 5’-CGTAATAACTTCGTATAGCATACATTATACGAAGTTATACGA-3’. After assembly, the average estimated span distance of the 10-kb library was about 7.5 kb; we speculated that the shrinkage was due to the molecule disruption during library preparation (Supplementary Table S1). We performed paired-end sequencing on the HiSeq X-Ten platform (Illumina) and produced 391.46 Gb of raw data (Supplementary Table S1). The genome size was estimated through the flow cytometry (BD Biosciences, Franklin Lake, NJ, USA) analysis and K-mer distribution. The reads were filtered using a skewer [48] with the following criteria: trimming a 3^΄^-end base to achieve quality >30 and exclusion of short-insert library reads (250 and 500 bp) with a read length <100 bp or average quality <30. For large-insert library reads (2–10 kb), transposase adapter sequences were used for adapter searching and trimming, and the max mismatch rate was set to 10%. Trimming reads from the 3’ end till Q>20 after trimming and reads with a read length <18 bp or average quality <30 were filtered out. Finally, 315.93-Gb reads were retained for genome assembly (Supplementary Table S1) through SOAPdenovo2 (SOAPdenovo2, RRID:SCR_014986) [49]. K-mer size selection was performed using KmerGenie [50] with a 250-bp insert library and a recommended 83-mer; then k-mer sizes of 63, 73, 83, and 93 were used for assembly with default parameters, and the optimal k-mer size (k = 83) was selected based on the N50 length in each k-mer size. The reads from the small-insert libraries were used for contig construction to assemble the *P. ginseng* genome. The read pairs from the small- and large-insert libraries were then utilized to join the contigs into the scaffolds. Further scaffolding was performed using the large-insert libraries with SSPACE (SSPACE, RRID:SCR_005056) (Configuration files in Supplementary Text) [51]. Finally, the small-insert libraries were used for gap closure of the scaffolds using GapCloser (GapCloser, RRID:SCR_015026) [52]. In order to annotate genes, scaffolds with a length of less than 1000 bp were filtered out, which also may cause some lost content.

The 2 short-insert library reads were aligned onto the assembly through BWA (BWA, RRID:SCR_010910) with default parameters to evaluate the assembly quality [53]. We performed the BUSCO v2 (BUSCO, RRID:SCR_015008) analysis [30] with the recently released plant dataset from OrthoDB v9.1 (OrthoDB, RRID:SCR_011980) [54] to test the completeness of the scaffolds. A total of 75 878 transcripts assembled from RNA-Seq dataset (the assembly process was described in the “Transcriptome sequencing and analysis” section) were mapped back to the draft genome using BLAST (BLASTN, an identity cutoff value of 90% and a coverage cutoff value of 90%) [55].

### Ginsenoside distribution and content analysis

The main roots of Da-Ma-Ya (a local cultivar of ginseng) were used for metabolome analysis and transcriptome analysis. The frozen transverse sections of the ginseng main root with 20-μm thickness were prepared using a microcryotome for DESI-MS imaging. The ginsenoside distribution was evaluated on a Xevo G2-XS Tof mass spectrometer with the DESI source (Waters Corporation). The MS images were created by spraying N_2_ gas-focused solvent stream directly onto the sample to produce the MS spectra from the surface, which was then rastered across the sample at regular intervals to build a 2D image. Image creation was performed using high-definition imaging (HDI) software (Waters Corporation) with the following parameters: X and Y pixel size 100 μm; raster speed 400 μm/s; spray solvent 90% MeOH, 10% H_2_O, 0.1 mM NH_4_Cl, and 0.1 mM leucine enkephalin delivered at 1.5 μL/min; MS at negative polarity, 4.5 kV capillary voltage, 80 V cone voltage, and mass range *m/z* 100–1200. The MS images were created from raw MS files through HDI with leucine enkephalin as the lockmass (*m/z* 554.2615) for high-resolution MS. The DESI-MS/MS images were created for ginsenoside Rf/Rg1 (*m/z* 799.48, −H adduct) and ginsenoside Rd/Re (*m/z* 945.54, −H adduct), and collision energy from 10–40 (arbitrary units).

The 3 independent ginseng root samples were divided into 3 portions: periderm, cortex, and stele, which were crushed and mixed with methanol containing 0.1% methanoic acid. The mixture was frozen for 1 hour and then centrifuged. The upper layer was collected, filtrated, and transferred to a sample vial to be injected and analyzed by HPLC for ginsenoside content measurement.

### Transcriptome sequencing and analysis

The total RNA was extracted from the periderm, cortex, and stele using TRIzol® Reagent (Invitrogen, Carlsbad, CA, USA) to construct a sequencing library. The RNA-Seq transcriptome libraries were prepared following the TruSeq^TM^ RNA sample preparation kit (Illumina). mRNA was isolated with polyA selection by oligo (dT) beads and fragmented using a fragmentation buffer. Generally, cDNA synthesis, end repair, A-base addition, and the ligation of the Illumina-indexed adaptors were performed according to Illumina's protocol. The libraries were selected based on the size of the cDNA target fragments of 200–300 bp, followed by polymerase chain reaction (PCR) amplification using Phusion DNA polymerase (New England Biolabs, USA) for 15 PCR cycles. After quantification, the paired-end libraries were sequenced by HiSeq X-Ten (Illumina).

Raw reads generated by RNA-seq of different parts of ginseng root were trimmed and quality controlled by Skewer with the following parameter: adapter sequences searching and trimming with 10% max mismatch rate, trimming reads from the 3’ end till Q>20, trimmed reads with read length <100 bp or average quality <30 were filtered out. Thereafter, Trinity software with default parameters were applied for *de novo* assembly. The total length of 75 878 assembled transcripts is 70 273 566 bp; max, min, and N50 length are 12 639 bp, 201 bp, and 1446 bp, respectively. The clean reads were separately aligned to the *P. ginseng* genome in the orientation mode using TopHat (TopHat, RRID:SCR_013035) [56, 57]. For comparing the gene expression pattern among the different tissues of *P. ginseng*, 6 other tissue RNA-Seq datasets from NCBI (accession number SRP066368) were analyzed [35]. The expression level for each transcript was calculated using the fragments per kilobase of exon per million mapped reads (FPKM) method to identify differentially expressed genes (DEGs) among the different samples. Cuffdiff (Cuffdiff, RRID:SCR_001647) [58, 59] was used for the differential expression analysis. The DEGs were selected using the following criteria: the logarithm of the fold change >2 and the false discovery rate (FDR) <0.05. GO functional enrichment and KEGG pathway analyses were performed using Goatools [60] and KOBAS [61], respectively, to understand the function of DEGs [62]. DEGs were significantly enriched in GO terms and metabolic pathways when their Bonferroni-corrected *P-*value was <0.05. The hierarchical clustering analysis of the expression profiles was performed using the hclust command in R and the default complete linkage method. The R package WGCNA [63] was used to identify the co-expression modules.

### Repeat detection, gene prediction, and annotation

We detected the repeat content of the *P. ginseng* genome through an approach combining *de novo* prediction and homology-based searching. Three *de novo* prediction programs, namely PILER-DF v1.0 [64], RepeatModeler (RepeatModeler, RRID:SCR_015027) v1.0.8 [65], and LTR_FINDER v1.06 [66], were used to construct the *de novo* repeat library. The homology-based approach involves searching commonly used databases of known repetitive sequences. RepeatMasker v4.06 [65] was used for the DNA-level identification with Repbase (a database of eukaryotic repetitive elements) using RepeatMasker v4.06 [65], and RepeatProteinMask was utilized for protein level identification, which ran WuBlastX against the TE protein database. The tandem repeats in the genome assembly were identified through the tandem repeat finder.

The gene models of the *P. ginseng* genome were predicted using the MAKER-P pipeline [67]. The available ginseng EST, mRNA datasets, and protein datasets were used to generate the first-pass gene annotation. The resulting GFF3 file was used for *ab initio* gene predictor SNAP training [68]. The 75 878 transcripts assembled from the RNA-Seq data were used as transcript clues for the second-pass MAKER-P annotation. For further gene function annotation, the transcript encoding the longest protein sequence for each gene was defined as the representative sequence. First, each protein was searched against the NR [69], KOG [70], and Swiss-Prot [71] databases using BLASTx. The best similar hit with an E-value <1.0e−5 was considered the gene annotation information. Second, each protein was annotated according to the GO database [72], and Blast2GO (Blast2GO, RRID:SCR_005828) was used to obtain GO terms representing a biological process, cellular component, and molecular function. Finally, all proteins were searched against the KEGG database with the KAAS tool [73, 74]. Multiple plant organisms were selected to obtain the KEGG ortholog IDs of the best homologous genes.

### Gene family identification and phylogenetic analysis

Thirteen other diploid plant genomes were used for cluster identification to determine the ortholog genes and to elucidate the evolution of the genome, in addition to the *P. ginseng* genome (Supplementary Table S9). The longest representative sequence of each genome under the pairwise sequence similarities among all input proteins was calculated using an all-by-all BlastP with an E-value 1e−10, which was used to cluster the genes by OrthoMCL (OrthoMCL DB: Ortholog Groups of Protein Sequences, RRID:SCR_007839) [75]. The peptide sequences from 383 single-copy orthologous gene clusters were extracted to construct a phylogenetic tree and estimate the divergence time. After the multiple sequence alignment by MUSCLE (MUSCLE, RRID:SCR_011812) [76] and the poorly aligned region removal by GBLOCKs [77], the high-quality blocks were converted (back-translation) in CDS and concatenated into 1 super-gene for each species. With these super-genes, a phylogenetic tree was constructed with RAxML (RAxML, RRID:SCR_006086) through the PROTGAMMAJTT model [78].

The divergence time was estimated by MCMCtree program with 10 000 sampling times, 50 sampling rate, and 50 000 iteration burn-ins [79]. Two runs were performed to ensure convergence. The divergence time between monocots–dicots (140–150 Mya) or Arabidopsis–tomato or grape–tomato (110–124 Mya) was used to calibrate the divergence time [80, 81]. Four species were selected for the lineage-specific evolutionary rate estimation with codeML through the free-ratio model. The genes with dS >3 or dN/dS >3 were filtered. Furthermore, the codeML with the branch-site model was used to estimate the branch-based ratio of nonsynonymous to synonymous substitution rate (ω or dN/dS). The branch-site model parameters were set as follows: null hypothesis: model = 2, NSsites = 2, fix_omega = 1, omega = 1; alternative hypothesis: model = 2, NSsites = 2, fix_omega = 0, omega = 1.

### UGT family analysis, molecule modeling, and docking

Multiple alignments were performed using cluster X2 [82]. Phylogenetic trees were generated through MEGA 5.0 (MEGA Software, RRID:SCR_000667) [83]. The genetic distances were estimated using the pairwise distance amino acid substitution matrix with 100 bootstrap replicates.

The coordinates in pdb format of the small molecules protopanaxadiol and protopanaxatriol were built using Corina software [84]. The homology models of the 18 UGTs from *P. ginseng* were built using the crystal structures as templates searched using the Swiss-model server [85, 86]. The docking of the protopanaxadiol or protopanaxatriol and the UDP-glucose in the constructed models was performed with Patchdock [87–89]. The ligand docking results were visualized with the PyMOL molecular graphics system [90].

## Availability of supporting data and materials

Sequencing data are available via NCBI under the project number PRJNA385956. The latest versions of the genome assemblies and annotation are available through our website at http://ginseng.vicp.io:23488/. The sequencing data of genome and transcriptome and other supporting data were deposited at *Giga*DB [28].

## Additional files

Figure S1: The genome size of *P. ginseng* estimation. (a) Distribution of 17-mer frequency. In total 183.82 Gb of high-quality short-insert reads (250 bp and 500 bp) were used to generate the 17-mer depth distribution curve frequency information. (b) Flow cytometry analysis by comparison with *Glycine max*.

Figure S2: Distribution of sequence depth for the *P. ginseng* genome. The x-axis and the y-axis represent the depth and percentage of corresponding DNA bases.

Figure S3: Venn diagram of the distribution of functional annotated genes searched against the Nr, GO, KEGG, KOG, and SWISS-PROT databases.

Figure S4: DESI-MS/MS images of Rf and Rg1 in *P. ginseng* root cross-sections. (a) MS/MS spectrum of m/z 799.52. (b) Molecular structural formula of Rg1 and Rf. (c) The DESI-MS/MS images of Rg1 and Rf.

Figure S5: DESI-MS/MS images of Rd and Re in *P. ginseng* root cross-sections. (a) MS/MS spectrum of m/z 745.54. (b) Molecular structural formula of Rd and Re. (c) The DESI-MS/MS images of Rd and Re.

Figure S6: DESI-MS/MS images of Rb1 in *P. ginseng* root cross-sections. (a) MS/MS spectrum of m/z 1107.60. (b) Molecular structural formula of Rb1. (c) The DESI-MS/MS images of Rb1.

Figure S7: The root of 4 years of *P. ginseng*. (a) The microscopic section of the main root of *P. ginseng*. (b) The main roots were peeled into 3 parts roughly, including periderm, cortex, and stele.

Figure S8: The GO enrichment analysis of gene expression in ginseng root sections. The differential genes between (a) periderm vs cortex and (b) periderm vs stele.

Figure S9: Hierarchical cluster tree showing coexpression modules identified with 42 006 genes (among which 7456 genes with slight variance were excluded from the analyses) through the WGCNA. The modules corresponding to the branches are represented by colors in the first color band underneath the tree, and the remaining color bands reveal highly correlated (red) or anticorrelated (blue) transcripts for the total ginsenosides, Rb1 and Rg1. Red indicates a highly positive correlation with the corresponding gene, white denotes a weak correlation, and a blue module represents a highly negative correlation.

Figure S10: Sequencing depth of the PG22765 cluster. The alignment information (.bam file) of the Scaffold20639, which contains the PG22765 UGT gene cluster, was extracted from full alignment (mapping 500-bp library reads to draft genome using BWA-mem) and used to calculating sequencing depth. Except for the gap, repeat region, or high-GC-content area, the mapping depth was around ×20.

Figure S11: Homology docking of PgUGT23 enzyme from *P. ginseng*. (a) PgUGT23 protopanaxadiol ligand site. (b) PgUGT23 protopanaxadiol overall structure. (c) PgUGT23 protopanaxatriol ligand site. (d) PgUGT23 protopanaxatriol overall structure.

Figure S12: The putative proteins involved in MEP pathway.

Figure S13: The functional assay of this gene PG22997. (a) The electrophoretogram of SDS-PAGE for recombinant PG22997. (b, c) The reactions that PG22997 catalyzes the ginsenoside Rf2 to ginsenoside Rd and ginsenoside Rh2 to ginsenoside Rg3.

Table S1: Statistics of *P. ginseng* genome sequencing.

Table S2: Statistics of *P. ginseng* transcriptome sequencing.

Table S3: Statistics of 17-mer analysis.

Table S4: Predicting the repetitive elements.

Table S5: Categories of TEs predicted in the ginseng genome.

Table S6: Details of repetitive elements in ginseng genome.

Table S7: The transcription factors.

Table S8: The transporter superfamilies.

Table S9: Genome list used for phylogeny estimation.

Table S10: Identification of ginsenosides.

Table S11: Ginsenoside concentrations in different tissues (peridem, cortex, and stele) of roots.

Table S12: Alignment score of HMGRs by CLUSTALW.

Table S13: HMGR location prediction features analyzed by WoLF SPORT.

Table S14: PgHMGR family expression in different tissues.

## Abbreviations

AACT: acetyl-CoA C-acetyltransferase; BUSCOs: Benchmarking Universal Single-Copy Orthologs; CAS: cycloartenol synthase; CDS: coding sequence; DDS: dammarenediol synthase; DESI-MS: desorption electrospray ionization-mass spectrometry; DMAPP: dimethylallyl diphosphate; DXR: 1-deoxy-D-xylulose-5-phosphate reductoisomerase; DXS: 1-deoxy-D-xylulose-5-phosphate synthase; EST: expression sequences tags; FPP: farnesyl diphosphate; FPS: farnesyl diphosphate synthase; GT: glycosyltransferase; HMBPP: (E)-4-hydroxy-3-methyl-but-2-enyl pyrophosphate; HMGCoA: 3-hydroxy-3-methylglutaryl-CoA; HMGR: 3-hydroxy-3-methylglutaryl-CoA reductase; HMGS: 3-hydroxy-3-methylglutaryl-CoA synthase; HPLC: high-performance liquid chromatography; IDI: isopentenyl-diphosphate delta-isomerase; IPP: isopentenyl diphosphate; IPP: isopentenyl diphosphate; LAS: lanosterol synthase; LTR: long terminal repeat; MEP: 2-C-methyl-D-erythritol 4-phosphate; MVA: mevalonic acid; MVD: mevalonate diphosphate decarboxylase; MVK: mevalonate kinase; MVP: mevalonate phosphate; MVPP: diphosphomevalonate; OAS: oleanolic acid synthase; PMK: phosphomevalonate kinase; PPD: protopanaxadiol; PPDS: protopanaxadiol synthase; PPT: protopanaxatriol; PPTS: protopanaxatriol synthase; SE: squalene epoxidase; SQS: squalene synthase; SS: squalene synthase; UDP: uridine diphosphate; UGT: UDP-glycosyltransferase; WGCNA: weighted gene coexpression network analysis; β-AS: β-amyrin synthase.

## Competing interests

The authors declare no competing financial interests.

## Funding

This work is supported by the grants from the National Natural Science Foundation of China (81 403 053, 81 503 469), the China Academy of Chinese Medical Sciences (ZZ0808021), the Guangdong Provincial Hospital of Chinese Medicine Special Fund (2015KT1817), the China Academy of Chinese Medical Sciences Special Fund for Health Service Development of Chinese Medicine (ZZ0908067), and National Cancer Institute, National Institutes of Health (CA154295).

## Author contributions

C.S.L. and C.Y.C. initiated the study, designed the experiments, reviewed the data, and drafted the manuscript. C.Y., X.S.M., Y.Q.G., B.R., Z.J.J., Z.X.Y., Z.J., J.Z.W., L.Z.X., Z.L.J., C.R.Y., Z.G.W., and R.W. designed and performed the experiments. X.J., L.B.S., S.H., Q.J., W.M.L., L.G.Z., Z.L., ZhuY.J., Z.C. and Q.L.R. analyzed the data. X.J., X.S.M., C.Y., L.B.S., D.L.L., L.X.W., ZhangY.J., D.A., R.N., and H.Z.H. wrote the manuscript.

## Supplementary Material

GIGA-D-17-00036_Original-Submission.pdf

GIGA-D-17-00036_Revision-1.pdf

GIGA-D-17-00036_Revision-2.pdf

GIGA-D-17-00036_Revision-3.pdf

Response-to-Reviewer-Comments_Original-Submission.pdf

Response-to-Reviewer-Comments_Revision-1.pdf

Response-to-Reviewer-Comments_Revision-2.pdf

Reviewer-1-Report-(Original-Submission).pdf

Reviewer-1-Report-(Revision-1).pdf

Reviewer-2-Report-(Original-Submission).pdf

Reviewer-2-Report-(Revision-1).pdf

Reviewer-2_Original-Submission-(Attachment).pdf

Supplement Tables and Figures
