# Supplementary material for: Panax ginseng genome examination for ginsenoside biosynthesis
Source: Gigascience. 2017 Oct 5;6(11):1–15. doi: 10.1093/gigascience/gix093 (PMC5710592; doi:10.1093/gigascience/gix093)
Supplement: GIGA-D-17-00036_Revision-1.pdf [file gix093_giga-d-17-00036_revision-1.pdf]

GigaScience

Ginseng genome examination for ginsenoside biosynthesis

--Manuscript Draft--

**Manuscript Number:**	GIGA-D-17-00036R1	
**Full Title:**	Ginseng genome examination for ginsenoside biosynthesis	
**Article Type:**	Research	
**Funding Information:**	National Natural Science Foundation of China (81403053)	Dr. Jiang Xu
	National Natural Science Foundation of China (81503469)	Dr. Shuiming Xiao
	China Academy of Chinese Medical Sciences (ZZ0808021)	Prof. Shilin Chen
	Guangdong Provincial Hospital of Chinese Medicine Special Fund (2015KT1817)	Prof. Zhihai Huang
	China Academy of Chinese Medical Sciences Special Fund (ZZ0908067)	Prof. Shilin Chen
	National Cancer Institute (US) (CA154295)	Prof. Yungchi Cheng
**Abstract:**	Background: Ginseng, which contains ginsenosides characterized as bioactive compounds, has been regarded as an important traditional medicine for several millennia. Howerver, the genetic background of ginseng remains poorly understood partly because of the plant's large and complex genome composition.<br>Results: We report the entire genome sequence of Panax ginseng using next-generation sequencing. The 3.5 Gb nucleotide sequence contained more than 60% repeats and encoded 42,006 predicted genes. Twenty-two transcriptome datasets and mass spectrometry images of ginseng roots were adopted to precisely quantify the functional genes. Thirty-one genes were identified to be involved in the mevalonic acid pathway. Eight of these genes were annotated as 3-hydroxy-3-methylglutaryl-CoA reductases, which displayed diverse structures and expression characteristics. A total of 225 UDP-glycosyltransferase (UGTs) were identified, and these UGTs accounted for one of the largest gene families of ginseng. Tandem repeats contributed to the duplication and divergence of UGTs. Molecular modeling of UGTs in the 71, 74, and 94 families revealed a regiospecific conserved motif located at the N-terminus. Molecular docking predicted that this motif captured ginsenoside precursors.<br>Conclusion: The panorama of ginseng genome represents a valuable resource for understaning and improving the breeding, cultivation, and synthesis biology of this key herb.	
**Corresponding Author:**	Jiang Xu, PhD<br><br>CHINA	
**Corresponding Author Secondary Information:**		
**Corresponding Author's Institution:**		
**Corresponding Author's Secondary Institution:**		
**First Author:**	Jiang Xu, PhD	
**First Author Secondary Information:**		
**Order of Authors:**	Jiang Xu, PhD	
	Yang Chu, PhD	
	Shuiming Xiao, PhD	

Powered by Editorial Manager® and ProduXion Manager® from Aries Systems Corporation

	Baosheng Liao, M.D.
	Qinggang Yin, PhD
	Rui Bai, M.D.
	He Su, PhD
	Linlin Dong, PhD
	Xiwen Li, PhD
	Jun Qian, PhD
	Jingjing Zhang, PhD
	Yujun Zhang, PhD
	Xiaoyan Zhang, M.D.
	Mingli Wu, M.D.
	Jie Zhang, M.D.
	Guozheng Li, PhD
	Lei Zhang, PhD
	Zhenzhan Chang, PhD
	Yuebin Zhang, PhD
	Zhengwei Jia, PhD
	Zhixiang Liu, PhD
	Daniel Afreh, PhD
	Ruth Nahurira, PhD
	Lianjuan Zhang, M.D.
	Ruiyang Cheng, M.D.
	Yingjie Zhu, PhD
	Guangwei Zhu, PhD
	Wei Rao, PhD
	Chao Zhou, PhD
	Lirui Qiao, PhD
	Zhihai Huang, PhD
	Yungchi Cheng, PhD
	Shilin Chen, PhD
**Order of Authors Secondary Information:**	
**Response to Reviewers:**	Dear Dr. Hans Zauner,<br><br>We thank you and the reviewers for your detailed comments, which really benefit our manuscript. We have carefully considered all comments and accordingly revised our manuscript. Please find below our point-by-point replies to the comments and detailed explanations of all changes ("V1" refers to the originally submitted version and "R1" is the revised version; all revisions in R1 were tracked). Thank you!<br><br>Reviewer #1: The manuscript entitled: "Ginseng genome examination for ginsenoside biosynthesis" by Xu Jiang et al., presents results of sequencing, characterization and annotation of the P. ginseng genome. The authors have further shown the distribution of the ginsenosides molecules in root tissues using mass spectrometry and liquid chromatography, and in order to identify candidate genes possible involved in the ginsenosides biosynthesis pathway an analysis of expression and co-expression

networks using RNA-seq data was performed. A set of key genes involved in the ginsenosides biosynthesis were further characterized and interestingly some of them showed tissue specificity and activity during microbial infection. Overall, the experiments and analysis for this study were well conducted. And the results presented in this manuscript are important for the research of its field. However, the manuscript presents major faults that need to be addressed prior publication.
Reply: Thank you for your positive assessment. We have revised our work according to the comments.

Major comments
1. How old were the P. ginseng plants used for genome sequencing?
Reply: The ginseng sample we used for genome sequencing came from a 4-year-old P. ginseng plant. We have added this information in the method section of R1.

2. The Microbial-resistance section needs to be greatly improved since there is a lot of missing information:
Reply: Thank you for pointing out this deficiency. In V1, we used published data (Gao et al. 2016) to recalculate gene expression during fungus infection. Although it is important for ginseng cultivation, this section is still slightly correlated with other parts of the manuscript. Considering the two reviewers' advice, the microbial-resistance section has been removed in R1. Nevertheless, we have attempted to answer the questions below as best as we can. We are truly grateful for the reviewer's comments and suggestions.

a. The statement "Ginsenosides comprise a group of defense metabolites" needs to be supported by a reference.
Reply: Thank you for pointing this out. Indeed, this statement should be supported by some references to increase readers' understanding. Triterpenoid saponins are considered as defense compounds against pathogenic microbes and herbivores (Osbourn, 1996; reviewed by Augustin et al., 2011). A recent study also reveals that unigenes involved in ginsenoside biosynthesis play important roles in the response to Cylindrocarpon destructans infection (Gao et al., 2016).

b. What does the authors mean in the statement: "responses to microbial infection are important for ginsenoside biosynthesis"? Is the ginsenosides biosynthesis pathway lead by defense responses of the plant? Does this mean that if the plant is not under biotic stress the ginsenosides are not produced? What happen with the ginsenosides biosynthesis during C. destructans infection? Does the ginsenosides increase? Please clarify and support the statement above with a scientific reference.
Reply: Thank you for the reviewer's concerns. We obtained this viewpoint from two aspects. First, the ginsenoside biosynthesis pathway may be activated by microbial infection, and ginsenosides reportedly accumulate during C. destructans infection (Chi et al., 2016). Ginsenosides also increase after eliciting SA (Tewari et al., 2011) and MeJA (Lim et al., 2005; Lee et al., 2017). Second, some reports suppose that ginsenosides are phytoalexins and may be the evolution products for microbial resistance (Gao et al., 2016; Hu et al., 2007; Kim et al., 2015). Although we have removed this part in R1, clarifying this statement in future work is still important.

c. The authors mentioned that 1652 resistance genes were annotated base on "plant resistance gene database". Please add the reference for this database.
Reply: Thank you for pointing this out. The Plant Resistance Genes database (PRGdb; http://prgdb.org) is a comprehensive resource of resistance genes (R-genes), a major class of genes in plant genomes that conveys disease resistance against pathogens (Sanseverino et al., 2010).

d. The last paragraph on page 15 starting at "The expression analysis showed that 160 resistance genes …" till the end, it is not supported by any figure or table and it is not clear what expression analysis were performed. (FPKM?, WGCNA?, qPCR?)
Reply: We apologize for the confusion caused by this paragraph. We used a FPKM analysis result here.

e. The first paragraph on page 16 "The transcriptomes of ginseng induced by C. destructants … were recalculated". What does the authors mean by "transcriptomes were recalculated"? Did the authors sequence RNA of infected plants? All this

information is missing in the methods section.
Reply: We regret the unclear description. We used the published transcriptome read (Gao et al., 2016) mapping of our genome sequence to recalculate gene expression (FPKM value) during fungus infection. Thus, we did not sequence the RNA of infected plants by ourselves, and information was not described in detail in the method section.

f. In the next statement, starting at "A total of 35008 genes were predicted, of these genes 28481 were expressed … the initial point" has to be supported by evidence. There is not figure or table that shows this analysis. How did the authors get the genes? Did they perform an RNA-seq assembly? If it so, where are the methods and metrics of the RNA-seq assembly?
Reply: We apologize for the confusion. A total of 35 008 genes were identified using time-course RNA-seq data by Gao et al. (2016) As we stated in our reply to Question e, the RNA data were from a published paper (Gao et al., 2016). To avoid misunderstanding, we have deleted this section.

g. The statement "At 0.25 DPI, 104 resistance genes were highly expressed and were possibly involved in C. destructans recognition" needs to be supported by evidence and a reference. The level of gene expression might not be related with the pathogen recognition at all.
Reply: We completely agree with the reviewer's point. The resistance-gene expression level may be influenced by many factors, including fungus infection.

h. The next paragraph starting at "At 0.25 DPI" till "Figure S10" describes a GO enrichment test described in figure S10. However figure S10 does not show what described in the text.
Reply: We regret the unclear description. Considering the weak correlation between this microbial-resistance section and the other parts of the manuscript, we have removed this section including the GO enrichment test and Fig. S10 in R1.

i. The entire section is not well described or described at all in methods.
Reply: We agree with the reviewer's point. The other reviewer also pointed that this section is weakly related to the other parts of the manuscript. Considering both reviewers' suggestions, we have deleted the entire microbial-resistance section in R1.

1. The methods and tools in methods sections are not properly cited. For example, BLAST, WGCNA, R, etc., lack of reference. Databases such as GO, KEGG, KOG, etc., are not properly cited. The authors should cite the scientific manuscript of the tools and databases and not only the website.
Reply: Thank you for noticing these points that we ignored in V1. We have accordingly corrected these errors and added appropriate references to R1. For example, BLAST (Altschul et al., 1990), WGCNA (Langfelder and Horvath, 2008), GO (Ashburner et al., 2000), KEGG (Kanehisa et al., 2003), and KOG (Koonin et al., 2004) have been cited in R1.

2. Repeat prediction, gene prediction and annotation section in Methods. Page 26. The authors mention that transcripts assembled from RNA-seq data were use for MAKER-P annotation. However there is none information regarding to RNA-seq assembly. What assembler was used? What parameters were used? How many transcripts were obtained? Etc.,The metrics of the transcriptome assembly are not described at all.
Reply: We regret not describing this assembly step in detail. Thank you for pointing this out. We conducted RNA-seq assembly using Trinity software with default parameters and generated 75 878 transcripts, which were used for further gene annotation as RNA evidence. The total length of 75 878 transcripts was 70 273 566 bp, max, min, and the N50 lengths were 12 639, 201, and 1446 bp, respectively.

Minor comments
Page 7. Missing space: "RNA-seqlibraries"
Reply: Thank you for pointing this out. We have revised this fault in line 10, page 7 in R1.

Page 7. Please add the reference for "13 published ginseng RNA-seq data".
Reply: Thank you. The 13 published ginseng RNA-seq data came from two references (Gao et al., 2016; Wang et al., 2015). In R1, we deleted all seven RNA-seq datasets

from Gao et al. (2016) and added another two datasets from Wang et al. (2015).

Page 13. Figure 5. Please rename figures in order of citation. (5a, 5d, 5c, 5b -> 5a, 5b, 5c, 5d)
Reply: Thank you for this kind suggestion. We have revised the order of Fig. 5 in R1.

Page 14. The sentence "The expression patterns of different HMGR types differed" does not make sense. Please re-phrase.
Reply: Thank you for pointing out this error. We have replaced this sentence with “The expression patterns of dissimilar HMGR types differed among various organs” in line 13, page 14 in R1.

Page 20. Add the figure number that support this statement: "same PgHMGR subfamily, they exhibited different correlation patterns with other genes"
Reply: This statement was based on the results shown in Figs. 3b (R1) and 6b (V1). However, this sentence that was related to C. destructan infection has been deleted in R1.

Page 22. Genome sequencing and assembly. How old were the plants used in this study?
Reply: We used a 4-year-old P. ginseng plant in this study. The information has been added in the method section in R1.

Page 22. Please add the scientific reference for SOAPdenovo assembler.
Reply: Thank you for your suggestion. We have added the reference for SOAPdenovo2 assembler (Luo et al., 2012) as Reference 56 in R1.

Page 23. Last row of the first paragraph. There is missing information for the cutoff criteria. "BLAStN, a cutoff value of 90%". What is this 90%? Identity?
Reply: We meant an identity cutoff value of 90% and have clarified our meaning as “BLASTN, an identity cutoff value of 90%, and a coverage cutoff value of 90%” in lines 13–14, page 21.

Page 28. Please add the reference for PyMOL.
Reply: Thank you for this suggestion. We have added the reference for PyMOL (Seeliger and Groot, 2010) as Reference 89 in R1.

Figures:
Figure 5b. What does it show? FPKM values? Is it an qPCR? Y axis needs a label. In the figure legend the figure was described as a qPCR analysis however nothing of this is described in methods and primers sequences are not provide.
Reply: Fig. 5b shows the FPKM values. The Y-axis-label has been added to Fig. 5b in R1.

Figure S10. The legend of this figure does not provide enough information of the figure.
Reply: Thank you for pointing this out. As answered previously, we have deleted Fig. S10.

Reviewer #2: Xu Jiang et al., present a work on genome assembly and annotation of Panax ginseng, with subsequent rna-SEQ analysis and metabolite analysis of root tissues to advance the understanding of the ginsenosides' synthesis pathway. All in all this project is interesting and the current work will merit publication once a number of major comments are resolved. The text could also benefit from some re-organisation to remove duplication, and present the information and technical details in a more concise and ordered fashion. I will focus this review mainly on the sequencing aspects of the paper, with special emphasis on the genome assembly which is my field of expertise.

I feel this paper could gain a lot by tightening up the methods. This will either provide more support for the ginsenoside biosynthesis analysis in which case the focus can highlight that result or if there is no more support for that analysis then the paper can be refocused on a stronger resource description about the genome assembly and differential expression dataset.

Powered by Editorial Manager® and ProduXion Manager® from Aries Systems Corporation

Reply: We sincerely appreciate the authoritative and constructive suggestions of Bernardo J. Clavijo and Luis Yanes. We have revised our work according to the comments.

Major comments
Experimental design, data description and availability:

1)Reads submitted to public archives should be raw, except only for demultiplexing. This is a prerequisite for reproducibility and traceability. All data that has been submitted trimmed or otherwise pre-processed must be resubmitted.
Reply: Thank you for pointing this out. We have already uploaded raw reads to NCBI, and here are the SRA accession numbers:
EXPERIMENT: PG10000 (SRX2955138),
EXPERIMENT: PG250 (SRX2955139),
EXPERIMENT: PG500 (SRX2955140),
EXPERIMENT: PG2000 (SRX2955141), and
EXPERIMENT: PG5000 (SRX2955142).

2)There is no description of the protocols used to generate the paired end data and long mate paired data for genome assembly. Specially with the LMP data, the reads seem untrimmed, but they do contain substantial amounts of short-insert contamination which may be better accounted for if the protocol is properly described.
Reply: We have added the information, including the library construction, on how to generate paired end data and long mate paired data in R1. We apologize for the unclear description in V1 about processing the LMP data. We used Skewer (Jiang et al., 2014) for adapter trimming and low-quality base filtering with these parameters. Transposase adapter sequences were used for adapter searching and trimming, and the maximum mismatch rate was set to 10%; trimming reads were from 3′ end until Q>20. After trimming, reads (2–10 kb) with read length <18 bp or average quality <30 were filtered out.

3)There is no justification for the choice of line IR826 over any other line, and no mention whether the independent samples for differential expression are from a single plant or different plants, and if those are from the IR826 line.
Reply: We had two reasons for selecting line IR826. First, the heterozygosity (1‰–5‰) of this line was relative low, which favored genome assembly. Second, we accumulated some horticultural data of IR826 in a previous study. As IR826 was the strain we used for field planting and was still in its test phase, the samples we used for tissue transcription profile were collected from different plants (some of which were not line IR826).

4)In general, including datasets from different studies for differential expression analysis is not automatically reliable or even comparable. The use of external datasets for every tissue but root needs to be properly supported by analysis that shows statistical validity of equivalent background conditions, plus detailed description of similarities between experimental conditions. Also, the replicate structure of the samples should be the same. While this may be completely true, it is not discussed in the manuscript. If these criteria are not met, there should be explicit description of why this analysis is still considered valuable and its limitations.
Reply: We agree with the reviewer’s suggestion. Analyzing new and previous datasets together was faulty. Thus, the transcription analysis of various organs was reformed with new datasets from a single study (Wang et al., 2015). The transcription profile of three different tissues in root was further analyzed for the expression patterns of different HMGR types.

Genome Assembly

5)There is no justification for the choice of assembler and no discussion about parameters (why that particular K, etc). Again, detailed description of the parametrisation for each tool is needed, with some justification when appropriate.
Reply: Thank you for this suggestion. We tested Kmer size using KmerGenie (Chikhi et al., 2014), and it recommended using an 83-mer to assemble. Then, we tried to assemble the Ginseng genome using SOAPdenovo2 and AbySS 1.9, but it ran out of memory after using AbySS 1.9. Consequently, we used 63-, 73-, 83-, and 93-mer to

assemble SOAPdenovo2 and obtained the longest N50 when using 83-mer. This information has been added to manuscript R1 (lines 20–21, page 20; lines 1–2, page 21).

6)I analysed the assembly with a KAT spectra-CN plot (using k=31, only the short-insert libraries), with the following result:
This leads to a number of comments:

7.1)The kmer coverage at k=31 is around 20x using both PE libraries, which is lower that would be recommended to construct contigs (around 35x to 100x is probably best practice nowadays). I expect the coverage at the K used for assembly to be even lower.
Reply: Thank you for this technical suggestion. When we started this project, we followed previous genome sequencing projects and decided to sequence 30X depth for every shotgun library. However, after read trimming, the total depth was a bit lower than 30X. We also have noticed some new standards for genome assembly (Clavijo et al., 2017) and have decided to follow these standards in future work.

7.2)There is a loss of single-copy content (represented by the black distribution's peak at x=20) and generation of some content in the assembly not present on the reads (x=0). While this can be explained because of the gap closing and some N inclusion in the assembly, it merits some discussion and validation.
Reply: Thank you for pointing this out. To increase the efficiency of gene prediction, we have filtered fragmental scaffolds <1000 bp long. We performed KAT analysis on scaffolds before and after filtering with the 500 bp library. No significant content loss on unfiltered scaffolds ensued. Thus, we believe that the loss of single-copy content was due to length filtering.

7.3)There is duplication on the main single-copy distribution at x=20. This could explain some of the content loss (again, due to gap closing? analyses before and after gap closing would make this clear). At the same time, SOAPdenovo2's scaffolding introduces a single N between contigs when a negative distance between them is estimated by the scaffolding algorithm. This produces both unnecessary duplication of sequence and some misassembles. There are more than 350K instances of single Ns on the assembly and soap does not output Ns on the contigs, so this should be examined.
Reply: Thank you for pointing this out. We have performed KAT analysis at 7.2, we do not believe that a single Ns led to content loss.

8)The scaffolding method is slightly unorthodox, consisting of contig assembly and scaffolding with SOAPdenovo (I assume SOAPdenovo2, but it is not properly cited and no version is mentioned), then gap closing, then a further round of scaffolding with SSPACE. In general there are better and more modern methods to do this same work. Running an ABySS assembly end-to-end and comparing the results may be a good starting point.
Reply: Thank you for the suggestion. Yes, we used SOAPdenovo2, and the reference (Luo et al., 2012) has been added to R1. We also used ABySS 1.9 but had a large computer node with 1 TB memory only, and the ABySS assembly failed because of insufficient memory.

8.1)Re-scaffolding with the same datasets should not be needed, so I would suggest the authors either just use SOAP's contigs into SSPACE, or do not perform a second round of scaffolding. Performing gap closure before any scaffolding is also not recommended, so in any case this should be the last step in the assembly. As this assembly procedure does not follow best practices, I would have also expected to see a detailed description of the results at every step (i.e. contiguity, N content, etc). Also, the detailed configuration files and commands executed to produce the assemblies should be provided on the supplementary material and referred to in the main text (this is valid for all other analyses).
Reply: Thank you for the suggestion. We have unclearly described our assembly process and have accordingly made corrections. First, we used SOAPdenovo2 to assemble contigs and scaffolds with 250 and 500 bp short-insert libraries and 2000 bp long-mate-pair library. We then used SSPACE for scaffolding with 2K bp, 5K bp, and

10K bp long-mate-pair libraries. Afterwards, we used Gapcloser for gap closing using short libraries and obtain the final scaffolds. We have validated genome assemblies with LMP data, recalculated the mate-pair distance, and speculated that no significant overscaffolding occurred. We have added SSPACE configuration files into the Supplementary Text.

8.2)Any scaffolding such as this needs some validation, at the very least by analysing synteny with related species, and or by producing wet-lab validation of some junctions.
Reply: Yes, thank you for the suggestion. We have validated the junction through alignment with published P. ginseng BAC sequences. We observed good collinearity between the draft genome and BAC sequences as shown in the following figure.

8.3)We ran library fragment size analysis in the LMP libraries by mapping them to the assembly and found significant short-insert (pair-end orientation) contamination. Fragment-size distributions also changed if the assembly was split at N runs. Peaks at exactly the "nominal" distances, specifically for the 10 Kbp library that was actually 7.5 Kbp, disappeared. This finding indicated some effect of overscaffolding. Performing this kind of analyses at every step of the assembly pipeline helped evaluate where biases were introduced.

8.4)The 10Kbp library is in fact 7.5Kbp so all references to that size should be updated. If 10Kbp has been used as size in software that does not correct for it automatically it may need to be re-run.
Reply: Thank you for pointing this out. We have run a validation test on size distribution on contigs and scaffolds. We mapped each LMP library read to contigs and scaffolds. We obtained a similar size distribution of each library between contigs and scaffolds. We speculated that small-insert-size fragments were easier to cyclize when constructing an LMP library. Similar size-distribution results showed no significant overscaffolding in scaffolds.

9)Transcript assembly mappability is shown as a metric of assembly reliability, but it is unclear that any de novo transcript assembly was performed. If that is the case, the de novo transcript assembly must be properly described and discussed.
Reply: We conducted RNA-seq assembly by using Trinity software with default parameters through one RNA-seq library and generated 75 878 transcripts (N50 length, 1446 bp; average sequence length, 926 bp), which were used for further gene annotation as RNA evidence. We have added these information to the manuscript.

10)The mapping of the 500bp library to assess non-bias is ad-hoc and I am not aware of methods that would support this (it also introduces mappability bias, etc, etc). For completeness I would prefer either metrics based on how well the original reads map back to the assembly or simply kmer spectra completeness (Disclaimer: I am the senior author of the KAT publication).
Reply: We have mapped 250 and 500 bp libraries to the draft genome using BWA mem with default parameters, which had 99.77% and 99.95% reads mapped. We also performed KAT analysis, and the result was 7.2. We found no significant content loss for unfiltered scaffolds.

RNA-seq and differential expression

11)The whole method of the transcript assembly, mapping support and numbers does not add up easily. A diagram of the annotation pipeline would clarify this, with numbers of transcripts, support for them, etc. This is a KEY point if this manuscript wants to present a resource for the community.
Reply: Thank you for pointing out this deficiency. Transcripts were de novo assembled with Trinity. Specifically, raw reads generated by RNA-seq of different parts of ginseng root were trimmed and quality controlled by skewers with the following parameters: 10% of the maximum mismatch rate, trimming reads from the 3′ end until Q>20, and trimmed reads with read length <100 bp or average quality <30 were filtered out. Thereafter, Trinity software with default parameters were used for de novo assembly. A total of 75 878 transcripts (N50 length, 1446bp; average sequence length, 926 bp)

Powered by Editorial Manager® and ProduXion Manager® from Aries Systems Corporation

were assembled and used for further gene annotation as RNA evidence. The clean RNA-seq reads were aligned to the draft genome in the orientation mode through TopHat, and gene expression levels were calculated using FPKM value.

12)As mentioned in (4) the usage of dataset from previous studies needs more justification and support.
Reply: As we stated in in (4), analyzing new and previous datasets together was defective. Thus, the transcription analysis of various organs was reformed with new datasets from a single study (Wang et al., 2015). The transcription profile of three different tissues in root was analyzed in terms of the expression patterns of various HMGR types.

13)Figure 3e is unclear and probably also belongs in the supplementary.
Reply: Thank you for the suggestion. We have moved this figure to the supplementary.

14)Figure 4a shows "a possible pathway" but gives little extra detail or justification and it is not clear from the main text neither. I think this should be expanded and properly described in the main text.
Reply: Thank you for the suggestion. Fig. 4 shows that ginsenosides were mainly biosynthesized through the themevalonic acid (MVA) pathway utilizing the precursor IPP in the cytosol. This pathway is an ancestral metabolic route in all organisms, and eukaryotes have conserved enzymes from MVA to IPP synthesis. Given that the end product is IPP, this pathway is also accepted as the upstream pathway of triterpenoid saponins, most of which have been studied well. Thus, in the main text, we showed only our new discovery, i.e., that homologous enzymes were found in P. ginseng by BLAST and motif searching. If more detailed information is needed, Reference 52 (Kim et al., 2015) would be helpful.

15)Figure 4b shows an extremely different expression levels between root and all the other tissues. While this is expected and could be supportive of other claims in the manuscript, this also shows the division of new vs. pre-existent datasets. I think differential expression between the new datasets on this manuscript should become the main focus of this figure and the relevant section. If RNA-seq data for the other tissues can be regenerated and/or shown to be perfectly comparable for this purpose, then I would suggest using differential expression patterns to test the robustness of the proposed pathway membership among the proposed genes, and even maybe trying to find "missing members" of the pathway.
Reply: Thank you for the suggestion. As explained in Q4, we replotted the heatmaps using previous datasets and new datasets independently.

16)Figure 5b uses bars and SD to show 3-point datasets. This hides the real data and leads to a feeling of over-confidence in what is effectively a small-n case. Replace with a plot showing every point. The mean can be indicated within this plots with a line, but SD is mostly meaningless with N=3.
Reply: We appreciate the reviewer's advice. Showing small sample size data using a plot was appropriate. Thus, we have redrawn Fig. 5c, replacing the bars and SD with plots showing every point.

17)I can't finish to understand Figure 7, and I am conscious about the rna-seq dataset origin is playing a role here too.
Reply: We regret the unclear description in Fig. 7, especially Fig. 7b. We have added some information to Fig. 7 (V1)/Fig. 6 (R1) to make it clearer. The upper part of Fig. 6b (R1) is a sketch of a UGT73 cluster; the line shows the cluster length and the arrows represent gene length and direction. The bottom part is the expression level of these genes, and we wanted to show that although these genes were in the same gene cluster and belonged to the same gene family, their expression patterns differed. Further study of these genes may benefit the biosynthesis analysis of ginsenosides. Thank you!

Pathway analysis

18)The first paragraph of this section is difficult to read, rewrite for clarity, please.
Reply: The MVA pathway is a conserved pathway for sterols or terpenoids synthesis. We have shortened the description of this pathway, which may have decreased the

Powered by Editorial Manager® and ProduXion Manager® from Aries Systems Corporation

clarity of this paragraph. Accordingly, we have added a couple of references (References 12, 14, and 17) to this part and to increase understanding.

19)Copy number assessment of genes based on a WGS fragmented assembly as this one should at the very least be validated by using kmer coverage and/or read mapping depth. Moreover since there is a mention to putting together some of the genes manually because they were fragmented on the original assembly.
Reply: We have extracted the alignment information (.bam file) of the scaffold that contained the UGT gene cluster from full alignment (mapping 500 bp library reads to draft genome using BWA mem). We then calculated the mapping depth of this scaffold. Except for the gap, repeat region, or high-GC-content area, the mapping depth was around 20X.

Microbial resistance

20)I am not very sure how this section relates to the rest of the manuscript.
Reply: Thank you for the suggestion. Although it is important for ginseng cultivation, this section was still slightly correlated with other parts of the manuscript. Considering the two reviewers' advice, the microbial-resistance section has been removed in R1. We are truly grateful for the reviewer's comments and suggestions.

21)The seven-time-points experiment is a whole analysis on its own, same need for experimental design explanation and rationale as in previous sections apply.
Reply: We apologize for the confusion. These data were from a previously published paper (Gao et al., 2016) and contained limited explanation and rationale in V1.

22)The last sentence is too strong on its claim for the evidence presented and seems to have no experimental validation.
Reply: We apologize for including the statement without adequate evidence. This section has been accordingly deleted in R1.

Discussion

23)The claim for LTR content being significantly higher than previously thought is a huge one, and should be supported better starting by a proper statement of the methods used in both studies. De novo methods for LTR content estimation exist that can give a figure without the bias of the assembly. If this claim is well supported it should feature more prominently, as it will probably change the understanding of the genome's history and evolution. Also, further dating of LTRs and extra analyses could help elucidate why the results are so different to previous reports.
Reply: Thank you for the professional suggestion. We think it is because of the major reason that we use the whole genome analysis rather than the BAC sequences. However, this comparison is a little unfairly as whole genome possess more information naturally. We are doing more analysis about ginseng repeats. We hope we can get more interesting information from our data.

Minor comments

1)Figure 1a should be a table, Figures 1b and 1c needs either more explanation over new findings or important confirmation or may just be moved to supplementary. I personally find it impossible to extract any information from figures like 1c.
Reply: Considering the limit in number of figures/tables and that Fig. 1a seems to be insufficiently vivid as an individual table, this figure was thus integrated into Fig. 1. We also believed that rebuilding the phylogentic tree can show the possible speciation history of different species and give feedback information for genome-assembly quality. Given that phylogenetic and Venn diagram analyses are conventional methods (Guan et al., 2016; Wei et al., 2016), we decided to retain keep these information in the original manuscript.

2)The abstract should be adjusted to get rid of unnecessary nitty-gritty details, and provide a more focused description of what the manuscript is aiming for (assembly+rnaseq or biosynthesis analysis, but tighter and more conclusive). Conclusions should not be along the lines of "this will help further work" but at the very

Powered by Editorial Manager® and ProduXion Manager® from Aries Systems Corporation

least along the lines of "this supports this, this, and this types of analyses/developments".
Reply: Thank you for your suggestion. We have rewritten the Abstract and deleted some information.
Background: Ginseng, which contains ginsenosides characterized as bioactive compounds, has been regarded as an important traditional medicine for several millennia. However, the genetic background of ginseng remains poorly understood partly because of the plant's large and complex genome composition.
Results: We report the entire genome sequence of Panax ginseng using next-generation sequencing. The 3.5 Gb nucleotide sequence contained more than 60% repeats and encoded 42 006 genes. Twenty-two transcriptome datasets and mass spectrometry images of ginseng roots were adopted to precisely quantify the functional genes. Thirty-one genes were identified to be involved in the mevalonic acid pathway. Eight of these genes were annotated as 3-hydroxy-3-methylglutaryl-CoA reductases, which displayed diverse structures and expression characteristics. A total of 225 UDP-glycosyltransferase (UGTs) were identified, and these UGTs accounted for one of the largest gene families of ginseng. Tandem repeats contributed to the duplication and divergence of UGTs. Molecular modeling of UGTs in 71, 74, and 94 families revealed a regiospecific conserved motif located at the N-terminus. Molecular docking predicted that this motif captured ginsenoside precursors.
Conclusions: The panorama of ginseng genome is a valuable resource for understanding and improving the breeding, cultivation, and synthesis biology of this key herb.

3)The organisation of the text could be much clearer (i.e. have all the data about experimental design and sequencing for genome assembly in one place in the methods, the same for differential expression, etc).
Reply: Thank you for this suggestion, some adjustments have been made to increase text clarity in R1.

4)There is no justification on the plant organisms selected for comparison.
Reply: Thank you for pointing this out. Giseng is a traditional herb and has a long cultivation history in East Asia. We selected the plant organisms based on the physiological characteristics of P. ginseng during its cultivation. We also referred to some references regarding organism selection. We selected tissues based on the mass-spectrometry imaging results of ginseng root. Our future studies will include more detailed comparison works.

5)Figure 3b and 3c are plotted in such a way that some points are "hidden" behind other points.
Reply: Thank you for pointing this out. We have modified the Fig. 3b and 3c in R1 to show all sample points.

6)Figure 5 is the first point where "4-year-old" is mentioned. This belongs in methods and needs some explanation.
Reply: Thank you for pointing out this issue. We described the sample information in the method section in R1.

7)Figures 6b and 6c provide no information. Should be removed and/or a section (possibly in the supplementary) describing their results and importance should be explicitly written.
Reply: Thank you for the suggestion. We have deleted them in R1 considering that the microbial-resistance section has been removed.

8)The sections for phylogenetic analysis and gene family identification are vague and/or add little information for the rest of the manuscript's analyses. They can either be moved to supplementary or their links to the rest of the manuscript stressed and the analyses strengthened.
Reply: Thank you for the kind suggestion. We have highlighted the reason why we chose to keep this part in minor comment 1. Furthermore, we have strengthened our findings by highlighting the importance of phylogenetic analysis and introducing some necessary citations in R1.

9)In general the discussion has some claims that do not seem completely supported

	from the current analyses, but I feel a plant biologist will be better suited to judge that.<br>Reply: Thank you for your comment. These suggestions are indeed very valuable to our work.<br><br>Best regards,<br>Bernardo J. Clavijo<br>NOTE: review produced with extensive help from Luis Yanes from my research team.
**Additional Information:**	
**Question**	**Response**
Are you submitting this manuscript to a special series or article collection?	No
**Experimental design and statistics**<br><br>Full details of the experimental design and statistical methods used should be given in the Methods section, as detailed in our Minimum Standards Reporting Checklist. Information essential to interpreting the data presented should be made available in the figure legends.<br><br>Have you included all the information requested in your manuscript?	Yes
**Resources**<br><br>A description of all resources used, including antibodies, cell lines, animals and software tools, with enough information to allow them to be uniquely identified, should be included in the Methods section. Authors are strongly encouraged to cite Research Resource Identifiers (RRIDs) for antibodies, model organisms and tools, where possible.<br><br>Have you included the information requested as detailed in our Minimum Standards Reporting Checklist?	Yes
**Availability of data and materials**<br><br>All datasets and code on which the conclusions of the paper rely must be either included in your submission or deposited in publicly available repositories (where available and ethically appropriate), referencing such data using a unique identifier in the references and in the "Availability of Data and Materials" section of your manuscript.	Yes

Have you have met the above requirement as detailed in our Minimum Standards Reporting Checklist?	

Powered by Editorial Manager® and ProduXion Manager® from Aries Systems Corporation

Manuscript Click here to download Manuscript Manuscript R1.docx

Ginseng genome examination for ginsenoside biosynthesis

Xu Jiang[1, *], Chu Yang[1, *], Xiao Shuiming[1, *], Liao Baosheng[1, *], Yin Qinggang[1], Bai Rui[1], Su He[1, 2], Dong Linlin[1], Li Xiwen[1], Qian Jun[1], Zhang Jingjing[1], Zhang Yujun[1], Zhang Xiaoyan[1], Wu Mingli[1], Zhang Jie[1], Li Guozheng[3], Zhang Lei[4], Chang Zhenzhan[5], Zhang Yuebin[6], Jia Zhengwei[7], Liu Zhixiang[1], Daniel Afreh[8], Ruth Nahurira[8], Zhang Lianjuan[1], Cheng Ruiyang[1], Zhu Yingjie[1], Zhu Guangwei[1], Rao Wei[7], Zhou Chao[7], Qiao Lirui[7], Huang Zhihai[2], Cheng Yung-Chi[9,$], Chen Shilin[1,$]

[1]*Institute of Chinese Materia Medica, China Academy of Chinese Medical Sciences, Beijing 100700, China*

[2]*Guangdong Provincial Hospital of Chinese Medicine, Guangzhou 510006, China*

[3]*National Data Center of Traditional Chinese Medicine, China Academy of Chinese Medical Sciences, Beijing 100700, China*

[4]*Institute of Basic Research in Clinical Medicine, China Academy of Chinese Medical Sciences, Beijing 100700, China*

[5]*Department of Biophysics, School of Basic Medical Sciences, Peking University Health Scien ce Center, Beijing 100191, China*

[6]*State Key Laboratory of Molecular Reaction Dynamics, Dalian Institute of Chemical Physics, Chinese Academy of Sciences, Dalian 116023, China*

[7]*Waters Corporation Shanghai Science & Technology Co Ltd, Shanghai 201206, China*

[8]*Institute of Crop Science, Chinese Academy of Agricultural Sciences/Key Laboratory of Crop Physiology and Ecology, Ministry of Agriculture, Beijing 100081, China*

[9]*Department of Pharmacology, School of Medicine, Yale University, New Haven, 06510, CT, USA*

* These four authors contributed equally to this work.

[$] Correspondence: Chen Shilin[a], Cheng Yungchi[b]

[a]E-mail: slchen@icmm.ac.cn

[b]E-mail: yccheng@yale.edu

Abstract

Background: Ginseng, which contains ginsenosides characterized as bioactive compounds, has been regarded as an important traditional medicine for several millennia. Howerver, the genetic background of ginseng remains poorly understood partly because of the plant's large and complex genome composition.

Results: We report the entire genome sequence of *Panax ginseng* using next-generation sequencing. The 3.5 Gb nucleotide sequence contained more than 60% repeats and encoded 42,006 predicted genes. Twenty-two transcriptome datasets and mass spectrometry images of ginseng roots were adopted to precisely quantify the functional genes. Thirty-one genes were identified to be involved in the mevalonic acid pathway. Eight of these genes were annotated as 3-hydroxy-3-methylglutaryl-CoA reductases, which displayed diverse structures and expression characteristics. A total of 225 UDP-glycosyltransferase (UGTs) were identified, and these UGTs accounted for one of the largest gene families of ginseng. Tandem repeats contributed to the duplication and divergence of UGTs. Molecular modeling of UGTs in the 71, 74, and 94 families revealed a regiospecific conserved motif located at the N-terminus. Molecular docking predicted that this motif captured ginsenoside precursors.

Conclusion: The panorama of ginseng genome represents a valuable resource for understaning and improving the breeding, cultivation, and synthesis biology of this key herb.

Key words: *Panax ginseng*; ginsenosides; genome; mass spectrometry imaging

Background

Panax ginseng C. A. Mey, a deciduous perennial plant belonging to the Araliaceae family, has been clinically used as a precious herbal medicine for several millennia in East Asia [1]. The name ginseng was translated from the pronunciation of the Chinese words “Ren shen” [2]. Modern pharmacological research confirmed that ginsenosides, the major bioactive compound of *P. ginseng*, exhibit multiple therapeutic activities. These activities include antitumor, antihypertensive, antivirus, and immune modulatory activities [3]. Therefore, *P. ginseng* is used as a general tonic or adatogen to promote longevity, particularly in China, Korea, and Japan [4].

Different ginseng tissues, such as the root and rhizome used in clinical practice, show significant differences in quality evaluation, commercial application, and clinical efficacy because of variations in ginsenosides [5]. Ginsenosides are frequently allocated and accumulated in specific tissues through transport systems for storage or defense. Chemical analysis, immunological staining, and microscopic imaging have all demonstrated that the ginseng cortex and periderm contain higher amounts of protopanaxadiol (PPD)-type ginsenoside (Rb1, Rb2, or Rc) and protopanaxatriol (PPT)-type ginsenoside (Rf) than those of the root medulla [6-8]. Histochemical staining also confirmed that ginsenosides are mainly located in the oil canals of the periderm and outer cortex regions of the root but not in the xylem nor pith [9, 10]. Considering their potential physiological role [11], the ginsenoside enrichment in the periderm is consistent with the plant’s biological function as phytoanticipin, which protects plants against pathogens.

Although the pharmacological importance of ginsenosides has been well established, their biosynthetic enzymes and regulatory mode remain unknown [12-17]. Ginsenosides are

biosynthesized through the cytosolic mevalonic acid (MVA) pathway, which is initiated by acetyl coenzyme A and ended with the terpene precursor isopentenyl diphosphate (IPP). After a series condensation reactions, a linear C30 molecule, that is, squalene, is generated [18] and converted into (S)-2,3-oxidosqualene [19] through cyclization [20]. Subsequently, after multiple oxidation events (e.g., mediated by cytochrome P450-dependent monooxygenases) [21-23], various types of ginsenoside precursors, including oleanolic acid and PPD/PPT, are formed. The precursors are then further decorated through glycosylation reactions [12, 13, 17].

The glycosylation reaction, namely the transfer of a sugar moiety to a specific acceptor, is performed by glycosyltransferases (GTs), a group of multigene superfamilies. The GTs that utilize uridine diphosphate (UDP) activated sugar molecules as donors are referred to as UDP-glycosyltransferases (UGTs). The diversity of the UGTs has been demonstrated by comparing genomic and complementary DNA (cDNA) sequences. In our previous work, 129 potential UGT sequences were predicted on the basis of annotation results from the transcriptome data of *P. ginseng* roots, stems, leaves, and flowers. Some of the sequences may encode enzymes responsible for ginsenoside backbone modification [24]. However, only a limited number of UGTs that glycosylate triterpenoid aglycones have been described in plants, such as *Medicago truncatula* [25], *Saponaria vaccaria* [26], *Barbarea vulgaris* [27], *Glycine max* [28], and *P. ginseng* [29-31]. Yan *et al.* [30] reported that the UGTPg1 from *P. ginseng* glycosylates the C20-OH of PPD and its derived ginsenosides in a regiospecific manner. Two recently identified UGTs from *P. ginseng* (PgUGT74AE2 and PgUGT94Q2) catalyze the glycosylation of the C3–OH of PPD to obtain Rh2 and elongate the glucose moiety of Rh2 to generate Rg3 [31]. Wei *et al.* [32] found that UGT1 and its homologous genes from *P. ginseng* can glycosylate PPT to

produce PPT-derived ginsenosides, which contain several key amino acids that determine their activities and substrate regiospecificities.

The functional genomic analysis of ginseng significantly progressed but still requires improvement. First, the analysis of gene and transcript expression has mainly focused on ginseng organs, but the ginsenoside content and types vary among different tissues within the same organ. Hence, the screening of potential key genes responsible for synthesizing and modifying ginsenosides by association analysis of transcriptome and chemical substances is not comprehensive. Second, gene duplication often leads to functional divergence. Even paralogous genes that execute the same function are usually regulated in different modes. In ginseng, the ubiquitous duplicated genes are difficult to fully illustrate using current datasets. Therefore, the analysis of the whole genome sequence and transcriptomes by the accurate location of ginsenosides may promote the precise mining of genes associated with ginsenoside synthesis. Herein, we present the genome sequence of *P. ginseng* and comprehensively characterize the genes responsible for ginsenoside biosynthesis and modification in the plant.

Data Description

Genomic DNA was extracted from the 4-year old *P. ginseng* line IR826, a strain cultivated by the Institute of Chinese Materia Medica. Five libraries with insert sizes ranging from 250 bp to 10 kb were constructed using the commercial library prep kits (Vazyme Biotech). Paired-end sequencing were performed using the HiSeq platform (Illumina) and 391.46 Gb raw data were produced (Supplementary Table S1). The raw reads were trimmed using skewer pipeline to remove low quality or duplicated reads. After trimming, 315.93 Gb data were used for genome

assembly. The final assembly was checked using Benchmarking Universal Single-Copy Orthologs (BUSCOs). The frozen transverse sections of the ginseng main root with 20 μm thickness were prepared using a microcryotome for DESI-MS imaging. The ginsenoside distribution was evaluated on a Xevo G2-XS Tof mass spectrometer with the DESI source. The image creation was performed using high-definition imaging (HDI) software (Waters Corporation) with the following parameters: X and Y pixel size 100 μm; raster speed 400 μm/s; spray solvent 90% MeOH, 10% H_2O, 0.1 mM NH_4Cl, and 0.1 mM leucine enkephalin delivered at 1.5 μl/min; MS at negative polarity, 4.5 kV capillary voltage, 80 V cone voltage, and mass range m/z 100-1,200. Total RNA were isolated from the periderm, cortex, and stele to construct RNA-seq libraries, each for triplicates. The RNA-Seq transcriptome libraries were prepared following the TruSeq™ RNA sample preparation kit (Illumina). After quantification, the paired-end libraries were sequenced by HiSeq 4000 (Illumina) (Supplementary Table S2). Except the nine RNA-seq data generated in this study, 13 published ginseng RNA-seq data were re-used. Further details about sample collection, DNA/RNA extraction, library construction, sequencing and mass spectrometry imaging can be found in the Methods section. All genome data have been uploaded to GigaDB [33] and sequencing reads can be found at NCBI (https://www.ncbi.nlm.nih.gov/) under the project number PRJNA385956.

Analyses

Characteristics of the *P. ginseng* genome

Genomic DNA was extracted from the 4-year old *P. ginseng* line IR826, a strain cultivated by the Institute of Chinese Materia Medica. This strain contains an estimated genome size of

3.5 Gb based on the k-mer prediction and flow cytometry analysis (Supplementary Figure S1; Supplementary Table S3). Approximately 112 X coverages of the raw sequence were generated using the Illumina HiSeq platform (Supplementary Table S1). After filtering, 91 X high-quality reads were adopted for assembly (Supplementary Table S1). The results provided a 3.43 Gb draft assembly with a contig N50 of 21.98 kb and a scaffold N50 of 108.71 kb (Fig. 1a). Shotgun libraries with an insert size of 250 bp and 500 bp was mapped to the assembly, which have read mapping rate 99.77% and 99.95% respectively. The Poisson-like distribution of the sequence depth per base represents a nonbiased sequencing and assembly (Supplementary Figure S2). To confirm the accuracy, the 75,878 transcripts assembled from RNA-Seq data using Trinity [34] with default parameters were mapped back to the assembly with a mapping rate of 97.76%. Furthermore, Benchmarking Universal Single-Copy Orthologs (BUSCOs) [35] were used for quality assessment. A total of 1,323 (91.88%) CEG proteins, of which 24 BUSCOs were fragments, were determined in this assembly; 98.19% of the proteins were fully annotated, indicating the accuracy of the assembly.

More than 62% of the ginseng genome was predicted to be repeats; about 83.5% of the repeats were annotated as long terminal repeats (LTRs) (Supplementary Table S4 and S5). Ty3/Gypsy is the most abundant retro-element superfamily and accounts for 42.8% of the genome (Supplementary Table S6), which was higher than previously reported [36]. Moreover, the amount of Ty1/Copia comprised approximately 8.3% of the whole genome and exceeded previous predictions [36] (Supplementary Table S6). For the DNA transposon class, CMC was the most abundant repeat type and comprised 43 Mb of approximately 1.3% of the genome (Supplementary Table S6).

A total of 42,006 protein-coding gene models were predicted on the basis of *ab initio* and comparison methods using the MAKER pipeline. That is, 88% of these models were supported by the assembled RNA-Seq transcripts. More than 95.6% of the gene models contained homologs in the GenBank nonredundant database (E-value=1e−5). About 73.47% annotations could be assigned to Gene Ontology (GO) catalogs, and 68.39% could be assigned to Kyoto Encyclopedia of Genes and Genomes (KEGG) pathways (Supplementary Figure S3). Among these annotations, the following genes were obtained: 488 cytochrome P450 genes, including the PPD-ginsenosides synthase (PPDS) CYP716A47, PPT-ginsenosides synthase (PPTS) CYP716A53, and oleanolic acid synthase CYP716A52; 2,556 transcription factors; and 3,745 transporters (Supplementary Table S7 and S8).

Ortholog analysis of *P. ginseng* was conducted using 13 other plants (Supplementary Table S9). More than 75% of the gene models in *P. ginseng* were classified into 12,231 gene families, with 1,648 unique gene families for *P. ginseng* itself (Fig. 1b). The average gene number per gene family was 2.59, which was the highest among all 14 plants. This finding indicates the occurrence of duplication events during the evolution of *P. ginseng*. 383 single copy genes identified by ortholog analysis, we constructed a phylogenetic tree using the maximum-likelihood method. *Daucus carota* from Umbelliferae was found to be the closest relative of *P. ginseng* among all the compared species, diverging approximately 66 Myr ago (Fig. 1c), which is further supporting the relative evolutionary relationships between *Daucus carota* and *Panax ginseng* (http://www.uniprot.org/taxonomy/4054), and supporting the prevailing hypothesis of seed plants' phylogeny [37].

Metabolism and transcriptome of the ginseng root

Desorption electrospray ionization mass spectrometry (DESI-MS) imaging was used to elucidate the spatial distribution of ginsenosides within the ginseng root sections. Ginsenosides Rg1/Rf, pseudo Rc1, Ra1/Ra2, Rd/Re, Rs1/Rs2, and Ra3 were identified and summarized (Fig. 2b; Supplementary Table S10). Ginsenosides Rg1/Rf were highly concentrated within the outer bark and inner core areas of the root. Rd/Re Rs1/Rs2, Ra1/Ra2, and pseudoginsenoside Rc1 were distributed at high concentrations in the bark and at low concentrations in the center (Fig. 2c). Ginsenoside Ra3 exhibited a diffuse distribution within the cross section and a high concentration around the bark (Fig. 2c). These isomers were distinguished by DESI-tandem mass spectrometry (MS/MS). For Rf/Rg1, fragmentation of the monosaccharide group $C_6H_{10}O_5$ (162.05 Da) and disaccharide group $C_{12}H_{22}O_{11}$ (342.12 Da) produced fragments at *m/z* 637.46 and 457.15, which corresponded to different spatial distributions (Supplementary Figure S4). The characteristic MS/MS transitions were *m/z* 603.08 for Rd and *m/z* 799.52 for Re (Supplementary Figure S5). The enrichment of Rb1 around the bark was also confirmed through DESI-MS/MS (Supplementary Figure S6).

On the basis of anatomical characteristics, we categorized the ginseng main root into periderm, cortex, and stele for further quantitative analysis (Supplementary Figure S7). High-performance liquid chromatography (HPLC) results showed that the contents of ginsenosides Rg1, Re, Rf, Rg2, Rb1, Rc, Rb2, and Rd were significantly higher in the periderm ($P<0.001$) than in the cortex and stele (Fig. 3a; Supplementary Table S11). The PCA and PLS-DA plots

showed the distinct clustering among the periderm, cortex, and stele groups (Fig. 3b and c). The findings suggest the different distribution of ginsenosides.

More than 34,000 predicted genes were detected from the transcriptome data. Among these genes, 27,450 were expressed in the three sections, and 7,456 genes were not detected in any section. The samples were clustered into three distinct groups by expression profile. The expression pattern of genes in the cortex was closer to the stele than to the periderm (Fig. 3d). A total of 2,530, 2,688, and 711 differentially expressed genes were found between the periderm and cortex, the periderm and stele, and the cortex and stele, respectively. GO enrichment analysis showed that differential genes between the periderm and cortex, as well as the periderm and stele, were mainly associated with metabolic processes and response to stimuli (Supplementary Figure S8). The total number of genes were grouped into 64 modules through weighted gene coexpression network analysis (WGCNA). The total ginsenoside content was considered as the weighted factor, and three of the modules were positively correlated with ginsenosides. The most correlated module contained 15,762 genes, indicating the complex mechanisms involved in ginsenoside synthesis and regulation (Supplementary Figure S9).

Conserved biosynthesis pathway of ginsenosides

As triterpenoid saponins, ginsenosides are mainly biosynthesized using the precursor IPP produced through the MVA pathway, which includes conserved enzymes in eukaryotes [12, 14, 17]. In this study, 31 genes encoding 10 upstream enzymes were identified by BLAST search and motif finding (Fig. 4a). Except for acetyl-CoA C-acetyltransferase (AACT), all of these 10 enzymes displayed multiple copies and isoforms; 5 enzymes (8 in 3-hydroxy-3-methylglutaryl-

CoA reductase [HMGR], 4 each in squalene synthase [SS] and squalene epoxidase [SE], and 3 each in phosphomevalonate kinase [PMK] and 3-hydroxy-3-methylglutaryl-CoA synthase [HMGS]) had multiple copies and isoforms. One of the PMKs may be a potential pseudogene, with several termination codons dividing the coding regions. The four other enzymes (mevalonate kinase [MVK], mevalonate diphosphate decarboxylase [MVD], isopentenyl-diphosphate delta-isomerase [IDI], and farnesyl diphosphate synthase [FPS]) possessed two copies each. Such common occurrence of the multicopy phenomenon in ginseng MVA enzymes may be associated with the diverse regulatory control of triterpenoid or steroid biosynthesis in the plant. After the formation of two 3-oxidosqualenes, different ginsenoside precursors are cyclized and hydroxylated by various enzymes. In this assembly, five beta-amyrin synthases (beta-ASs), three oleanolic acid synthases (OASs), three dammarendiol synthases (DDSs), and three PPDSs, three PPTSs were identified. In addition, 100 terpenoid synthases were annotated, including one lanosterol synthase (LAS) and one cycloarstenol synthase (CAS) for ginseng sterol precursor cyclization.

The transcriptome of nine released RNA-Seq data (arm root, rhizome, stem, leaf blade, leaflet pedicel, leaflet peduncle, fruit pedicel, seed and fruit flesh) [38] were used for the expression analysis of ginsenoside biosynthesis upstream genes. Two organs from subterranean part were grouped into one clan. By contrast, the aerial parts, were grouped into another clan (Fig. 4b). The samples, fruit flesh and seed, were relatively privileged possibly because of their singleness as reproductive organs. Some genes were coexpressed in different organs. For example, PG07131 (HMGR), PG03840 (HMGR), PG11918 (SE), and PG28400 (PPTS) were particularly expressed in the fruit flesh sample but not in the other tissues. Meanwhile, PG19915

(OAS), PG16025 (SE), PG00849 (beta-AS), and PG37498 (HMGR) were coexpressed in the seed. In leaf blade, PG02251 (HMGR), PG38245 (HMGR), PG13769 (DDS), PG09257 (DDS), and PG03815 (CAS) were higher expressed. On the basis of hierarchical cluster analysis, the upstream genes were clustered into different groups with specific expression pattern. This pattern may be related to the organ-specific chemical distribution of ginseng (Fig. 4b).

Sequence analysis of the *P. ginseng* HMGR (PgHMGR) family

HMGRs catalyze the conversion of HMG-COA into MVA, which has been considered as the first committed step of ginsenoside synthesis. Eight HMGR-encoding genes were annotated. The full length of these genes were achieved by manual curation. Four of these genes showed high similarity to previously reported PgHMGR1 (with average similarity of 94.25%), and the other four genes showed similarity to PgHMGR2 (with average similarity of 93.26%) (Supplementary Table S12). Given the primary structure of putative peptide sequences, the eight PgHMGRs were further grouped into four subfamilies, namely, PgHMGR1.1 (PG16235, PG37498), PgHMGR1.2 (PG00233, PG15732), PgHMGR2.1 (PG03840, PG07131), and PgHMGR2.2 (PG38245, PG02251) (Fig. 5a). The PgHMGR1 family attained relatively shorter lengths, with 573 amino acids (aa) for HMGR1.1 and 565 aa for HMGR1.2. By contrast, the PgHMGR2 family revealed relatively long lengths, with 594 aa for HMGR2.1 and 589 aa for HMGR2.2 (Fig. 5b). Most of the PgHMGR-encoding genes (except PG15732) contain four exons and share the same exon phase pattern with the combination “0-2-1-0”. The PgHMGR2 family was 63 bp longer than PgHMGR1 in the first exon region, but both families were roughly the same in size as the three other exons. The introns among the PgHMGR-coding genes

fluctuated more than did the exons. Among the introns, the second intron varied the most, with a standard variation of 187 bp (Fig. 5d).

The deduced PgHMGRs were highly conserved at the C-terminal for MVA catalysis but were divergent at the N-terminal for membrane anchoring. Similar to most plants, all of the PgHMGRs contained a membrane anchor domain with a typical helix–loop–helix structure, a linker region for connection, two HMG-COA-binding motifs (MP(I/V)GY(I/V)QIP and TTEGCLVA), and two NADPH-binding motifs (DAMGMNM and GTVGGGT) (Fig. 5b). Therefore, the functional sites of all HMGRs were composed of similar residues, especially in the core region containing catalytic domains. Differences mainly located at the N-terminal were responsible for HMGR subcellular localization (Supplementary Table S13). All the deduced proteins, except HMGR1.2 (PG00233 and PG15732), attained a triple consecutive arginine region. This characteristic was implicated for endoplasmic reticulum retention. The expression patterns of different HMGR types differed among various organs (Fig. 4b and 5c). From the calculation of fragments per kilobase of exon model per million mapped reads (FPKM), the HMGR1 family expressed more stably with average FPKM CV of 81.97% and average extreme deviation of 1,107.81. Meanwhile, HMGR2 attained an average CV of 162.57% and average extreme deviation of 5,652.10, which was about 5 times higher than that of the HMGR1s (Supplementary Table S14). The HMGR2s were distinct among the tissues. Similar to PG07131 and PG03840, HMGR2.1 was highly expressed in fruit flesh and seedbut rarely in all other tissues (Supplementary Table S14). The excessive deviation of PG07131 reached 13,976.63, showing extreme tissue specificity (Supplementary Table S14). The two members of the HMGR2.2 family were prevalently expressed in leaf blades and highly expressed in the roots

(Supplementary Table S14). Analysis of the expression patterns of HMGRs indicated that they may perform different task assignments in ginseng development.

UGTs of *P. ginseng*

UGTs are in charge of transferring glycosyl moieties to acceptor molecules, including ginsenosides. The ginseng genome encodes a large, diverse set of UGTs. A total of 225 UGTs were identified, accounting for one of the largest gene families in ginseng. The length of these putative UGTs ranged from 74 aa to 575 aa. Moreover, the predicted isoelectric point ranged from 4.45 to 9.54. The identified UGTs were newly classified according to the standardization of the UGT Nomenclature Committee. As a result, all the UGTs were assigned to 24 subfamilies (Fig. 6a). UGT73 was the most abundant group (with 30 members), followed by UGT74 and UGT94 (with 25 and 24 members, respectively). Compared with *D. carota*, UGT74 and UGT71 notably expanded, whereas UGT93 largely shrank. Seventy-eight UGTs were found to be physically clustered into 30 groups, and the largest group contained five members. The PgUGTs were clustered similar to tandem repeats and generally belonged to the same subfamily. Similar to the largest cluster, all the members originated from an ancestral UGT73, with similarity ranging from 48% to 92%. The high similarity indicated that these genes may have evolved from recent genome duplications or newly unequal recombination events.

The expression module of UGTs also showed high tissue specificity. Similar to the mentioned gene cluster, the expression patterns of these UGTs considerably differed, although all of them originated from the same gene family (UGT73) (Fig. 6b). PG22765-1 was the most highly expressed member with an average FKPM of 3,089 and was the only highly expressed gene in

the root, followed by PG22765-2 with an average FKPM of 1,957. Meanwhile, PG22765-5 was the most fluctuant gene, with a CV of 186.72. This UGT was rarely expressed in the organ root, stem, or leaf but highly expressed in the fruit. Hence, even UGTs that belong to the same family or located closely showed a distinctly regulated gene expression.

For functional analysis, 18 UGTs from families 71, 74, or 94 were chosen for molecular modeling and docking. The models of PPD and PPT were selected as docking substrates, and UGT-Glc was selected as sugar donor. The N-terminal I/V-G/S-H motif, the C-terminal W-N-S-X-L-E motif, and the C-terminal Y-G/A-E-Q motif of UGT71 family; the N-terminal motif Q-G-H-X-N/S and the C-terminal H-C/S-G-W-N-S-T-X-E motif of UGT74 family; and the N-terminal H/Q/Y-G-H motif and the C-terminal D-Q motif of UGT94 family were predicted to bind specifically to the sugar acceptors (Supplementary Figure S10). The results showed that the key residues in the N-terminal may have been subject to selection pressure during evolution for a particular substrate binding.

Discussion

Herbgenomics has been proposed as a global platform for securing the synthesis pathways of bioactive compounds [39-42]. This manuscript presented the genome of *P. ginseng*, which is the representative of herbs. The assembly confirmed the previous per-haploid-genome estimation of *P. ginseng* at approximately 3.5 Gb. Second only to *Ginkgo biloba*, ginseng harbors the largest genome among the sequenced medicinal plants [43]. Detailed structural analysis revealed that more than 62% of the genome consisted of repeats. This value is the highest among those of all sequenced angiosperms, similar to orchid (61%) and higher than

sorghum (58%), grape (49%), and rice (35%) [44-47]. LTR is a key factor in genome expansion. In *P. ginseng*, LTR accounted for 52% of the genome, which is 1.5-fold higher than a previous estimation using bacterial artificial chromosomes (BACs) [36]. This result further emphasized the importance of whole-genome sequencing in the analysis of species evolution.

Compared with regular chromatography methods, DESI-MS enables the exploration of secondary metabolite distribution in tissues and even in cells. The resolution of DESI-MS typically reaches 100 μm or higher [48]. The spatial distribution images can show the continuous changes of ginsenosides in the ginseng root cross sections. These findings are expected to contribute to the screening of the physiological role, transport process, and accumulation of ginsenosides during ginseng growth and development, as well as in defense reactions, as responses to environmental challenges. DESI-MS can directly analyze isomeric compounds *in situ* [49]. Imaging ginsenosides by mass spectrometry confirmed the spatial maldistribution of ginsenosides. The data hence provided evidence for further gene expression analysis. Meanwhile, some ginsenosides accumulated in the root center, suggesting multiple sources of ginsenoside supply (Fig. 2). Schramek *et al.* found by ^{13}C-label tracing that the precursor units of ginsenosides are transferred from the leaves to the roots [50]. However, the mechanism underlying this long-distance transport and allocation remains unknown. Kim *et al.* speculated that ATP-binding cassette transporters or multidrug and toxic compound extrusion transporters may be involved in the transport process [51]. In the present research, more than 4000 transporters, including 331 ABC superfamily transporters and 71 MATE transporters, were identified. The obtained sequence information would facilitate future biochemical studies on ginsenoside transport.

The IPP for ginsenoside biosynthesis is generally produced via the MVA route. However, inhibition assays indicated that the methylerythritol phosphate (MEP) pathway compensated for IPP production when MVA was blocked. The MEP pathway is initiated by condensation between D-glyceraldehyde-3-phosphate and pyruvate by 1-deoxy-D-xylulose 5-phosphate synthase (DXP synthase, DXS). The pathway then terminates with the conversion of 4-hydroxy-3-methyl-butenyl 1-diphosphate (HMBPP) into IPP or dimethylallyl diphosphate (DMAPP) by isoprenoid synthase-containing protein H (IspH). In ginseng, the putative proteins involved in the MEP pathway were found to include 9 DXSs, 4 DXRs (DXP reductoisomerase), 2 IspDs, 4 IspEs, 5 IspFs, 4 IspGs, and 5 IspHs (Supplementary Figure S11). Similar to that in the MVA pathway, the members of the MEP route share a common multicopy phenomenon. Gene duplication was usually followed by functional divergence and metabolite diversity. As a result, certain ginsenosides or genes accumulated in different organs or tissues. Hitherto, this correlation has been largely unappreciated. Kim *et al.* cloned three SQSs based on ginseng expression sequences tags (ESTs) and reported their expression preferences [52]. Kim *et al.* found two copies of HMGR in ginseng and speculated that PgHMGR1 plays a general role in secondary metabolite production, whereas PgHMGR2 may be related to age-dependent ginsenoside accumulation in the root [53]. In the present study, more than two up to eight PgHMGRs were encoded by ginseng genomes. Of these PgHMGRs, four belong to the HMGR1 family and four belong to HMGR2 family. Each family can be grouped into two subfamilies. The expression of PgHMGR2s was more fluctuant than that of PgHMGR1s among organs or tissues in ginseng. This result suggests that PgHMGR2 may conduct the regulation roles in terpene/phytosterol production during ginseng development. These results imply that

the presence of multiple isoforms in the MVA/MEP route may contribute to flexible production or regulation of triterpene biosynthesis.

The glycosylation of triterpenes may increase their water solubility and modify their biological activities. In ginseng, UGTs are necessary for the ginsenoside biosynthesis by transferring monosaccharides to triterpene aglycones at C-3, C-6, or C-20 for the PPD- or PPT-type ginsenosides [51]. UGTs belong to a large and diverse gene family and can recognize a wide range of natural compounds as acceptor molecules. Triterpene glucosyltransferases belong to the UGT families 71, 73, 74, and 94 [25, 30]. These families are the most abundant UGT families in ginseng. Compared with other plants, triterpene glucosyltransferases were enriched in the ginseng genome during the evolution. This enrichment can partially account for the diversification of ginsenosides. Eighteen UGTs from UGT 71, 74, and 94 were selected for molecular modeling and docking. The results indicated that these UGTs were conserved in a three-dimensional structure and displayed a general regiospecificity but not tight substrate specificity. This finding can be proven by the report of Wei et al., stating that certain UGTs can modify both PPD- and PPT-type ginsenosides *in vitro* [32]. We have cloned and have prokaryotically expressed a putative UGT gene of ginseng with only one synonymous mutation to previously reported PgUGT94Q2 [31]. Normally, the functional assay of this gene is the same as reported; this gene can catalyze the conversion of ginsenoside Rh2 into ginsenoside Rg3 and that of ginsenoside Rf2 to ginsenoside Rd (Supplementary Figure S12). Further biochemical experiments are required for other candidate tetracyclic triterpene UGTs.

This research showed the genome sequence of *P. ginseng*. The pathway for the synthesis of ginsenosides was described and examined. Multiple copies of the MVA pathway and the fully

described UGTs demonstrated the importance of the whole genome sequencing, while the knowledge of the specific expression of the isoform of MVA enzymes and the expansion of particular members of UGTs expanded the understanding of the regulation of ginsenoside biosynthesis. This research will contribute to ginseng breeding, cultivation, and synthesis biology and provides an effective solution for plant functional genomic analysis with increased throughput, precision, and sensitivity [54].

Methods

Genome sequencing and assembly

Genomic DNA was extracted from the 4-year old *P. ginseng* line IR826, a strain cultivated by the Institute of Chinese Materia Medica. Five libraries with insert sizes ranging from 250 bp to 10 kb were constructed. We performed the paired-end sequencing on the HiSeq platform (Illumina) and produced 391.46 Gb raw data (Supplementary Table S1). The genome size was estimated through the flow cytometry (BD Biosciences) analysis and K-mer distribution. The reads were filtered using a skewer [55] with the following criteria: trimming a 3′-end base to achieve quality >30 and exclusion of a short-insert library reads (250 and 500 bp) with a read length <100 bp or average quality <30; For large-insert library reads (2-10 kb), transposase adapter sequences were used for adapter searching and trimming, and max mismatch rate set to 10%, trimming reads from 3’ end till Q>20, after trimming, reads with a read length <18 bp or average quality <30 were filtered out. Finally, 315.93 Gb reads were retained for genome assembly (Supplementary Table S1) through SOAPdenovo2 [56]. K-mer size selection was performed using KmerGenie [57] with 250bp-insert library and it recommended 83-mer, then

k-mer size of 63, 73, 83, and 93 were used for assemble with default parameters, and the optimal k-mer size (k = 83) was selected based on the N50 length in each k-mer size. The reads from the small-insert libraries were used for contig construction to assemble the *P. ginseng* genome. The read pairs from the small- and large-insert libraries were then utilized to join the contigs into the scaffolds. Further scaffolding was performed using the large-insert libraries with SSPACE [58] (Configuration files in Supplementary Text). Finally, the small-insert libraries were used for gap closure of the scaffolds using GapCloser [59].

The two short-insert library reads were aligned onto the assembly through BWA mem with default parameters to evaluate the assembly quality [60]. We performed the BUSCO v2 analysis [35] with the recently released plant dataset from OrthoDB v9.1 [61] to test the completeness of the scaffolds. A total of 75,878 transcripts assembled from RNA-Seq dataset (assemble process was described at "Transcriptome sequencing and analysis" section) were mapped back to the draft genome using BLAST [62] (BLASTN, an identity cutoff value of 90%, and a coverage cutoff value of 90%).

Ginsenoside distribution and content analysis

The frozen transverse sections of the ginseng main root with 20 μm thickness were prepared using a microcryotome for DESI-MS imaging. The ginsenoside distribution was evaluated on a Xevo G2-XS Tof mass spectrometer with the DESI source (Waters Corporation). The MS images were created by spraying N_2 gas-focused solvent stream directly onto the sample to produce the MS spectra from the surface, which was then rastered across the sample at regular intervals to build a 2D image. The image creation was performed using high-definition imaging

(HDI) software (Waters Corporation) with the following parameters: X and Y pixel size 100 µm; raster speed 400 µm/s; spray solvent 90% MeOH, 10% H_2O, 0.1 mM NH_4Cl, and 0.1 mM leucine enkephalin delivered at 1.5 µl/min; MS at negative polarity, 4.5 kV capillary voltage, 80 V cone voltage, and mass range *m/z* 100-1,200. The MS images were created from raw MS files through HDI with leucine enkephalin as the lockmass (*m/z* 554.2615) for high-resolution MS. The DESI-MS/MS images were created for ginsenoside Rf/Rg1 (*m/z* 799.48, −H adduct) and ginsenoside Rd/Re (*m/z* 945.54, −H adduct), and collision energy from 10-40 (arbitrary units).

The three independent ginseng root samples were divided into three portions: periderm, cortex, and stele, which were crushed and mixed with methanol containing 0.1% methanoic acid. The mixture was frozen for 1 h and then centrifuged. The upper layer was collected, filtrated, and transferred to a sample vial to be injected and analyzed by HPLC for ginsenoside content measurement.

Transcriptome sequencing and analysis

The total RNA was extracted from the periderm, cortex, and stele using TRIzol® Reagent (Invitrogen) to construct a sequencing library. The RNA-Seq transcriptome libraries were prepared following the TruSeq™ RNA sample preparation kit (Illumina). mRNA was isolated with polyA selection by oligo(dT) beads and fragmented using a fragmentation buffer. Generally, cDNA synthesis, end repair, A-base addition, and the ligation of the Illumina-indexed adaptors were performed according to Illumina’s protocol. The libraries were selected based on the size of the cDNA target fragments of 200–300 bp, followed by PCR amplification

using Phusion DNA polymerase (New England Biolabs) for 15 PCR cycles. After quantification, the paired-end libraries were sequenced by HiSeq 4000 (Illumina).

Raw reads generated by RNA-seq of different parts of ginseng root were trimmed and quality controlled by Skewer with following parameter: adapter sequences searching and trimming with 10% max mismatch rate, trimming reads from 3’ end till Q>20, trimmed reads with read length <100 bp or average quality <30 were filtered out. Thereafter, Trinity software with default parameters were applied for de novo assembly. The total length of 75,878 assembled transcripts is 70,273,566 bp, max, min and N50 length are 12,639 bp, 201bp and 1,446 bp respectively. The clean reads were separately aligned to the *P. ginseng* genome in the orientation mode through the TopHat software (http://tophat.cbcb.umd.edu/) [63]. For comparing the gene expression pattern among the different tissues of *P. ginseng*, six other tissue RNA-Seq datasets from NCBI (accession number SRP066368) were analyzed [38]. The expression level for each transcript was calculated using the fragments per kilobase of exon per million mapped reads (FRKM) method to identify differentially expressed genes (DEGs) among the different samples. Cuffdiff (http://cufflinks.cbcb.umd.edu/) [64] was used for the differential expression analysis. The DEGs were selected using the following criteria: the logarithm of the fold change >2 and the false discovery rate (FDR) <0.05. The Gene Ontology (GO) functional enrichment and KEGG pathway analyses were performed through the Goatools (https://github.com/tanghaibao/Goatools) and KOBAS (http://kobas.cbi.pku.edu.cn/home.do), respectively, to understand the function of DEGs [65]. DEGs were significantly enriched in GO terms and metabolic pathways when their Bonferroni-corrected P-value is <0.05. The hierarchical clustering analysis of the expression profiles was performed using the hclust

command in R and the default complete linkage method. The R package WGCNA [66] was used to identify the co-expression modules.

Repeat detection, gene prediction, and annotation

We detected the repeat content of the *P. ginseng* genome through an approach combining de novo prediction and homology-based searching. Three de novo prediction programs, namely, PILER-DF V1.0 [67], RepeatModeler V1.0.8 (http://www.repeatmasker.org), and LTR_FINDER V1.06 [68], were used to construct the de novo repeat library. The homology-based approach involves searching commonly used databases of known repetitive sequences. RepeatMasker V4.06 (http://www.repeatmasker.org) was used for the DNA-level identification with Repbase (a database of eukaryotic repetitive elements) using RepeatMasker V4.06 (http://www.repeatmasker.org), and RepeatProteinMask was utilized for protein level identification, which ran WuBlastX against the TE protein database. The tandem repeats in the genome assembly were identified through the tandem repeat finder.

The gene models of the *P. ginseng* genome were predicted using the MAKER-P pipeline [69]. The available ginseng EST, mRNA datasets, and protein datasets were used to generate the first-pass gene annotation. The resulting GFF3 file was used for *ab initio* gene predictor SNAP training [70]. The 75,878 transcripts assembled from the RNA-Seq data were used as transcript clues for the second-pass MAKER-P annotation. For further gene function annotation, the transcript encoding the longest protein sequence for each gene was defined as the representative sequence. First, each protein was searched against the NR [71], KOG [72], and Swiss-Prot [73] databases using BLASTx. The best similar hit with an E-value <1.0e−5 was considered the

gene annotation information. Second, each protein was annotated according to the GO database [74], and Blast2GO was used to obtain GO terms representing a biological process, cellular component, and molecular function. Finally, all proteins were searched against the KEGG database [75] with the KAAS tool (http://www.genome.jp/tools/kaas/). Multiple plant organisms were selected to obtain the KEGG ortholog IDs of the best homologous genes.

Gene family identification and phylogenetic analysis

Thirteen other diploid plant genomes were used for cluster identification to determine the ortholog genes and to elucidate the evolution of the genome, in addition to the *P. ginseng* genome (Supplementary Table S9). The longest representative sequence of each genome under the pairwise sequence similarities among all input proteins was calculated using an all-by-all BlastP with an E-value 1e−10, which was used to cluster the genes by OrthoMCL [76]. The peptide sequences from 383 single copy orthologous gene clusters were extracted to construct a phylogenetic tree and estimate the divergence time. After the multiple sequence alignment by MUSCLE [77] and the poorly aligned region removal by GBLOCKs [78], the high-quality blocks were converted (back-translation) in CDS and concatenated into one super-gene for each species. With these super-genes, a phylogenetic tree was constructed with RAxML through the PROTGAMMAJTT model [79].

The divergence time was estimated by MCMCtree program with 10,000 sampling times, 50 sampling rate, and 50,000 iteration burn-ins [80]. Two runs were performed to ensure convergence. The divergence time between monocots–dicots (140-150 Mya) or Arabidopsis–tomato or grape–tomato (110-124 Mya) was used to calibrate the divergence time [81-83]. Four

species were selected for the lineage-specific evolutionary rate estimation with codeML through the free-ratio model. The genes with dS >3 or dN/dS >3 were filtered. Furthermore, the codeML with the branch-site model was used to estimate the branch-based ratio of nonsynonymous to synonymous substitution rate (ω or dN/dS). The branch-site model parameters were set as follows: null hypothesis: model = 2, NSsites = 2, fix_omega = 1, omega = 1; alternative hypothesis: model = 2, NSsites = 2, fix_omega = 0, omega = 1.

UGT family analysis, molecule modeling, and docking

Multiple alignments were performed using cluster X2 [84]. Phylogenetic trees were generated through MEGA 5.0 software [85]. The genetic distances were estimated using the pairwise distance amino acid substitution matrix with 100 bootstrap replicates.

The coordinates in pdb format of the small molecules protopanaxadiol and protopanaxatriol were built using the Corina (https://www.mn-am.com/online_demos/corina_demo_interactive). The homology models of the 18 UGTs from *P. ginseng* were built using the crystal structures as templates searched through the Swiss-model server http://swissmodel.expasy.org [86]. The docking of the protopanaxadiol or protopanaxatriol and the UDP-glucose in the constructed models was performed with Patchdock at http://bioinfo3D.cs.tau.ac.il/Patchdock [87, 88]. The ligand docking results were visualized with PyMOL molecular graphics system [89].

Funding

This work is supported by the grants from the National Natural Science Foundation of China (81403053, 81503469), the China Academy of Chinese Medical Sciences (ZZ0808021), the

Guangdong Provincial Hospital of Chinese Medicine Special Fund (2015KT1817), the China Academy of Chinese Medical Sciences Special Fund for Health Service Development of Chinese Medicine (ZZ0908067), and National Cancer Institute, NIH, USA (CA154295).

Availability of supporting data and materials

The genome assemblies and annotation are available through our website at http://ginseng.vicp.io:23488/. The sequencing data of genome and transcriptome were deposited at GigaDB [33].

Author contributions

CSL and CYC initiated the study, designed the experiments, reviewed the data, and drafted the manuscript. CY, XSM, YQG, BR, ZJJ, ZXY, ZJ, JZW, LZX, ZLJ, CRY, ZGW and RW designed and performed the experiments. XJ, LBS, SH, QJ, WML, LGZ, ZL, ZhuYJ, ZC and QLR analyzed the data. XJ, XSM, CY, LBS, DLL, LXW, ZhangYJ, DA, RN and HZH wrote the manuscript.

Abbreviations

AACT Acetyl-CoA C-acetyltransferase

BUSCOs Benchmarking Universal Single-Copy Orthologs

CAS cycloartenol synthase

CDS Coding sequence

DDS dammarenediol synthase

DESI-MS Desorption Electrospray Ionization-Mass Spectrometry

DMAPP dimethylallyl diphosphate

DXR 1-deoxy-D-xylulose-5-phosphate reductoisomerase

DXS 1-deoxy-D-xylulose-5-phosphate synthas

EST expression sequences tags

FPP farnesyl diphosphate

FPS farnesyl diphosphate synthase

GT glycosyltransferase

HMBPP (E)-4-Hydroxy-3-methyl-but-2-enyl pyrophosphate

HMGCoA 3-hydroxy-3-methylglutaryl-CoA

HMGR 3-hydroxy-3-methylglutaryl-CoA reductase

HMGS 3-hydroxy-3-methylglutaryl- CoA synthase

HPLC High Performance Liquid Chromatography

IDI isopentenyl-diphosphate delta-isomerase

IPP isopentenyl diphosphate

IPP Isopentenyl diphosphate

LAS lanosterol synthase

LTR long terminal repeat

MEP 2-C-Methyl-D-erythritol 4-phosphate

MVA mevalonic acid

MVD mevalonate diphosphate decarboxylase

MVK mevalonate kinase

MVP mevalonate phosphate

MVPP diphosphomevalonate

Myr million years

OAS oleanolic acid synthase

PMK phosphomevalonate kinase

PPD protopanaxadiol

PPDS protopanaxadiol synthase

PPT protopanaxatriol

PPTS protopanaxatriol synthase

SE squalene epoxidase

SQS Squalene synthase

SS squalene synthase

UDP uridine diphosphate

UGT UDP-glycosyltransferase

WGCNA weighted gene coexpression network analysis

β-AS β-amyrin synthase

Competing financial interests

The authors declare no competing financial interests.

References

1. Hemmerly TE. A ginseng farm in Lawrence County, Tennessee. Econ Bot. 1977;31(2):160-2.

2. Leung KW. Pharmacology of Ginsenosides. In: Ramawat KG, Mérillon J-M, editors. Natural Products. Berlin Heidelberg: Springer; 2013. p. 3497-514.
3. Leung KW, Wong ST. Pharmacology of ginsenosides: a literature review. Chin Med. 2010;5(1):20.
4. Yun T-K. Brief introduction of *Panax ginseng* C. A. Meyer. J Korean Med Sci. 2001;16(Suppl):S3-5.
5. Zhang YC, Li G, Jiang C, Yang B, Yang HJ, Xu HY, et al. Tissue-specific distribution of ginsenosides in different aged ginseng and antioxidant activity of ginseng leaf. Molecules. 2014;19(11):17381-99.
6. Fukuda N, Shan S, Tanaka H, Shoyama Y. New staining methodology: eastern blotting for glycosides in the field of Kampo medicines. J Nat Med. 2005;60(1):21-7.
7. Taira S, Ikeda R, Yokota N, Osaka I, Sakamoto M, Kato M, et al. Mass spectrometric imaging of ginsenosides localization in *Panax ginseng* root. Am J Chin Med. 2010;38(3):485-93.
8. Yokota S, Onohara Y, Shoyama Y. Immunofluorescence and immunoelectron microscopic localization of medicinal substance, Rb1, in several plant parts of *Panax ginseng*. Curr Drug Disc Technol. 2011;8(1):51-9.
9. Christensen LP, Jensen M, Kidmose U. Simultaneous determination of ginsenosides and polyacetylenes in American ginseng root (*Panax quinquefolium* L.) by high-performance liquid chromatography. J Agric Food Chem. 2006;54(24):8995-9003.
10. Tani T, Kubo M, Katsuki T, Higashino M, Hayashi T, Arichi S. Histochemistry II. Ginsenosides in ginseng (*Panax ginseng*, Root). J Nat Prod. 1981;44(4):401-7.
11. Augustin JM, Kuzina V, Andersen SB, Bak S. Molecular activities, biosynthesis and evolution of triterpenoid saponins. Cheminform. 2011;72(28):435-57.
12. Haralampidis K, Trojanowska M, Osbourn AE. Biosynthesis of triterpenoid saponins in plants. Adv Biochem Eng Biotechnol. 2002;75(75):31-49.
13. Jenner H, Townsend BJ, Osbourn A. Unravelling triterpene glycoside synthesis in plants: phytochemistry and functional genomics join forces. Planta. 2005;220(4):503-6.
14. Liang Y, Zhao S. Progress in understanding of ginsenoside biosynthesis. Plant Biol. 2008;10(4):415-21.
15. Osbourn A, Goss RJM, Field RA. The saponins: polar isoprenoids with important and diverse biological activities. Nat Prod Rep. 2011;28(7):1261-8.
16. Sawai S, Saito K. Triterpenoid biosynthesis and engineering in plants. Front Plant Sci. 2011;2(25):25.
17. Thimmappa R, Geisler K, Louveau T, O'Maille P, Osbourn A. Triterpene biosynthesis in plants. Annu Rev Plant Biol. 2014;65(65):225-57.
18. Lee M-H, Jeong J-H, Seo J-W, Shin C-G, Kim Y-S, In J-G, et al. Enhanced triterpene and phytosterol biosynthesis in *Panax ginseng* overexpressing squalene synthase gene. Plant Cell Physiol. 2004;45(8):976-84.
19. Han J-Y, In J-G, Kwon Y-S, Choi YE. Regulation of ginsenoside and phytosterol biosynthesis by RNA interferences of squalene epoxidase gene in *Panax ginseng*. Phytochemistry. 2009;71(1):36-46.

20. Phillips DR, Rasbery JM, Bartel B, Matsuda SP. Biosynthetic diversity in plant triterpene cyclization. Curr Opin Plant Biol. 2006;9(3):305-14.
21. Han J-Y, Kim H-J, Kwon Y-S, Choi Y-E. The Cyt P450 enzyme CYP716A47 catalyzes the formation of protopanaxadiol from dammarenediol-II during ginsenoside biosynthesis in *Panax ginseng*. Plant Cell Physiol. 2011;52(12):2062-73.
22. Han JY, Hwang HS, Choi SW, Kim HJ, Choi YE. Cytochrome P450 CYP716A53v2 catalyzes the formation of protopanaxatriol from protopanaxadiol during ginsenoside biosynthesis in *Panax Ginseng*. Plant Cell Physiol. 2012;53(9):1535-45.
23. Han J-Y, Kim M-J, Ban Y-W, Hwang H-S, Choi Y-E. The involvement of β-amyrin 28-oxidase (CYP716A52v2) in oleanane-type ginsenoside biosynthesis in *Panax ginseng*. Plant Cell Physiol. 2013;54(12):2034-46.
24. Li C, Zhu Y, Xu G, Chao S, Luo H, Song J, et al. Transcriptome analysis reveals ginsenosides biosynthetic genes, microRNAs and simple sequence repeats in *Panax ginseng* C. A. Meyer. BMC Genomics. 2013;14(1):245.
25. Lahoucine Achnine, David V. Huhman, Mohamed A. Farag, Lloyd W. Sumner, Jack W. Blount, Dixon RA. Genomics-based selection and functional characterization of triterpene glycosyltransferases from the model legume *Medicago truncatula*. Plant J. 2005;41(6):875–87.
26. Meesapyodsuk D, Balsevich J, Reed DW, Covello PS. Saponin biosynthesis in *Saponaria vaccaria*. cDNAs encoding beta-amyrin synthase and a triterpene carboxylic acid glucosyltransferase. Plant Physiol. 2007;143(2):959-69.
27. Augustin JM, Drok S, Shinoda T, Sanmiya K, Nielsen JK, Khakimov B, et al. UDP-glycosyltransferases from the UGT73C subfamily in *Barbarea vulgaris* catalyze sapogenin 3-O-glucosylation in saponin-mediated insect resistance. Plant Physiol. 2012;160(4):1881-95.
28. Shibuya M, Nishimura K, Yasuyama N, Ebizuka Y. Identification and characterization of glycosyltransferases involved in the biosynthesis of soyasaponin I in *Glycine max*. FEBS Lett. 2010;584(11):2258-64.
29. Wang P, Wei Y, Fan Y, Liu Q, Wei W, Yang C, et al. Production of bioactive ginsenosides Rh2 and Rg3 by metabolically engineered yeasts. Metab Eng. 2015;29:97-105.
30. Yan X, Fan Y, Wei W, Wang P, Liu Q, Wei Y, et al. Production of bioactive ginsenoside compound K in metabolically engineered yeast. Cell Res. 2014;24(6):770-3.
31. Jung S-C, Kim W, Park SC, Jeong J, Park MK, Lim S, et al. Two ginseng UDP-glycosyltransferases synthesize ginsenoside Rg3 and Rd. Plant Cell Physiol. 2014;55(12):2177-88.
32. Wei W, Wang P, Wei Y, Liu Q, Yang C, Zhao G, et al. Characterization of *Panax ginseng* UDP-glycosyltransferases catalyzing protopanaxatriol and biosyntheses of bioactive ginsenosides F1 and Rh1 in metabolically engineered yeasts. Mol Plant. 2015;8(9):1412-24.
33. Xu J, Chu Y, Xiao S, Liao B, Yin Q, Bai R, et al. *De novo* sequencing of *Panax ginseng*. GigaScience Database. 2017.
34. Grabherr MG, Haas BJ, Yassour M, Levin JZ, Thompson DA, Amit I, et al. Full-length transcriptome assembly from RNA-Seq data without a reference genome. Nat Biotechnol. 2011;29(7):644-52.

35. Simao FA, Waterhouse RM, Ioannidis P, Kriventseva EV, Zdobnov EM. BUSCO: assessing genome assembly and annotation completeness with single-copy orthologs. Bioinformatics. 2015;31(19):3210-2.

36. Choi H-I, Waminal NE, Park HM, Kim N-H, Choi BS, Park M, et al. Major repeat components covering one-third of the ginseng (*Panax ginseng* C. A. Meyer) genome and evidence for allotetraploidy. Plant J. 2014;77(6):906–16.

37. Ruhfel BR, Gitzendanner MA, Soltis PS, Soltis DE, Burleigh JG. From algae to angiosperms–inferring the phylogeny of green plants (*Viridiplantae*) from 360 plastid genomes. BMC Evol Biol. 2014;14:23.

38. Wang K, Jiang S, Sun C, Lin Y, Rui Y, Yi W, et al. The spatial and temporal transcriptomic landscapes of ginseng, *Panax ginseng* C. A. Meyer. Sci Rep. 2015;5:18283.

39. Chen S, Song J, Sun C, Xu J, Zhu Y, Verpoorte R, et al. Herbal genomics: examining the biology of traditional medicines. Science. 2015;347(6219):S27-S9.

40. Chen S, Song J. Herbgenomics. China Journal of Chinese Materia Medica. 2016;41(21):3881-9.

41. Chen S, Xu J, Liu C, Zhu Y, Nelson DR, Zhou S, et al. Genome sequence of the model medicinal mushroom *Ganoderma lucidum*. Nat Commun. 2012;3(2):913.

42. Huang Z, Xu J, Xiao S, Liao B, Gao Y, Zhai C, et al. Comparative optical genome analysis of two pangolin species: *Manis pentadactyla* and *Manis javanica*. GigaScience. 2016;5(1):1-5.

43. Guan R, Zhao Y, Zhang H, Fan G, Liu X, Zhou W, et al. Draft genome of the living fossil *Ginkgo biloba*. GigaScience. 2016;5(1):49.

44. Cai J, Liu X, Vanneste K, Proost S, Tsai W-C, Liu K-W, et al. The genome sequence of the orchid *Phalaenopsis equestris*. Nat Genet. 2015;47(2):65.

45. Matsumoto T, Wu J, Kanamori H, Katayose Y, Fujisawa M, Namiki N, et al. The map-based sequence of the rice genome. Nature. 2005;436(7052):793-800.

46. Jaillon O, Aury J-M, Noel B, Policriti A, Clepet C, Casagrande A, et al. The grapevine genome sequence suggests ancestral hexaploidization in major angiosperm phyla. Nature. 2007;449(7161):463-7.

47. Paterson AH, Bowers JE, Bruggmann R, Dubchak I, Grimwood J, Gundlach H, et al. The *Sorghum bicolor* genome and the diversification of grasses. Nature. 2009;457(7229):551-6.

48. Mach J. Mass spectrometry imaging with single-cell resolution: spatial distribution of lipids in cotton seeds. Plant Cell. 2012;24(2):371.

49. Li B, Hansen SH, Janfelt C. Direct imaging of plant metabolites in leaves and petals by desorption electrospray ionization mass spectrometry. Int J Mass spectrom. 2013;348(2):15-22.

50. Schramek N, Huber C, Schmidt S, Dvorski S, Knispel N, Ostrozhenkova E, et al. Biosynthesis of ginsenosides in field-grown *Panax ginseng*. JSM Biotechnol Biomed Eng. 2014;2(1):1033.

51. Kim Y-J, Zhang D, Yang D-C. Biosynthesis and biotechnological production of ginsenosides. Biotechnol Adv. 2015;33(6):717-35.

52. Kim T-D, Han J-Y, Huh GH, Choi YE. Expression and functional characterization of three squalene synthase genes associated with saponin biosynthesis in *Panax ginseng*. Plant Cell Physiol. 2011;52(1):125-37.

53. Kim YJ, Lee OR, Oh JY, Jang MG, Yang DC. Functional analysis of 3-hydroxy-3-methylglutaryl coenzyme a reductase encoding genes in triterpene saponin-producing ginseng. Plant Physiol. 2014;165(1):373-87.
54. Yang W, Zhang Y, Wu W, Huang L, Guo D, Liu C. Approaches to establish Q-markers for the quality standards of traditional Chinese medicines. Acta Pharmaceutica Sinica B. 2017; http://dx.doi.org/10.1016/j.apsb.2017.04.012.
55. Jiang H, Lei R, Ding SW, Zhu S. Skewer: a fast and accurate adapter trimmer for next-generation sequencing paired-end reads. BMC Bioinformatics. 2014;15(1):182.
56. Luo R, Liu B, Xie Y, Li Z, Huang W, Yuan J, et al. SOAPdenovo2: an empirically improved memory-efficient short-read *de novo* assembler. GigaScience. 2012;1(1):18.
57. Chikhi R, Medvedev P. Informed and automated *k*-mer size selection for genome assembly. Bioinformatics. 2014;30(1):31-7.
58. Boetzer M, Henkel CV, Jansen HJ, Butler D, Pirovano W. Scaffolding pre-assembled contigs using SSPACE. Bioinformatics. 2011;27(4):578-9.
59. Li R, Zhu H, Ruan J, Qian W, Fang X, Shi Z, et al. *De novo* assembly of human genomes with massively parallel short read sequencing. Genome Res. 2010;20(2):265-72.
60. Li H, Durbin R. Fast and accurate short read alignment with Burrows-Wheeler transform. Bioinformatics. 2009;25(14):1754-60.
61. Zdobnov EM, Tegenfeldt F, Kuznetsov D, M.Waterhouse R, Simao FA, Ioannidis P, et al. OrthoDB v9.1: cataloging evolutionary and functional annotations for animal, fungal, plant, archaeal, bacterial and viral orthologs. Nucleic Acids Res. 2016;45(D1):D744-9.
62. Altschul SF, Gish W, Miller W, Myers EW, Lipman DJ. Basic local alignment search tool. J Mol Biol. 1990;215(3):403-10.
63. Langmead B, Salzberg SL. Fast gapped-read alignment with Bowtie 2. Nat Methods. 2012;9(4):357-9.
64. Trapnell C, Hendrickson DG, Sauvageau M, Goff L, Rinn JL, Pachter L. Differential analysis of gene regulation at transcript resolution with RNA-seq. Nat Biotechnol. 2013;31(1):46-53.
65. Xie C, Mao X, Huang J, Ding Y, Wu J, Dong S, et al. KOBAS 2.0: a web server for annotation and identification of enriched pathways and diseases. Nucleic Acids Res. 2011;39(suppl 2):W316-22.
66. Langfelder P, Horvath S. WGCNA: an R package for weighted correlation network analysis. BMC Bioinformatics. 2008;9:559.
67. Edgar RC, Myers EW. PILER: identification and classification of genomic repeats. Bioinformatics. 2005;21(suppl 1):i152-8.
68. Xu Z, Wang H. LTR_FINDER: an efficient tool for the prediction of full-length LTR retrotransposons. Nucleic Acids Res. 2007;35(suppl 2):W265-8.
69. Campbell MS, Law M, Holt C, Stein JC, Moghe GD, Hufnagel DE, et al. MAKER-P: a tool kit for the rapid creation, management, and quality control of plant genome annotations. Plant Physiol. 2014;164(2):513-24.
70. Johnson AD, Handsaker RE, Pulit SL, Nizzari MM, O'Donnell CJ, Bakker PIWd. SNAP: a web-based tool for identification and annotation of proxy SNPs using HapMap. Bioinformatics. 2008;24(24):2938-9.

71. Yangyang D, Jianqi L, Songfeng W, Yunping Z, Yaowen C, Fuchu H. Integrated nr database in protein annotation system and its localization. Computer Engineering. 2006;32(5):71-2.
72. Koonin EV, Fedorova ND, Jackson JD, Jacobs AR, Krylov DM, Makarova KS, et al. A comprehensive evolutionary classification of proteins encoded in complete eukaryotic genomes. Genome Biol. 2004;5(2):R7.
73. Apweiler R, Bairoch A, Wu CH, Barker WC, Boeckmann B, Ferro S, et al. UniProt: the Universal Protein knowledgebase. Nucleic Acids Res. 2004;32(suppl 1):D115-9.
74. Ashburner M, Ball CA, Blake JA, Botstein D, Butler H, Cherry JM, et al. Gene ontology: tool for the unification of biology. Nat Genet. 2000;25(1):25-9.
75. Kanehisa M, Goto S, Kawashima S, Okuno Y, Hattori M. The KEGG resource for deciphering the genome. Nucleic Acids Res. 2003;32(suppl 1):D277-80.
76. Li L, Stoeckert CJ, Roos DS. OrthoMCL: identification of ortholog groups for eukaryotic genomes. Genome Res. 2003;13(9):2178-89.
77. Edgar RC. MUSCLE: multiple sequence alignment with high accuracy and high throughput. Nucleic Acids Res. 2004;32(5):1792-7.
78. Talavera G, Castresana J. Improvement of phylogenies after removing divergent and ambiguously aligned blocks from protein sequence alignments. Syst Biol. 2007;56(4):564-77.
79. Stamatakis A. RAxML Version 8: a tool for phylogenetic analysis and post-analysis of large phylogenies. Bioinformatics. 2014;30(9):1312-3.
80. Yang Z. PAML: a program package for phylogenetic analysis by maximum likelihood. Bioinformatics. 1997;13(5):555-6.
81. Huang C-H, Sun R, Hu Y, Zeng L, Zhang N, Cai L, et al. Resolution of Brassicaceae phylogeny using nuclear genes uncovers nested radiations and supports convergent morphological evolution. Mol Biol Evol. 2016;33(2):394-412.
82. Massoni J, Couvreur TL, Sauquet H. Five major shifts of diversification through the long evolutionary history of Magnoliidae (angiosperms). BMC Evol Biol. 2015;15:49.
83. Barreda VD, Palazzesi L, Tellería MC, Olivero EB, Raine JI, Forest F. Early evolution of the angiosperm clade Asteraceae in the Cretaceous of Antarctica. Proc Natl Acad Sci U S A. 2015;112(35):10989-94.
84. Jeanmougin F, Thompson JD, Gouy M, Higgins DG, Gibson TJ. Multiple sequence alignment with Clustal X. Trends Biochem Sci. 1998;23(10):403-5.
85. Tamura K, Dudley J, Nei M, Kumar S. MEGA4: molecular evolutionary genetics analysis (MEGA) software version 4.0. Mol Biol Evol. 2007;24(8):1596-9.
86. Schwede T, Kopp J, Guex N, Peitsch MC. SWISS-MODEL: an automated protein homology-modeling server. Nucleic Acids Res. 2003;31(13):3381-5.
87. Duhovny D, Nussinov R, Wolfson HJ. Efficient unbound docking of rigid molecules. Lect Notes Comput Sci. 2002;2452:185-200.
88. Schneidman-Duhovny D, Inbar Y, Nussinov R, Wolfson HJ. PatchDock and SymmDock: servers for rigid and symmetric docking. Nucleic Acids Res. 2005;33(suppl 2):W363-7.
89. Seeliger D, Groot BLd. Ligand docking and binding site analysis with PyMOL and Autodock/Vina. J Comput Aided Mol Des. 2010;24(5):417-22.

Figure legends

Fig. 1 *P. ginseng* genome assembly and functional gene annotations. a Statistical analysis of the *P. ginseng* draft genome. **b** Phylogenetic tree and divergence data of 14 species, including *P. ginseng*, based on the proteins of 383 single-copy genes annotated to the genome sequence of each species. **c** Distribution of orthologous gene families in *P. ginseng* and four sequenced species: carrot (*Daucus carota*), coffee (*Coffea canephora*), *Arabidopsis* (*Arabidopsis thaliana*), and tomato (*Solanum lycopersicum*).

Fig. 2 Ginsenoside distribution in the *P. ginseng* root cross sections that obtained through mass spectrometric imaging based on the desorption electrospray ionization-mass spectrometry (DESI–MS). a Optical image of the main root. **b** TMS image spectrum. **c** DESI–MS image of metabolites and ginsenosides: maltose, citbismine C, Rg1/Rf, pseudo-Rc1, Ra1/Ra2, Rd/Re, Rs1/Rs2, and Ra3. Scale bar=2 mm.

Fig. 3 Metabolism and transcriptome analysis of *P. ginseng* root. a HPLC chromatograms of the ginsenosides Rg1, Re, Rf, Rg2, Rb1, Rc, Rb2, and Rd standards. **b** PCA score plots based on the HPLC dataset (●periderm, ●cortex, and ●stele). **c** PLS-DA score plots based on the HPLC dataset. **d** Cluster tree of the ginseng samples based on the expression pattern of 42006 genes. The leaves of the tree correspond to the different ginseng tissue samples (periderm, Per; cortex, Cor; stele, Ste). The color bands beneath the tree represent the relative content of the total ginsenosides, Rb1 and Rg1 (red indicates high values).

Fig. 4 Gene expression in the MVA pathway for ginsenosides in *P. ginseng*. a Possible biosynthesis pathway for ginsenosides with the designated candidate genes. AACT, acetyl-CoA C-acetyltransferase; HMGS, 3-hydroxy-3-methylglutaryl-CoA synthase; HMGCoA, 3-hydroxy-3-methylglutaryl-CoA; HMGR, 3-hydroxy-3-methylglutaryl-CoA reductase; MVK, mevalonate kinase; MVP, mevalonate phosphate; PMK, phosphomevalonate kinase; MVPP, diphosphomevalonate; MVD, mevalonate diphosphate decarboxylase; IPP, isopentenyl diphosphate; DMAPP, dimethylallyl diphosphate; IDI, isopentenyl-diphosphate delta-isomerase; FPS, farnesyl diphosphate synthase; FPP, farnesyl diphosphate; SS, squalene synthase; SE, squalene epoxidase; β-AS, β-amyrin synthase; DDS, dammarenediol synthase; LAS, lanosterol synthase; CAS, cycloartenol synthase; OAS, oleanolic acid synthase; PPDS, protopanaxadiol synthase; PPTS, protopanaxatriol synthase. **b** Heatmap of the candidate biosynthesis pathway gene expression patterns in nine organs from *P. ginseng*.

Fig. 5 Sequence analysis and transcript levels of the HMGR gene family. a Phylogenetic analysis of PgHMGRs and characterized HMGRs from other plants. **b** Multiple alignments of the amino acid sequences of PgHMGRs with homologous HMGRs from *Arabidopsis*. The black boxes indicate identical residues; the gray boxes represent identical residues for at least two of the sequences. Functional domains are highlighted in colored boxes (red, membrane domain; green, linker domain; and blue, catalytic domain). The two putative HMGR-CoA-binding sites, two NADP(H)-binding sites, and ER retention motifs are denoted by square boxes. **c** Tissue-specific PgHMGR expression patterns in 4-year-old roots. The data represent the mean ± SD of the three independent samples. **d** Genomic DNA structure of PgHMGRs. The exons

are represented by the green-filled square boxes. The lines between the boxes correspond to the introns. The numbers above the exons indicate the length in bp.

Fig. 6 Analysis of UGTs from *P. ginseng*. a All the identified UGTs which newly classified according to the standardization of the UGT Nomenclature Committee were assigned to 24 subfamilies. **b** The expression (lower) of UGT gene copies (PG22765) from the same scaffold (upper) in the different tissues of *P. ginseng*.

Figure 1

Click here to download Figure Figure 1.tif

a

	Size (bp)	Number
**Contig**		
N90	4,516	150,620
N80	8,639	103,388
N70	12,833	75,040
N50	21,977	39,481
Longest	574,138	—
Total size	2,999,700,459	337,439
**Scaffold**		
N90	24,143	33,423
N80	45,718	23,391
N70	65,171	17,168
N50	108,708	9,072
Longest	1,303,414	—
Total size	3,414,349,854	83,074
Gap ratio	12.15%	—

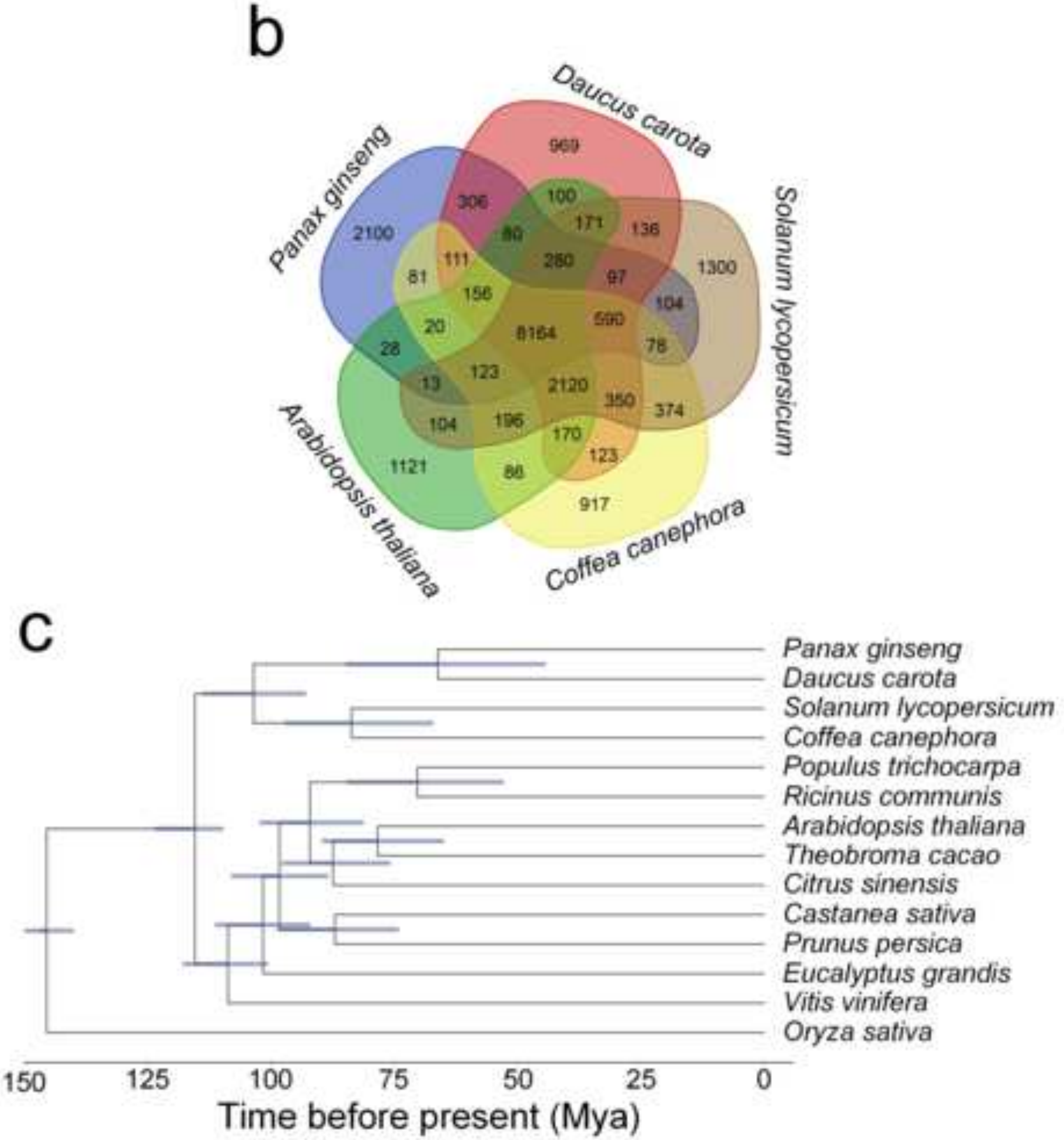

Figure 2

Click here to download Figure Figure 2.tif

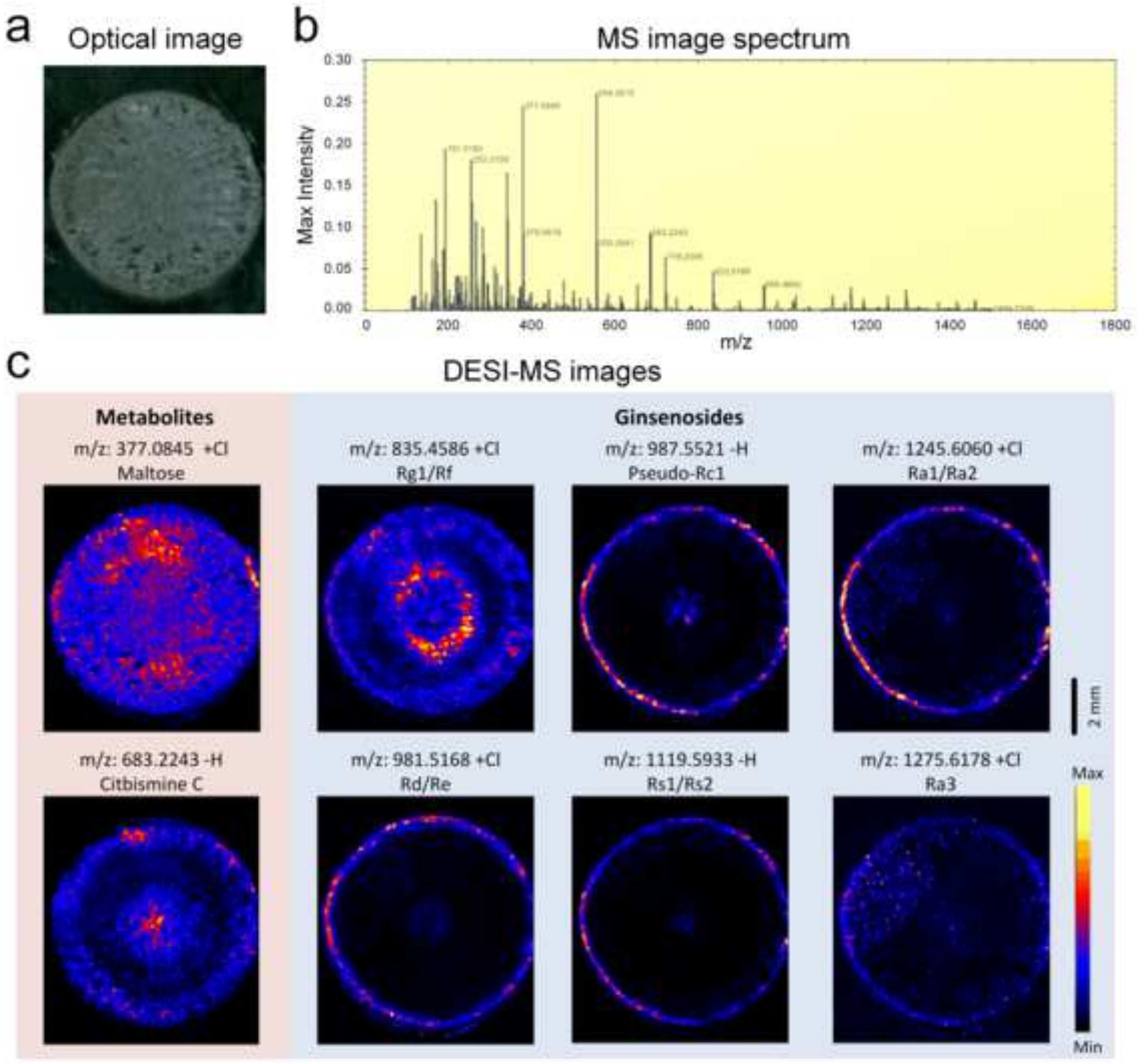

Figure 3

Click here to download Figure Figure 3.tif

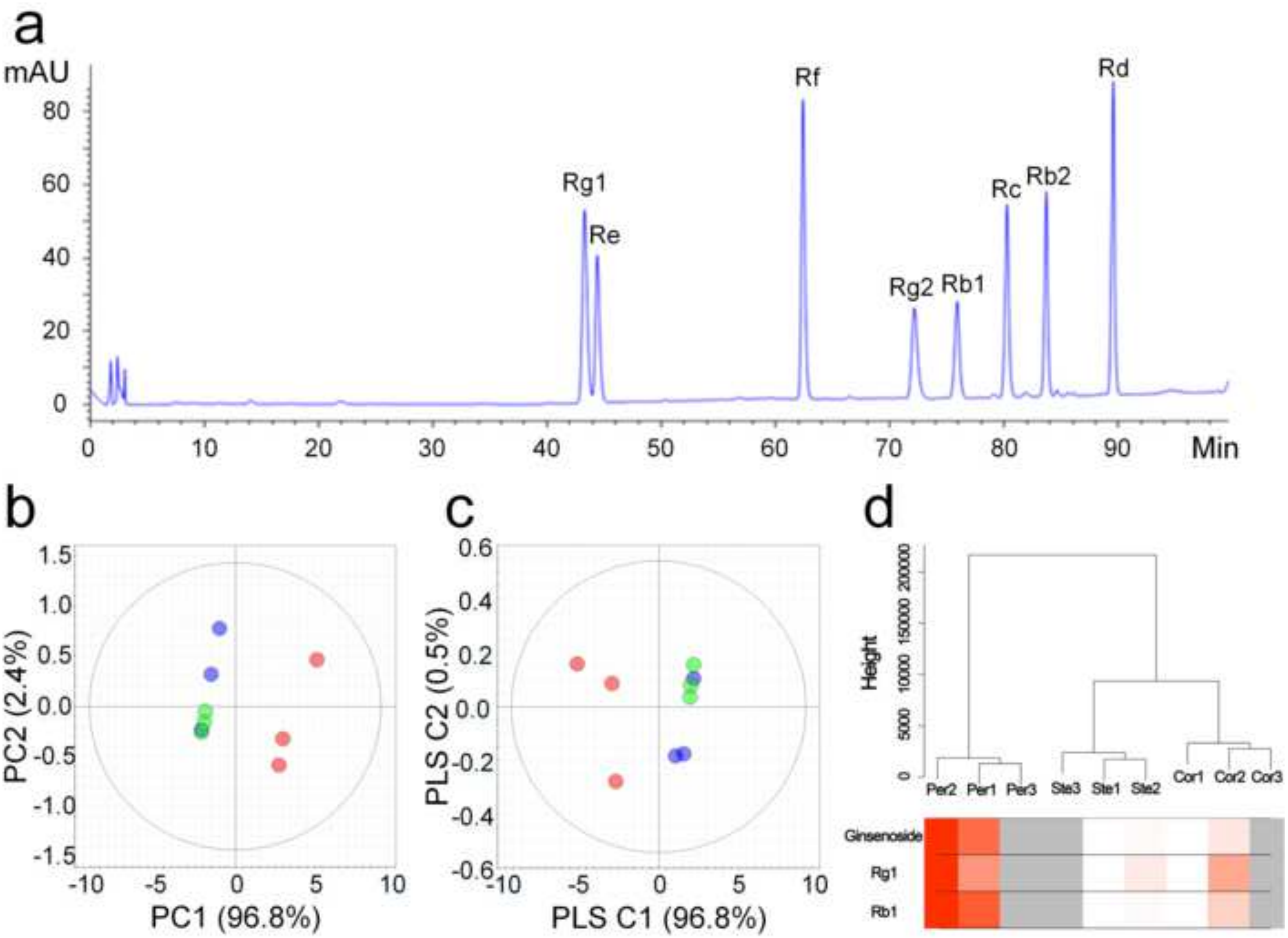

Figure 4

Click here to download Figure Figure 4.tif

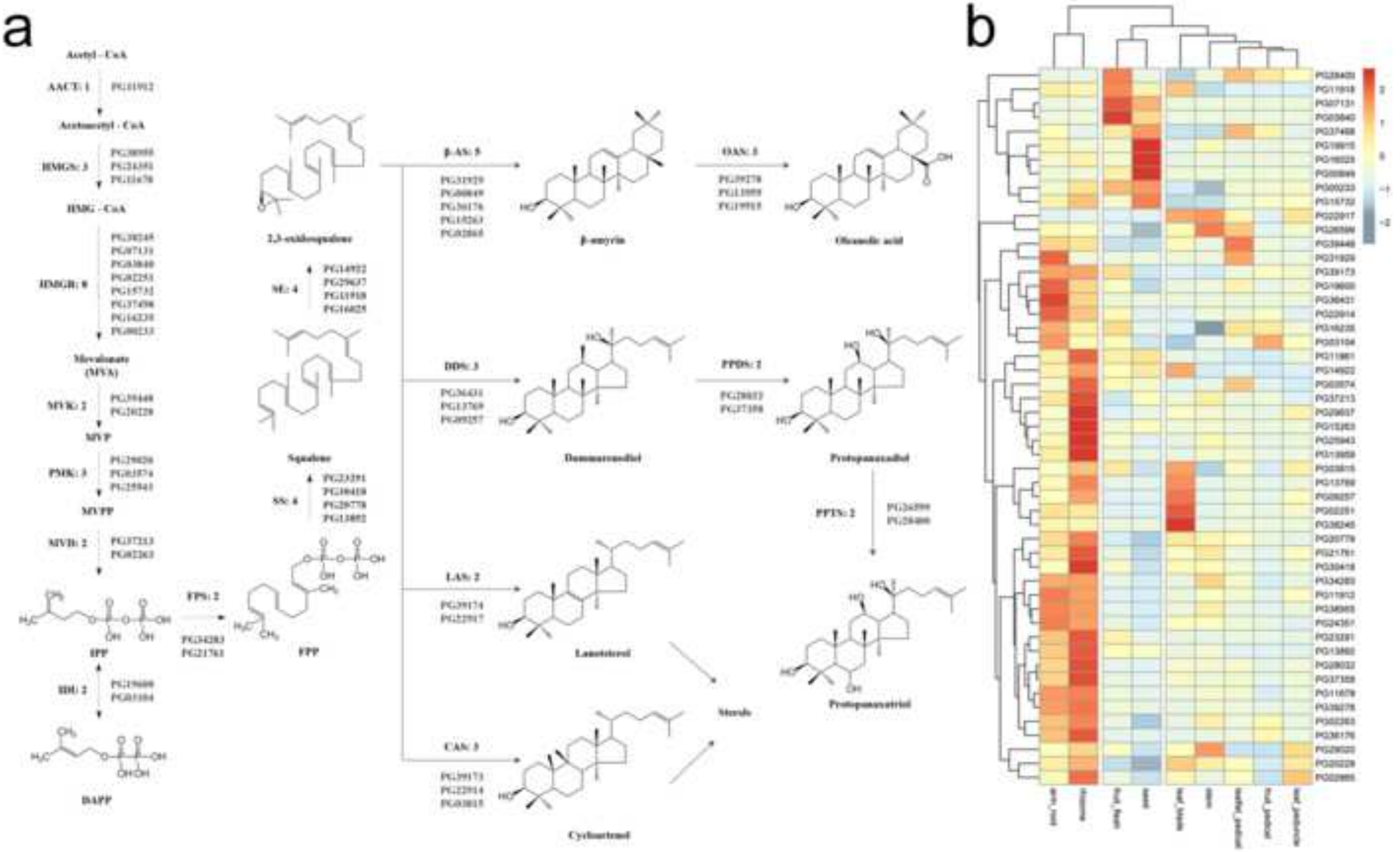

Figure 5 Click here to download Figure Figure 5.tif

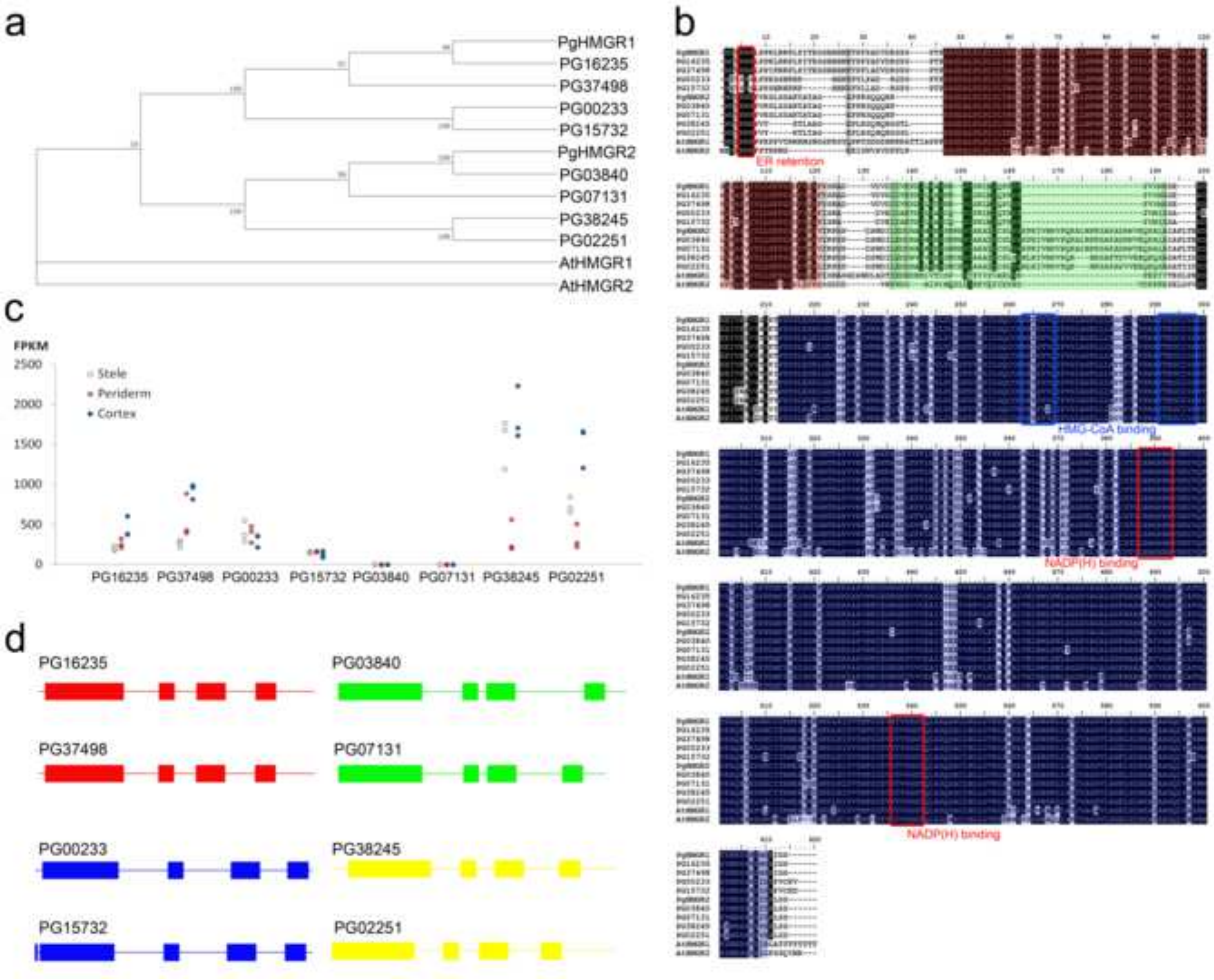

Figure 6

Click here to download Figure Figure 6.tif

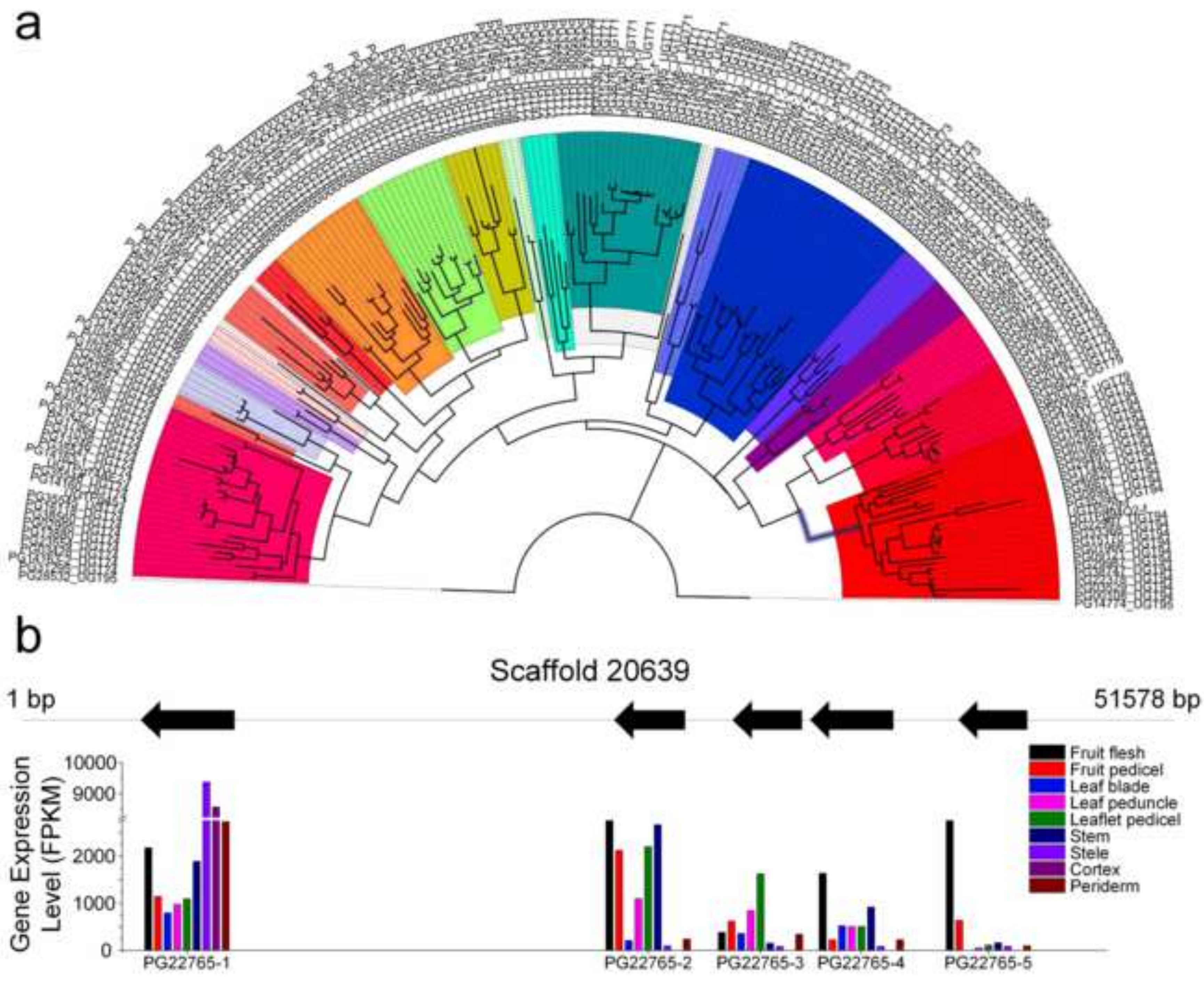

Supplementary Figures

Click here to access/download
Supplementary Material
Supplementary Figures R1.docx

Supplementary Tables

Click here to access/download
Supplementary Material
Supplementary Tables R1.docx

Click here to access/download
Supplementary Material
Supplementary Text R1.docx

 Click here to download Personal Cover Cover letter R1.pdf

Dear Dr. Hans Zauner,

We thank you and the reviewers for your detailed comments, which really benefit our manuscript. We have carefully considered all comments and accordingly revised our manuscript. Please find below our point-by-point replies to the comments and detailed explanations of all changes ("V1" refers to the originally submitted version and "R1" is the revised version; all revisions in R1 were tracked). Thank you!

Reviewer #1: The manuscript entitled: "Ginseng genome examination for ginsenoside biosynthesis" by Xu Jiang *et al.*, presents results of sequencing, characterization and annotation of the *P. ginseng* genome. The authors have further shown the distribution of the ginsenosides molecules in root tissues using mass spectrometry and liquid chromatography, and in order to identify candidate genes possible involved in the ginsenosides biosynthesis pathway an analysis of expression and co-expression networks using RNA-seq data was performed. A set of key genes involved in the ginsenosides biosynthesis were further characterized and interestingly some of them showed tissue specificity and activity during microbial infection. Overall, the experiments and analysis for this study were well conducted. And the results presented in this manuscript are important for the research of its field. However, the manuscript presents major faults that need to be addressed prior publication.
Reply: Thank you for your positive assessment. We have revised our work according to the comments.

Major comments
1. How old were the *P. ginseng* plants used for genome sequencing?
Reply: The ginseng sample we used for genome sequencing came from a 4-year-old *P. ginseng* plant. We have added this information in the method section of R1.

2. The Microbial-resistance section needs to be greatly improved since there is a lot of missing information:
Reply: Thank you for pointing out this deficiency. In V1, we used published data (Gao *et al*. 2016) to recalculate gene expression during fungus infection. Although it is important for ginseng cultivation, this section is still slightly correlated with other parts of the manuscript. Considering the two reviewers' advice, the microbial-resistance section has been removed in R1. Nevertheless, we have attempted to answer the questions below as best as we can. We are truly grateful for the reviewer's comments and suggestions.

a. The statement "Ginsenosides comprise a group of defense metabolites" needs to be supported by a reference.
Reply: Thank you for pointing this out. Indeed, this statement should be supported by some references to increase readers' understanding. Triterpenoid saponins are considered as defense compounds against pathogenic microbes and herbivores (Osbourn, 1996; reviewed by Augustin *et al.*, 2011). A recent study also reveals that unigenes involved in ginsenoside biosynthesis play important roles in the response to *Cylindrocarpon destructans* infection (Gao *et al.*, 2016).

b. What does the authors mean in the statement: "responses to microbial infection are important for ginsenoside biosynthesis"? Is the ginsenosides biosynthesis pathway lead by defense responses of the plant? Does this mean that if the plant is not under biotic stress the ginsenosides are not produced? What happen with the ginsenosides biosynthesis during *C. destructans* infection? Does the ginsenosides increase? Please clarify and support the statement above with a scientific reference.
Reply: Thank you for the reviewer's concerns. We obtained this viewpoint from two aspects. First, the ginsenoside biosynthesis pathway may be activated by microbial infection, and ginsenosides reportedly accumulate during *C. destructans* infection (Chi *et al.*, 2016). Ginsenosides also increase after eliciting SA (Tewari *et al.*, 2011) and MeJA (Lim *et al.*, 2005; Lee *et al.*, 2017). Second, some reports suppose that ginsenosides are phytoalexins and may be the evolution products for microbial resistance (Gao *et al.*, 2016; Hu *et al.*, 2007; Kim *et al.*, 2015). Although we have removed this part in R1, clarifying this statement in future work is still important.

c. The authors mentioned that 1652 resistance genes were annotated base on "plant resistance gene database". Please add the reference for this database.
Reply: Thank you for pointing this out. The Plant Resistance Genes database (PRGdb; http://prgdb.org) is a comprehensive resource of resistance genes (R-genes), a major class of genes in plant genomes that conveys disease resistance against pathogens (Sanseverino *et al.*, 2010).

d. The last paragraph on page 15 starting at "The expression analysis showed that 160 resistance genes …" till

the end, it is not supported by any figure or table and it is not clear what expression analysis were performed. (FPKM?, WGCNA?, qPCR?)
Reply: We apologize for the confusion caused by this paragraph. We used a FPKM analysis result here.

e. The first paragraph on page 16 "The transcriptomes of ginseng induced by *C. destructants* … were recalculated". What does the authors mean by "transcriptomes were recalculated"? Did the authors sequence RNA of infected plants? All this information is missing in the methods section.
Reply: We regret the unclear description. We used the published transcriptome read (Gao *et al.*, 2016) mapping of our genome sequence to recalculate gene expression (FPKM value) during fungus infection. Thus, we did not sequence the RNA of infected plants by ourselves, and information was not described in detail in the method section.

f. In the next statement, starting at "A total of 35008 genes were predicted, of these genes 28481 were expressed … the initial point" has to be supported by evidence. There is not figure or table that shows this analysis. How did the authors get the genes? Did they perform an RNA-seq assembly? If it so, where are the methods and metrics of the RNA-seq assembly?
Reply: We apologize for the confusion. A total of 35 008 genes were identified using time-course RNA-seq data by Gao *et al.* (2016) As we stated in our reply to Question e, the RNA data were from a published paper (Gao *et al.*, 2016). To avoid misunderstanding, we have deleted this section.

g. The statement "At 0.25 DPI, 104 resistance genes were highly expressed and were possibly involved in *C. destructans* recognition" needs to be supported by evidence and a reference. The level of gene expression might not be related with the pathogen recognition at all.
Reply: We completely agree with the reviewer’s point. The resistance-gene expression level may be influenced by many factors, including fungus infection.

h. The next paragraph starting at "At 0.25 DPI" till "Figure S10" describes a GO enrichment test described in figure S10. However figure S10 does not show what described in the text.
Reply: We regret the unclear description. Considering the weak correlation between this microbial-resistance section and the other parts of the manuscript, we have removed this section including the GO enrichment test and Fig. S10 in R1.

i. The entire section is not well described or described at all in methods.
Reply: We agree with the reviewer’s point. The other reviewer also pointed that this section is weakly related to the other parts of the manuscript. Considering both reviewers’ suggestions, we have deleted the entire microbial-resistance section in R1.

1. The methods and tools in methods sections are not properly cited. For example, BLAST, WGCNA, R, etc., lack of reference. Databases such as GO, KEGG, KOG, etc., are not properly cited. The authors should cite the scientific manuscript of the tools and databases and not only the website.
Reply: Thank you for noticing these points that we ignored in V1. We have accordingly corrected these errors and added appropriate references to R1. For example, BLAST (Altschul *et al.*, 1990), WGCNA (Langfelder and Horvath, 2008), GO (Ashburner *et al.*, 2000), KEGG (Kanehisa *et al.*, 2003), and KOG (Koonin *et al.*, 2004) have been cited in R1.

2. Repeat prediction, gene prediction and annotation section in Methods. Page 26. The authors mention that transcripts assembled from RNA-seq data were use for MAKER-P annotation. However there is none information regarding to RNA-seq assembly. What assembler was used? What parameters were used? How many transcripts were obtained? Etc.,The metrics of the transcriptome assembly are not described at all.
Reply: We regret not describing this assembly step in detail. Thank you for pointing this out. We conducted RNA-seq assembly using Trinity software with default parameters and generated 75 878 transcripts, which were used for further gene annotation as RNA evidence. The total length of 75 878 transcripts was 70 273 566 bp, max, min, and the N50 lengths were 12 639, 201, and 1446 bp, respectively.

Minor comments
Page 7. Missing space: "RNA-seqlibraries"
Reply: Thank you for pointing this out. We have revised this fault in line 10, page 7 in R1.

Page 7. Please add the reference for "13 published ginseng RNA-seq data".
Reply: Thank you. The 13 published ginseng RNA-seq data came from two references (Gao *et al.*, 2016; Wang *et al.*, 2015). In R1, we deleted all seven RNA-seq datasets from Gao *et al.* (2016) and added another two datasets from Wang *et al*. (2015).

Page 13. Figure 5. Please rename figures in order of citation. (5a, 5d, 5c, 5b -> 5a, 5b, 5c, 5d)
Reply: Thank you for this kind suggestion. We have revised the order of Fig. 5 in R1.

Page 14. The sentence "The expression patterns of different HMGR types differed" does not make sense. Please re-phrase.
Reply: Thank you for pointing out this error. We have replaced this sentence with "The expression patterns of dissimilar HMGR types differed among various organs" in line 13, page 14 in R1.

Page 20. Add the figure number that support this statement: "same PgHMGR subfamily, they exhibited different correlation patterns with other genes"
Reply: This statement was based on the results shown in Figs. 3b (R1) and 6b (V1). However, this sentence that was related to *C. destructan* infection has been deleted in R1.

Page 22. Genome sequencing and assembly. How old were the plants used in this study?
Reply: We used a 4-year-old *P. ginseng* plant in this study. The information has been added in the method section in R1.

Page 22. Please add the scientific reference for SOAPdenovo assembler.
Reply: Thank you for your suggestion. We have added the reference for SOAPdenovo2 assembler (Luo *et al.*, 2012) as Reference 56 in R1.

Page 23. Last row of the first paragraph. There is missing information for the cutoff criteria. "BLAStN, a cutoff value of 90%". What is this 90%? Identity?
Reply: We meant an identity cutoff value of 90% and have clarified our meaning as "BLASTN, an identity cutoff value of 90%, and a coverage cutoff value of 90%" in lines 13–14, page 21.

Page 28. Please add the reference for PyMOL.
Reply: Thank you for this suggestion. We have added the reference for PyMOL (Seeliger and Groot, 2010) as Reference 89 in R1.

Figures:
Figure 5b. What does it show? FPKM values? Is it an qPCR? Y axis needs a label. In the figure legend the figure was described as a qPCR analysis however nothing of this is described in methods and primers sequences are not provide.
Reply: Fig. 5b shows the FPKM values. The Y-axis-label has been added to Fig. 5b in R1.

Figure S10. The legend of this figure does not provide enough information of the figure.
Reply: Thank you for pointing this out. As answered previously, we have deleted Fig. S10.

Reviewer #2: Xu Jiang *et al.*, present a work on genome assembly and annotation of *Panax ginseng*, with subsequent rna-SEQ analysis and metabolite analysis of root tissues to advance the understanding of the ginsenosides' synthesis pathway. All in all this project is interesting and the current work will merit publication once a number of major comments are resolved. The text could also benefit from some re-organisation to remove duplication, and present the information and technical details in a more concise and ordered fashion. I will focus this review mainly on the sequencing aspects of the paper, with special emphasis on the genome assembly which is my field of expertise.

I feel this paper could gain a lot by tightening up the methods. This will either provide more support for the ginsenoside biosynthesis analysis in which case the focus can highlight that result or if there is no more support for that analysis then the paper can be refocused on a stronger resource description about the genome assembly and differential expression dataset.
Reply: We sincerely appreciate the authoritative and constructive suggestions of Bernardo J. Clavijo and Luis

Yanes. We have revised our work according to the comments.

Major comments
Experimental design, data description and availability:

1) Reads submitted to public archives should be raw, except only for demultiplexing. This is a prerequisite for reproducibility and traceability. All data that has been submitted trimmed or otherwise pre-processed must be resubmitted.
Reply: Thank you for pointing this out. We have already uploaded raw reads to NCBI, and here are the SRA accession numbers:

EXPERIMENT: PG10000 (SRX2955138),
EXPERIMENT: PG250 (SRX2955139),
EXPERIMENT: PG500 (SRX2955140),
EXPERIMENT: PG2000 (SRX2955141), and
EXPERIMENT: PG5000 (SRX2955142).

2) There is no description of the protocols used to generate the paired end data and long mate paired data for genome assembly. Specially with the LMP data, the reads seem untrimmed, but they do contain substantial amounts of short-insert contamination which may be better accounted for if the protocol is properly described.
Reply: We have added the information, including the library construction, on how to generate paired end data and long mate paired data in R1. We apologize for the unclear description in V1 about processing the LMP data. We used Skewer (Jiang *et al.,* 2014) for adapter trimming and low-quality base filtering with these parameters. Transposase adapter sequences were used for adapter searching and trimming, and the maximum mismatch rate was set to 10%; trimming reads were from 3′ end until Q>20. After trimming, reads (2–10 kb) with read length <18 bp or average quality <30 were filtered out.

3) There is no justification for the choice of line IR826 over any other line, and no mention whether the independent samples for differential expression are from a single plant or different plants, and if those are from the IR826 line.
Reply: We had two reasons for selecting line IR826. First, the heterozygosity (1‰–5‰) of this line was relative low, which favored genome assembly. Second, we accumulated some horticultural data of IR826 in a previous study. As IR826 was the strain we used for field planting and was still in its test phase, the samples we used for tissue transcription profile were collected from different plants (some of which were not line IR826).

4) In general, including datasets from different studies for differential expression analysis is not automatically reliable or even comparable. The use of external datasets for every tissue but root needs to be properly supported by analysis that shows statistical validity of equivalent background conditions, plus detailed description of similarities between experimental conditions. Also, the replicate structure of the samples should be the same. While this may be completely true, it is not discussed in the manuscript. If these criteria are not met, there should be explicit description of why this analysis is still considered valuable and its limitations.
Reply: We agree with the reviewer's suggestion. Analyzing new and previous datasets together was faulty. Thus, the transcription analysis of various organs was reformed with new datasets from a single study (Wang *et al.,* 2015). The transcription profile of three different tissues in root was further analyzed for the expression patterns of different HMGR types.

Genome Assembly

5) There is no justification for the choice of assembler and no discussion about parameters (why that particular K, etc). Again, detailed description of the parametrisation for each tool is needed, with some justification when appropriate.
Reply: Thank you for this suggestion. We tested Kmer size using KmerGenie (Chikhi *et al.,* 2014), and it recommended using an 83-mer to assemble. Then, we tried to assemble the Ginseng genome using SOAPdenovo2 and AbySS 1.9, but it ran out of memory after using AbySS 1.9. Consequently, we used 63-, 73-, 83-, and 93-mer to assemble SOAPdenovo2 and obtained the longest N50 when using 83-mer. This information has been added to manuscript R1 (lines 20–21, page 20; lines 1–2, page 21).

6) I analysed the assembly with a KAT spectra-CN plot (using k=31, only the short-insert libraries), with the following result:
This leads to a number of comments:

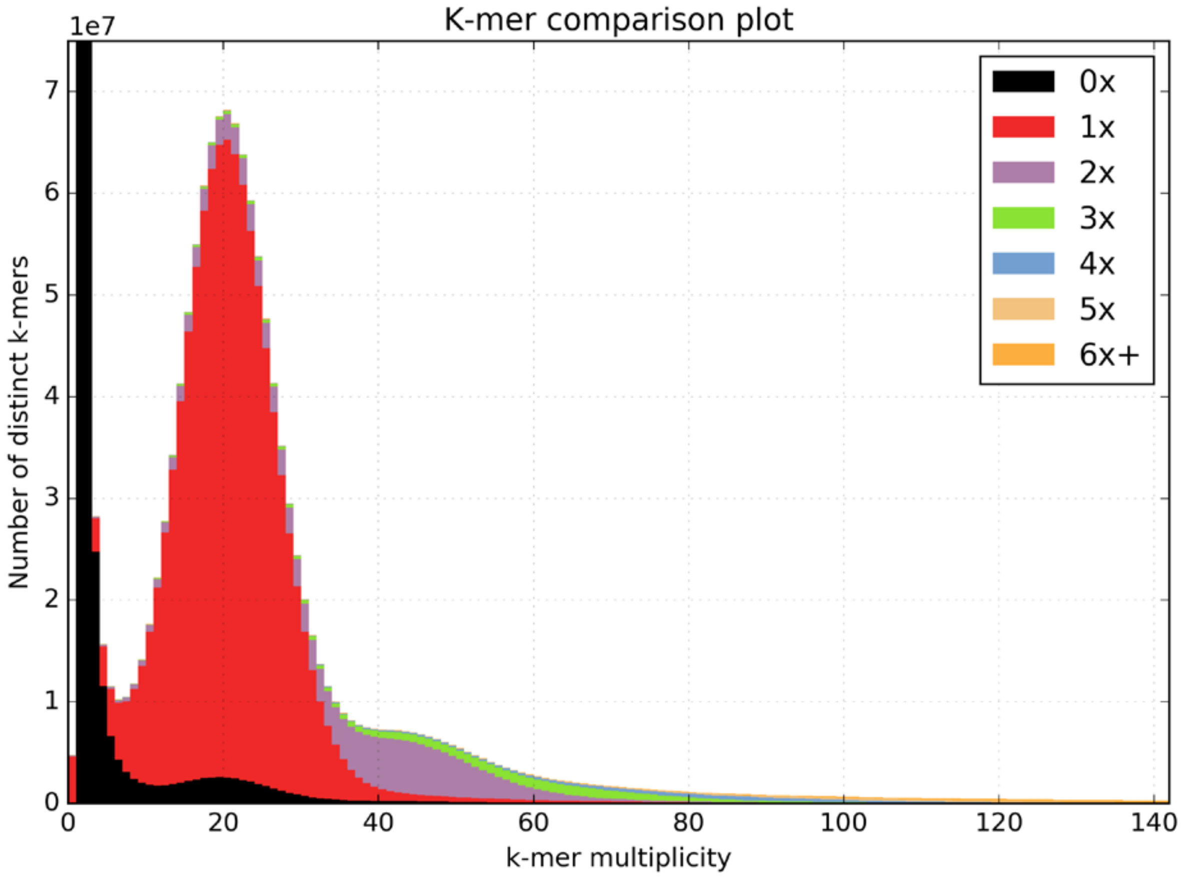

7.1) The kmer coverage at k=31 is around 20x using both PE libraries, which is lower that would be recommended to construct contigs (around 35x to 100x is probably best practice nowadays). I expect the coverage at the K used for assembly to be even lower.
Reply: Thank you for this technical suggestion. When we started this project, we followed previous genome sequencing projects and decided to sequence 30X depth for every shotgun library. However, after read trimming, the total depth was a bit lower than 30X. We also have noticed some new standards for genome assembly (Clavijo *et al.*, 2017) and have decided to follow these standards in future work.

7.2) There is a loss of single-copy content (represented by the black distribution's peak at x=20) and generation of some content in the assembly not present on the reads (x=0). While this can be explained because of the gap closing and some N inclusion in the assembly, it merits some discussion and validation.
Reply: Thank you for pointing this out. To increase the efficiency of gene prediction, we have filtered fragmental scaffolds <1000 bp long. We performed KAT analysis on scaffolds before and after filtering with the 500 bp library. No significant content loss on unfiltered scaffolds ensued. Thus, we believe that the loss of single-copy content was due to length filtering.

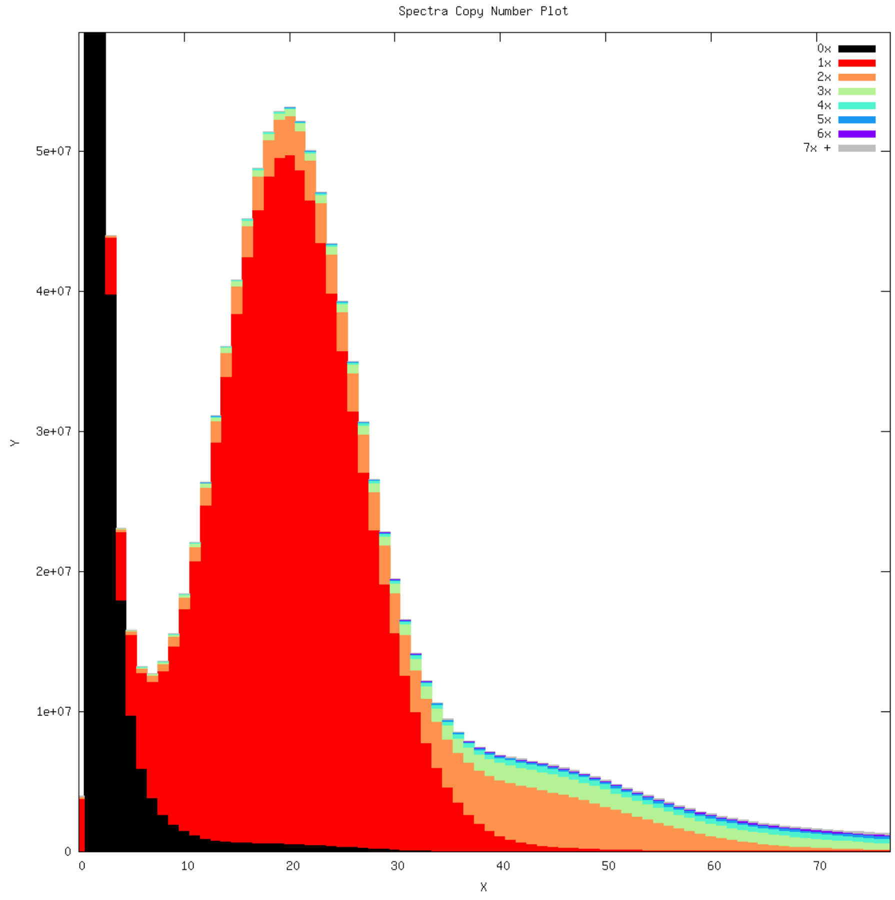

7.3) There is duplication on the main single-copy distribution at x=20. This could explain some of the content loss (again, due to gap closing? analyses before and after gap closing would make this clear). At the same time, SOAPdenovo2's scaffolding introduces a single N between contigs when a negative distance between them is estimated by the scaffolding algorithm. This produces both unnecessary duplication of sequence and some misassembles. There are more than 350K instances of single Ns on the assembly and soap does not output Ns on the contigs, so this should be examined.

Reply: Thank you for pointing this out. We have performed KAT analysis at 7.2, we do not believe that a single Ns led to content loss.

8) The scaffolding method is slightly unorthodox, consisting of contig assembly and scaffolding with SOAPdenovo (I assume SOAPdenovo2, but it is not properly cited and no version is mentioned), then gap

```
$ egrep -c '[ACTG]N[ACTG]' Ginseng_genome_assembly_v1.fasta

368679
```

closing, then a further round of scaffolding with SSPACE. In general there are better and more modern methods to do this same work. Running an ABySS assembly end-to-end and comparing the results may be a good starting point.

Reply: Thank you for the suggestion. Yes, we used SOAPdenovo2, and the reference (Luo *et al.*, 2012) has been added to R1. We also used ABySS 1.9 but had a large computer node with 1 TB memory only, and the ABySS assembly failed because of insufficient memory.

8.1) Re-scaffolding with the same datasets should not be needed, so I would suggest the authors either just use SOAP's contigs into SSPACE, or do not perform a second round of scaffolding. Performing gap closure before any scaffolding is also not recommended, so in any case this should be the last step in the assembly. As this assembly procedure does not follow best practices, I would have also expected to see a detailed description

of the results at every step (i.e. contiguity, N content, etc). Also, the detailed configuration files and commands executed to produce the assemblies should be provided on the supplementary material and referred to in the main text (this is valid for all other analyses).
Reply: Thank you for the suggestion. We have unclearly described our assembly process and have accordingly made corrections. First, we used SOAPdenovo2 to assemble contigs and scaffolds with 250 and 500 bp short-insert libraries and 2000 bp long-mate-pair library. We then used SSPACE for scaffolding with 2K bp, 5K bp, and 10K bp long-mate-pair libraries. Afterwards, we used Gapcloser for gap closing using short libraries and obtain the final scaffolds. We have validated genome assemblies with LMP data, recalculated the mate-pair distance, and speculated that no significant overscaffolding occurred. We have added SSPACE configuration files into the Supplementary Text.

8.2) Any scaffolding such as this needs some validation, at the very least by analysing synteny with related species, and or by producing wet-lab validation of some junctions.
Reply: Yes, thank you for the suggestion. We have validated the junction through alignment with published *P. ginseng* BAC sequences. We observed good collinearity between the draft genome and BAC sequences as shown in the following figure.

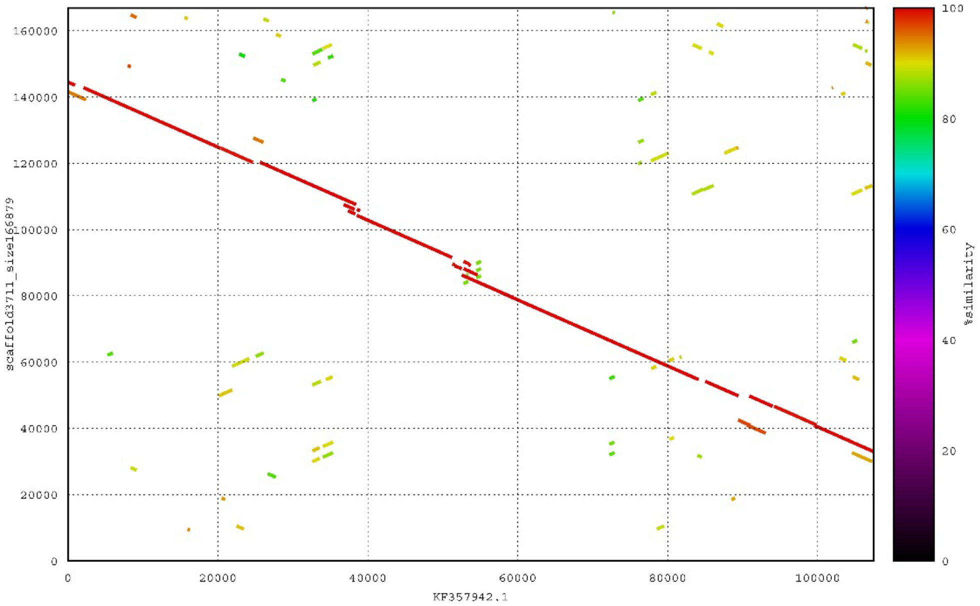

8.3) We ran library fragment size analysis in the LMP libraries by mapping them to the assembly and found significant short-insert (pair-end orientation) contamination. Fragment-size distributions also changed if the assembly was split at N runs. Peaks at exactly the "nominal" distances, specifically for the 10 Kbp library that was actually 7.5 Kbp, disappeared. This finding indicated some effect of overscaffolding. Performing this kind of analyses at every step of the assembly pipeline helped evaluate where biases were introduced.

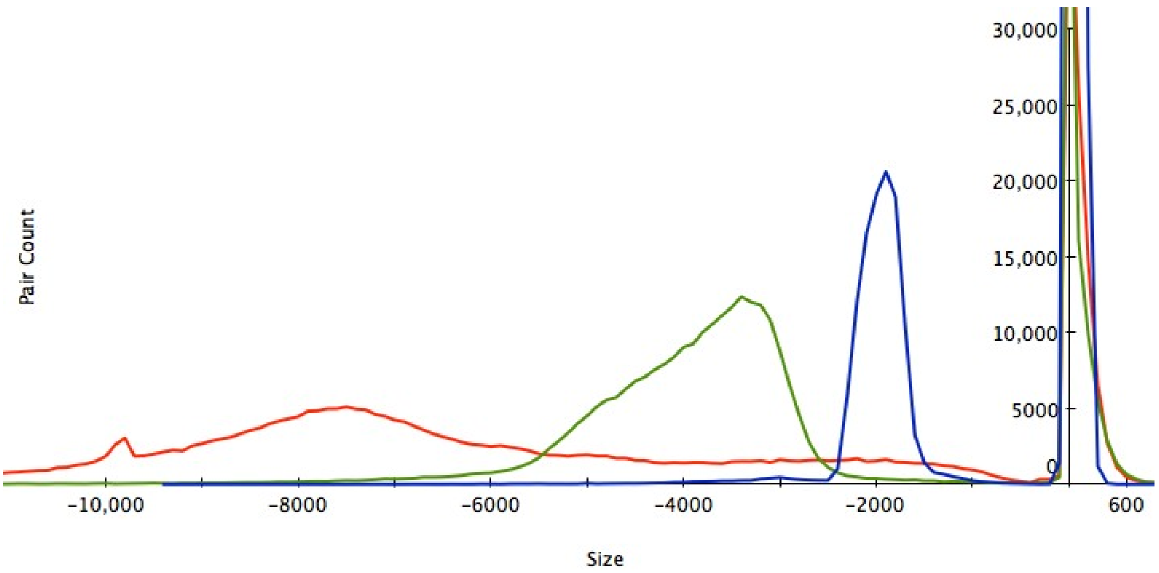

8.4) The 10Kbp library is in fact 7.5Kbp so all references to that size should be updated. If 10Kbp has been used as size in software that does not correct for it automatically it may need to be re-run.
Reply: Thank you for pointing this out. We have run a validation test on size distribution on contigs and scaffolds. We mapped each LMP library read to contigs and scaffolds. We obtained a similar size distribution of each library between contigs and scaffolds. We speculated that small-insert-size fragments were easier to cyclize when constructing an LMP library. Similar size-distribution results showed no significant overscaffolding in scaffolds.

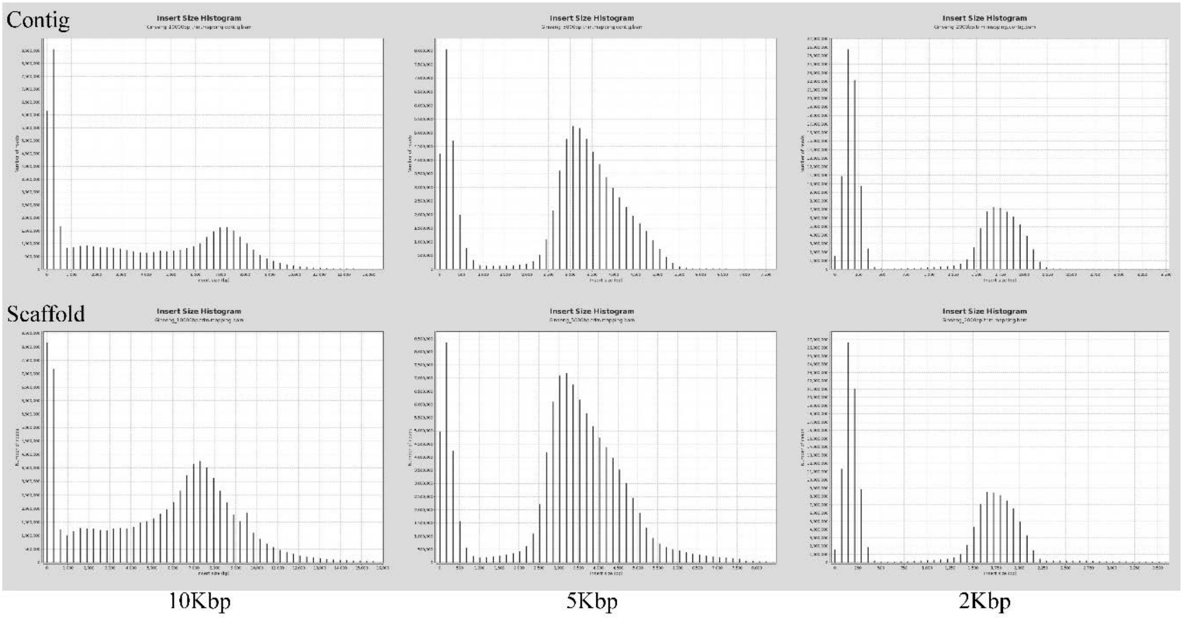

10Kbp 5Kbp 2Kbp

9) Transcript assembly mappability is shown as a metric of assembly reliability, but it is unclear that any *de novo* transcript assembly was performed. If that is the case, the *de novo* transcript assembly must be properly described and discussed.
Reply: We conducted RNA-seq assembly by using Trinity software with default parameters through one RNA-seq library and generated 75 878 transcripts (N50 length, 1446 bp; average sequence length, 926 bp), which were used for further gene annotation as RNA evidence. We have added these information to the manuscript.

10) The mapping of the 500bp library to assess non-bias is ad-hoc and I am not aware of methods that would

support this (it also introduces mappability bias, etc, etc). For completeness I would prefer either metrics based on how well the original reads map back to the assembly or simply kmer spectra completeness (Disclaimer: I am the senior author of the KAT publication).
Reply: We have mapped 250 and 500 bp libraries to the draft genome using BWA mem with default parameters, which had 99.77% and 99.95% reads mapped. We also performed KAT analysis, and the result was 7.2. We found no significant content loss for unfiltered scaffolds.

RNA-seq and differential expression

11) The whole method of the transcript assembly, mapping support and numbers does not add up easily. A diagram of the annotation pipeline would clarify this, with numbers of transcripts, support for them, etc. This is a KEY point if this manuscript wants to present a resource for the community.
Reply: Thank you for pointing out this deficiency. Transcripts were *de novo* assembled with Trinity. Specifically, raw reads generated by RNA-seq of different parts of ginseng root were trimmed and quality controlled by skewers with the following parameters: 10% of the maximum mismatch rate, trimming reads from the 3′ end until Q>20, and trimmed reads with read length <100 bp or average quality <30 were filtered out. Thereafter, Trinity software with default parameters were used for de novo assembly. A total of 75 878 transcripts (N50 length, 1446bp; average sequence length, 926 bp) were assembled and used for further gene annotation as RNA evidence. The clean RNA-seq reads were aligned to the draft genome in the orientation mode through TopHat, and gene expression levels were calculated using FPKM value.

12) As mentioned in (4) the usage of dataset from previous studies needs more justification and support.
Reply: As we stated in in (4), analyzing new and previous datasets together was defective. Thus, the transcription analysis of various organs was reformed with new datasets from a single study (Wang *et al.*, 2015). The transcription profile of three different tissues in root was analyzed in terms of the expression patterns of various HMGR types.

13) Figure 3e is unclear and probably also belongs in the supplementary.
Reply: Thank you for the suggestion. We have moved this figure to the supplementary.

14) Figure 4a shows "a possible pathway" but gives little extra detail or justification and it is not clear from the main text neither. I think this should be expanded and properly described in the main text.
Reply: Thank you for the suggestion. Fig. 4 shows that ginsenosides were mainly biosynthesized through the themevalonic acid (MVA) pathway utilizing the precursor IPP in the cytosol. This pathway is an ancestral metabolic route in all organisms, and eukaryotes have conserved enzymes from MVA to IPP synthesis. Given that the end product is IPP, this pathway is also accepted as the upstream pathway of triterpenoid saponins, most of which have been studied well. Thus, in the main text, we showed only our new discovery, i.e., that homologous enzymes were found in *P. ginseng* by BLAST and motif searching. If more detailed information is needed, Reference 52 (Kim *et al.*, 2015) would be helpful.

15) Figure 4b shows an extremely different expression levels between root and all the other tissues. While this is expected and could be supportive of other claims in the manuscript, this also shows the division of new vs. pre-existent datasets. I think differential expression between the new datasets on this manuscript should become the main focus of this figure and the relevant section. If RNA-seq data for the other tissues can be regenerated and/or shown to be perfectly comparable for this purpose, then I would suggest using differential expression patterns to test the robustness of the proposed pathway membership among the proposed genes, and even maybe trying to find "missing members" of the pathway.
Reply: Thank you for the suggestion. As explained in Q4, we replotted the heatmaps using previous datasets and new datasets independently.

16) Figure 5b uses bars and SD to show 3-point datasets. This hides the real data and leads to a feeling of over-confidence in what is effectively a small-n case. Replace with a plot showing every point. The mean can be indicated within this plots with a line, but SD is mostly meaningless with N=3.
Reply: We appreciate the reviewer’s advice. Showing small sample size data using a plot was appropriate. Thus, we have redrawn Fig. 5c, replacing the bars and SD with plots showing every point.

17) I can't finish to understand Figure 7, and I am conscious about the rna-seq dataset origin is playing a role here too.

Reply: We regret the unclear description in Fig. 7, especially Fig. 7b. We have added some information to Fig. 7 (V1)/Fig. 6 (R1) to make it clearer. The upper part of Fig. 6b (R1) is a sketch of a UGT73 cluster; the line shows the cluster length and the arrows represent gene length and direction. The bottom part is the expression level of these genes, and we wanted to show that although these genes were in the same gene cluster and belonged to the same gene family, their expression patterns differed. Further study of these genes may benefit the biosynthesis analysis of ginsenosides. Thank you!

Pathway analysis

18) The first paragraph of this section is difficult to read, rewrite for clarity, please.
Reply: The MVA pathway is a conserved pathway for sterols or terpenoids synthesis. We have shortened the description of this pathway, which may have decreased the clarity of this paragraph. Accordingly, we have added a couple of references (References 12, 14, and 17) to this part and to increase understanding.

19) Copy number assessment of genes based on a WGS fragmented assembly as this one should at the very least be validated by using kmer coverage and/or read mapping depth. Moreover since there is a mention to putting together some of the genes manually because they were fragmented on the original assembly.
Reply: We have extracted the alignment information (.bam file) of the scaffold that contained the UGT gene cluster from full alignment (mapping 500 bp library reads to draft genome using BWA mem). We then calculated the mapping depth of this scaffold. Except for the gap, repeat region, or high-GC-content area, the mapping depth was around 20X.

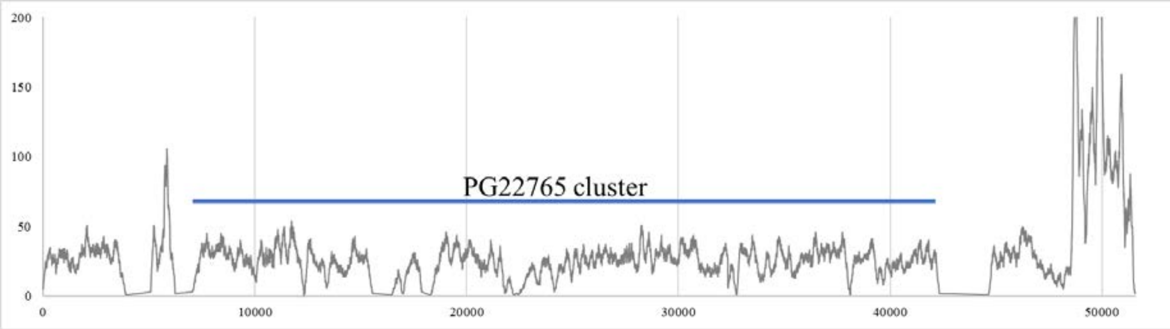

Microbial resistance

20) I am not very sure how this section relates to the rest of the manuscript.
Reply: Thank you for the suggestion. Although it is important for ginseng cultivation, this section was still slightly correlated with other parts of the manuscript. Considering the two reviewers' advice, the microbial-resistance section has been removed in R1. We are truly grateful for the reviewer's comments and suggestions.

21) The seven-time-points experiment is a whole analysis on its own, same need for experimental design explanation and rationale as in previous sections apply.
Reply: We apologize for the confusion. These data were from a previously published paper (Gao *et al.*, 2016) and contained limited explanation and rationale in V1.

22) The last sentence is too strong on its claim for the evidence presented and seems to have no experimental validation.
Reply: We apologize for including the statement without adequate evidence. This section has been accordingly deleted in R1.

Discussion

23) The claim for LTR content being significantly higher than previously thought is a huge one, and should be supported better starting by a proper statement of the methods used in both studies. *De novo* methods for LTR content estimation exist that can give a figure without the bias of the assembly. If this claim is well supported it should feature more prominently, as it will probably change the understanding of the genome's history and evolution. Also, further dating of LTRs and extra analyses could help elucidate why the results are

so different to previous reports.
Reply: Thank you for the professional suggestion. We think it is because of the major reason that we use the whole genome analysis rather than the BAC sequences. However, this comparison is a little unfairly as whole genome possess more information naturally. We are doing more analysis about ginseng repeats. We hope we can get more interesting information from our data.

Minor comments

1) Figure 1a should be a table, Figures 1b and 1c needs either more explanation over new findings or important confirmation or may just be moved to supplementary. I personally find it impossible to extract any information from figures like 1c.
Reply: Considering the limit in number of figures/tables and that Fig. 1a seems to be insufficiently vivid as an individual table, this figure was thus integrated into Fig. 1. We also believed that rebuilding the phylogentic tree can show the possible speciation history of different species and give feedback information for genome-assembly quality. Given that phylogenetic and Venn diagram analyses are conventional methods (Guan *et al.,* 2016; Wei *et al.,* 2016), we decided to retain keep these information in the original manuscript.

2) The abstract should be adjusted to get rid of unnecessary nitty-gritty details, and provide a more focused description of what the manuscript is aiming for (assembly+rnaseq or biosynthesis analysis, but tighter and more conclusive). Conclusions should not be along the lines of "this will help further work" but at the very least along the lines of "this supports this, this, and this types of analyses/developments".
Reply: Thank you for your suggestion. We have rewritten the Abstract and deleted some information.
Background: Ginseng, which contains ginsenosides characterized as bioactive compounds, has been regarded as an important traditional medicine for several millennia. However, the genetic background of ginseng remains poorly understood partly because of the plant's large and complex genome composition.
Results: We report the entire genome sequence of Panax ginseng using next-generation sequencing. The 3.5 Gb nucleotide sequence contained more than 60% repeats and encoded 42 006 genes. Twenty-two transcriptome datasets and mass spectrometry images of ginseng roots were adopted to precisely quantify the functional genes. Thirty-one genes were identified to be involved in the mevalonic acid pathway. Eight of these genes were annotated as 3-hydroxy-3-methylglutaryl-CoA reductases, which displayed diverse structures and expression characteristics. A total of 225 UDP-glycosyltransferase (UGTs) were identified, and these UGTs accounted for one of the largest gene families of ginseng. Tandem repeats contributed to the duplication and divergence of UGTs. Molecular modeling of UGTs in 71, 74, and 94 families revealed a regiospecific conserved motif located at the N-terminus. Molecular docking predicted that this motif captured ginsenoside precursors.
Conclusions: The panorama of ginseng genome is a valuable resource for understanding and improving the breeding, cultivation, and synthesis biology of this key herb.

3) The organisation of the text could be much clearer (i.e. have all the data about experimental design and sequencing for genome assembly in one place in the methods, the same for differential expression, etc).
Reply: Thank you for this suggestion, some adjustments have been made to increase text clarity in R1.

4) There is no justification on the plant organisms selected for comparison.
Reply: Thank you for pointing this out. Giseng is a traditional herb and has a long cultivation history in East Asia. We selected the plant organisms based on the physiological characteristics of *P. ginseng* during its cultivation. We also referred to some references regarding organism selection. We selected tissues based on the mass-spectrometry imaging results of ginseng root. Our future studies will include more detailed comparison works.

5) Figure 3b and 3c are plotted in such a way that some points are "hidden" behind other points.
Reply: Thank you for pointing this out. We have modified the Fig. 3b and 3c in R1 to show all sample points.

6) Figure 5 is the first point where "4-year-old" is mentioned. This belongs in methods and needs some explanation.
Reply: Thank you for pointing out this issue. We described the sample information in the method section in R1.

7) Figures 6b and 6c provide no information. Should be removed and/or a section (possibly in the

supplementary) describing their results and importance should be explicitly written.
Reply: Thank you for the suggestion. We have deleted them in R1 considering that the microbial-resistance section has been removed.

8) The sections for phylogenetic analysis and gene family identification are vague and/or add little information for the rest of the manuscript's analyses. They can either be moved to supplementary or their links to the rest of the manuscript stressed and the analyses strengthened.
Reply: Thank you for the kind suggestion. We have highlighted the reason why we chose to keep this part in minor comment 1. Furthermore, we have strengthened our findings by highlighting the importance of phylogenetic analysis and introducing some necessary citations in R1.

9) In general the discussion has some claims that do not seem completely supported from the current analyses, but I feel a plant biologist will be better suited to judge that.
Reply: Thank you for your comment. These suggestions are indeed very valuable to our work.

Best regards,
Bernardo J. Clavijo
NOTE: review produced with extensive help from Luis Yanes from my research team.
