# Supplementary material for: Panax ginseng genome examination for ginsenoside biosynthesis
Source: Gigascience. 2017 Oct 5;6(11):1–15. doi: 10.1093/gigascience/gix093 (PMC5710592; doi:10.1093/gigascience/gix093)
Supplement: GIGA-D-17-00036_Revision-2.pdf [file gix093_giga-d-17-00036_revision-2.pdf]

GigaScience

Ginseng genome examination for ginsenoside biosynthesis

--Manuscript Draft--

**Manuscript Number:**	GIGA-D-17-00036R2
**Full Title:**	Ginseng genome examination for ginsenoside biosynthesis
**Article Type:**	Research
**Funding Information:**	National Natural Science Foundation of China (81403053) — Dr. Jiang Xu<br>National Natural Science Foundation of China (81503469) — Dr. Shuiming Xiao<br>China Academy of Chinese Medical Sciences (ZZ0808021) — Prof. Shilin Chen<br>Guangdong Provincial Hospital of Chinese Medicine Special Fund (2015KT1817) — Prof. Zhihai Huang<br>China Academy of Chinese Medical Sciences Special Fund (ZZ0908067) — Prof. Shilin Chen<br>National Cancer Institute (US) (CA154295) — Prof. Yungchi Cheng
**Abstract:**	Background: Ginseng, which contains ginsenosides characterized as bioactive compounds, has been regarded as an important traditional medicine for several millennia. Howerver, the genetic background of ginseng remains poorly understood partly because of the plant's large and complex genome composition.<br>Results: We report the entire genome sequence of Panax ginseng using next-generation sequencing. The 3.5 Gb nucleotide sequence contained more than 60% repeats and encoded 42,006 predicted genes. Twenty-two transcriptome datasets and mass spectrometry images of ginseng roots were adopted to precisely quantify the functional genes. Thirty-one genes were identified to be involved in the mevalonic acid pathway. Eight of these genes were annotated as 3-hydroxy-3-methylglutaryl-CoA reductases, which displayed diverse structures and expression characteristics. A total of 225 UDP-glycosyltransferase (UGTs) were identified, and these UGTs accounted for one of the largest gene families of ginseng. Tandem repeats contributed to the duplication and divergence of UGTs. Molecular modeling of UGTs in the 71, 74, and 94 families revealed a regiospecific conserved motif located at the N-terminus. Molecular docking predicted that this motif captured ginsenoside precursors.<br>Conclusion: The panorama of ginseng genome represents a valuable resource for understaning and improving the breeding, cultivation, and synthesis biology of this key herb.
**Corresponding Author:**	Jiang Xu, PhD<br><br>CHINA
**Corresponding Author Secondary Information:**	
**Corresponding Author's Institution:**	
**Corresponding Author's Secondary Institution:**	
**First Author:**	Jiang Xu, PhD
**First Author Secondary Information:**	
**Order of Authors:**	Jiang Xu, PhD<br>Yang Chu, PhD<br>Shuiming Xiao, PhD

Powered by Editorial Manager® and ProduXion Manager® from Aries Systems Corporation

	Baosheng Liao, M.D.
	Qinggang Yin, PhD
	Rui Bai, M.D.
	He Su, PhD
	Linlin Dong, PhD
	Xiwen Li, PhD
	Jun Qian, PhD
	Jingjing Zhang, PhD
	Yujun Zhang, PhD
	Xiaoyan Zhang, M.D.
	Mingli Wu, M.D.
	Jie Zhang, M.D.
	Guozheng Li, PhD
	Lei Zhang, PhD
	Zhenzhan Chang, PhD
	Yuebin Zhang, PhD
	Zhengwei Jia, PhD
	Zhixiang Liu, PhD
	Daniel Afreh, PhD
	Ruth Nahurira, PhD
	Lianjuan Zhang, M.D.
	Ruiyang Cheng, M.D.
	Yingjie Zhu, PhD
	Guangwei Zhu, PhD
	Wei Rao, PhD
	Chao Zhou, PhD
	Lirui Qiao, PhD
	Zhihai Huang, PhD
	Yungchi Cheng, PhD
	Shilin Chen, PhD
**Order of Authors Secondary Information:**	
**Response to Reviewers:**	Dear Dr. Hans Zauner,<br><br>We appreciate you and the reviewers for your precious comments. We have carefully considered all comments for our last version and accordingly revised our manuscript. Please find below our point-by-point replies to the comments and detailed explanations of all changes ("R1" refers to the submitted revised version and "R2" is the new revised version; all revisions in R2 were tracked). All the changes were highlighted in the revised manuscript. Thank you!<br><br><br>Reviewer #1: The manuscript entitled: "Ginseng genome examination for ginsenoside biosynthesis" by Xu Jiang et al. was previously revised and some major considerations were made. I would like to thank the authors for answering point-by-point all my inquiries. I'm satisfied with the answers and don't have any further questions. I'm okay

with this version of the manuscript for its publication.

Thank you for reviewer #1's positive comments and thank you for permitting our work. We acknowledge the useful suggestion of reviewer #1. Thank you very much.

Reviewer #2: The manuscript has substantially improved, there is still some points that I would like to see considered (minor) and fixed (major) before publication:

Many thanks for reviewer #2's useful suggestion. Followings are our point-by-point replies, please check them.

Minor points partially addressed:
* The single-N issue with soap scaffolding still stands. It can be overlooked, although I would recommend it mentioned for transparency. In fairness I have failed to mention it on my own manuscripts on occasion out of a lack of knowledge about the issue, but it could help further understand assembly characteristics if needed. This can affect things like read mapping and gene annotation.
Thank you for pointing out this problem. We have added the information of single-Ns at the note of Table 1 Statistical analysis of the P. ginseng draft genome.
* The justification for the use of line IR826 needs to be written into the main manuscript, as soon as this line is mentioned.
Thank you for pointing out this problem. We have added the content in Page6, Line 16.
* The fact that some tissues come from another line needs to be written too and its possible implications for the analyses discussed (i.e. do the samples cluster by line?).
Thank you for pointing out this problem. We have added the cultivar name in Page 22 Line 1. We didn't find obvious difference among samples.
* I am not sure if the UTG analysis never included the extra expression datasets (in which case it is ok) or if it did why it is not changing.
We are sorry we didn't find any interesting information from the analysis of expression dataset in all UGTs, so we only put the expression analysis of a UGT73 gene cluster in this manuscript. We hope in future work we can get more useful information.
* The answer to my question about the ginsenosides' pathway should be included in the main text for clarity.
The pathway introduction was included in the introduction. We have highlighted in Page 4 Line 22.
* The copy number assessment justification could be included in the text for extra support (even if only in supplementary), for all relevant genes.
Thank you for this suggestion. We have added the justification at the supplementary(Supplementary Figure 10).
* Figure 1a should be a table. A table is a table, it sounds tautological but it is still true. Images do not allow automated data analysis by things like paper-crawlers and such.
Thank you for pointing out this problem. We agree with the reviewer's suggestion. We have split the table as Table 1.

Major points still standing:
* The library of nominal size 10Kbp is still referred as the "10Kbp" library in the manuscript without explanation of its actual 7.5Kbp fragment size mode. The effect of using this library as 10Kbp is noticeable both in the fragment size analysis the authors did and in the one I did. While this does not in my view invalidate the results of the assembly (most likely effect is to have some N runs of incorrect length here and there), the description of the library needs to be updated.
Thank you for pointing out this problem. We have added a column "Estimated insert size(bp)" at Supplementary Table S1. The estimated insert size was calculated using reads alignment.
* There is still no description of which lab protocol was used to produce LMP data. Mentions to transposase on the processing seem to indicate Nextera, we could not find content of nextera adaptors, so it would be good for reusability of this data to describe the protocol.
We are sorry for this negligence. Except the 2kb mate-paired library, all the libraries were constructed using the commercial library prep kits (Vazyme Biotech). The 2kb mate-paired library was constructed using 454 method(Cre/loxp recombination system), the linkage adapter was 5'-CGTAATAACTTCGTATAGCATACATTATACGAAGTTATACGA-3'. We have added the instruction in Page 20 Line 15. The check results of adapters were listed as

	following:<br><br>2kb<br><br>5kb<br><br><br>10kb<br><br>* The filtering of scaffolds <1000bp, which originates some sequence loss, is not mentioned in the manuscript. While this may be the right choice, it needs to be written and its effect of "possibly loosing some content" at the very least mentioned.<br>Thank you for mentioning this problem. We have noticed it in Page 21 Line 11, please check it.<br>* While the Figure in 5c was updated, the description was not. This could be expanded to show the main finding of this particular figure's analysis, which is unclear to me given that most of the patters are not clear now that the points are being represented.<br>We apologize for this negligence. The description in previous manuscript has been updated, but without labeling. Thereby, we just added the label of Fig 5c in the manuscript R2 at Page 14 Line 18. Thank you for your chariness suggestion.<br>* The estimation of LTR at 1.5x what was previously thought needs to be either put in context or presented with extreme care to highlight methodological differences.<br>Thank you for pointing out this problem. We have added the comments for the LTR change in Page 17 Line 5. We agreed with the reviewer that it should be highlighted here for the difference of BAC and WGS.
**Additional Information:**	
**Question**	**Response**
Are you submitting this manuscript to a special series or article collection?	No
**Experimental design and statistics**<br><br><br>Full details of the experimental design and statistical methods used should be given in the Methods section, as detailed in our Minimum Standards Reporting Checklist. Information essential to interpreting the data presented should be made available in the figure legends.<br><br><br>Have you included all the information requested in your manuscript?	Yes
**Resources**<br><br><br>A description of all resources used, including antibodies, cell lines, animals and software tools, with enough information to allow them to be uniquely identified, should be included in the Methods section. Authors are strongly encouraged to cite Research Resource Identifiers (RRIDs) for antibodies, model organisms and tools, where possible.<br><br><br>Have you included the information	Yes

requested as detailed in our Minimum Standards Reporting Checklist?	
**Availability of data and materials**<br><br>All datasets and code on which the conclusions of the paper rely must be either included in your submission or deposited in publicly available repositories (where available and ethically appropriate), referencing such data using a unique identifier in the references and in the “Availability of Data and Materials” section of your manuscript.<br><br>Have you have met the above requirement as detailed in our Minimum Standards Reporting Checklist?	Yes

Manuscript Click here to download Manuscript Manuscript R2.docx

Panax ginseng genome examination for ginsenoside biosynthesis

Xu Jiang[1, *], Chu Yang[1, *], Xiao Shuiming[1, *], Liao Baosheng[1, *], Yin Qinggang[1], Bai Rui[1], Su He[1, 2], Dong Linlin[1], Li Xiwen[1], Qian Jun[1], Zhang Jingjing[1], Zhang Yujun[1], Zhang Xiaoyan[1], Wu Mingli[1], Zhang Jie[1], Li Guozheng[3], Zhang Lei[4], Chang Zhenzhan[5], Zhang Yuebin[6], Jia Zhengwei[7], Liu Zhixiang[1], Daniel Afreh[8], Ruth Nahurira[8], Zhang Lianjuan[1], Cheng Ruiyang[1], Zhu Yingjie[1], Zhu Guangwei[1], Rao Wei[7], Zhou Chao[7], Qiao Lirui[7], Huang Zhihai[2], Cheng Yung-Chi[9,$], Chen Shilin[1,$]

[1]*Institute of Chinese Materia Medica, China Academy of Chinese Medical Sciences, Beijing 100700, China*

[2]*Guangdong Provincial Hospital of Chinese Medicine, Guangzhou 510006, China*

[3]*National Data Center of Traditional Chinese Medicine, China Academy of Chinese Medical Sciences, Beijing 100700, China*

[4]*Institute of Basic Research in Clinical Medicine, China Academy of Chinese Medical Sciences, Beijing 100700, China*

[5]*Department of Biophysics, School of Basic Medical Sciences, Peking University Health Science Center, Beijing 100191, China*

[6]*State Key Laboratory of Molecular Reaction Dynamics, Dalian Institute of Chemical Physics, Chinese Academy of Sciences, Dalian 116023, China*

[7]*Waters Corporation Shanghai Science & Technology Co Ltd, Shanghai 201206, China*

[8]*Institute of Crop Science, Chinese Academy of Agricultural Sciences/Key Laboratory of Crop Physiology and Ecology, Ministry of Agriculture, Beijing 100081, China*

[9]*Department of Pharmacology, School of Medicine, Yale University, New Haven, 06510, CT, USA*

* These four authors contributed equally to this work.

[$] Correspondence: Chen Shilin[a], Cheng Yungchi[b]

[a]E-mail: slchen@icmm.ac.cn

[b]E-mail: yccheng@yale.edu

Abstract

Background: Ginseng, which contains ginsenosides characterized as bioactive compounds, has been regarded as an important traditional medicine for several millennia. Howerver, the genetic background of ginseng remains poorly understood partly because of the plant's large and complex genome composition.

Results: We report the entire genome sequence of *Panax ginseng* using next-generation sequencing. The 3.5 Gb nucleotide sequence contained more than 60% repeats and encoded 42,006 predicted genes. Twenty-two transcriptome datasets and mass spectrometry images of ginseng roots were adopted to precisely quantify the functional genes. Thirty-one genes were identified to be involved in the mevalonic acid pathway. Eight of these genes were annotated as 3-hydroxy-3-methylglutaryl-CoA reductases, which displayed diverse structures and expression characteristics. A total of 225 UDP-glycosyltransferase (UGTs) were identified, and these UGTs accounted for one of the largest gene families of ginseng. Tandem repeats contributed to the duplication and divergence of UGTs. Molecular modeling of UGTs in the 71, 74, and 94 families revealed a regiospecific conserved motif located at the N-terminus. Molecular docking predicted that this motif captured ginsenoside precursors.

Conclusion: The panorama of ginseng genome represents a valuable resource for understaning and improving the breeding, cultivation, and synthesis biology of this key herb.

Key words: *Panax ginseng*; ginsenosides; genome; mass spectrometry imaging

Background

Panax ginseng C. A. Mey, a deciduous perennial plant belonging to the Araliaceae family, has been clinically used as a precious herbal medicine for several millennia in East Asia [1]. The name ginseng was translated from the pronunciation of the Chinese words “Ren shen” [2]. Modern pharmacological research confirmed that ginsenosides, the major bioactive compound of *P. ginseng*, exhibit multiple therapeutic activities. These activities include antitumor, antihypertensive, antivirus, and immune modulatory activities [3]. Therefore, *P. ginseng* is used as a general tonic or adatogen to promote longevity, particularly in China, Korea, and Japan [4].

Different ginseng tissues, such as the root and rhizome used in clinical practice, show significant differences in quality evaluation, commercial application, and clinical efficacy because of variations in ginsenosides [5]. Ginsenosides are frequently allocated and accumulated in specific tissues through transport systems for storage or defense. Chemical analysis, immunological staining, and microscopic imaging have all demonstrated that the ginseng cortex and periderm contain higher amounts of protopanaxadiol (PPD)-type ginsenoside (Rb1, Rb2, or Rc) and protopanaxatriol (PPT)-type ginsenoside (Rf) than those of the root medulla [6-8]. Histochemical staining also confirmed that ginsenosides are mainly located in the oil canals of the periderm and outer cortex regions of the root but not in the xylem nor pith [9, 10]. Considering their potential physiological role [11], the ginsenoside enrichment in the periderm is consistent with the plant’s biological function as phytoanticipin, which protects plants against pathogens.

Although the pharmacological importance of ginsenosides has been well established, their biosynthetic enzymes and regulatory mode remain unknown [12-17]. Ginsenosides are

biosynthesized through the cytosolic mevalonic acid (MVA) pathway, which is initiated by acetyl coenzyme A and ended with the terpene precursor isopentenyl diphosphate (IPP). After a series condensation reactions, a linear C30 molecule, that is, squalene, is generated [18] and converted into (S)-2,3-oxidosqualene [19] through cyclization [20]. Subsequently, after multiple oxidation events (e.g., mediated by cytochrome P450-dependent monooxygenases) [21-23], various types of ginsenoside precursors, including oleanolic acid and PPD/PPT, are formed. The precursors are then further decorated through glycosylation reactions [12, 13, 17].

The glycosylation reaction, namely the transfer of a sugar moiety to a specific acceptor, is performed by glycosyltransferases (GTs), a group of multigene superfamilies. The GTs that utilize uridine diphosphate (UDP) activated sugar molecules as donors are referred to as UDP-glycosyltransferases (UGTs). The diversity of the UGTs has been demonstrated by comparing genomic and complementary DNA (cDNA) sequences. In our previous work, 129 potential UGT sequences were predicted on the basis of annotation results from the transcriptome data of *P. ginseng* roots, stems, leaves, and flowers. Some of the sequences may encode enzymes responsible for ginsenoside backbone modification [24]. However, only a limited number of UGTs that glycosylate triterpenoid aglycones have been described in plants, such as *Medicago truncatula* [25], *Saponaria vaccaria* [26], *Barbarea vulgaris* [27], *Glycine max* [28], and *P. ginseng* [29-31]. Yan *et al.* [30] reported that the UGTPg1 from *P. ginseng* glycosylates the C20-OH of PPD and its derived ginsenosides in a regiospecific manner. Two recently identified UGTs from *P. ginseng* (PgUGT74AE2 and PgUGT94Q2) catalyze the glycosylation of the C3–OH of PPD to obtain Rh2 and elongate the glucose moiety of Rh2 to generate Rg3 [31]. Wei *et al.* [32] found that UGT1 and its homologous genes from *P. ginseng* can glycosylate PPT to

produce PPT-derived ginsenosides, which contain several key amino acids that determine their activities and substrate regiospecificities.

The functional genomic analysis of ginseng significantly progressed but still requires improvement. First, the analysis of gene and transcript expression has mainly focused on ginseng organs, but the ginsenoside content and types vary among different tissues within the same organ. Hence, the screening of potential key genes responsible for synthesizing and modifying ginsenosides by association analysis of transcriptome and chemical substances is not comprehensive. Second, gene duplication often leads to functional divergence. Even paralogous genes that execute the same function are usually regulated in different modes. In ginseng, the ubiquitous duplicated genes are difficult to fully illustrate using current datasets. Therefore, the analysis of the whole genome sequence and transcriptomes by the accurate location of ginsenosides may promote the precise mining of genes associated with ginsenoside synthesis. Herein, we present the genome sequence of *P. ginseng* and comprehensively characterize the genes responsible for ginsenoside biosynthesis and modification in the plant.

Data Description

Genomic DNA was extracted from the 4-year old *P. ginseng* line IR826, a high quality strain with low heterozygosity. This strain is cultivated by the Institute of Chinese Materia Medica in Jilin province. Five libraries with insert sizes ranging from 250 bp to 10 kb were constructed. Paired-end sequencing were performed using the HiSeq platform (Illumina) and 391.46 Gb raw data were produced (Supplementary Table S1). The raw reads were trimmed using skewer pipeline to remove low quality or duplicated reads. After trimming, 315.93 Gb data were used

for genome assembly. The final assembly was checked using Benchmarking Universal Single-Copy Orthologs (BUSCOs). The frozen transverse sections of the ginseng main root with 20 μm thickness were prepared using a microcryotome for DESI-MS imaging. The ginsenoside distribution was evaluated on a Xevo G2-XS Tof mass spectrometer with the DESI source. The image creation was performed using high-definition imaging (HDI) software (Waters Corporation) with the following parameters: X and Y pixel size 100 μm; raster speed 400 μm/s; spray solvent 90% MeOH, 10% H_2O, 0.1 mM NH_4Cl, and 0.1 mM leucine enkephalin delivered at 1.5 μl/min; MS at negative polarity, 4.5 kV capillary voltage, 80 V cone voltage, and mass range m/z 100-1,200. Total RNA were isolated from the periderm, cortex, and stele to construct RNA-seq libraries, each for triplicates. The RNA-Seq transcriptome libraries were prepared following the TruSeq™ RNA sample preparation kit (Illumina). After quantification, the paired-end libraries were sequenced by HiSeq 4000 (Illumina) (Supplementary Table S2). Except the nine RNA-seq data generated in this study, 13 published ginseng RNA-seq data were re-used. Further details about sample collection, DNA/RNA extraction, library construction, sequencing and mass spectrometry imaging can be found in the Methods section. All genome data have been uploaded to GigaDB [33] and sequencing reads can be found at NCBI (https://www.ncbi.nlm.nih.gov/) under the project number PRJNA385956.

Analyses

Characteristics of the *P. ginseng* genome

Genomic DNA was extracted from the 4-year old *P. ginseng* line IR826, a strain cultivated by the Institute of Chinese Materia Medica. This strain contains an estimated genome size of

3.5 Gb based on the k-mer prediction and flow cytometry analysis (Supplementary Figure S1; Supplementary Table S3). Approximately 112 X coverages of the raw sequence were generated using the Illumina HiSeq platform (Supplementary Table S1). After filtering, 91 X high-quality reads were adopted for assembly (Supplementary Table S1). The results provided a 3.43 Gb draft assembly with a contig N50 of 21.98 kb and a scaffold N50 of 108.71 kb (Table 1). Shotgun libraries with an insert size of 250 bp and 500 bp was mapped to the assembly, which have read mapping rate 99.77% and 99.95% respectively. The Poisson-like distribution of the sequence depth per base represents a nonbiased sequencing and assembly (Supplementary Figure S2). To confirm the accuracy, the 75,878 transcripts assembled from RNA-Seq data using Trinity [34] with default parameters were mapped back to the assembly with a mapping rate of 97.76%. Furthermore, Benchmarking Universal Single-Copy Orthologs (BUSCOs) [35] were used for quality assessment. A total of 1,323 (91.88%) CEG proteins, of which 24 BUSCOs were fragments, were determined in this assembly; 98.19% of the proteins were fully annotated, indicating the accuracy of the assembly.

More than 62% of the ginseng genome was predicted to be repeats; about 83.5% of the repeats were annotated as long terminal repeats (LTRs) (Supplementary Table S4 and S5). Ty3/Gypsy is the most abundant retro-element superfamily and accounts for 42.8% of the genome (Supplementary Table S6), which was higher than previously reported [36]. Moreover, the amount of Ty1/Copia comprised approximately 8.3% of the whole genome and exceeded previous predictions [36] (Supplementary Table S6). For the DNA transposon class, CMC was the most abundant repeat type and comprised 43 Mb of approximately 1.3% of the genome (Supplementary Table S6).

A total of 42,006 protein-coding gene models were predicted on the basis of *ab initio* and comparison methods using the MAKER pipeline. That is, 88% of these models were supported by the assembled RNA-Seq transcripts. More than 95.6% of the gene models contained homologs in the GenBank nonredundant database (E-value=1e−5). About 73.47% annotations could be assigned to Gene Ontology (GO) catalogs, and 68.39% could be assigned to Kyoto Encyclopedia of Genes and Genomes (KEGG) pathways (Supplementary Figure S3). Among these annotations, the following genes were obtained: 488 cytochrome P450 genes, including the PPD-ginsenosides synthase (PPDS) CYP716A47, PPT-ginsenosides synthase (PPTS) CYP716A53, and oleanolic acid synthase CYP716A52; 2,556 transcription factors; and 3,745 transporters (Supplementary Table S7 and S8).

Ortholog analysis of *P. ginseng* was conducted using 13 other plants (Supplementary Table S9). More than 75% of the gene models in *P. ginseng* were classified into 12,231 gene families, with 1,648 unique gene families for *P. ginseng* itself (Fig. 1a). The average gene number per gene family was 2.59, which was the highest among all 14 plants. This finding indicates the occurrence of duplication events during the evolution of *P. ginseng*. 383 single copy genes identified by ortholog analysis, we constructed a phylogenetic tree using the maximum-likelihood method. *Daucus carota* from Umbelliferae was found to be the closest relative of *P. ginseng* among all the compared species, diverging approximately 66 Myr ago (Fig. 1b), which is further supporting the relative evolutionary relationships between *Daucus carota* and *Panax ginseng* (http://www.uniprot.org/taxonomy/4054), and supporting the prevailing hypothesis of seed plants' phylogeny [37].

Metabolism and transcriptome of the ginseng root

Desorption electrospray ionization mass spectrometry (DESI-MS) imaging was used to elucidate the spatial distribution of ginsenosides within the ginseng root sections. Ginsenosides Rg1/Rf, pseudo Rc1, Ra1/Ra2, Rd/Re, Rs1/Rs2, and Ra3 were identified and summarized (Fig. 2b; Supplementary Table S10). Ginsenosides Rg1/Rf were highly concentrated within the outer bark and inner core areas of the root. Rd/Re Rs1/Rs2, Ra1/Ra2, and pseudoginsenoside Rc1 were distributed at high concentrations in the bark and at low concentrations in the center (Fig. 2c). Ginsenoside Ra3 exhibited a diffuse distribution within the cross section and a high concentration around the bark (Fig. 2c). These isomers were distinguished by DESI-tandem mass spectrometry (MS/MS). For Rf/Rg1, fragmentation of the monosaccharide group $C_6H_{10}O_5$ (162.05 Da) and disaccharide group $C_{12}H_{22}O_{11}$ (342.12 Da) produced fragments at *m/z* 637.46 and 457.15, which corresponded to different spatial distributions (Supplementary Figure S4). The characteristic MS/MS transitions were *m/z* 603.08 for Rd and *m/z* 799.52 for Re (Supplementary Figure S5). The enrichment of Rb1 around the bark was also confirmed through DESI-MS/MS (Supplementary Figure S6).

On the basis of anatomical characteristics, we categorized the ginseng main root into periderm, cortex, and stele for further quantitative analysis (Supplementary Figure S7). High-performance liquid chromatography (HPLC) results showed that the contents of ginsenosides Rg1, Re, Rf, Rg2, Rb1, Rc, Rb2, and Rd were significantly higher in the periderm ($P<0.001$) than in the cortex and stele (Fig. 3a; Supplementary Table S11). The PCA and PLS-DA plots

showed the distinct clustering among the periderm, cortex, and stele groups (Fig. 3b and c). The findings suggest the different distribution of ginsenosides.

More than 34,000 predicted genes were detected from the transcriptome data. Among these genes, 27,450 were expressed in the three sections, and 7,456 genes were not detected in any section. The samples were clustered into three distinct groups by expression profile. The expression pattern of genes in the cortex was closer to the stele than to the periderm (Fig. 3d). A total of 2,530, 2,688, and 711 differentially expressed genes were found between the periderm and cortex, the periderm and stele, and the cortex and stele, respectively. GO enrichment analysis showed that differential genes between the periderm and cortex, as well as the periderm and stele, were mainly associated with metabolic processes and response to stimuli (Supplementary Figure S8). The total number of genes were grouped into 64 modules through weighted gene coexpression network analysis (WGCNA). The total ginsenoside content was considered as the weighted factor, and three of the modules were positively correlated with ginsenosides. The most correlated module contained 15,762 genes, indicating the complex mechanisms involved in ginsenoside synthesis and regulation (Supplementary Figure S9).

Conserved biosynthesis pathway of ginsenosides

As triterpenoid saponins, ginsenosides are mainly biosynthesized using the precursor IPP produced through the MVA pathway, which includes conserved enzymes in eukaryotes [12, 14, 17]. In this study, 31 genes encoding 10 upstream enzymes were identified by BLAST search and motif finding (Fig. 4a). Except for acetyl-CoA C-acetyltransferase (AACT), all of these 10 enzymes displayed multiple copies and isoforms; 5 enzymes (8 in 3-hydroxy-3-methylglutaryl-

CoA reductase [HMGR], 4 each in squalene synthase [SS] and squalene epoxidase [SE], and 3 each in phosphomevalonate kinase [PMK] and 3-hydroxy-3-methylglutaryl-CoA synthase [HMGS]) had multiple copies and isoforms. One of the PMKs may be a potential pseudogene, with several termination codons dividing the coding regions. The four other enzymes (mevalonate kinase [MVK], mevalonate diphosphate decarboxylase [MVD], isopentenyl-diphosphate delta-isomerase [IDI], and farnesyl diphosphate synthase [FPS]) possessed two copies each. Such common occurrence of the multicopy phenomenon in ginseng MVA enzymes may be associated with the diverse regulatory control of triterpenoid or steroid biosynthesis in the plant. After the formation of two 3-oxidosqualenes, different ginsenoside precursors are cyclized and hydroxylated by various enzymes. In this assembly, five beta-amyrin synthases (beta-ASs), three oleanolic acid synthases (OASs), three dammarendiol synthases (DDSs), and three PPDSs, three PPTSs were identified. In addition, 100 terpenoid synthases were annotated, including one lanosterol synthase (LAS) and one cycloarstenol synthase (CAS) for ginseng sterol precursor cyclization.

The transcriptome of nine released RNA-Seq data (arm root, rhizome, stem, leaf blade, leaflet pedicel, leaflet peduncle, fruit pedicel, seed and fruit flesh) [38] were used for the expression analysis of ginsenoside biosynthesis upstream genes. Two organs from subterranean part were grouped into one clan. By contrast, the aerial parts, were grouped into another clan (Fig. 4b). The samples, fruit flesh and seed, were relatively privileged possibly because of their singleness as reproductive organs. Some genes were coexpressed in different organs. For example, PG07131 (HMGR), PG03840 (HMGR), PG11918 (SE), and PG28400 (PPTS) were particularly expressed in the fruit flesh sample but not in the other tissues. Meanwhile, PG19915

(OAS), PG16025 (SE), PG00849 (beta-AS), and PG37498 (HMGR) were coexpressed in the seed. In leaf blade, PG02251 (HMGR), PG38245 (HMGR), PG13769 (DDS), PG09257 (DDS), and PG03815 (CAS) were higher expressed. On the basis of hierarchical cluster analysis, the upstream genes were clustered into different groups with specific expression pattern. This pattern may be related to the organ-specific chemical distribution of ginseng (Fig. 4b).

Sequence analysis of the *P. ginseng* HMGR (PgHMGR) family

HMGRs catalyze the conversion of HMG-COA into MVA, which has been considered as the first committed step of ginsenoside synthesis. Eight HMGR-encoding genes were annotated. The full length of these genes were achieved by manual curation. Four of these genes showed high similarity to previously reported PgHMGR1 (with average similarity of 94.25%), and the other four genes showed similarity to PgHMGR2 (with average similarity of 93.26%) (Supplementary Table S12). Given the primary structure of putative peptide sequences, the eight PgHMGRs were further grouped into four subfamilies, namely, PgHMGR1.1 (PG16235, PG37498), PgHMGR1.2 (PG00233, PG15732), PgHMGR2.1 (PG03840, PG07131), and PgHMGR2.2 (PG38245, PG02251) (Fig. 5a). The PgHMGR1 family attained relatively shorter lengths, with 573 amino acids (aa) for HMGR1.1 and 565 aa for HMGR1.2. By contrast, the PgHMGR2 family revealed relatively long lengths, with 594 aa for HMGR2.1 and 589 aa for HMGR2.2 (Fig. 5b). Most of the PgHMGR-encoding genes (except PG15732) contain four exons and share the same exon phase pattern with the combination “0-2-1-0”. The PgHMGR2 family was 63 bp longer than PgHMGR1 in the first exon region, but both families were roughly the same in size as the three other exons. The introns among the PgHMGR-coding genes

fluctuated more than did the exons. Among the introns, the second intron varied the most, with a standard variation of 187 bp (Fig. 5d).

The deduced PgHMGRs were highly conserved at the C-terminal for MVA catalysis but were divergent at the N-terminal for membrane anchoring. Similar to most plants, all of the PgHMGRs contained a membrane anchor domain with a typical helix–loop–helix structure, a linker region for connection, two HMG-COA-binding motifs (MP(I/V)GY(I/V)QIP and TTEGCLVA), and two NADPH-binding motifs (DAMGMNM and GTVGGGT) (Fig. 5b). Therefore, the functional sites of all HMGRs were composed of similar residues, especially in the core region containing catalytic domains. Differences mainly located at the N-terminal were responsible for HMGR subcellular localization (Supplementary Table S13). All the deduced proteins, except HMGR1.2 (PG00233 and PG15732), attained a triple consecutive arginine region. This characteristic was implicated for endoplasmic reticulum retention. The expression patterns of different HMGR types differed among various organs (Fig. 4b and 5c). From the calculation of fragments per kilobase of exon model per million mapped reads (FPKM), the HMGR1 family expressed more stably with average FPKM CV of 81.97% and average extreme deviation of 1,107.81. Meanwhile, HMGR2 attained an average CV of 162.57% and average extreme deviation of 5,652.10, which was about 5 times higher than that of the HMGR1s (Supplementary Table S14). The HMGR2s were distinct among the tissues. Similar to PG07131 and PG03840, HMGR2.1 was highly expressed in fruit flesh and seedbut rarely in all other tissues (Supplementary Table S14). The excessive deviation of PG07131 reached 13,976.63, showing extreme tissue specificity (Supplementary Table S14). The two members of the HMGR2.2 family were prevalently expressed in leaf blades and highly expressed in the roots

(Supplementary Table S14). Analysis of the expression patterns of HMGRs indicated that they may perform different task assignments in ginseng development.

UGTs of *P. ginseng*

UGTs are in charge of transferring glycosyl moieties to acceptor molecules, including ginsenosides. The ginseng genome encodes a large, diverse set of UGTs. A total of 225 UGTs were identified, accounting for one of the largest gene families in ginseng. The length of these putative UGTs ranged from 74 aa to 575 aa. Moreover, the predicted isoelectric point ranged from 4.45 to 9.54. The identified UGTs were newly classified according to the standardization of the UGT Nomenclature Committee. As a result, all the UGTs were assigned to 24 subfamilies (Fig. 6a). UGT73 was the most abundant group (with 30 members), followed by UGT74 and UGT94 (with 25 and 24 members, respectively). Compared with *D. carota*, UGT74 and UGT71 notably expanded, whereas UGT93 largely shrank. Seventy-eight UGTs were found to be physically clustered into 30 groups, and the largest group contained five members. The PgUGTs were clustered similar to tandem repeats and generally belonged to the same subfamily. Similar to the largest cluster, all the members originated from an ancestral UGT73, with similarity ranging from 48% to 92%. The high similarity indicated that these genes may have evolved from recent genome duplications or newly unequal recombination events.

The expression module of UGTs also showed high tissue specificity. Similar to the mentioned gene cluster, the expression patterns of these UGTs considerably differed, although all of them originated from the same gene family (UGT73) (Fig. 6b, Supplementary Figure S10). PG22765-1 was the most highly expressed member with an average FKPM of 3,089 and was

the only highly expressed gene in the root, followed by PG22765-2 with an average FKPM of 1,957. Meanwhile, PG22765-5 was the most fluctuant gene, with a CV of 186.72. This UGT was rarely expressed in the organ root, stem, or leaf but highly expressed in the fruit. Hence, even UGTs that belong to the same family or located closely showed a distinctly regulated gene expression.

For functional analysis, 18 UGTs from families 71, 74, or 94 were chosen for molecular modeling and docking. The models of PPD and PPT were selected as docking substrates, and UGT-Glc was selected as sugar donor. The N-terminal I/V-G/S-H motif, the C-terminal W-N-S-X-L-E motif, and the C-terminal Y-G/A-E-Q motif of UGT71 family; the N-terminal motif Q-G-H-X-N/S and the C-terminal H-C/S-G-W-N-S-T-X-E motif of UGT74 family; and the N-terminal H/Q/Y-G-H motif and the C-terminal D-Q motif of UGT94 family were predicted to bind specifically to the sugar acceptors (Supplementary Figure S11). The results showed that the key residues in the N-terminal may have been subject to selection pressure during evolution for a particular substrate binding.

Discussion

Herbgenomics has been proposed as a global platform for securing the synthesis pathways of bioactive compounds [39-42]. This manuscript presented the genome of *P. ginseng*, which is the representative of herbs. The assembly confirmed the previous per-haploid-genome estimation of *P. ginseng* at approximately 3.5 Gb. Second only to *Ginkgo biloba*, ginseng harbors the largest genome among the sequenced medicinal plants [43]. Detailed structural analysis revealed that more than 62% of the genome consisted of repeats. This value is the

highest among those of all sequenced angiosperms, similar to orchid (61%) and higher than sorghum (58%), grape (49%), and rice (35%) [44-47]. LTR is a key factor in genome expansion. In *P. ginseng*, LTR accounted for 52% of the genome, which is 1.5-fold higher than a previous estimation using bacterial artificial chromosomes (BACs) [36]. As whole genome sequence possessed more information than BAC naturally, this change may be ascribed to the methodology differences. The result further emphasized the importance of whole-genome sequencing in the analysis of repeats and species evolution.

Compared with regular chromatography methods, DESI-MS enables the exploration of secondary metabolite distribution in tissues and even in cells. The resolution of DESI-MS typically reaches 100 μm or higher [48]. The spatial distribution images can show the continuous changes of ginsenosides in the ginseng root cross sections. These findings are expected to contribute to the screening of the physiological role, transport process, and accumulation of ginsenosides during ginseng growth and development, as well as in defense reactions, as responses to environmental challenges. DESI-MS can directly analyze isomeric compounds *in situ* [49]. Imaging ginsenosides by mass spectrometry confirmed the spatial maldistribution of ginsenosides. The data hence provided evidence for further gene expression analysis. Meanwhile, some ginsenosides accumulated in the root center, suggesting multiple sources of ginsenoside supply (Fig. 2). Schramek *et al.* found by ^{13}C-label tracing that the precursor units of ginsenosides are transferred from the leaves to the roots [50]. However, the mechanism underlying this long-distance transport and allocation remains unknown. Kim *et al.* speculated that ATP-binding cassette transporters or multidrug and toxic compound extrusion transporters may be involved in the transport process [51]. In the present research, more than

4000 transporters, including 331 ABC superfamily transporters and 71 MATE transporters, were identified. The obtained sequence information would facilitate future biochemical studies on ginsenoside transport.

The IPP for ginsenoside biosynthesis is generally produced via the MVA route. However, inhibition assays indicated that the methylerythritol phosphate (MEP) pathway compensated for IPP production when MVA was blocked. The MEP pathway is initiated by condensation between D-glyceraldehyde-3-phosphate and pyruvate by 1-deoxy-D-xylulose 5-phosphate synthase (DXP synthase, DXS). The pathway then terminates with the conversion of 4-hydroxy-3-methyl-butenyl 1-diphosphate (HMBPP) into IPP or dimethylallyl diphosphate (DMAPP) by isoprenoid synthase-containing protein H (IspH). In ginseng, the putative proteins involved in the MEP pathway were found to include 9 DXSs, 4 DXRs (DXP reductoisomerase), 2 IspDs, 4 IspEs, 5 IspFs, 4 IspGs, and 5 IspHs (Supplementary Figure S12). Similar to that in the MVA pathway, the members of the MEP route share a common multicopy phenomenon. Gene duplication was usually followed by functional divergence and metabolite diversity. As a result, certain ginsenosides or genes accumulated in different organs or tissues. Hitherto, this correlation has been largely unappreciated. Kim *et al.* cloned three SQSs based on ginseng expression sequences tags (ESTs) and reported their expression preferences [52]. Kim *et al.* found two copies of HMGR in ginseng and speculated that PgHMGR1 plays a general role in secondary metabolite production, whereas PgHMGR2 may be related to age-dependent ginsenoside accumulation in the root [53]. In the present study, more than two up to eight PgHMGRs were encoded by ginseng genomes. Of these PgHMGRs, four belong to the HMGR1 family and four belong to HMGR2 family. Each family can be grouped into two

subfamilies. The expression of PgHMGR2s was more fluctuant than that of PgHMGR1s among organs or tissues in ginseng. This result suggests that PgHMGR2 may conduct the regulation roles in terpene/phytosterol production during ginseng development. These results imply that the presence of multiple isoforms in the MVA/MEP route may contribute to flexible production or regulation of triterpene biosynthesis.

The glycosylation of triterpenes may increase their water solubility and modify their biological activities. In ginseng, UGTs are necessary for the ginsenoside biosynthesis by transferring monosaccharides to triterpene aglycones at C-3, C-6, or C-20 for the PPD- or PPT-type ginsenosides [51]. UGTs belong to a large and diverse gene family and can recognize a wide range of natural compounds as acceptor molecules. Triterpene glucosyltransferases belong to the UGT families 71, 73, 74, and 94 [25, 30]. These families are the most abundant UGT families in ginseng. Compared with other plants, triterpene glucosyltransferases were enriched in the ginseng genome during the evolution. This enrichment can partially account for the diversification of ginsenosides. Eighteen UGTs from UGT 71, 74, and 94 were selected for molecular modeling and docking. The results indicated that these UGTs were conserved in a three-dimensional structure and displayed a general regiospecificity but not tight substrate specificity. This finding can be proven by the report of Wei et al., stating that certain UGTs can modify both PPD- and PPT-type ginsenosides *in vitro* [32]. We have cloned and have prokaryotically expressed a putative UGT gene of ginseng with only one synonymous mutation to previously reported PgUGT94Q2 [31]. Normally, the functional assay of this gene is the same as reported; this gene can catalyze the conversion of ginsenoside Rh2 into ginsenoside

Rg3 and that of ginsenoside Rf2 to ginsenoside Rd (Supplementary Figure S13). Further biochemical experiments are required for other candidate tetracyclic triterpene UGTs.

This research showed the genome sequence of *P. ginseng*. The pathway for the synthesis of ginsenosides was described and examined. Multiple copies of the MVA pathway and the fully described UGTs demonstrated the importance of the whole genome sequencing, while the knowledge of the specific expression of the isoform of MVA enzymes and the expansion of particular members of UGTs expanded the understanding of the regulation of ginsenoside biosynthesis. This research will contribute to ginseng breeding, cultivation, and synthesis biology and provides an effective solution for plant functional genomic analysis with increased throughput, precision, and sensitivity [54].

Methods

Genome sequencing and assembly

Genomic DNA was extracted from the 4-year old *P. ginseng* line IR826, a strain cultivated by the Institute of Chinese Materia Medica. Five libraries with insert sizes ranging from 250 bp to 10 kb were constructed. Except the 2kb mate-paired library, all the libraries were constructed using the commercial library prep kits (Vazyme Biotech). The 2kb mate-paired library was constructed using Cre/loxP recombination system, the adapter was changed to 5'-CGTAATAACTTCGTATAGCATACATTATACGAAGTTATACGA-3'. We performed the paired-end sequencing on the HiSeq platform (Illumina) and produced 391.46 Gb raw data (Supplementary Table S1). The genome size was estimated through the flow cytometry (BD Biosciences) analysis and K-mer distribution. The reads were filtered using a skewer [55] with

the following criteria: trimming a 3′-end base to achieve quality >30 and exclusion of a short-insert library reads (250 and 500 bp) with a read length <100 bp or average quality <30; For large-insert library reads (2-10 kb), transposase adapter sequences were used for adapter searching and trimming, and max mismatch rate set to 10%, trimming reads from 3' end till Q>20, after trimming, reads with a read length <18 bp or average quality <30 were filtered out. Finally, 315.93 Gb reads were retained for genome assembly (Supplementary Table S1) through SOAPdenovo2 [56]. K-mer size selection was performed using KmerGenie [57] with 250bp-insert library and it recommended 83-mer, then k-mer size of 63, 73, 83, and 93 were used for assemble with default parameters, and the optimal k-mer size (k = 83) was selected based on the N50 length in each k-mer size. The reads from the small-insert libraries were used for contig construction to assemble the *P. ginseng* genome. The read pairs from the small- and large-insert libraries were then utilized to join the contigs into the scaffolds. Further scaffolding was performed using the large-insert libraries with SSPACE [58] (Configuration files in Supplementary Text). Finally, the small-insert libraries were used for gap closure of the scaffolds using GapCloser [59]. In order to annotate genes, scaffolds with length less than 1000bp were filtered out, however the filtering may lead to some content lost.

The two short-insert library reads were aligned onto the assembly through BWA mem with default parameters to evaluate the assembly quality [60]. We performed the BUSCO v2 analysis [35] with the recently released plant dataset from OrthoDB v9.1 [61] to test the completeness of the scaffolds. A total of 75,878 transcripts assembled from RNA-Seq dataset (assemble process was described at "Transcriptome sequencing and analysis" section) were mapped back

to the draft genome using BLAST [62] (BLASTN, an identity cutoff value of 90%, and a coverage cutoff value of 90%).

Ginsenoside distribution and content analysis

Main root of Damaya (a local cultivar of ginseng) were used for metabolome analysis and transcriptome analysis. The frozen transverse sections of the ginseng main root with 20 μm thickness were prepared using a microcryotome for DESI-MS imaging. The ginsenoside distribution was evaluated on a Xevo G2-XS Tof mass spectrometer with the DESI source (Waters Corporation). The MS images were created by spraying N_2 gas-focused solvent stream directly onto the sample to produce the MS spectra from the surface, which was then rastered across the sample at regular intervals to build a 2D image. The image creation was performed using high-definition imaging (HDI) software (Waters Corporation) with the following parameters: X and Y pixel size 100 μm; raster speed 400 μm/s; spray solvent 90% MeOH, 10% H_2O, 0.1 mM NH_4Cl, and 0.1 mM leucine enkephalin delivered at 1.5 μl/min; MS at negative polarity, 4.5 kV capillary voltage, 80 V cone voltage, and mass range *m/z* 100-1,200. The MS images were created from raw MS files through HDI with leucine enkephalin as the lockmass (*m/z* 554.2615) for high-resolution MS. The DESI-MS/MS images were created for ginsenoside Rf/Rg1 (*m/z* 799.48, −H adduct) and ginsenoside Rd/Re (*m/z* 945.54, −H adduct), and collision energy from 10-40 (arbitrary units).

The three independent ginseng root samples were divided into three portions: periderm, cortex, and stele, which were crushed and mixed with methanol containing 0.1% methanoic acid. The mixture was frozen for 1 h and then centrifuged. The upper layer was collected,

filtrated, and transferred to a sample vial to be injected and analyzed by HPLC for ginsenoside content measurement.

Transcriptome sequencing and analysis

The total RNA was extracted from the periderm, cortex, and stele using TRIzol® Reagent (Invitrogen) to construct a sequencing library. The RNA-Seq transcriptome libraries were prepared following the TruSeq™ RNA sample preparation kit (Illumina). mRNA was isolated with polyA selection by oligo(dT) beads and fragmented using a fragmentation buffer. Generally, cDNA synthesis, end repair, A-base addition, and the ligation of the Illumina-indexed adaptors were performed according to Illumina's protocol. The libraries were selected based on the size of the cDNA target fragments of 200–300 bp, followed by PCR amplification using Phusion DNA polymerase (New England Biolabs) for 15 PCR cycles. After quantification, the paired-end libraries were sequenced by HiSeq 4000 (Illumina).

Raw reads generated by RNA-seq of different parts of ginseng root were trimmed and quality controlled by Skewer with following parameter: adapter sequences searching and trimming with 10% max mismatch rate, trimming reads from 3' end till Q>20, trimmed reads with read length <100 bp or average quality <30 were filtered out. Thereafter, Trinity software with default parameters were applied for de novo assembly. The total length of 75,878 assembled transcripts is 70,273,566 bp, max, min and N50 length are 12,639 bp, 201bp and 1,446 bp respectively. The clean reads were separately aligned to the *P. ginseng* genome in the orientation mode through the TopHat software (http://tophat.cbcb.umd.edu/) [63]. For comparing the gene expression pattern among the different tissues of *P. ginseng*, six other tissue RNA-Seq datasets

from NCBI (accession number SRP066368) were analyzed [38]. The expression level for each transcript was calculated using the fragments per kilobase of exon per million mapped reads (FRKM) method to identify differentially expressed genes (DEGs) among the different samples. Cuffdiff (http://cufflinks.cbcb.umd.edu/) [64] was used for the differential expression analysis. The DEGs were selected using the following criteria: the logarithm of the fold change >2 and the false discovery rate (FDR) <0.05. The Gene Ontology (GO) functional enrichment and KEGG pathway analyses were performed through the Goatools (https://github.com/tanghaibao/Goatools) and KOBAS (http://kobas.cbi.pku.edu.cn/home.do), respectively, to understand the function of DEGs [65]. DEGs were significantly enriched in GO terms and metabolic pathways when their Bonferroni-corrected P-value is <0.05. The hierarchical clustering analysis of the expression profiles was performed using the hclust command in R and the default complete linkage method. The R package WGCNA [66] was used to identify the co-expression modules.

Repeat detection, gene prediction, and annotation

We detected the repeat content of the *P. ginseng* genome through an approach combining de novo prediction and homology-based searching. Three de novo prediction programs, namely, PILER-DF V1.0 [67], RepeatModeler V1.0.8 (http://www.repeatmasker.org), and LTR_FINDER V1.06 [68], were used to construct the de novo repeat library. The homology-based approach involves searching commonly used databases of known repetitive sequences. RepeatMasker V4.06 (http://www.repeatmasker.org) was used for the DNA-level identification with Repbase (a database of eukaryotic repetitive elements) using RepeatMasker V4.06

(http://www.repeatmasker.org), and RepeatProteinMask was utilized for protein level identification, which ran WuBlastX against the TE protein database. The tandem repeats in the genome assembly were identified through the tandem repeat finder.

The gene models of the *P. ginseng* genome were predicted using the MAKER-P pipeline [69]. The available ginseng EST, mRNA datasets, and protein datasets were used to generate the first-pass gene annotation. The resulting GFF3 file was used for *ab initio* gene predictor SNAP training [70]. The 75,878 transcripts assembled from the RNA-Seq data were used as transcript clues for the second-pass MAKER-P annotation. For further gene function annotation, the transcript encoding the longest protein sequence for each gene was defined as the representative sequence. First, each protein was searched against the NR [71], KOG [72], and Swiss-Prot [73] databases using BLASTx. The best similar hit with an E-value <1.0e−5 was considered the gene annotation information. Second, each protein was annotated according to the GO database [74], and Blast2GO was used to obtain GO terms representing a biological process, cellular component, and molecular function. Finally, all proteins were searched against the KEGG database [75] with the KAAS tool (http://www.genome.jp/tools/kaas/). Multiple plant organisms were selected to obtain the KEGG ortholog IDs of the best homologous genes.

Gene family identification and phylogenetic analysis

Thirteen other diploid plant genomes were used for cluster identification to determine the ortholog genes and to elucidate the evolution of the genome, in addition to the *P. ginseng* genome (Supplementary Table S9). The longest representative sequence of each genome under the pairwise sequence similarities among all input proteins was calculated using an all-by-all

BlastP with an E-value 1e−10, which was used to cluster the genes by OrthoMCL [76]. The peptide sequences from 383 single copy orthologous gene clusters were extracted to construct a phylogenetic tree and estimate the divergence time. After the multiple sequence alignment by MUSCLE [77] and the poorly aligned region removal by GBLOCKs [78], the high-quality blocks were converted (back-translation) in CDS and concatenated into one super-gene for each species. With these super-genes, a phylogenetic tree was constructed with RAxML through the PROTGAMMAJTT model [79].

The divergence time was estimated by MCMCtree program with 10,000 sampling times, 50 sampling rate, and 50,000 iteration burn-ins [80]. Two runs were performed to ensure convergence. The divergence time between monocots–dicots (140-150 Mya) or Arabidopsis–tomato or grape–tomato (110-124 Mya) was used to calibrate the divergence time [81-83]. Four species were selected for the lineage-specific evolutionary rate estimation with codeML through the free-ratio model. The genes with dS >3 or dN/dS >3 were filtered. Furthermore, the codeML with the branch-site model was used to estimate the branch-based ratio of nonsynonymous to synonymous substitution rate (ω or dN/dS). The branch-site model parameters were set as follows: null hypothesis: model = 2, NSsites = 2, fix_omega = 1, omega = 1; alternative hypothesis: model = 2, NSsites = 2, fix_omega = 0, omega = 1.

UGT family analysis, molecule modeling, and docking

Multiple alignments were performed using cluster X2 [84]. Phylogenetic trees were generated through MEGA 5.0 software [85]. The genetic distances were estimated using the pairwise distance amino acid substitution matrix with 100 bootstrap replicates.

The coordinates in pdb format of the small molecules protopanaxadiol and protopanaxatriol were built using the Corina (https://www.mn-am.com/online_demos/corina_demo_interactive). The homology models of the 18 UGTs from *P. ginseng* were built using the crystal structures as templates searched through the Swiss-model server http://swissmodel.expasy.org [86]. The docking of the protopanaxadiol or protopanaxatriol and the UDP-glucose in the constructed models was performed with Patchdock at http://bioinfo3D.cs.tau.ac.il/Patchdock [87, 88]. The ligand docking results were visualized with PyMOL molecular graphics system [89].

Funding

This work is supported by the grants from the National Natural Science Foundation of China (81403053, 81503469), the China Academy of Chinese Medical Sciences (ZZ0808021), the Guangdong Provincial Hospital of Chinese Medicine Special Fund (2015KT1817), the China Academy of Chinese Medical Sciences Special Fund for Health Service Development of Chinese Medicine (ZZ0908067), and National Cancer Institute, NIH, USA (CA154295).

Availability of supporting data and materials

The genome assemblies and annotation are available through our website at http://ginseng.vicp.io:23488/. The sequencing data of genome and transcriptome were deposited at GigaDB [33].

Author contributions

CSL and CYC initiated the study, designed the experiments, reviewed the data, and drafted the manuscript. CY, XSM, YQG, BR, ZJJ, ZXY, ZJ, JZW, LZX, ZLJ, CRY, ZGW and RW designed and performed the experiments. XJ, LBS, SH, QJ, WML, LGZ, ZL, ZhuYJ, ZC and QLR analyzed the data. XJ, XSM, CY, LBS, DLL, LXW, ZhangYJ, DA, RN and HZH wrote the manuscript.

Abbreviations

AACT Acetyl-CoA C-acetyltransferase

BUSCOs Benchmarking Universal Single-Copy Orthologs

CAS cycloartenol synthase

CDS Coding sequence

DDS dammarenediol synthase

DESI-MS Desorption Electrospray Ionization-Mass Spectrometry

DMAPP dimethylallyl diphosphate

DXR 1-deoxy-D-xylulose-5-phosphate reductoisomerase

DXS 1-deoxy-D-xylulose-5-phosphate synthas

EST expression sequences tags

FPP farnesyl diphosphate

FPS farnesyl diphosphate synthase

GT glycosyltransferase

HMBPP (E)-4-Hydroxy-3-methyl-but-2-enyl pyrophosphate

1 HMGCoA 3-hydroxy-3-methylglutaryl-CoA

2 HMGR 3-hydroxy-3-methylglutaryl-CoA reductase

3 HMGS 3-hydroxy-3-methylglutaryl- CoA synthase

4 HPLC High Performance Liquid Chromatography

5 IDI isopentenyl-diphosphate delta-isomerase

6 IPP isopentenyl diphosphate

7 IPP Isopentenyl diphosphate

8 LAS lanosterol synthase

9 LTR long terminal repeat

10 MEP 2-C-Methyl-D-erythritol 4-phosphate

11 MVA mevalonic acid

12 MVD mevalonate diphosphate decarboxylase

13 MVK mevalonate kinase

14 MVP mevalonate phosphate

15 MVPP diphosphomevalonate

16 Myr million years

17 OAS oleanolic acid synthase

18 PMK phosphomevalonate kinase

19 PPD protopanaxadiol

20 PPDS protopanaxadiol synthase

21 PPT protopanaxatriol

22 PPTS protopanaxatriol synthase

SE squalene epoxidase

SQS Squalene synthase

SS squalene synthase

UDP uridine diphosphate

UGT UDP-glycosyltransferase

WGCNA weighted gene coexpression network analysis

β-AS β-amyrin synthase

Competing financial interests

The authors declare no competing financial interests.

References

1. Hemmerly TE. A ginseng farm in Lawrence County, Tennessee. Econ Bot. 1977;31(2):160-2.

2. Leung KW. Pharmacology of Ginsenosides. In: Ramawat KG, Mérillon J-M, editors. Natural Products. Berlin Heidelberg: Springer; 2013. p. 3497-514.

3. Leung KW, Wong ST. Pharmacology of ginsenosides: a literature review. Chin Med. 2010;5(1):20.

4. Yun T-K. Brief introduction of *Panax ginseng* C. A. Meyer. J Korean Med Sci. 2001;16(Suppl):S3-5.

5. Zhang YC, Li G, Jiang C, Yang B, Yang HJ, Xu HY, et al. Tissue-specific distribution of ginsenosides in different aged ginseng and antioxidant activity of ginseng leaf. Molecules. 2014;19(11):17381-99.

6. Fukuda N, Shan S, Tanaka H, Shoyama Y. New staining methodology: eastern blotting for glycosides in the field of Kampo medicines. J Nat Med. 2005;60(1):21-7.

7. Taira S, Ikeda R, Yokota N, Osaka I, Sakamoto M, Kato M, et al. Mass spectrometric imaging of ginsenosides localization in *Panax ginseng* root. Am J Chin Med. 2010;38(3):485-93.

8. Yokota S, Onohara Y, Shoyama Y. Immunofluorescence and immunoelectron microscopic localization of medicinal substance, Rb1, in several plant parts of *Panax ginseng*. Curr Drug Disc Technol. 2011;8(1):51-9.

9. Christensen LP, Jensen M, Kidmose U. Simultaneous determination of ginsenosides and polyacetylenes in American ginseng root (*Panax quinquefolium* L.) by high-performance liquid chromatography. J Agric Food Chem. 2006;54(24):8995-9003.
10. Tani T, Kubo M, Katsuki T, Higashino M, Hayashi T, Arichi S. Histochemistry II. Ginsenosides in ginseng (*Panax ginseng*, Root). J Nat Prod. 1981;44(4):401-7.
11. Augustin JM, Kuzina V, Andersen SB, Bak S. Molecular activities, biosynthesis and evolution of triterpenoid saponins. Cheminform. 2011;72(28):435-57.
12. Haralampidis K, Trojanowska M, Osbourn AE. Biosynthesis of triterpenoid saponins in plants. Adv Biochem Eng Biotechnol. 2002;75(75):31-49.
13. Jenner H, Townsend BJ, Osbourn A. Unravelling triterpene glycoside synthesis in plants: phytochemistry and functional genomics join forces. Planta. 2005;220(4):503-6.
14. Liang Y, Zhao S. Progress in understanding of ginsenoside biosynthesis. Plant Biol. 2008;10(4):415-21.
15. Osbourn A, Goss RJM, Field RA. The saponins: polar isoprenoids with important and diverse biological activities. Nat Prod Rep. 2011;28(7):1261-8.
16. Sawai S, Saito K. Triterpenoid biosynthesis and engineering in plants. Front Plant Sci. 2011;2(25):25.
17. Thimmappa R, Geisler K, Louveau T, O'Maille P, Osbourn A. Triterpene biosynthesis in plants. Annu Rev Plant Biol. 2014;65(65):225-57.
18. Lee M-H, Jeong J-H, Seo J-W, Shin C-G, Kim Y-S, In J-G, et al. Enhanced triterpene and phytosterol biosynthesis in *Panax ginseng* overexpressing squalene synthase gene. Plant Cell Physiol. 2004;45(8):976-84.
19. Han J-Y, In J-G, Kwon Y-S, Choi YE. Regulation of ginsenoside and phytosterol biosynthesis by RNA interferences of squalene epoxidase gene in *Panax ginseng*. Phytochemistry. 2009;71(1):36-46.
20. Phillips DR, Rasbery JM, Bartel B, Matsuda SP. Biosynthetic diversity in plant triterpene cyclization. Curr Opin Plant Biol. 2006;9(3):305-14.
21. Han J-Y, Kim H-J, Kwon Y-S, Choi Y-E. The Cyt P450 enzyme CYP716A47 catalyzes the formation of protopanaxadiol from dammarenediol-II during ginsenoside biosynthesis in *Panax ginseng*. Plant Cell Physiol. 2011;52(12):2062-73.
22. Han JY, Hwang HS, Choi SW, Kim HJ, Choi YE. Cytochrome P450 CYP716A53v2 catalyzes the formation of protopanaxatriol from protopanaxadiol during ginsenoside biosynthesis in *Panax Ginseng*. Plant Cell Physiol. 2012;53(9):1535-45.
23. Han J-Y, Kim M-J, Ban Y-W, Hwang H-S, Choi Y-E. The involvement of β-amyrin 28-oxidase (CYP716A52v2) in oleanane-type ginsenoside biosynthesis in *Panax ginseng*. Plant Cell Physiol. 2013;54(12):2034-46.
24. Li C, Zhu Y, Xu G, Chao S, Luo H, Song J, et al. Transcriptome analysis reveals ginsenosides biosynthetic genes, microRNAs and simple sequence repeats in *Panax ginseng* C. A. Meyer. BMC Genomics. 2013;14(1):245.
25. Lahoucine Achnine, David V. Huhman, Mohamed A. Farag, Lloyd W. Sumner, Jack W. Blount, Dixon RA. Genomics-based selection and functional characterization of triterpene glycosyltransferases from the model legume *Medicago truncatula*. Plant J. 2005;41(6):875–87.

26. Meesapyodsuk D, Balsevich J, Reed DW, Covello PS. Saponin biosynthesis in *Saponaria vaccaria*. cDNAs encoding beta-amyrin synthase and a triterpene carboxylic acid glucosyltransferase. Plant Physiol. 2007;143(2):959-69.
27. Augustin JM, Drok S, Shinoda T, Sanmiya K, Nielsen JK, Khakimov B, et al. UDP-glycosyltransferases from the UGT73C subfamily in *Barbarea vulgaris* catalyze sapogenin 3-O-glucosylation in saponin-mediated insect resistance. Plant Physiol. 2012;160(4):1881-95.
28. Shibuya M, Nishimura K, Yasuyama N, Ebizuka Y. Identification and characterization of glycosyltransferases involved in the biosynthesis of soyasaponin I in *Glycine max*. FEBS Lett. 2010;584(11):2258-64.
29. Wang P, Wei Y, Fan Y, Liu Q, Wei W, Yang C, et al. Production of bioactive ginsenosides Rh2 and Rg3 by metabolically engineered yeasts. Metab Eng. 2015;29:97-105.
30. Yan X, Fan Y, Wei W, Wang P, Liu Q, Wei Y, et al. Production of bioactive ginsenoside compound K in metabolically engineered yeast. Cell Res. 2014;24(6):770-3.
31. Jung S-C, Kim W, Park SC, Jeong J, Park MK, Lim S, et al. Two ginseng UDP-glycosyltransferases synthesize ginsenoside Rg3 and Rd. Plant Cell Physiol. 2014;55(12):2177-88.
32. Wei W, Wang P, Wei Y, Liu Q, Yang C, Zhao G, et al. Characterization of *Panax ginseng* UDP-glycosyltransferases catalyzing protopanaxatriol and biosyntheses of bioactive ginsenosides F1 and Rh1 in metabolically engineered yeasts. Mol Plant. 2015;8(9):1412-24.
33. Xu J, Chu Y, Xiao S, Liao B, Yin Q, Bai R, et al. *De novo* sequencing of *Panax ginseng*. GigaScience Database. 2017.
34. Grabherr MG, Haas BJ, Yassour M, Levin JZ, Thompson DA, Amit I, et al. Full-length transcriptome assembly from RNA-Seq data without a reference genome. Nat Biotechnol. 2011;29(7):644-52.
35. Simao FA, Waterhouse RM, Ioannidis P, Kriventseva EV, Zdobnov EM. BUSCO: assessing genome assembly and annotation completeness with single-copy orthologs. Bioinformatics. 2015;31(19):3210-2.
36. Choi H-I, Waminal NE, Park HM, Kim N-H, Choi BS, Park M, et al. Major repeat components covering one-third of the ginseng (*Panax ginseng* C. A. Meyer) genome and evidence for allotetraploidy. Plant J. 2014;77(6):906–16.
37. Ruhfel BR, Gitzendanner MA, Soltis PS, Soltis DE, Burleigh JG. From algae to angiosperms–inferring the phylogeny of green plants (*Viridiplantae*) from 360 plastid genomes. BMC Evol Biol. 2014;14:23.
38. Wang K, Jiang S, Sun C, Lin Y, Rui Y, Yi W, et al. The spatial and temporal transcriptomic landscapes of ginseng, *Panax ginseng* C. A. Meyer. Sci Rep. 2015;5:18283.
39. Chen S, Song J, Sun C, Xu J, Zhu Y, Verpoorte R, et al. Herbal genomics: examining the biology of traditional medicines. Science. 2015;347(6219):S27-S9.
40. Chen S, Song J. Herbgenomics. China Journal of Chinese Materia Medica. 2016;41(21):3881-9.
41. Chen S, Xu J, Liu C, Zhu Y, Nelson DR, Zhou S, et al. Genome sequence of the model medicinal mushroom *Ganoderma lucidum*. Nat Commun. 2012;3(2):913.
42. Huang Z, Xu J, Xiao S, Liao B, Gao Y, Zhai C, et al. Comparative optical genome analysis of two pangolin species: *Manis pentadactyla* and *Manis javanica*. GigaScience. 2016;5(1):1-5.

43. Guan R, Zhao Y, Zhang H, Fan G, Liu X, Zhou W, et al. Draft genome of the living fossil *Ginkgo biloba*. GigaScience. 2016;5(1):49.

44. Cai J, Liu X, Vanneste K, Proost S, Tsai W-C, Liu K-W, et al. The genome sequence of the orchid *Phalaenopsis equestris*. Nat Genet. 2015;47(2):65.

45. Matsumoto T, Wu J, Kanamori H, Katayose Y, Fujisawa M, Namiki N, et al. The map-based sequence of the rice genome. Nature. 2005;436(7052):793-800.

46. Jaillon O, Aury J-M, Noel B, Policriti A, Clepet C, Casagrande A, et al. The grapevine genome sequence suggests ancestral hexaploidization in major angiosperm phyla. Nature. 2007;449(7161):463-7.

47. Paterson AH, Bowers JE, Bruggmann R, Dubchak I, Grimwood J, Gundlach H, et al. The *Sorghum bicolor* genome and the diversification of grasses. Nature. 2009;457(7229):551-6.

48. Mach J. Mass spectrometry imaging with single-cell resolution: spatial distribution of lipids in cotton seeds. Plant Cell. 2012;24(2):371.

49. Li B, Hansen SH, Janfelt C. Direct imaging of plant metabolites in leaves and petals by desorption electrospray ionization mass spectrometry. Int J Mass spectrom. 2013;348(2):15-22.

50. Schramek N, Huber C, Schmidt S, Dvorski S, Knispel N, Ostrozhenkova E, et al. Biosynthesis of ginsenosides in field-grown *Panax ginseng*. JSM Biotechnol Biomed Eng. 2014;2(1):1033.

51. Kim Y-J, Zhang D, Yang D-C. Biosynthesis and biotechnological production of ginsenosides. Biotechnol Adv. 2015;33(6):717-35.

52. Kim T-D, Han J-Y, Huh GH, Choi YE. Expression and functional characterization of three squalene synthase genes associated with saponin biosynthesis in *Panax ginseng*. Plant Cell Physiol. 2011;52(1):125-37.

53. Kim YJ, Lee OR, Oh JY, Jang MG, Yang DC. Functional analysis of 3-hydroxy-3-methylglutaryl coenzyme a reductase encoding genes in triterpene saponin-producing ginseng. Plant Physiol. 2014;165(1):373-87.

54. Yang W, Zhang Y, Wu W, Huang L, Guo D, Liu C. Approaches to establish Q-markers for the quality standards of traditional Chinese medicines. Acta Pharmaceutica Sinica B. 2017; http://dx.doi.org/10.1016/j.apsb.2017.04.012.

55. Jiang H, Lei R, Ding SW, Zhu S. Skewer: a fast and accurate adapter trimmer for next-generation sequencing paired-end reads. BMC Bioinformatics. 2014;15(1):182.

56. Luo R, Liu B, Xie Y, Li Z, Huang W, Yuan J, et al. SOAPdenovo2: an empirically improved memory-efficient short-read *de novo* assembler. GigaScience. 2012;1(1):18.

57. Chikhi R, Medvedev P. Informed and automated *k*-mer size selection for genome assembly. Bioinformatics. 2014;30(1):31-7.

58. Boetzer M, Henkel CV, Jansen HJ, Butler D, Pirovano W. Scaffolding pre-assembled contigs using SSPACE. Bioinformatics. 2011;27(4):578-9.

59. Li R, Zhu H, Ruan J, Qian W, Fang X, Shi Z, et al. *De novo* assembly of human genomes with massively parallel short read sequencing. Genome Res. 2010;20(2):265-72.

60. Li H, Durbin R. Fast and accurate short read alignment with Burrows-Wheeler transform. Bioinformatics. 2009;25(14):1754-60.

61. Zdobnov EM, Tegenfeldt F, Kuznetsov D, M.Waterhouse R, Simao FA, Ioannidis P, et al. OrthoDB v9.1: cataloging evolutionary and functional annotations for animal, fungal, plant, archaeal, bacterial and viral orthologs. Nucleic Acids Res. 2016;45(D1):D744-9.

62. Altschul SF, Gish W, Miller W, Myers EW, Lipman DJ. Basic local alignment search tool. J Mol Biol. 1990;215(3):403-10.

63. Langmead B, Salzberg SL. Fast gapped-read alignment with Bowtie 2. Nat Methods. 2012;9(4):357-9.

64. Trapnell C, Hendrickson DG, Sauvageau M, Goff L, Rinn JL, Pachter L. Differential analysis of gene regulation at transcript resolution with RNA-seq. Nat Biotechnol. 2013;31(1):46-53.

65. Xie C, Mao X, Huang J, Ding Y, Wu J, Dong S, et al. KOBAS 2.0: a web server for annotation and identification of enriched pathways and diseases. Nucleic Acids Res. 2011;39(suppl 2):W316-22.

66. Langfelder P, Horvath S. WGCNA: an R package for weighted correlation network analysis. BMC Bioinformatics. 2008;9:559.

67. Edgar RC, Myers EW. PILER: identification and classification of genomic repeats. Bioinformatics. 2005;21(suppl 1):i152-8.

68. Xu Z, Wang H. LTR_FINDER: an efficient tool for the prediction of full-length LTR retrotransposons. Nucleic Acids Res. 2007;35(suppl 2):W265-8.

69. Campbell MS, Law M, Holt C, Stein JC, Moghe GD, Hufnagel DE, et al. MAKER-P: a tool kit for the rapid creation, management, and quality control of plant genome annotations. Plant Physiol. 2014;164(2):513-24.

70. Johnson AD, Handsaker RE, Pulit SL, Nizzari MM, O'Donnell CJ, Bakker PIWd. SNAP: a web-based tool for identification and annotation of proxy SNPs using HapMap. Bioinformatics. 2008;24(24):2938-9.

71. Yangyang D, Jianqi L, Songfeng W, Yunping Z, Yaowen C, Fuchu H. Integrated nr database in protein annotation system and its localization. Computer Engineering. 2006;32(5):71-2.

72. Koonin EV, Fedorova ND, Jackson JD, Jacobs AR, Krylov DM, Makarova KS, et al. A comprehensive evolutionary classification of proteins encoded in complete eukaryotic genomes. Genome Biol. 2004;5(2):R7.

73. Apweiler R, Bairoch A, Wu CH, Barker WC, Boeckmann B, Ferro S, et al. UniProt: the Universal Protein knowledgebase. Nucleic Acids Res. 2004;32(suppl 1):D115-9.

74. Ashburner M, Ball CA, Blake JA, Botstein D, Butler H, Cherry JM, et al. Gene ontology: tool for the unification of biology. Nat Genet. 2000;25(1):25-9.

75. Kanehisa M, Goto S, Kawashima S, Okuno Y, Hattori M. The KEGG resource for deciphering the genome. Nucleic Acids Res. 2003;32(suppl 1):D277-80.

76. Li L, Stoeckert CJ, Roos DS. OrthoMCL: identification of ortholog groups for eukaryotic genomes. Genome Res. 2003;13(9):2178-89.

77. Edgar RC. MUSCLE: multiple sequence alignment with high accuracy and high throughput. Nucleic Acids Res. 2004;32(5):1792-7.

78. Talavera G, Castresana J. Improvement of phylogenies after removing divergent and ambiguously aligned blocks from protein sequence alignments. Syst Biol. 2007;56(4):564-77.

79. Stamatakis A. RAxML Version 8: a tool for phylogenetic analysis and post-analysis of large phylogenies. Bioinformatics. 2014;30(9):1312-3.
80. Yang Z. PAML: a program package for phylogenetic analysis by maximum likelihood. Bioinformatics. 1997;13(5):555-6.
81. Huang C-H, Sun R, Hu Y, Zeng L, Zhang N, Cai L, et al. Resolution of Brassicaceae phylogeny using nuclear genes uncovers nested radiations and supports convergent morphological evolution. Mol Biol Evol. 2016;33(2):394-412.
82. Massoni J, Couvreur TL, Sauquet H. Five major shifts of diversification through the long evolutionary history of Magnoliidae (angiosperms). BMC Evol Biol. 2015;15:49.
83. Barreda VD, Palazzesi L, Tellería MC, Olivero EB, Raine JI, Forest F. Early evolution of the angiosperm clade Asteraceae in the Cretaceous of Antarctica. Proc Natl Acad Sci U S A. 2015;112(35):10989-94.
84. Jeanmougin F, Thompson JD, Gouy M, Higgins DG, Gibson TJ. Multiple sequence alignment with Clustal X. Trends Biochem Sci. 1998;23(10):403-5.
85. Tamura K, Dudley J, Nei M, Kumar S. MEGA4: molecular evolutionary genetics analysis (MEGA) software version 4.0. Mol Biol Evol. 2007;24(8):1596-9.
86. Schwede T, Kopp J, Guex N, Peitsch MC. SWISS-MODEL: an automated protein homology-modeling server. Nucleic Acids Res. 2003;31(13):3381-5.
87. Duhovny D, Nussinov R, Wolfson HJ. Efficient unbound docking of rigid molecules. Lect Notes Comput Sci. 2002;2452:185-200.
88. Schneidman-Duhovny D, Inbar Y, Nussinov R, Wolfson HJ. PatchDock and SymmDock: servers for rigid and symmetric docking. Nucleic Acids Res. 2005;33(suppl 2):W363-7.
89. Seeliger D, Groot BLd. Ligand docking and binding site analysis with PyMOL and Autodock/Vina. J Comput Aided Mol Des. 2010;24(5):417-22.

Table legends

Table 1 Statistical analysis of the P. ginseng draft genome.

Figure legends

Fig. 1 *P. ginseng* genome assembly and functional gene annotations. a Phylogenetic tree and divergence data of 14 species, including *P. ginseng*, based on the proteins of 383 single-copy genes annotated to the genome sequence of each species. **b** Distribution of orthologous gene families in *P. ginseng* and four sequenced species: carrot (*Daucus carota*), coffee (*Coffea canephora*), *Arabidopsis* (*Arabidopsis thaliana*), and tomato (*Solanum lycopersicum*).

Fig. 2 Ginsenoside distribution in the *P. ginseng* root cross sections that obtained through mass spectrometric imaging based on the desorption electrospray ionization-mass spectrometry (DESI–MS). a Optical image of the main root. **b** TMS image spectrum. **c** DESI–MS image of metabolites and ginsenosides: maltose, citbismine C, Rg1/Rf, pseudo-Rc1, Ra1/Ra2, Rd/Re, Rs1/Rs2, and Ra3. Scale bar=2 mm.

Fig. 3 Metabolism and transcriptome analysis of *P. ginseng* root. a HPLC chromatograms of the ginsenosides Rg1, Re, Rf, Rg2, Rb1, Rc, Rb2, and Rd standards. **b** PCA score plots based on the HPLC dataset (●periderm, ●cortex, and ●stele). **c** PLS-DA score plots based on the HPLC dataset. **d** Cluster tree of the ginseng samples based on the expression pattern of 42006 genes. The leaves of the tree correspond to the different ginseng tissue samples (periderm, Per; cortex, Cor; stele, Ste). The color bands beneath the tree represent the relative content of the total ginsenosides, Rb1 and Rg1 (red indicates high values).

Fig. 4 Gene expression in the MVA pathway for ginsenosides in *P. ginseng*. a Possible biosynthesis pathway for ginsenosides with the designated candidate genes. AACT, acetyl-CoA C-acetyltransferase; HMGS, 3-hydroxy-3-methylglutaryl-CoA synthase; HMGCoA, 3-hydroxy-3-methylglutaryl-CoA; HMGR, 3-hydroxy-3-methylglutaryl-CoA reductase; MVK, mevalonate kinase; MVP, mevalonate phosphate; PMK, phosphomevalonate kinase; MVPP, diphosphomevalonate; MVD, mevalonate diphosphate decarboxylase; IPP, isopentenyl diphosphate; DMAPP, dimethylallyl diphosphate; IDI, isopentenyl-diphosphate delta-

isomerase; FPS, farnesyl diphosphate synthase; FPP, farnesyl diphosphate; SS, squalene synthase; SE, squalene epoxidase; β-AS, β-amyrin synthase; DDS, dammarenediol synthase; LAS, lanosterol synthase; CAS, cycloartenol synthase; OAS, oleanolic acid synthase; PPDS, protopanaxadiol synthase; PPTS, protopanaxatriol synthase. **b** Heatmap of the candidate biosynthesis pathway gene expression patterns in nine organs from *P. ginseng*.

Fig. 5 Sequence analysis and transcript levels of the HMGR gene family. a Phylogenetic analysis of PgHMGRs and characterized HMGRs from other plants. **b** Multiple alignments of the amino acid sequences of PgHMGRs with homologous HMGRs from *Arabidopsis*. The black boxes indicate identical residues; the gray boxes represent identical residues for at least two of the sequences. Functional domains are highlighted in colored boxes (red, membrane domain; green, linker domain; and blue, catalytic domain). The two putative HMGR-CoA-binding sites, two NADP(H)-binding sites, and ER retention motifs are denoted by square boxes. **c** Tissue-specific PgHMGR expression patterns in 4-year-old roots. The data represent the mean ± SD of the three independent samples. **d** Genomic DNA structure of PgHMGRs. The exons are represented by the green-filled square boxes. The lines between the boxes correspond to the introns. The numbers above the exons indicate the length in bp.

Fig. 6 Analysis of UGTs from *P. ginseng*. a All the identified UGTs which newly classified according to the standardization of the UGT Nomenclature Committee were assigned to 24 subfamilies. **b** The expression (lower) of UGT gene copies (PG22765) from the same scaffold (upper) in the different tissues of *P. ginseng*.

Table Click here to download Table Table.docx

Table 1 Statistical analysis of the P. ginseng draft genome.

	Size(bp)	Number
**Contig**		
N90	4,516	150,620
N80	8,639	103,388
N70	12,833	75,040
N50	21,977	39,481
Longest	574,183	-
Total size	2,999,700,459	337,439
**Scaffold**		
N90	24,143	33,423
N80	45,718	23,391
N70	65,171	17,168
N50	108,708	9,072
Longest	1,303,414	-
Total size	3,414,349,854	83,074
Gap ratio	12.15%*	-

* Among these gaps, 368,679 gaps are single-N.

Figure 1 Click here to download Figure Figure 1.tif

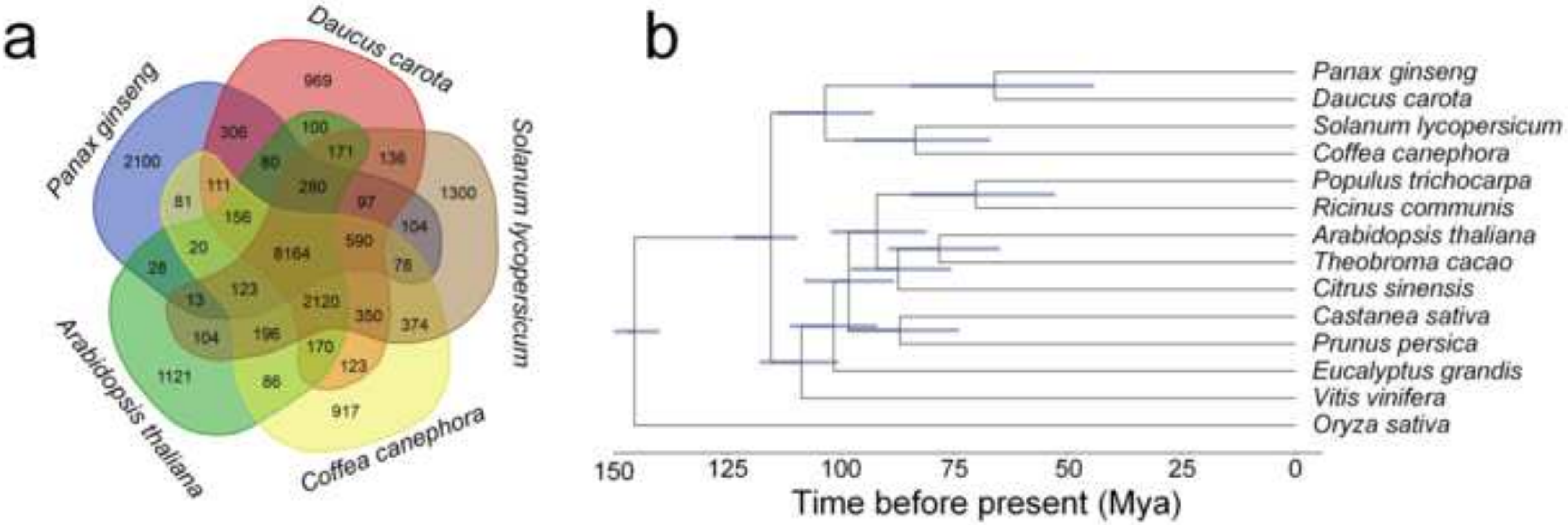

Figure 2

Click here to download Figure Figure 2.tif

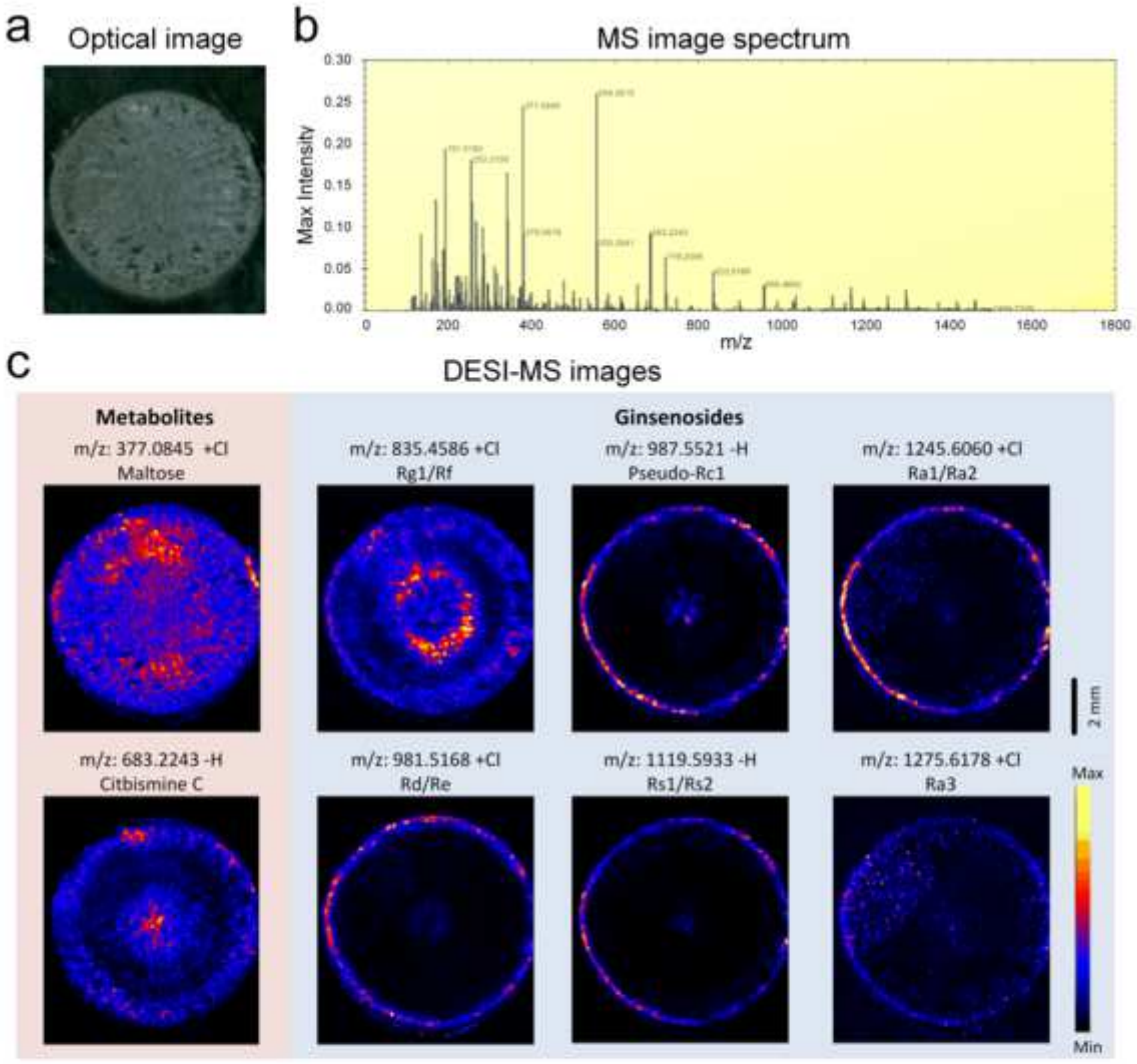

Figure 3

Click here to download Figure Figure 3.tif

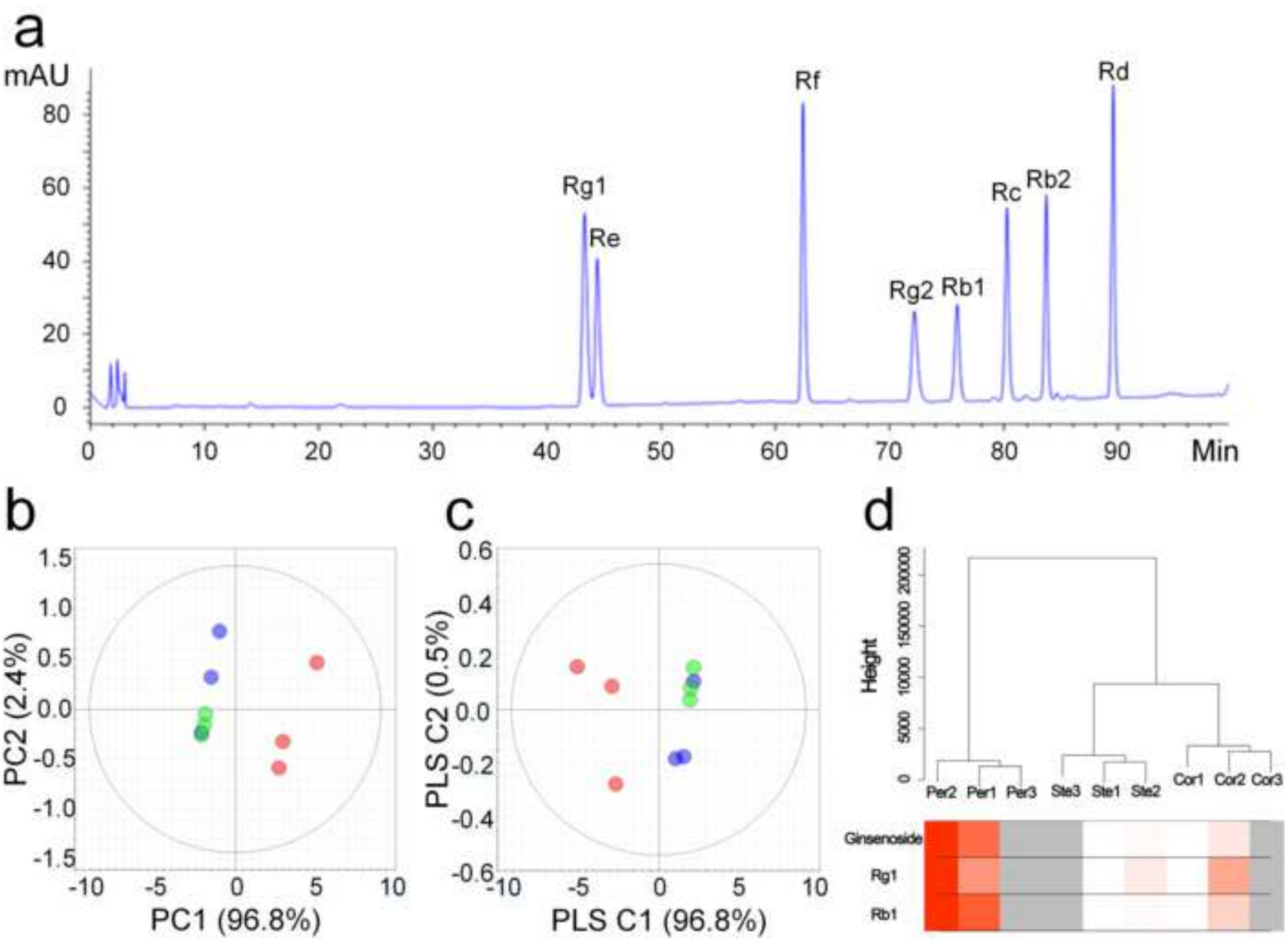

Figure 4 Click here to download Figure Figure 4.tif

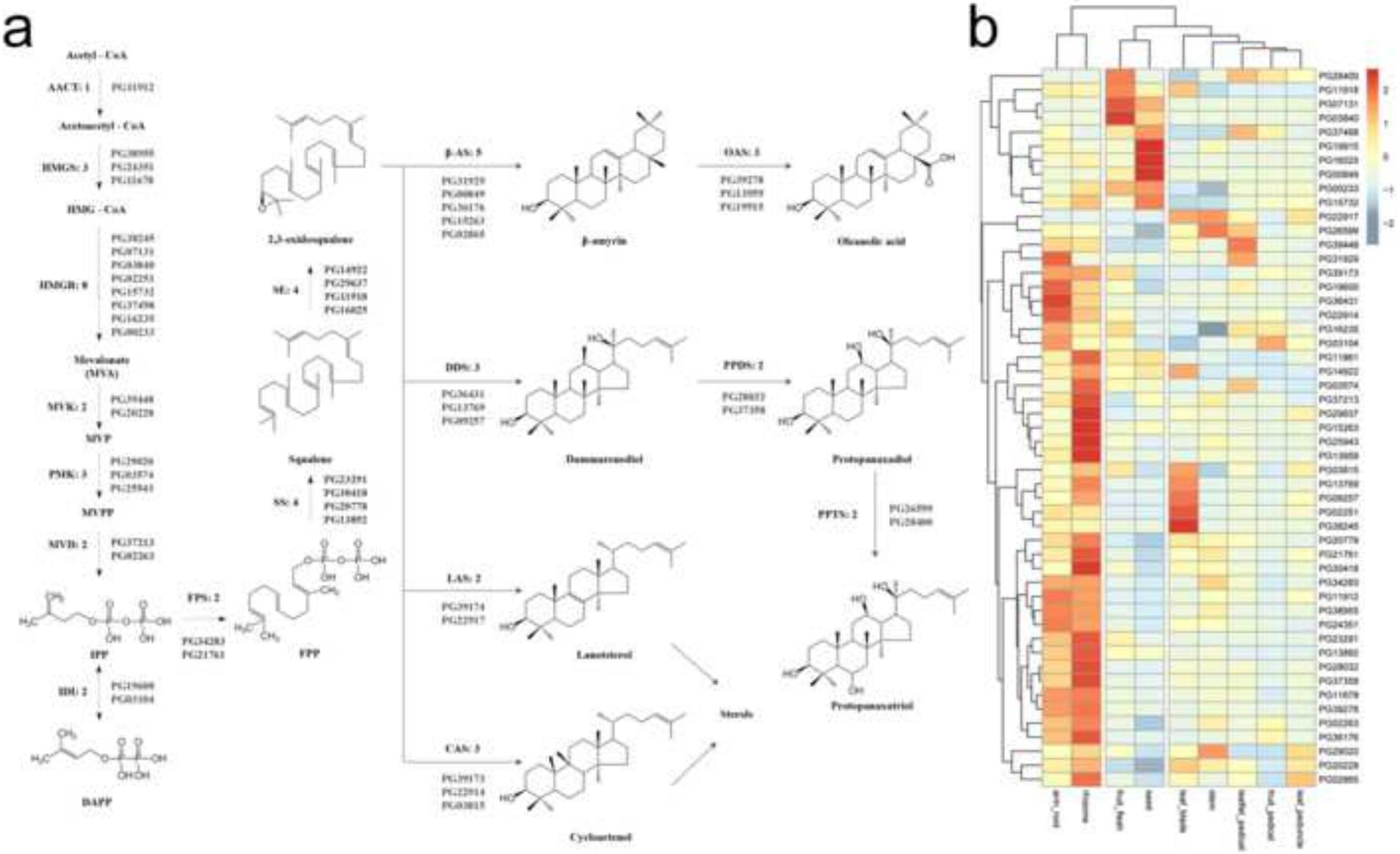

Figure 5

Click here to download Figure Figure 5.tif

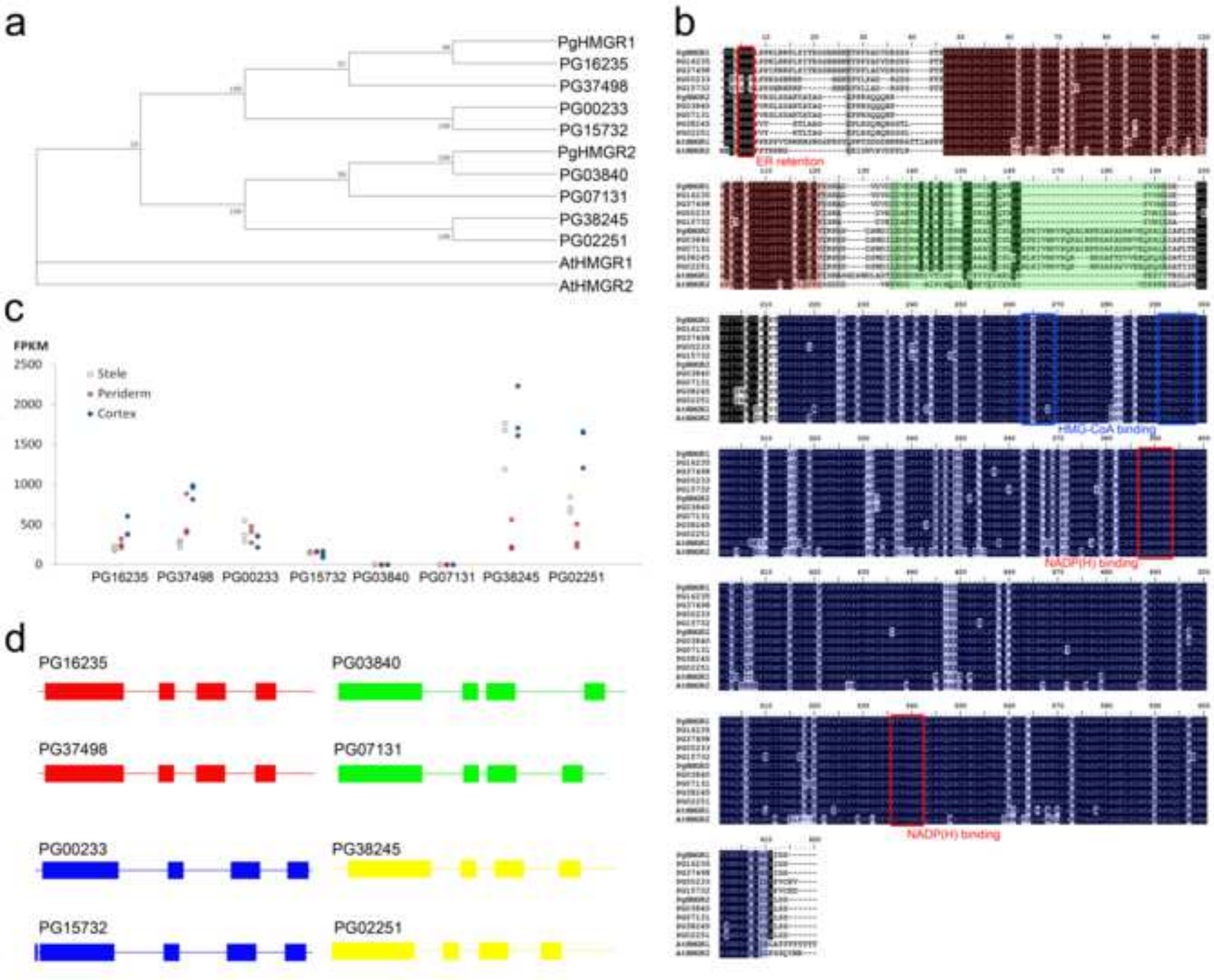

Figure 6

Click here to download Figure Figure 6.tif

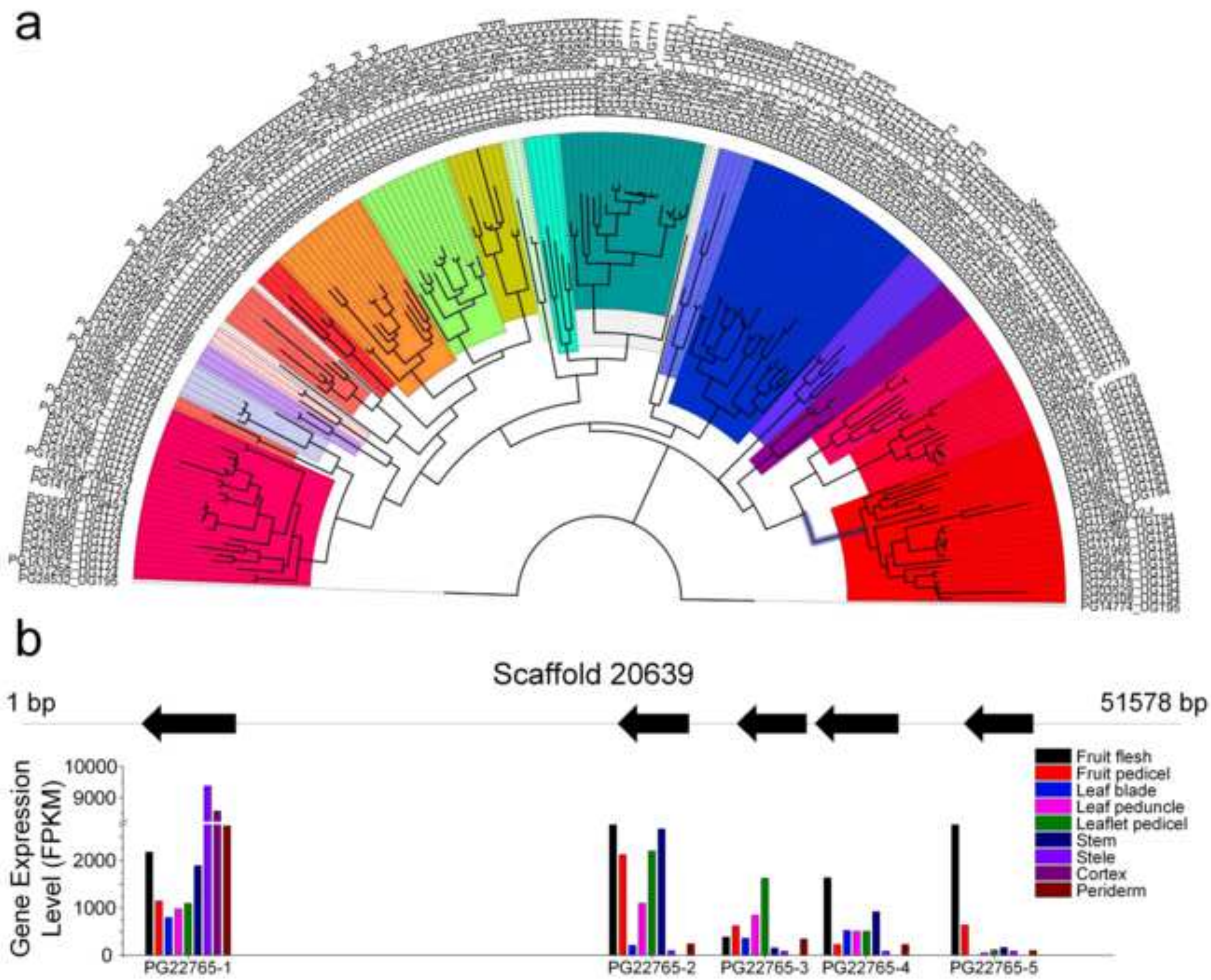

Click here to access/download
Supplementary Material
Supplementary Figures R2.docx

Supplementary Tables

Click here to access/download
Supplementary Material
Supplementary Tables R2.docx

Supplementary Text

Click here to access/download
Supplementary Material
Supplementary Text R1.docx

Dear Dr. Hans Zauner,

We appreciate you and the reviewers for your precious comments. We have carefully considered all comments for our last version and accordingly revised our manuscript. Please find below our point-by-point replies to the comments and detailed explanations of all changes (“R1” refers to the submitted revised version and “R2” is the new revised version; all revisions in R2 were tracked). All the changes were highlighted in the revised manuscript. Thank you!

Reviewer #1: The manuscript entitled: "Ginseng genome examination for ginsenoside biosynthesis" by Xu Jiang et al. was previously revised and some major considerations were made. I would like to thank the authors for answering point-by-point all my inquiries. I'm satisfied with the answers and don't have any further questions. I'm okay with this version of the manuscript for its publication.

Thank you for reviewer #1’s positive comments and thank you for permitting our work. We acknowledge the useful suggestion of reviewer #1. Thank you very much.

Reviewer #2: The manuscript has substantially improved, there is still some points that I would like to see considered (minor) and fixed (major) before publication:

Many thanks for reviewer #2’s useful suggestion. Followings are our point-by-point replies, please check them.

Minor points partially addressed:
* The single-N issue with soap scaffolding still stands. It can be overlooked, although I would recommend it mentioned for transparency. In fairness I have failed to mention it on my own manuscripts on occasion out of a lack of knowledge about the issue, but it could help further understand assembly characteristics if needed. This can affect things like read mapping and gene annotation.
Thank you for pointing out this problem. We have added the information of single-Ns at the note of Table 1 Statistical analysis of the P. ginseng draft genome.
* The justification for the use of line IR826 needs to be written into the main manuscript, as soon as this line is mentioned.
Thank you for pointing out this problem. We have added the content in Page6, Line 16.
* The fact that some tissues come from another line needs to be written too and its possible implications for the analyses discussed (i.e. do the samples

cluster by line?).

Thank you for pointing out this problem. We have added the cultivar name in Page 22 Line 1. We didn't find obvious difference among samples.

* I am not sure if the UTG analysis never included the extra expression datasets (in which case it is ok) or if it did why it is not changing.

We are sorry we didn't find any interesting information from the analysis of expression dataset in all UGTs, so we only put the expression analysis of a UGT73 gene cluster in this manuscript. We hope in future work we can get more useful information.

* The answer to my question about the ginsenosides' pathway should be included in the main text for clarity.

The pathway introduction was included in the introduction. We have highlighted in Page 4 Line 22.

* The copy number assessment justification could be included in the text for extra support (even if only in supplementary), for all relevant genes.

Thank you for this suggestion. We have added the justification at the supplementary(Supplementary Figure 10).

* Figure 1a should be a table. A table is a table, it sounds tautological but it is still true. Images do not allow automated data analysis by things like paper-crawlers and such.

Thank you for pointing out this problem. We agree with the reviewer's suggestion. We have split the table as Table 1.

Major points still standing:

* The library of nominal size 10Kbp is still referred as the "10Kbp" library in the manuscript without explanation of its actual 7.5Kbp fragment size mode. The effect of using this library as 10Kbp is noticeable both in the fragment size analysis the authors did and in the one I did. While this does not in my view invalidate the results of the assembly (most likely effect is to have some N runs of incorrect length here and there), the description of the library needs to be updated.

Thank you for pointing out this problem. We have added a column "Estimated insert size(bp)" at Supplementary Table S1. The estimated insert size was calculated using reads alignment.

* There is still no description of which lab protocol was used to produce LMP data. Mentions to transposase on the processing seem to indicate Nextera, we could not find content of nextera adaptors, so it would be good for reusability of this data to describe the protocol.

We are sorry for this negligence. Except the 2kb mate-paired library, all the libraries were constructed using the commercial library prep kits (Vazyme Biotech). The 2kb mate-paired library was constructed using 454 method(Cre/loxp recombination system), the linkage adapter was 5'-CGTAATAACTTCGTATAGCATACATTATACGAAGTTATACGA-3'. We

have added the instruction in Page 20 Line 15. The check results of adapters were listed as following:

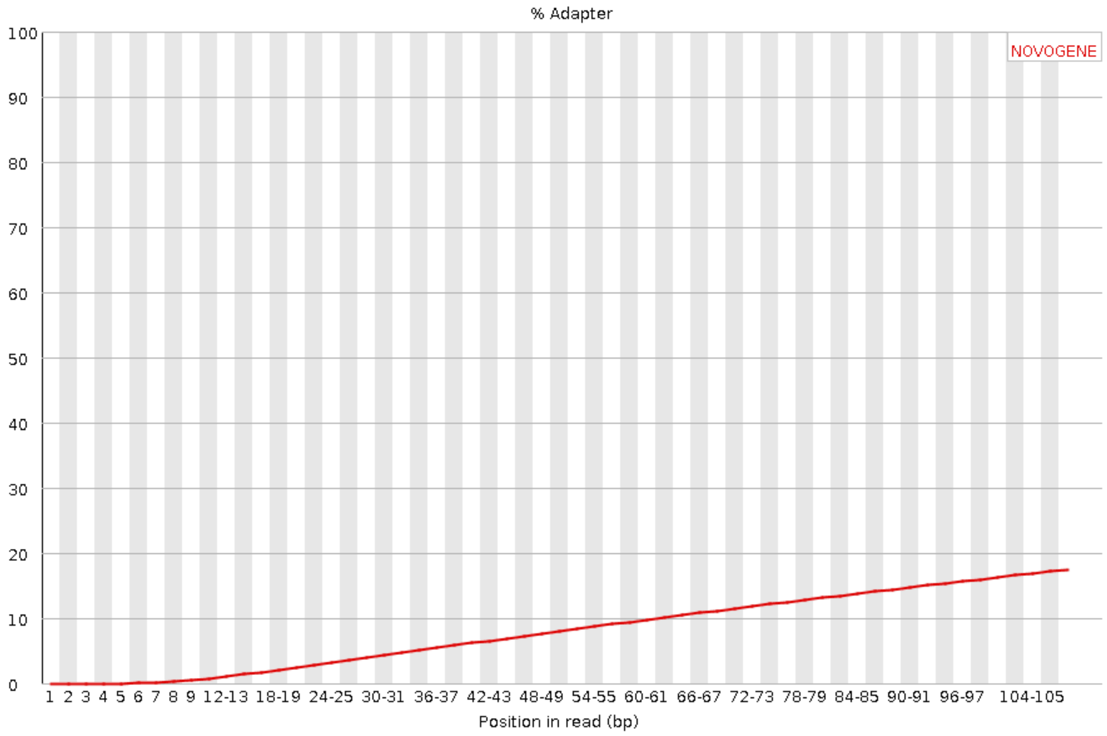

2kb

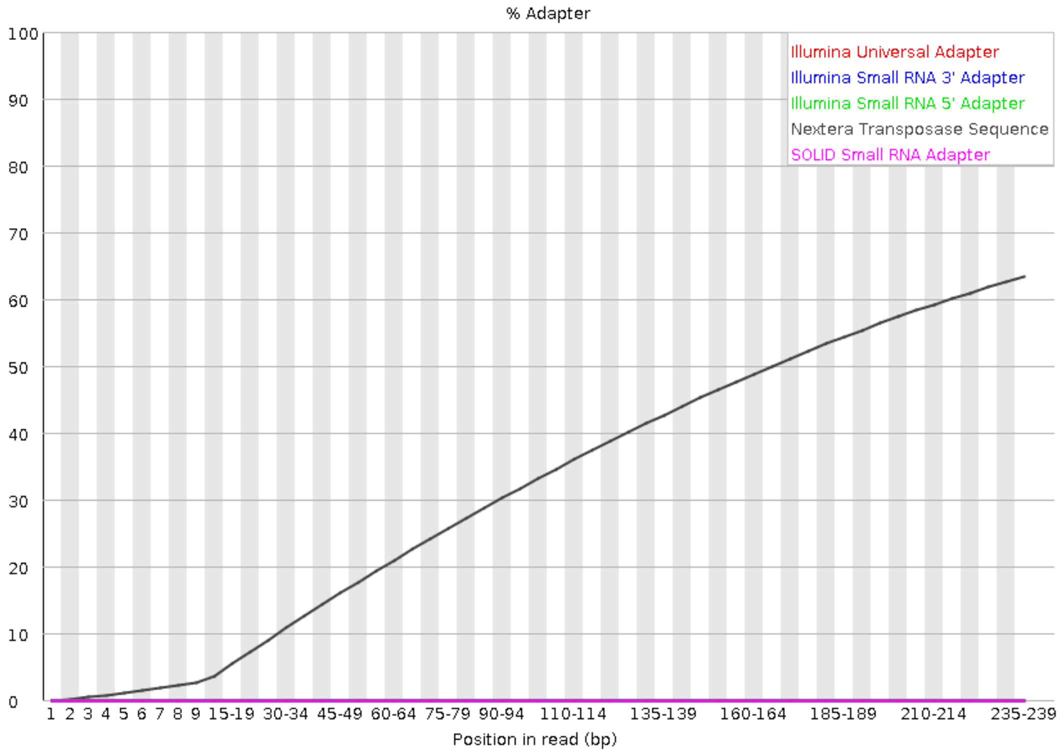

5kb

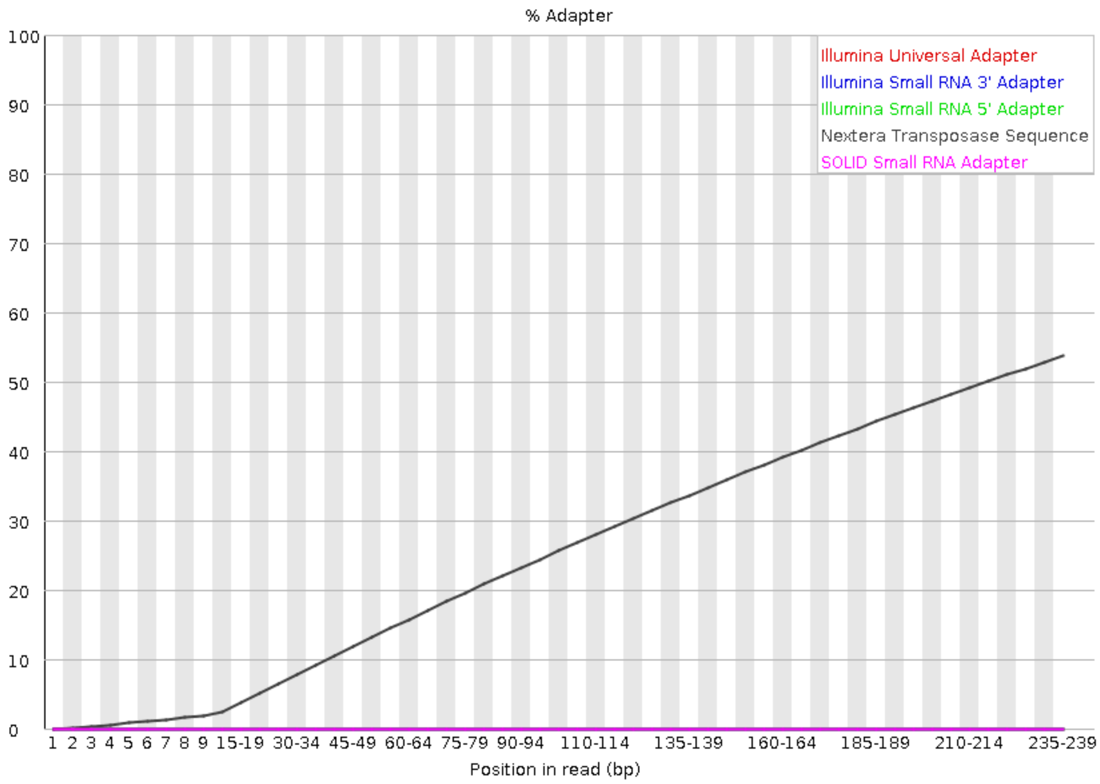

10kb

* The filtering of scaffolds <1000bp, which originates some sequence loss, is not mentioned in the manuscript. While this may be the right choice, it needs to be written and its effect of "possibly loosing some content" at the very least mentioned.

Thank you for mentioning this problem. We have noticed it in Page 21 Line 11, please check it.

* While the Figure in 5c was updated, the description was not. This could be expanded to show the main finding of this particular figure's analysis, which is unclear to me given that most of the patters are not clear now that the points are being represented.

We apologize for this negligence. The description in previous manuscript has been updated, but without labeling. Thereby, we just added the label of Fig 5c in the manuscript R2 at Page 14 Line 18. Thank you for your chariness suggestion.

* The estimation of LTR at 1.5x what was previously thought needs to be either put in context or presented with extreme care to highlight methodological differences.

Thank you for pointing out this problem. We have added the comments for the LTR change in Page 17 Line 5. We agreed with the reviewer that it should be highlighted here for the difference of BAC and WGS.
