# Supplementary material for: Panax ginseng genome examination for ginsenoside biosynthesis
Source: Gigascience. 2017 Oct 5;6(11):1–15. doi: 10.1093/gigascience/gix093 (PMC5710592; doi:10.1093/gigascience/gix093)
Supplement: Reviewer-2_Original-Submission-(Attachment).pdf [file gix093_reviewer-2_original-submission-(attachment).pdf]

Review for "Ginseng genome examination for ginsenoside biosynthesis"

Xu Jiang et al. present a work on genome assembly and annotation of *Panax ginseng*, with subsequent rna-SEQ analysis and metabolite analysis of root tissues to advance the understanding of the ginsenosides' synthesis pathway. All in all this project is interesting and the current work will merit publication once a number of major comments are resolved. The text could also benefit from some re-organisation to remove duplication, and present the information and technical details in a more concise and ordered fashion. I will focus this review mainly on the sequencing aspects of the paper, with special emphasis on the genome assembly which is my field of expertise.

I feel this paper could gain a lot by tightening up the methods. This will either provide more support for the ginsenoside biosynthesis analysis in which case the focus can highlight that result or if there is no more support for that analysis then the paper can be refocused on a stronger resource description about the genome assembly and differential expression dataset.

Major comments

Experimental design, data description and availability:

1) Reads submitted to public archives should be raw, except only for demultiplexing. This is a prerequisite for reproducibility and traceability. All data that has been submitted trimmed or otherwise pre-processed must be resubmitted.

2) There is no description of the protocols used to generate the paired end data and long mate paired data for genome assembly. Specially with the LMP data, the reads seem untrimmed, but they do contain substantial amounts of short-insert contamination which may be better accounted for if the protocol is properly described.

3) There is no justification for the choice of line IR826 over any other line, and no mention whether the independent samples for differential expression are from a single plant or different plants, and if those are from the IR826 line.

4) In general, including datasets from different studies for differential expression analysis is not automatically reliable or even comparable. The use of external datasets for every tissue but root needs to be properly supported by analysis that shows statistical validity of equivalent background conditions, plus

detailed description of similarities between experimental conditions. Also, the replicate structure of the samples should be the same. While this may be completely true, it is not discussed in the manuscript. If these criteria are not met, there should be explicit description of why this analysis is still considered valuable and its limitations.

Genome Assembly

5) There is no justification for the choice of assembler and no discussion about parameters (why that particular K, etc). Again, detailed description of the parametrisation for each tool is needed, with some justification when appropriate.

6) I analysed the assembly with a KAT spectra-CN plot (using k=31, only the short-insert libraries), with the following result:

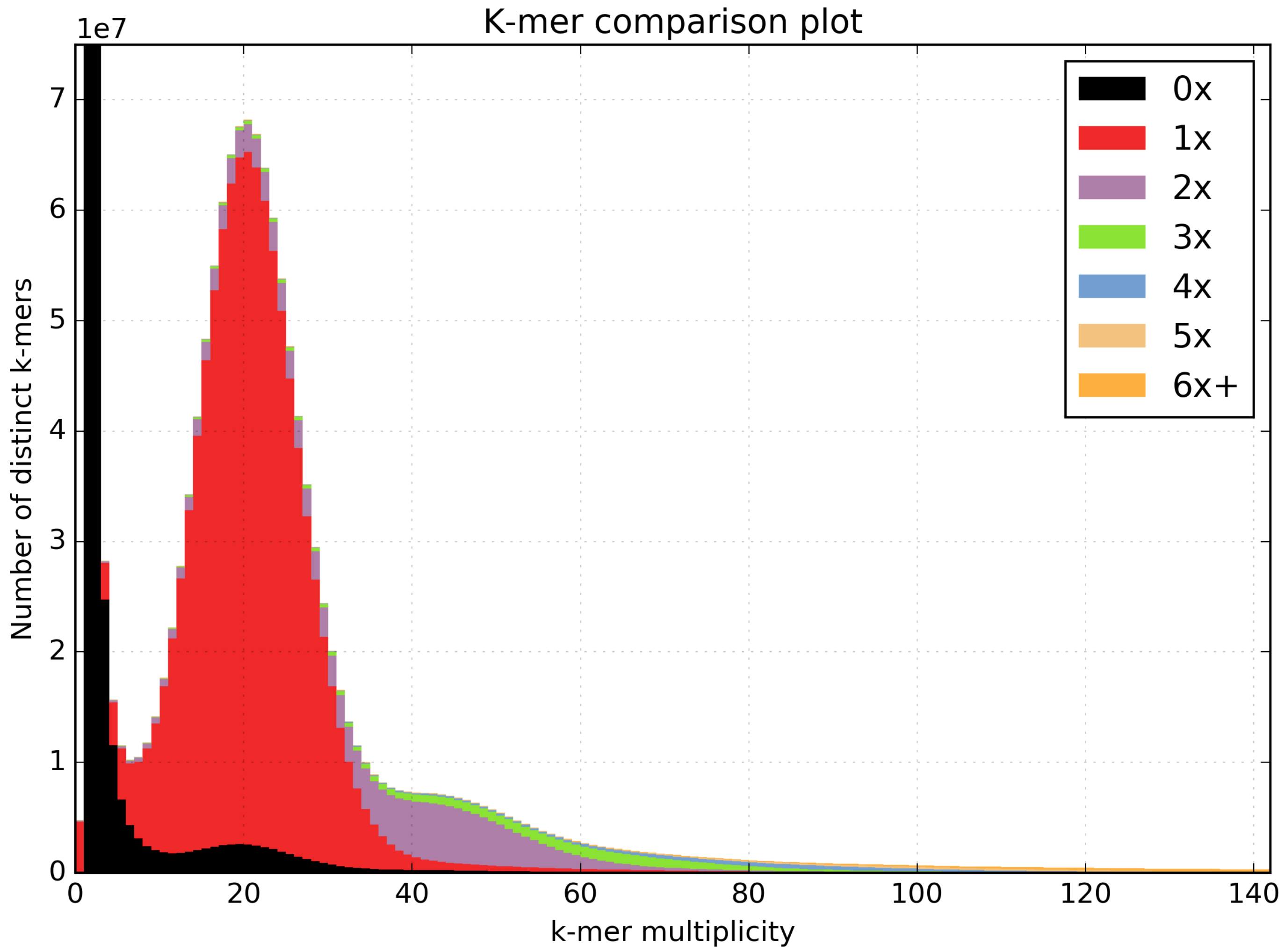

This leads to a number of comments:

7.1) The kmer coverage at k=31 is around 20x using both PE libraries, which is lower that would be recommended to construct contigs (around 35x to 100x is probably best practice nowadays). I expect the coverage at the K used for assembly to be even lower.

7.2) There is a loss of single-copy content (represented by the black distribution's peak at x=20) and generation of some content in the assembly not present on the reads (x=0). While this can be explained because of the gap closing and some N inclusion in the assembly, it merits some discussion and validation.

7.3) There is duplication on the main single-copy distribution at x=20. This could explain some of the content loss (again, due to gap closing? analyses before and after gap closing would make this clear). At the same time, SOAPdenovo2's scaffolding introduces a single N between contigs when a negative distance between them is estimated by the scaffolding algorithm. This produces both unnecessary duplication of sequence and some misassembles. There are more than 350K instances of single Ns on the assembly and soap does not output Ns on the contigs, so this should be examined.

```
$ egrep -c '[ACTG]N[ACTG]' Ginseng_genome_assembly_v1.fasta
368679
```

8) The scaffolding method is slightly unorthodox, consisting of contig assembly and scaffolding with SOAPdenovo (I assume SOAPdenovo2, but it is not properly cited and no version is mentioned), then gap closing, then a further round of scaffolding with SSPACE. In general there are better and more modern methods to do this same work. Running an ABySS assembly end-to-end and comparing the results may be a good starting point.

8.1) Re-scaffolding with the same datasets should not be needed, so I would suggest the authors either just use SOAP's contigs into SSPACE, or do not perform a second round of scaffolding. Performing gap closure before any scaffolding is also not recommended, so in any case this should be the last step in the assembly. As this assembly procedure does not follow best practices, I would have also expected to see a detailed description of the results at every step (i.e. contiguity, N content, etc). Also, the detailed configuration files and commands executed to produce the assemblies should be provided on the supplementary material and referred to in the main text (this is valid for all other analyses).

8.2) Any scaffolding such as this needs some validation, at the very least by analysing synteny with related species, and or by producing wet-lab validation of some junctions.

8.3) We've run library fragment size analysis in a the LMP libraries by mapping them to the assembly and found significant short-insert (pair end orientation) contamination. Also, the fragment size distributions change if the assembly is split at N runs: peaks at exactly the "nominal" distances, specially for the 10Kbp library which is actually 7.5Kbp, disappear. This indicates some effect of overscaffolding. Doing this kind of analyses at every step of the assembly pipeline would help evaluate where biases are introduced.

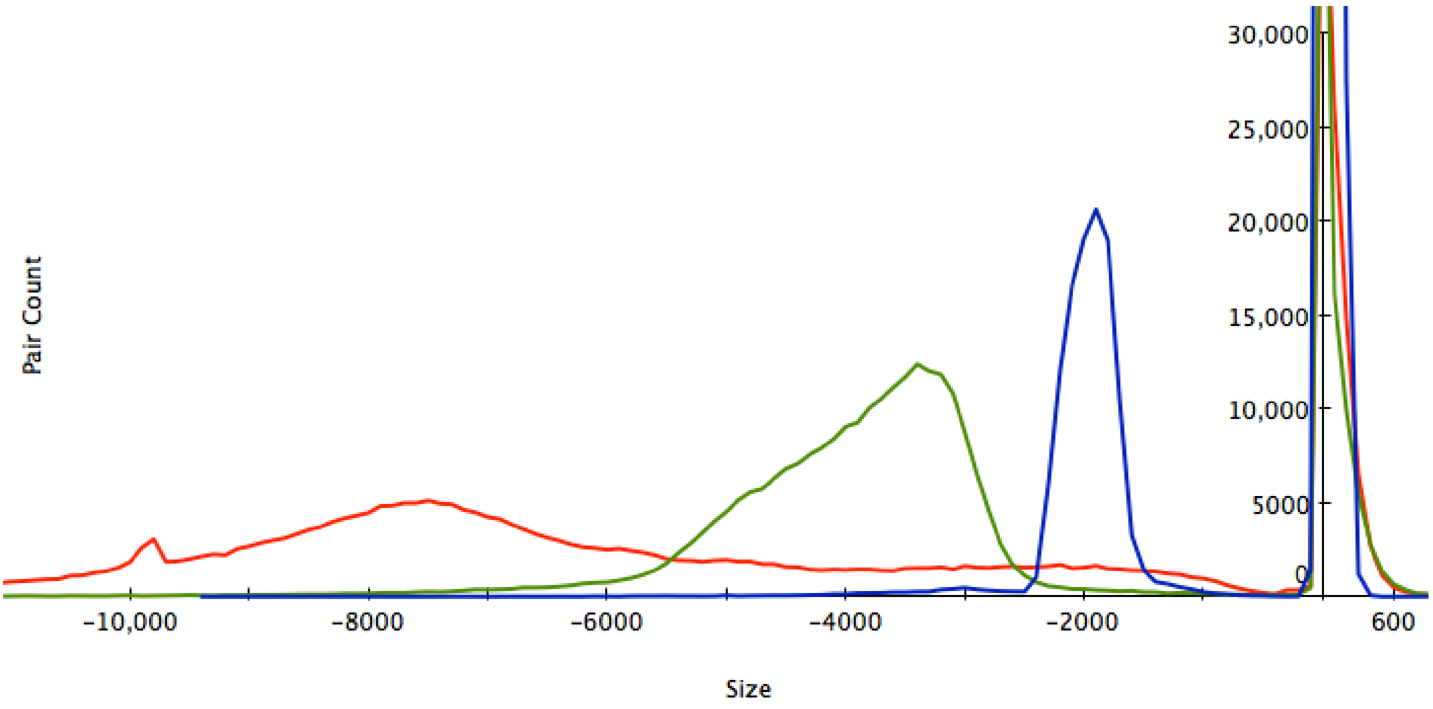

8.4) The 10Kbp library is in fact 7.5Kbp so all references to that size should be updated. If 10Kbp has been used as size in software that does not correct for it automatically it may need to be re-run.

9) Transcript assembly mappability is shown as a metric of assembly reliability, but it is unclear that any *de novo* transcript assembly was performed. If that is the case, the *de novo* transcript assembly must be properly described and discussed.

10) The mapping of the 500bp library to assess non-bias is ad-hoc and I am not aware of methods that would support this (it also introduces mappability bias, etc, etc). For completeness I would prefer either metrics based on how well the original reads map back to the assembly or simply kmer spectra completeness (Disclaimer: I am the senior author of the KAT publication).

RNA-seq and differential expression

11) The whole method of the transcript assembly, mapping support and numbers does not add up easily. A diagram of the annotation pipeline would clarify this, with numbers of transcripts, support for them, etc. This is a KEY point if this manuscript wants to present a resource for the community.

12) As mentioned in (4) the usage of dataset from previous studies needs more justification and support.

13) Figure 3e is unclear and probably also belongs in the supplementary.

14) Figure 4a shows "a possible pathway" but gives little extra detail or justification and it is not clear from the main text neither. I think this should be expanded and properly described in the main text.

15) Figure 4b shows an extremely different expression levels between root and all the other tissues. While this is expected and could be supportive of other claims in the manuscript, this also shows the division of

new vs. pre-existent datasets. I think differential expression between the new datasets on this manuscript should become the main focus of this figure and the relevant section. If RNA-seq data for the other tissues can be regenerated and/or shown to be perfectly comparable for this purpose, then I would suggest using differential expression patterns to test the robustness of the proposed pathway membership among the proposed genes, and even maybe trying to find "missing members" of the pathway.

16) Figure 5b uses bars and SD to show 3-point datasets. This hides the real data and leads to a feeling of over-confidence in what is effectively a small-n case. Replace with a plot showing every point. The mean can be indicated within this plots with a line, but SD is mostly meaningless with N=3.

17) I can't finish to understand Figure 7, and I am conscious about the rna-seq dataset origin is playing a role here too.

Pathway analysis

18) The first paragraph of this section is difficult to read, rewrite for clarity, please.

19) Copy number assessment of genes based on a WGS fragmented assembly as this one should at the very least be validated by using kmer coverage and/or read mapping depth. Moreover since there is a mention to putting together some of the genes manually because they were fragmented on the original assembly.

Microbial resistance

20) I am not very sure how this section relates to the rest of the manuscript.

21) The seven-time-points experiment is a whole analysis on its own, same need for experimental design explanation and rationale as in previous sections apply.

22) The last sentence is too strong on its claim for the evidence presented and seems to have no experimental validation.

Discussion

23) The claim for LTR content being significantly higher than previously thought is a huge one, and should be supported better starting by a proper statement of the methods used in both studies. *De novo* methods for LTR content estimation exist that can give a figure without the bias of the assembly. If this claim is well supported it should feature more prominently, as it will probably change the understanding of the genome's history and evolution. Also, further dating of LTRs and extra analyses could help elucidate why the results are so different to previous reports.

Minor comments

1) Figure 1a should be a table, Figures 1b and 1c needs either more explanation over new findings or important confirmation or may just be moved to supplementary. I personally find it impossible to extract any information from figures like 1c.

2) The abstract should be adjusted to get rid of unnecessary nitty-gritty details, and provide a more focused description of what the manuscript is aiming for (assembly+rnaseq or biosynthesis analysis, but tighter and more conclusive). Conclusions should not be along the lines of "this will help further work" but at the very least along the lines of "this supports this, this, and this types of analyses/developments".

3) The organisation of the text could be much clearer (i.e. have all the data about experimental design and sequencing for genome assembly in one place in the methods, the same for differential expression, etc).

4) There is no justification on the plant organisms selected for comparison.

5) Figure 3b and 3c are plotted in such a way that some points are "hidden" behind other points.

6) Figure 5 is the first point where "4-year-old" is mentioned. This belongs in methods and needs some explanation.

7) Figures 6b and 6c provide no information. Should be removed and/or a section (possibly in the supplementary) describing their results and importance should be explicitly written.

8) The sections for phylogenetic analysis and gene family identification are vague and/or add little information for the rest of the manuscript's analyses. They can either be moved to supplementary or their links to the rest of the manuscript stressed and the analyses strengthened.

9) In general the discussion has some claims that do not seem completely supported from the current analyses, but I feel a plant biologist will be better suited to judge that.

With my best regards,

Bernardo J. Clavijo

NOTE: review produced with extensive help from Luis Yanes from my research team.
