# Supplementary material for: Panax ginseng genome examination for ginsenoside biosynthesis
Source: Gigascience. 2017 Oct 5;6(11):1–15. doi: 10.1093/gigascience/gix093 (PMC5710592; doi:10.1093/gigascience/gix093)
Supplement: Supplement Tables and Figures [file gix093_supp.zip › Supplementary Figures R2.docx]

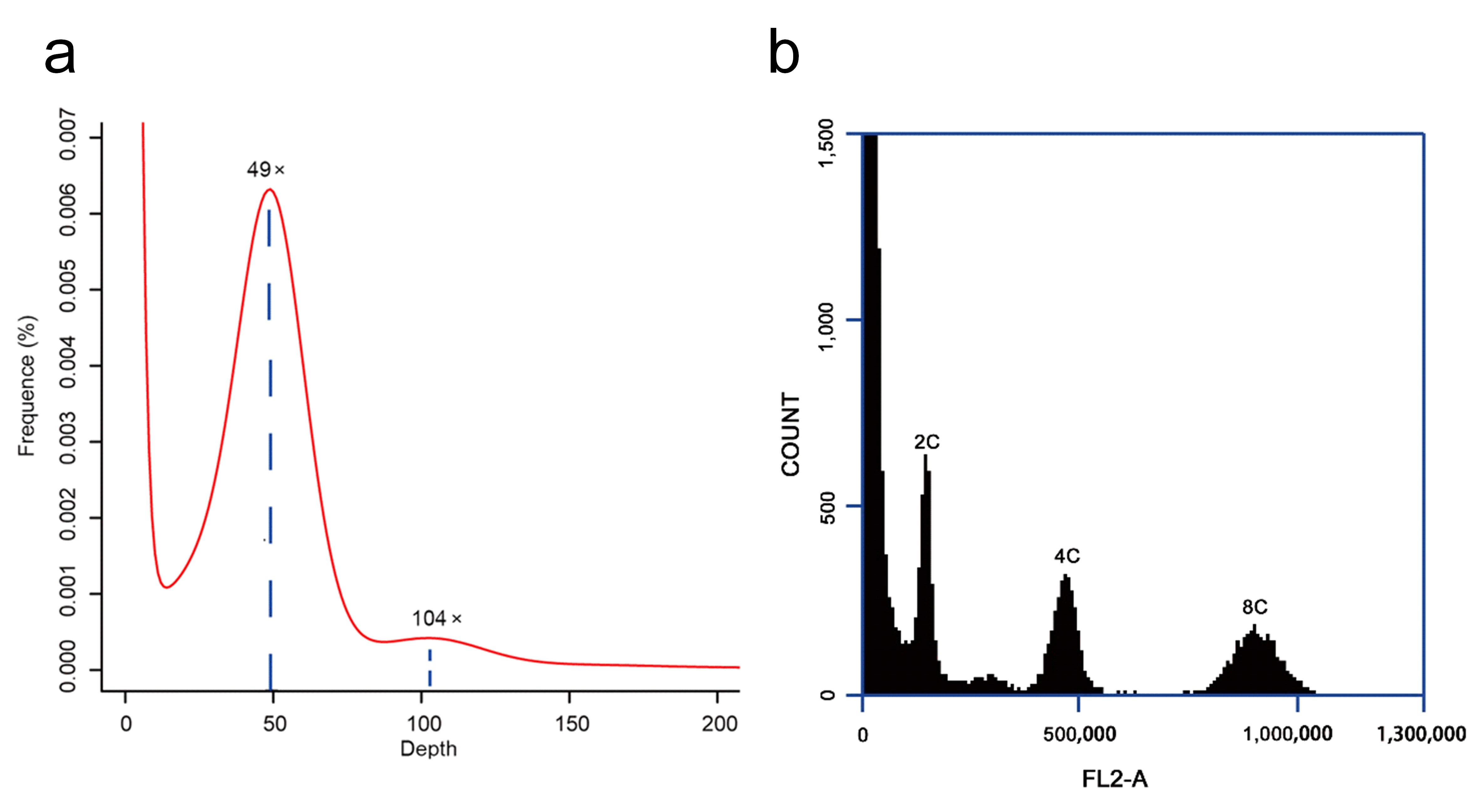


**Figure S1. The genome size of *P. ginseng* estimation. (a)** Distribution of 17-mer frequency. In total 183.82 Gb of high-quality short-insert reads (250 bp and 500 bp) were used to generate the 17-mer depth distribution curve frequency information. **(b)** Flow cytometry analysis by comparison with *Glycine max*.


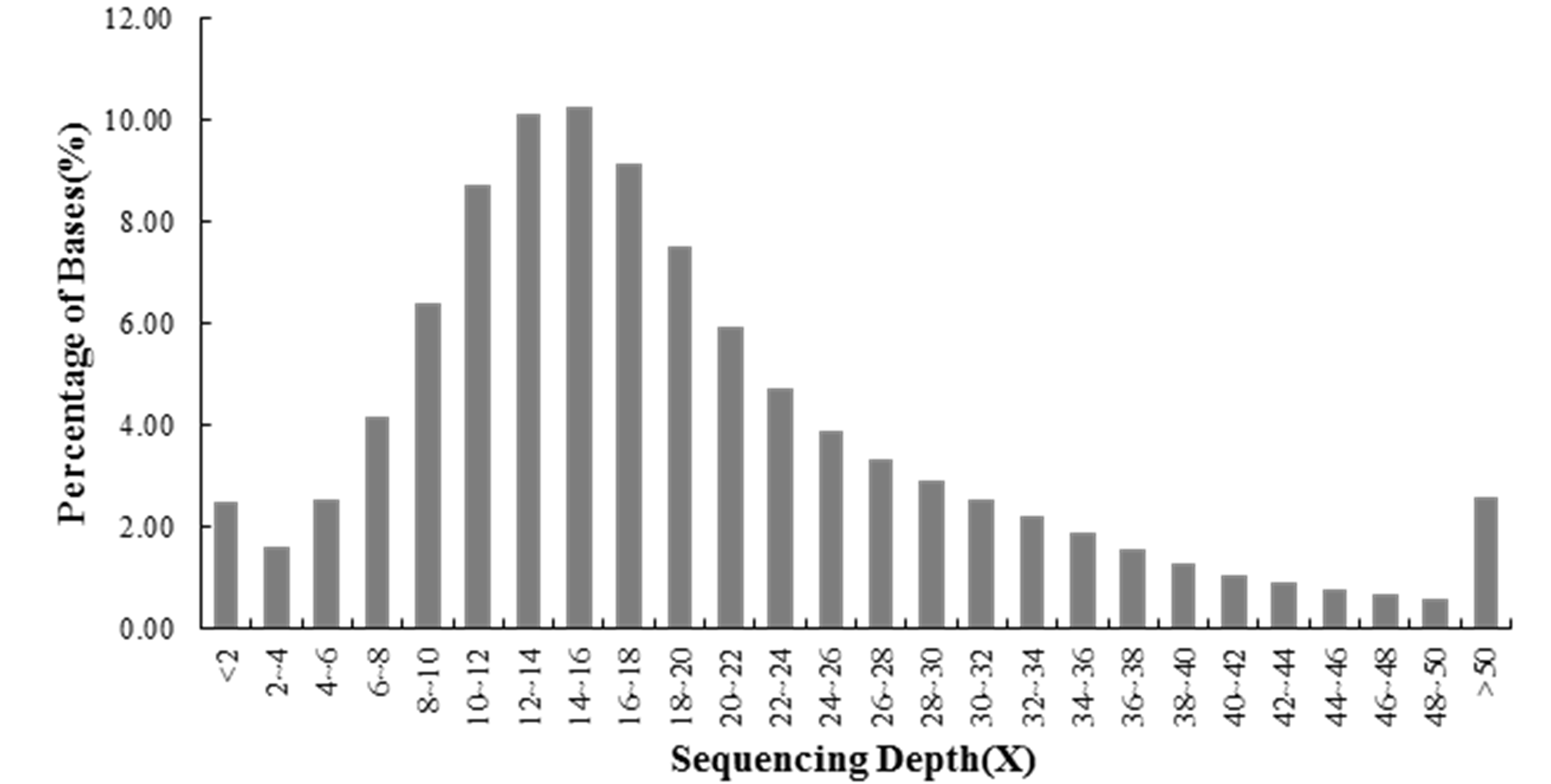


**Figure S2. Distribution of sequence depth for the *P. ginseng* genome.** The x-axis and the y-axis represent the depth and percentage of corresponding DNA bases.

**
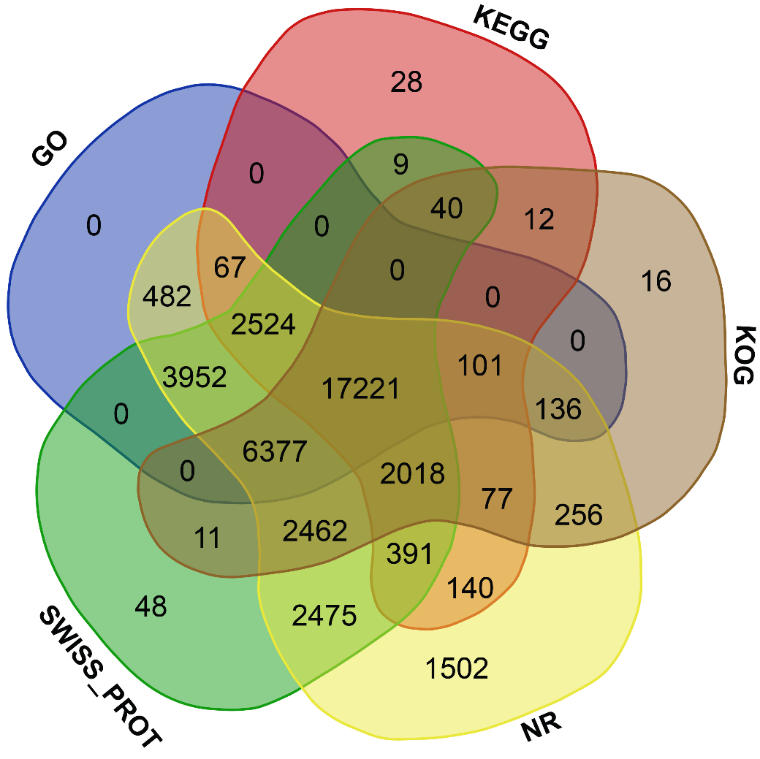
**

**Figure S3. Venn diagram of the distribution of functional annotated genes searched against Nr, GO, KEGG, KOG, and SWISS-PROT databases.**


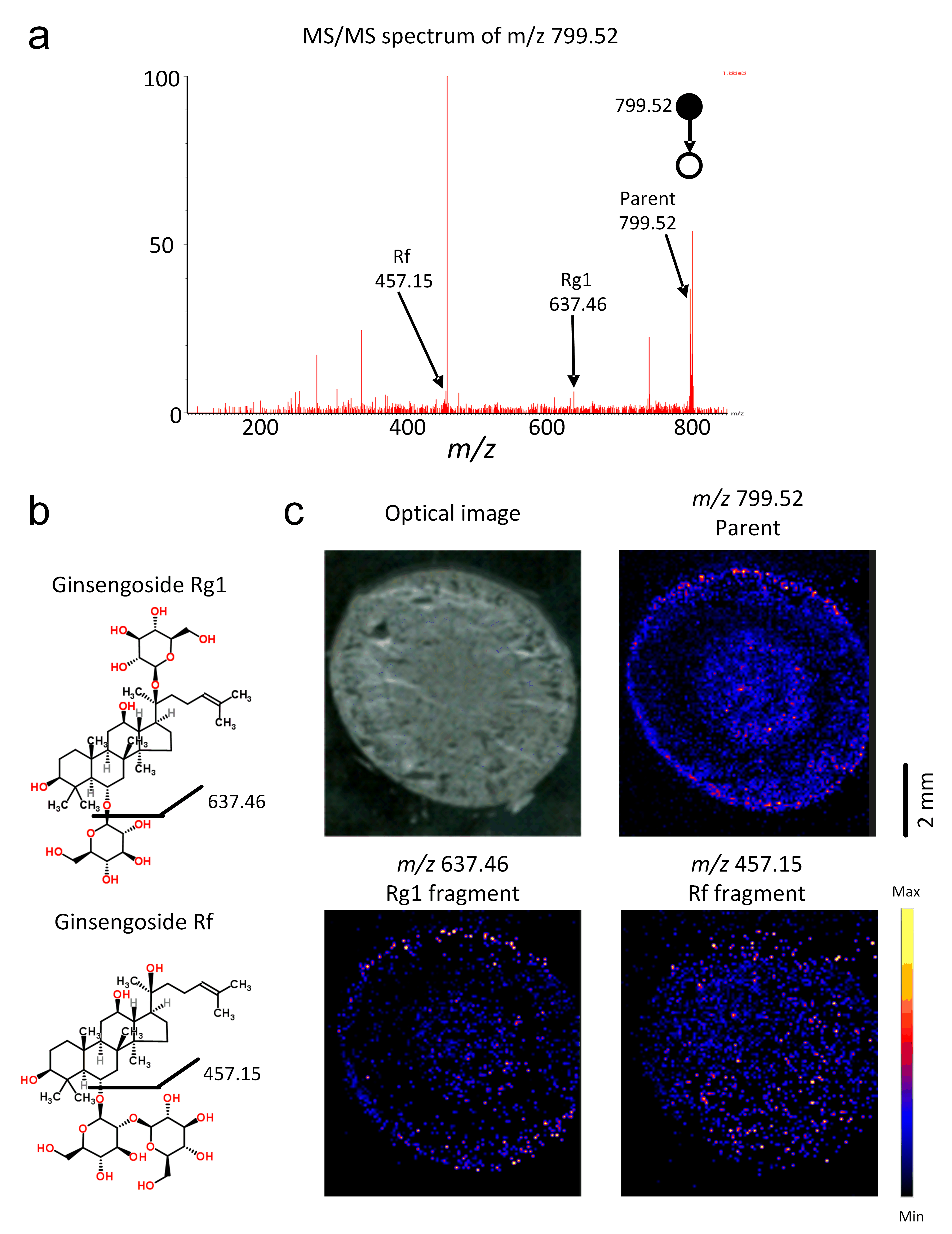


**Figure S4. DESI-MS/MS images of Rf and Rg1 in *P. ginseng* root cross sections. (a)** MS/MS spectrum of m/z 799.52. **(b)** Molecular structural formula of Rg1 and Rf. **(c)** The DESI-MS/MS images of Rg1 and Rf.


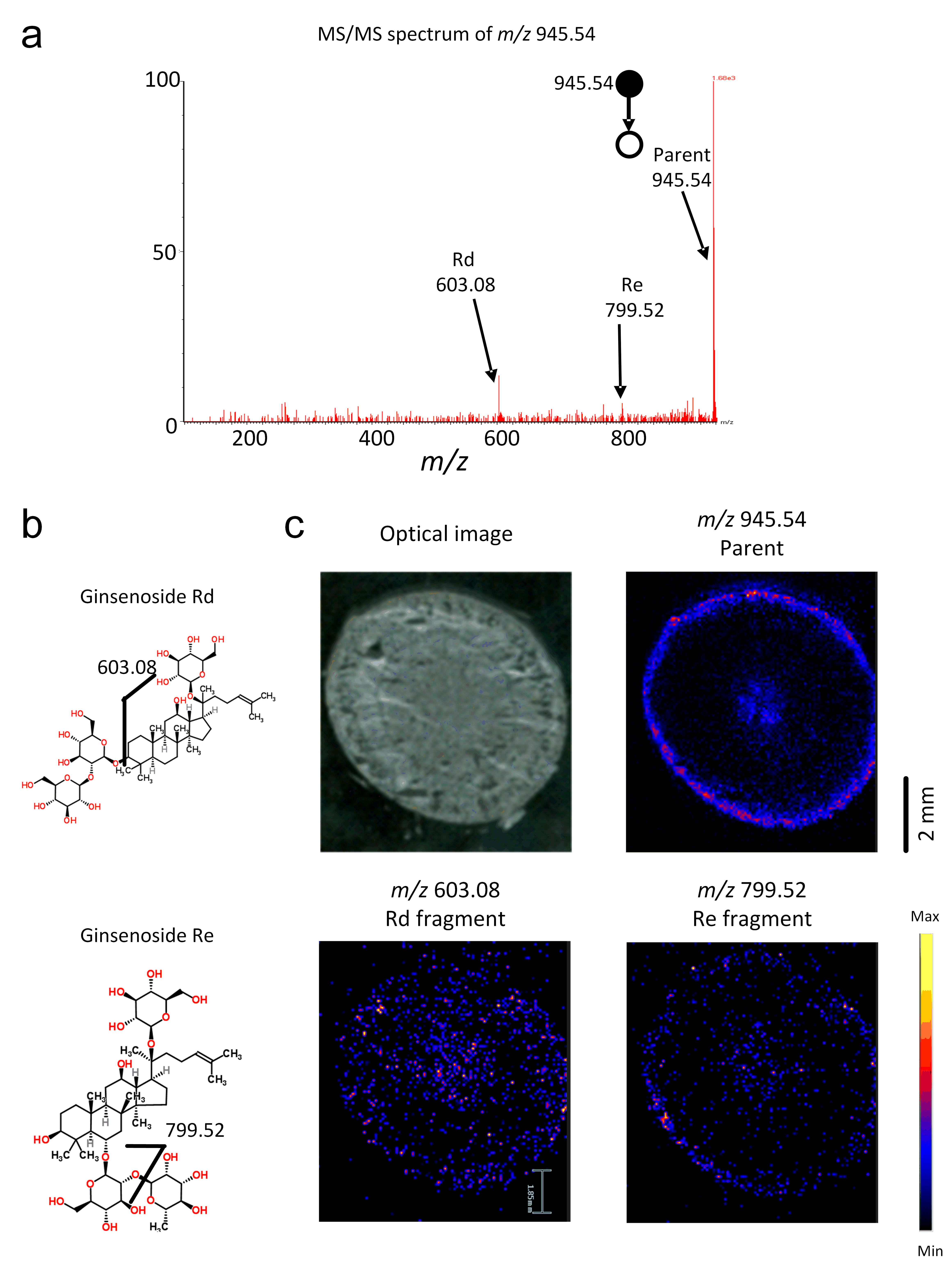


**Figure S5. DESI-MS/MS images of Rd and Re in *P. ginseng* root cross sections. (a)** MS/MS spectrum of m/z 745.54. **(b)** Molecular structural formula of Rd and Re. **(c)** The DESI-MS/MS images of Rd and Re.


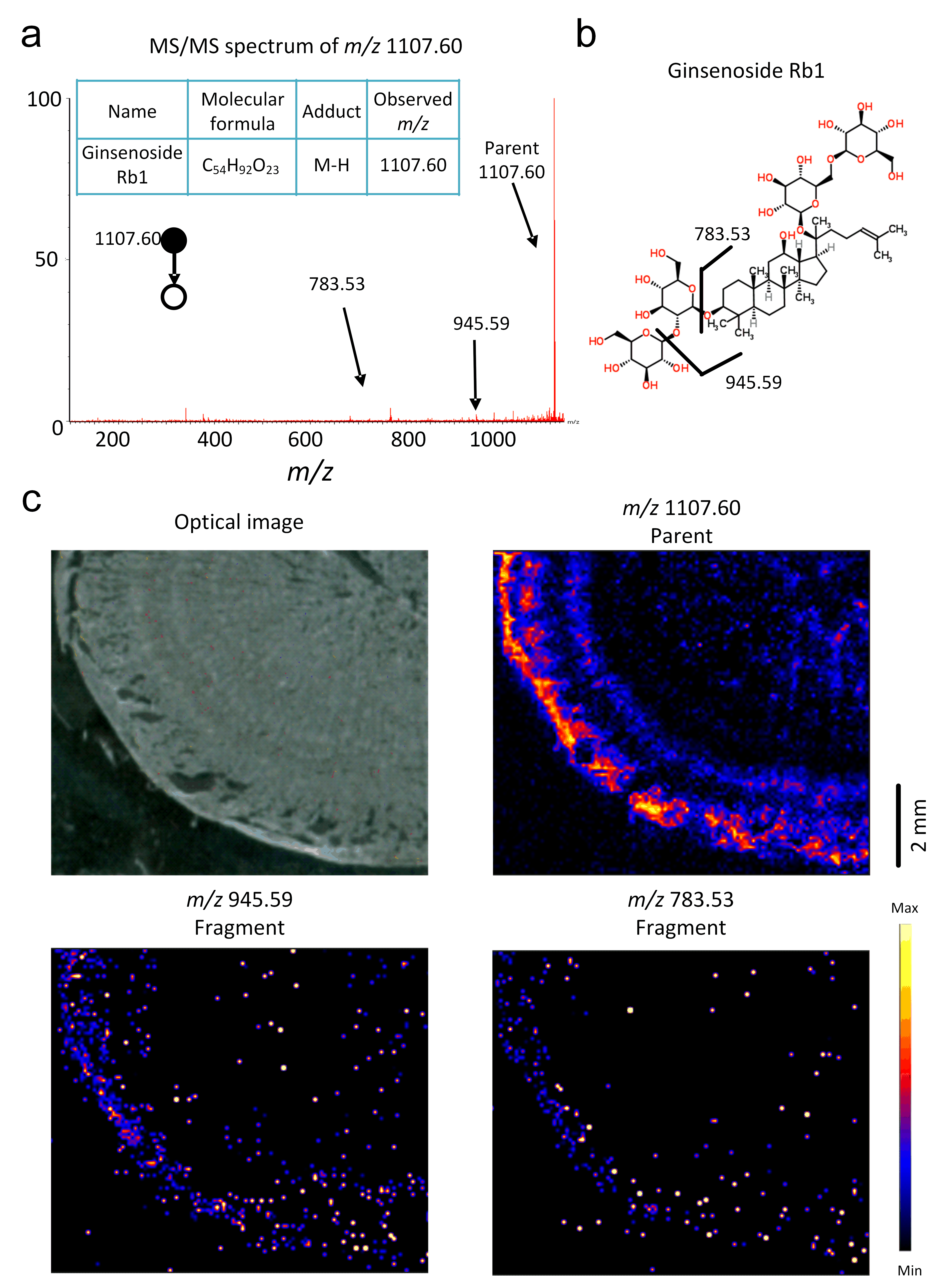


**Figure S6. DESI-MS/MS images of Rb1 in *P. ginseng* root cross sections. (a)** MS/MS spectrum of m/z 1107.60. **(b)** Molecular structural formula of Rb1. **(c)** The DESI-MS/MS images of Rb1.


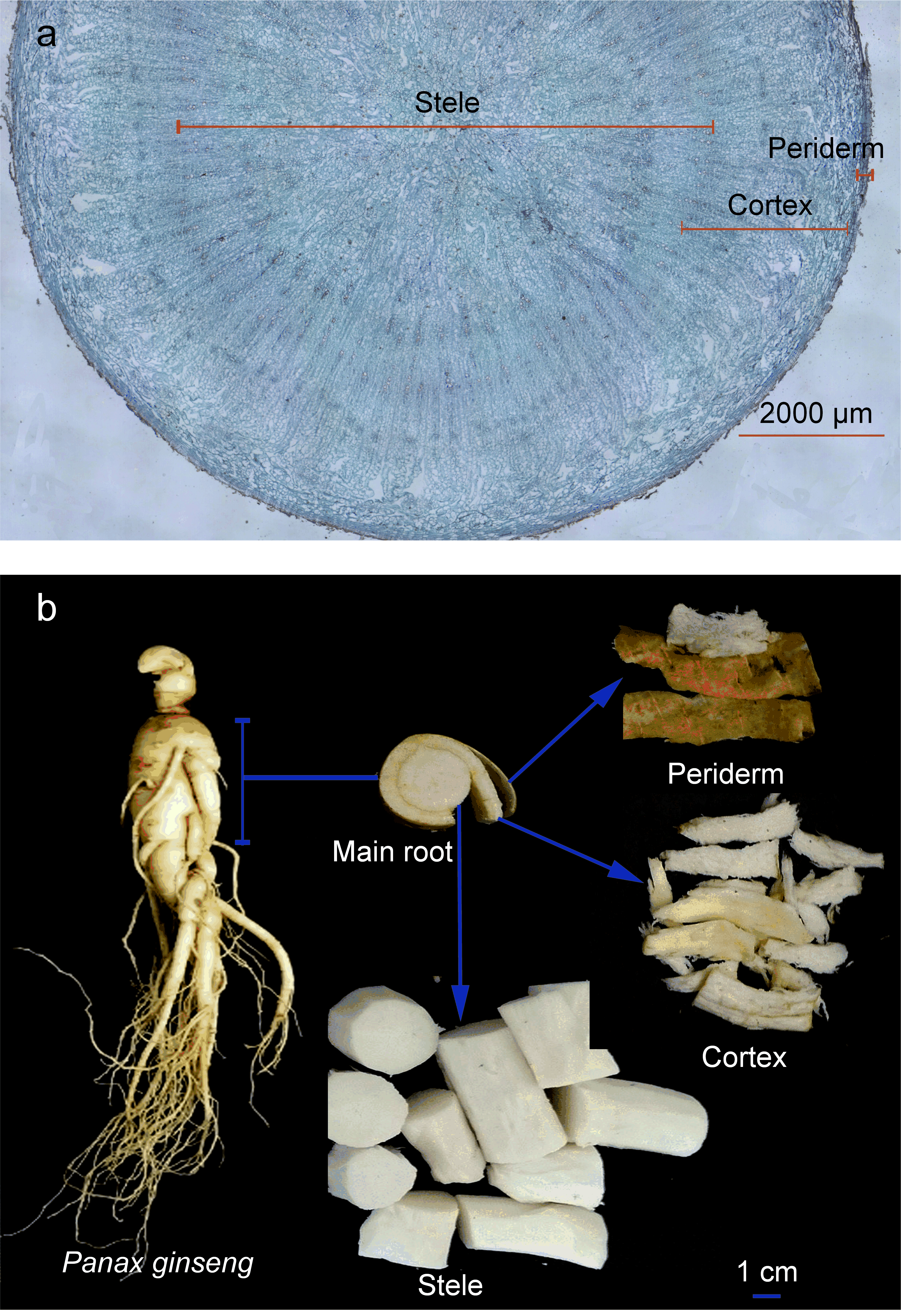


**Figure S7. The root of 4 years of *P. ginseng*. (a)** The microscopic section of the main root of *P. ginseng*. **(b)** The main root were peeled into three parts roughly, including periderm, cortex and stele.


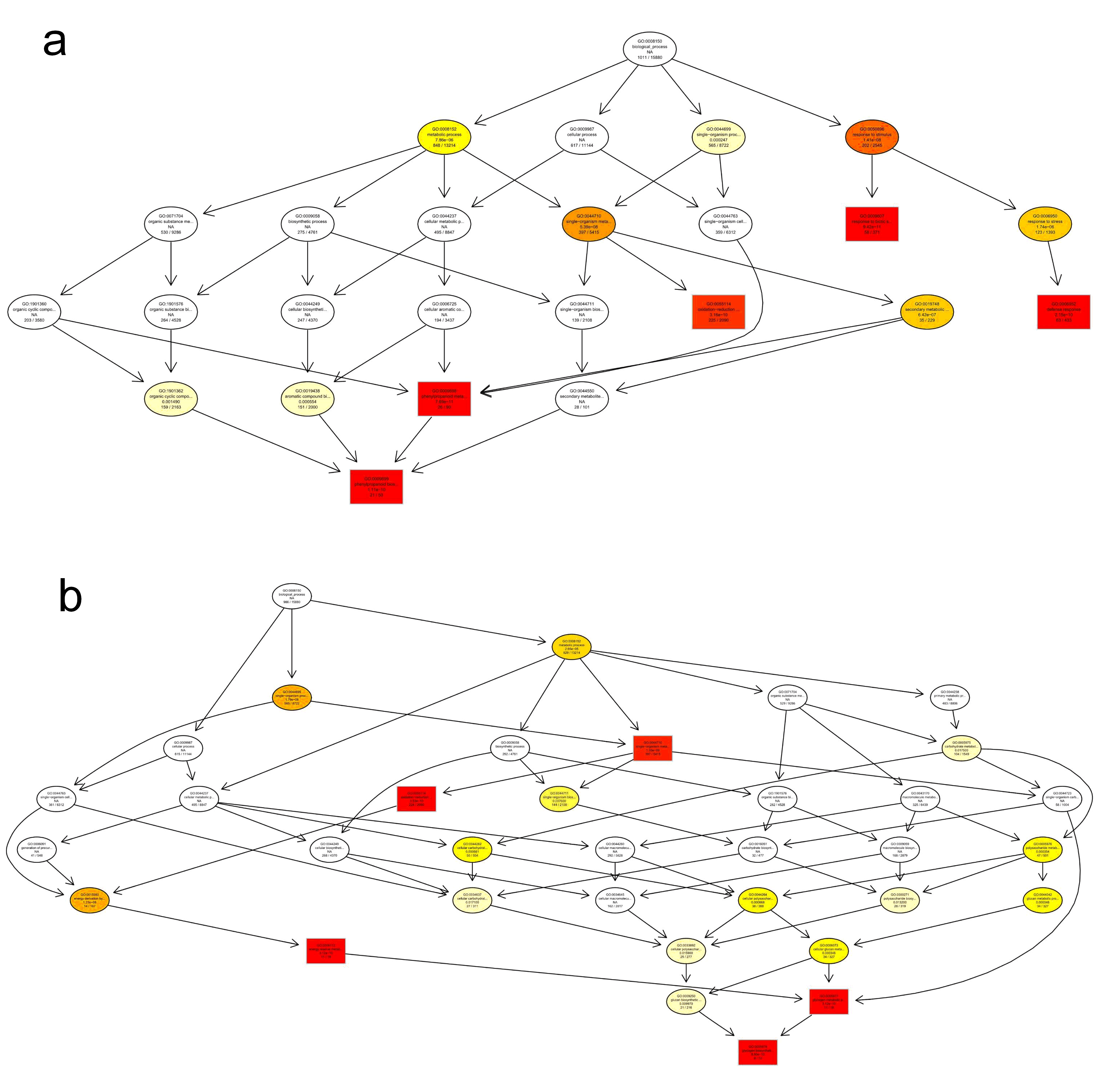


**Figure S8. The GO enrichment analysis of genes expression in ginseng root sections.** The differential genes between **(a)** periderm *vs* cortex and **(b)** periderm *vs* stele.

**Figure S9. Hierarchical cluster tree showing coexpression modules identified with 42006 genes (among which, 7456 genes with slight variance were excluded from the analyses) through the WGCNA.** The modules corresponding to the branches are represented by colors in the first color band underneath the tree, and the remaining color bands reveal highly correlated (red) or anticorrelated (blue) transcripts for the total ginsenosides, Rb1 and Rg1. “Red” indicates a highly positive correlation with the corresponding gene, “white” denotes a weak correlation, and “blue” module represents a highly negative correlation.


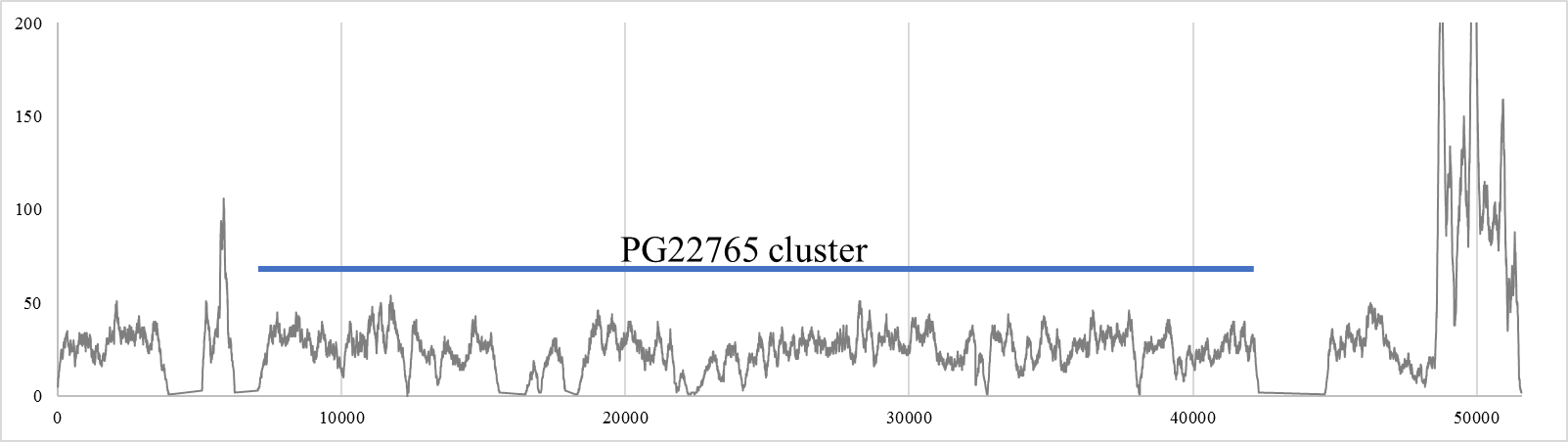


**Figure S10 Sequencing depth of PG22765-cluster.** The alignment information (.bam file) of the Scaffold20639 which contains the PG22765 UGT gene cluster was extracted from full alignment (mapping 500 bp library reads to draft genome using BWA-mem) and used to calculating sequencing depth. Except for the gap, repeat region, or high-GC-content area, the mapping depth was around 20X.


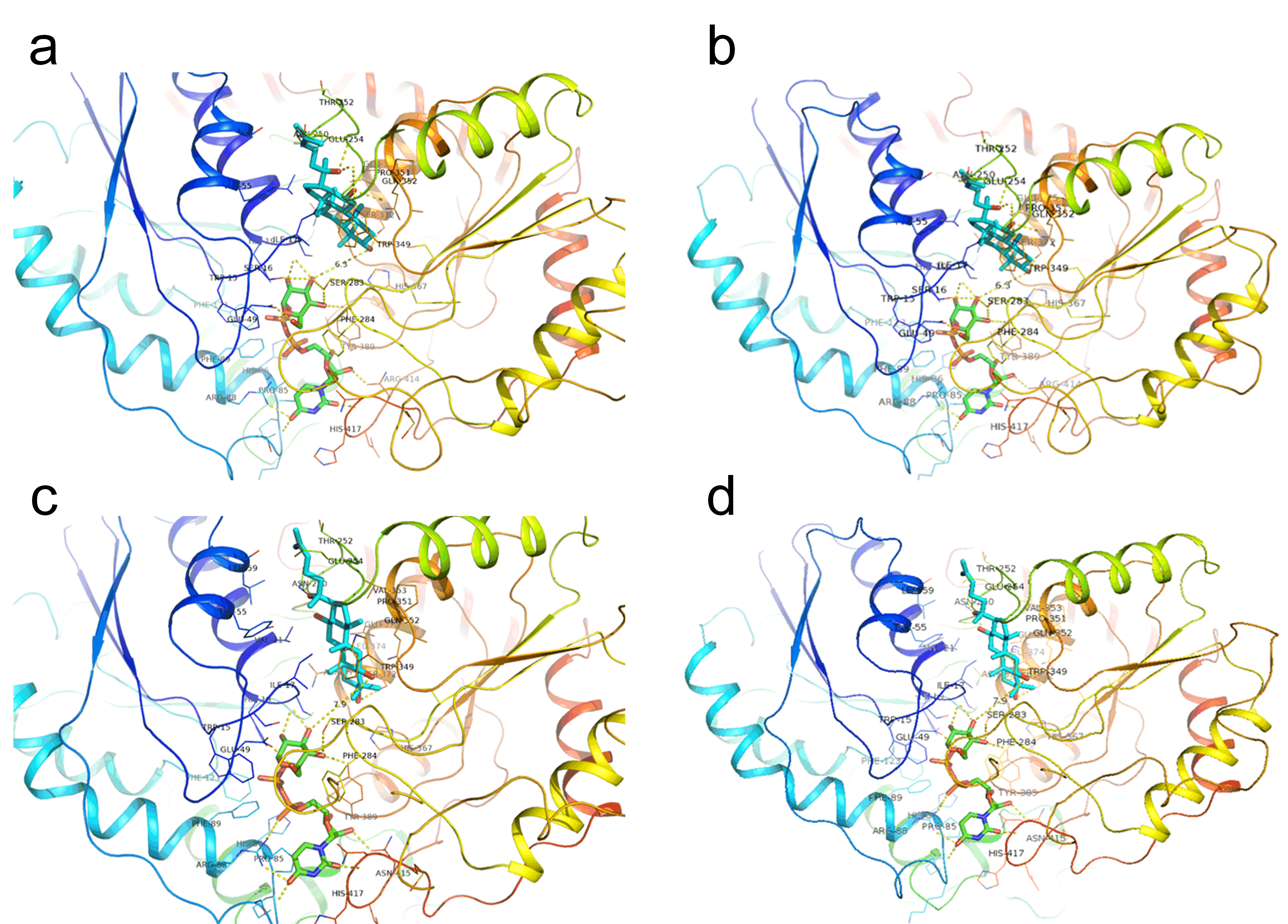


**Figure S11. Homology docking of PgUGT23 enzyme from *P. ginseng*. (a)** PgUGT23 protopanaxadiol ligand site. **(b)** PgUGT23 protopanaxadiol overall structure. **(c)** PgUGT23 protopanaxatriol ligand site. **(d)** PgUGT23 protopanaxatriol overall structure.


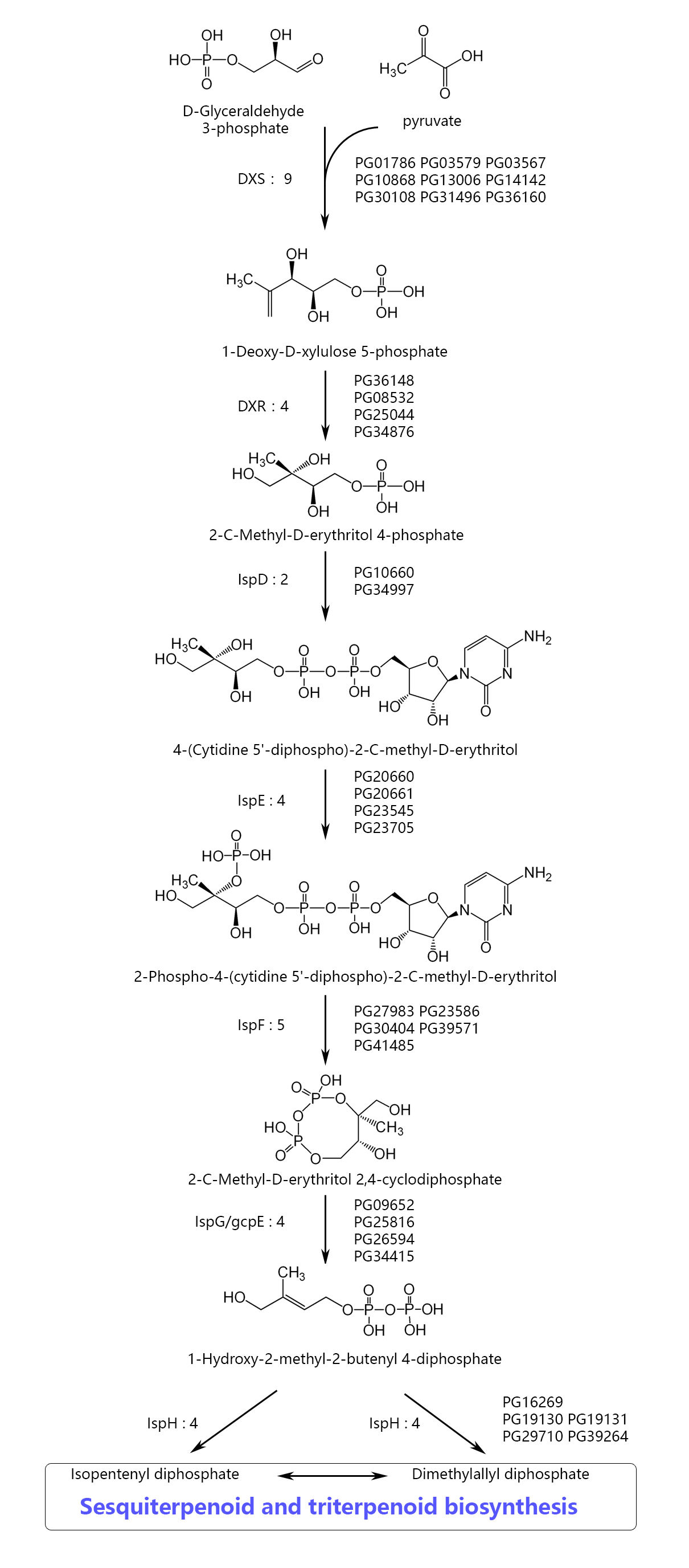


**Figure S12. The putative proteins involved in MEP pathway.**


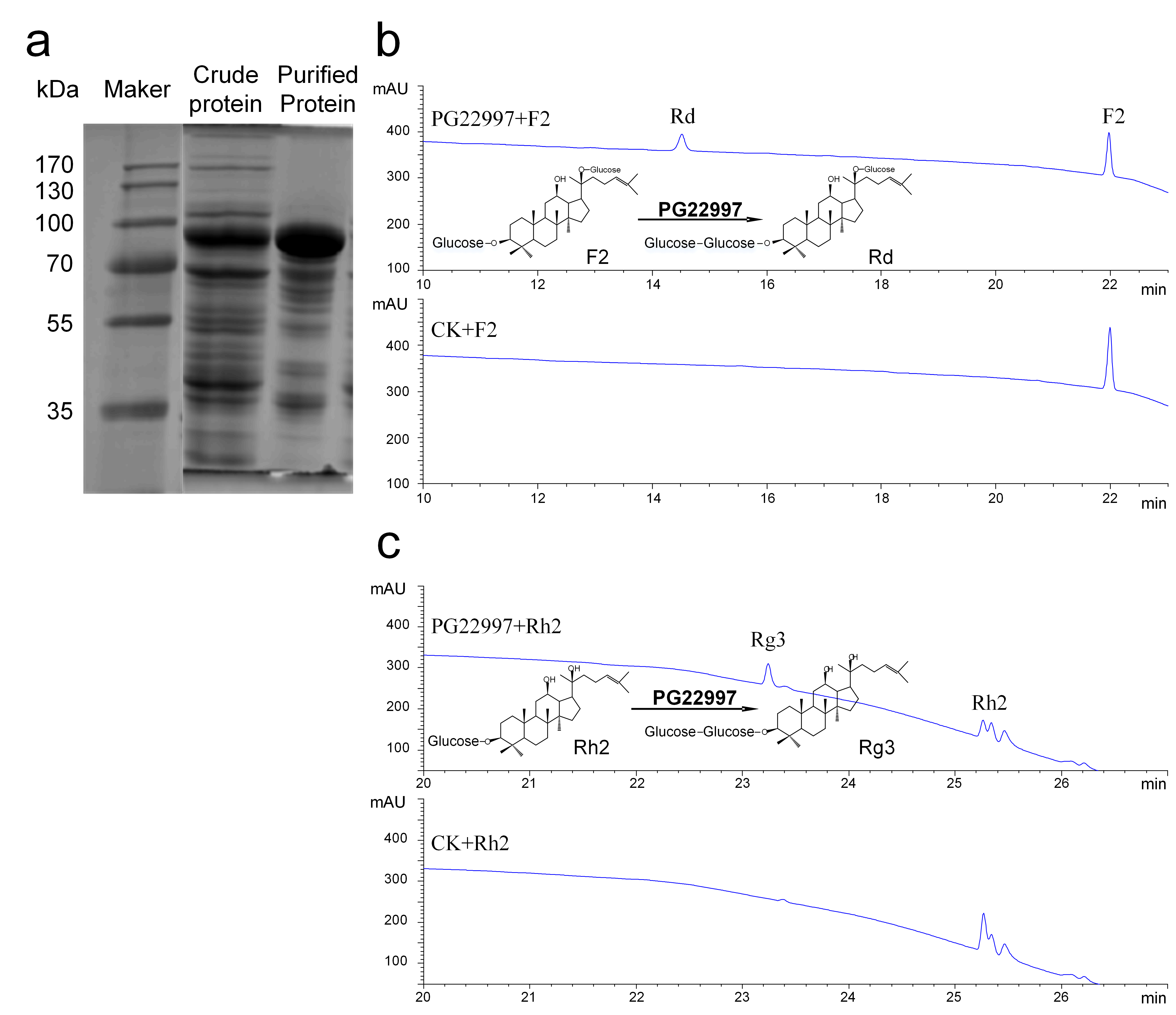


**Figure S13. The functional assay of this gene PG22997. (a)** The electrophoretogram of SDS-PAGE for recombinant PG22997. **(b, c)** The reactions that PG22997 catalyzes the ginsenoside Rf2 to ginsenoside Rd and ginsenoside Rh2 to ginsenoside Rg3.
