# Supplementary material for: Panax ginseng genome examination for ginsenoside biosynthesis
Source: Gigascience. 2017 Oct 5;6(11):1–15. doi: 10.1093/gigascience/gix093 (PMC5710592; doi:10.1093/gigascience/gix093)
Supplement: Supplement Tables and Figures [file gix093_supp.zip › Supplementary Tables R2.docx]

**Table S1. Statistics of *P. ginseng* genome sequencing.**

| **Insert size (bp)** | **Estimated insert size(bp)*** | **Raw Data** | | **Clean Data** | |  |
| --- | --- | --- | --- | --- | --- | --- |
|  |  | **Total data (Gb)** | **Sequence depth (X)** | **Total data (Gb)** | **Sequence depth**  **(X)** | |
| **250** | 248 | 93.73 | 26.93 | 90.65 | 26.05 | |
| **500** | 518 | 97.68 | 28.07 | 93.17 | 26.77 | |
| **2,000** | 1585 | 58.39 | 16.78 | 49.57 | 14.24 | |
| **5,000** | 3853 | 80.39 | 23.10 | 45.29 | 13.01 | |
| **10,000** | 7512 | 61.27 | 17.61 | 37.25 | 10.70 | |

***** Estimated insert size was calculated using alignments(.bam file) between reads and genome assembly.

**Table S2. Statistics of P. ginseng transcriptome sequencing.**

| Sample | Total data | Read length | Number of reads | Base pair(bp) | Q20 of reads | Q30 of reads | %GC |
| --- | --- | --- | --- | --- | --- | --- | --- |
| WGC093423-2M | 8.94G | 150 | 125,011,556 | 9,375,866,700 | 97.38% | 93.47% | 43 |
| WGC093424-6M | 8.25G | 150 | 115,291,028 | 8,646,827,100 | 97.53% | 93.85% | 43 |
| WGC093424-7M | 9.67G | 150 | 135,254,796 | 10,144,109,700 | 97.67% | 94.11% | 43 |
| WGC093423-2R | 8.16G | 150 | 114,121,468 | 8,559,110,100 | 97.39% | 93.46% | 43 |
| WGC093424-6R | 9.10G | 150 | 127,122,332 | 9,534,174,900 | 97.67% | 94.11% | 43 |
| WGC093424-7R | 9.38G | 150 | 131,115,496 | 9,833,662,200 | 97.62% | 94.00% | 43 |
| WGC093423-2Z | 9.21G | 150 | 128,732,628 | 9,654,947,100 | 97.45% | 93.58% | 43 |
| WGC093424-6Z | 9.33G | 150 | 130,506,240 | 9,787,968,000 | 97.58% | 93.97% | 43 |
| WGC093424-7Z | 9.77G | 150 | 136,564,660 | 10,242,349,500 | 97.30% | 93.38% | 43 |

**Table S3. Statistics of 17-mer analysis.**

| **Sample** | **Kmer** | **Kmer number** | **Peak depth** | **Genome size (bp)** | **Used bases** | **Used read number** | **Depth** |
| --- | --- | --- | --- | --- | --- | --- | --- |
| ***Panax ginseng*** | 17 | 163,927,969,288 | 47 | 3,487,829,133 | 183,819,034,360 | 1,243,191,567 | 52.7 |

**Table S4. Predicting the repetitive elements.**

| **Type** | **Repeat Size (bp)** | **% of genome** |
| --- | --- | --- |
| **TRF** | 162,525,961 | 4.74 |
| **Repeatmasker** | 453,238,472 | 13.20 |
| **Proteinmask** | 517,528,112 | 15.08 |
| **De novo** | 1,784,418,350 | 51.40 |
| **Total** | 2,133,957,634 | 62.17 |

1, The TRF was used to detect tandem repeats.

2, RepeatMasker was first used to predict de novo repeat elements in ginseng genome.

3, RepeatProteinMask was used to mask TE proteins.

4, RepeatMasker was used to identify the repeat in genome according to the de novo identification of repeat elements with RepeatModeler, PILER, LTR_FINDER and RepeatScout.

5, Total repeat regions were identified by combing all the repeat identified. For there are overlaps between different methods, the total region is shorter than the sum of repeats identified by all the methods.

| **Type** | **Repbase TEs** | | **TE proteins** | | **De novo** | | **Combined TEs** | |
| --- | --- | --- | --- | --- | --- | --- | --- | --- |
|  | **Length (bp)** | **% in genome** | **Length (bp)** | **% in genome** | **Length (bp)** | **% in genome** | **Length (bp)** | **% in genome** |
| **DNA** | 29,698,774 | 0.87 | 18,330,960 | 0.53 | 67,211,061 | 1.96 | 96,449,994 | 2.81 |
| **LINE** | 5,585,003 | 0.16 | 5,167,509 | 0.15 | 11,360,671 | 0.33 | 20,022,058 | 0.58 |
| **SINE** | 59,825 | 0.00 | 0 | 0.00 | 153,335 | 0.00 | 214,630 | 0.01 |
| **LTR** | 417,856,926 | 12.17 | 494,029,262 | 14.39 | 1,672,551,554 | 48.73 | 1,781,564,786 | 51.91 |
| **Other** | 37,944 | 0.00 | 381 | 0.00 | 5,525,921 | 0.16 | 38,476 | 0.00 |
| **Unknown** | 0 | 0.00 | 0 | 0.00 | 27,615,808 | 0.80 | 27,615,808 | 0.80 |
| **Total** | 453,238,472 | 13.20 | 517,528,112 | 15.08 | 1,784,418,350 | 51.99 | 1,925,905,752 | 56.11 |

**Table S5. Categories of TEs predicted in the ginseng genome.**

**Table S6. Details of repetitive elements in ginseng genome.**

| **Repeat Categories** | | | **Length (bp)** | **% of repeats** | **% of**  **assembled genome** |
| --- | --- | --- | --- | --- | --- |
| Total repeat fraction | | |  | 100 |  |
| Mobile Element | | |  |  |  |
|  | **Class I: Retroelement** | |  |  |  |
|  |  | LTR Retrotransposon |  |  |  |
|  |  | Ty1/Copia | 284,237,263 | 13.32 | 8.28 |
|  |  | Ty3/Gypsy | 1,469,576,948 | 68.87 | 42.82 |
|  |  | Other | 27,750,575 | 1.30 | 0.81 |
|  |  | non-LTR Retrotransposon |  |  |  |
|  |  | LINE | 20,022,058 | 0.94 | 0.58 |
|  |  | SINE | 214,630 | 0.01 | 0.01 |
|  |  | unclassified retroelement | 38,476 | 0.00 | 0.00 |
|  | **Class II: DNA Transposon** | |  |  |  |
|  |  | hAT | 17,064,802 | 0.80 | 0.50 |
|  |  | P | 1,808,992 | 0.08 | 0.05 |
|  |  | *PIF* | 3,122,527 | 0.15 | 0.09 |
|  |  | *PiggyBac* | 114,167 | 0.01 | 0.00 |
|  |  | Crypton | 480,876 | 0.02 | 0.01 |
|  |  | Helitron | 4,099,534 | 0.19 | 0.12 |
|  |  | Maverick | 1,446,967 | 0.07 | 0.04 |
|  |  | *CMC* | 43,226,842 | 2.03 | 1.26 |
|  |  | *MULE* | 18,006,951 | 0.84 | 0.52 |
|  |  | *Others* | 7,078,336 | 0.33 | 0.21 |
|  | **Tandem Repeats** | | 199,051,882 | 8.03 | 5.80 |
|  | **Unknown** | | 27,615,808 | 1.32 | 0.80 |

**Table S7. The transcription factors.**

| Order | Transcription Factors | Number |
| --- | --- | --- |
| 1 | AP2 | 52 |
| 2 | ARF | 28 |
| 3 | ARR-B | 14 |
| 4 | B3 | 46 |
| 5 | BBR-BPC | 18 |
| 6 | BES1 | 15 |
| 7 | bHLH | 208 |
| 8 | bZIP | 121 |
| 9 | C2H2 | 88 |
| 10 | C3H | 86 |
| 11 | CAMTA | 9 |
| 12 | CO-like | 19 |
| 13 | CPP | 10 |
| 14 | DBB | 14 |
| 15 | Dof | 54 |
| 16 | E2F/DP | 15 |
| 17 | EIL | 3 |
| 18 | ERF | 213 |
| 19 | FAR1 | 40 |
| 20 | G2-like | 55 |
| 21 | GATA | 45 |
| 22 | GeBP | 2 |
| 23 | GRAS | 76 |
| 24 | GRF | 20 |
| 25 | HB-other | 21 |
| 26 | HB-PHD | 2 |
| 27 | HD-ZIP | 90 |
| 28 | HRT-like | 3 |
| 29 | HSF | 44 |
| 30 | LBD | 80 |
| 31 | LFY | 2 |
| 32 | LSD | 10 |
| 33 | MIKC_MADS | 38 |
| 34 | M-type_MADS | 76 |
| 35 | MYB | 166 |
| 36 | MYB_related | 142 |
| 37 | NAC | 172 |
| 38 | NF-X1 | 4 |
| 39 | NF-YA | 14 |
| 40 | NF-YB | 27 |
| 41 | NF-YC | 15 |
| 42 | Nin-like | 19 |
| 43 | RAV | 4 |
| 44 | S1Fa-like | 4 |
| 45 | SAP | 2 |
| 46 | SBP | 49 |
| 47 | SRS | 14 |
| 48 | STAT | 2 |
| 49 | TALE | 40 |
| 50 | TCP | 34 |
| 51 | Trihelix | 59 |
| 52 | VOZ | 7 |
| 53 | Whirly | 3 |
| 54 | WOX | 30 |
| 55 | WRKY | 102 |
| 56 | YABBY | 2 |
| 57 | ZF-HD | 28 |
|  | Total | 2556 |

**Table S8. The transporter superfamilies.**

| Order | Transporter superfamilies | Number |
| --- | --- | --- |
| 1 | Protein Kinase (PK) Superfamily | 484 |
| 2 | ABC1, ABC2, ABC3 Superfamilies | 331 |
| 3 | MFS Superfamily | 295 |
| 4 | APC Superfamily | 230 |
| 5 | Drug/Metabolite Transporter (DMT) Superfamily | 128 |
| 6 | P-type ATPase (P-ATPase) Superfamily | 120 |
| 7 | Na+ Transporting Mrp Superfamily | 98 |
| 8 | VIC Superfamily | 92 |
| 9 | Mitochondrial Carrier (MC) Superfamily | 91 |
| 10 | Major Intrinsic Protein (MIP) Superfamily | 83 |
| 11 | Endomembrane Protein-Translocon (EMPT) Superfamily | 71 |
| 12 | Glycosyl Transferase/Transporter (GTT) Superfamily | 66 |
| 13 | Peroxisomal Peroxin (Pex)11/25/27 (Pex11/25/27) Superfamily | 59 |
| 14 | Multidrug/Oligosaccharidyl-lipid/Polysaccharide (MOP) Flippase Superfamily | 52 |
| 15 | CPA Superfamily | 51 |
| 16 | Cation Diffusion Facilitator (CDF) Superfamily | 44 |
| 17 | Transporter-Opsin-G protein-coupled receptor (TOG) Superfamily | 34 |
| 18 | IT Superfamily | 30 |
| 19 | Outer Membrane Pore-forming Protein (OMPP) Superfamily I | 28 |
| 20 | BART Superfamily | 27 |
| 21 | MACPF Superfamily | 19 |
| 22 | Resistance-Nodulation-Cell Division (RND) Superfamily | 12 |
| 23 | Outer Membrane Pore-forming Protein (OMPP) Superfamily IV (Tim17/OEP16/PxMPL (TOP) Superfamily) | 10 |
| 24 | Cytochrome b561 (Cytb561) superfamily | 7 |
| 25 | LysE Superfamily | 7 |
| 26 | CAAX Superfamily | 6 |
| 27 | Outer Membrane Pore-forming Protein (OMPP) Superfamily III | 5 |
| 28 | PTS-GFL Superfamily | 2 |
| 29 | Tail-anchored Membrane Protein Insertase (TAMP-I) Superfamily | 2 |
| 30 | ArsA ATPase (ArsA) Superfamily | 1 |
| 31 | Copper Resistance Superfamily | 1 |
| 32 | Tetraspan Junctional Complex Protein (4JC) Superfamily | 1 |
| 33 | Others | 1258 |
|  | Total | 3745 |

**Table S9. Genome list used for phylogeny estimation.**

| **Species** | **Taxonamy** | | **File Name** | **URL** | |  |
| --- | --- | --- | --- | --- | --- | --- |
| ***Daucus carota*** | | Eudicots;Apiales;Apiaceae | GCF_001625215.1_ASM162521v1_protein.faa.gz | | ftp://ftp.ncbi.nlm.nih.gov | |
| ***Panax ginseng*** | | Eudicots;Apiales;Araliaceae | Ginseng.all.maker.proteins.fasta | | — | |
| ***Arabidopsis thaliana*** | | Eudicots;Brassicales;Brassicaceae | Athaliana_167_TAIR10.protein.fa.gz | | https://phytozome.jgi.doe.gov/pz/portal.html# | |
| ***Castanea sativa*** | | Eudicots;Fagales;Fagaceae | Csativus_122_v1.0.protein.fa.gz | | https://phytozome.jgi.doe.gov/pz/portal.html# | |
| ***Coffea canephora*** | | Eudicots;Gentianales;Rubiaceae | coffea_pep.faa.gz | | http://coffee-genome.org/sites/coffee-genome.org | |
| ***Ricinus communis*** | | Eudicots;Malpighiales;Euphorbiaceae | Rcommunis_119_v0.1.protein.fa.gz | | https://phytozome.jgi.doe.gov/pz/portal.html# | |
| ***Populus trichocarpa*** | | Eudicots;Malpighiales;Salicaceae | Ptrichocarpa_210_v3.0.protein.fa.gz | | https://phytozome.jgi.doe.gov/pz/portal.html# | |
| ***Theobroma cacao*** | | Eudicots;Malvales;Malvaceae | Tcacao_233_v1.1.protein.fa.gz (multi-isoform) | | https://phytozome.jgi.doe.gov/pz/portal.html# | |
| ***Eucalyptus grandis*** | | Eudicots;Myrtales;Myrtaceae | Egrandis_297_v2.0.protein.fa.gz | | https://phytozome.jgi.doe.gov/pz/portal.html# | |
| ***Prunus persica*** | | Eudicots;Rosales;Rosaceae | Ppersica_298_v2.1.protein.fa.gz | | https://phytozome.jgi.doe.gov/pz/portal.html# | |
| ***Citrus sinensis*** | | Eudicots;Sapindales;Rutaceae | Csinensis_154_v1.1.protein.fa.gz | | https://phytozome.jgi.doe.gov/pz/portal.html# | |
| ***Solanum lycopersicum*** | | Eudicots;Solanales;Solanaceae | Slycopersicum_225_iTAGv2.3.protein.fa.gz | | https://phytozome.jgi.doe.gov/pz/portal.html# | |
| ***Vitis vinifera*** | | Eudicots;Vitales;Vitaceae | Vvinifera_145_Genoscope.12X.protein.fa.gz | | https://phytozome.jgi.doe.gov/pz/portal.html# | |
| ***Oryza sativa*** | | Monocots;Poales;Poaceae | Osativa_323_v7.0.protein.fa.gz | | https://phytozome.jgi.doe.gov/pz/portal.html# | |

**Table S10. Identification of ginsenosides.**

| **Name** | **Molecular formula** | | **Theoretical monoisotopic mass**  **(Da)** | | **Adduct** | | **Theoretical *m/z*** | | **Observed *m/z*** | | **Mass difference**  **(ppm)** | |
| --- | --- | --- | --- | --- | --- | --- | --- | --- | --- | --- | --- | --- |
| **Maltose** | | C_12_H_22_O_11_ | | 342.1162 | | M+Cl | | 377.0856 | | 377.0845 | | -2.9 |
| **Citbismine C** | | C_37_H_36_N_2_O_11_ | | 684.2319 | | M-H | | 683.2246 | | 683.2243 | | -0.4 |
| **Ginsenoside Rf/Rg1** | | C_42_H_72_O_14_ | | 800.4922 | | M+Cl | | 835.4616 | | 835.4586 | | -3.6 |
| **Ginsenoside Rd/Re** | | C_48_H_82_O_18_ | | 946.5501 | | M+Cl | | 981.5195 | | 981.5168 | | -2.8 |
| **Pseudoginsenoside Rc1** | | C_50_H_84_O_19_ | | 988.5607 | | M-H | | 987.5534 | | 987.5521 | | -1.3 |
| **Ginsenoside Rs1/Rs2** | | C_55_H_92_O_23_ | | 1120.6029 | | M-H | | 1119.5957 | | 1119.5933 | | -2.1 |
| **Ginsenoside Ra1/Ra2** | | C_58_H_98_O_26_ | | 1210.6346 | | M+Cl | | 1245.6062 | | 1245.6060 | | -0.2 |
| **Ginsenoside Ra3** | | C_59_H_100_O_27_ | | 1240.6452 | | M+Cl | | 1275.6146 | | 1275.6178 | | 2.5 |

**Table S11. Ginsenosides concentrations in different tissues (peridem, cortex and stele) of roots.**

| **Ginsenoside** | **Periderm** | **Cortex** | **Stele** | **P value** | **FDR** |
| --- | --- | --- | --- | --- | --- |
| **Rg1** | 2.03±0.72^a^ | 0.77±0.55^b^ | 0.31±0.14^b^ | 1.87E-02 | 1.87E-02 |
| **Re** | 5.91±0.92^a^ | 0.12±0.07^b^ | 0.11±0.04^b^ | 1.54E-05 | 6.16E-05 |
| **Rf** | 1.44±0.45^a^ | 0.32±0.16^b^ | 0.19±0.05^b^ | 2.57E-03 | 2.94E-03 |
| **Rg2** | 1.56±0.18^a^ | 0.06±0.05^b^ | 0.07±0.06^b^ | 4.59E-06 | 3.67E-05 |
| **Rb1** | 2.97±0.50^a^ | 0.55±0.37^b^ | 0.18±0.06^b^ | 1.47E-04 | 2.94E-04 |
| **Rc** | 2.54±0.54^a^ | 0.22±0.08^b^ | 0.14±0.01^b^ | 1.34E-04 | 2.94E-04 |
| **Rb2** | 2.24±0.56^a^ | 0.17±0.06^b^ | 0.11±0.01^b^ | 3.12E-04 | 4.99E-04 |
| **Rd** | 0.56±0.12^a^ | 0.20±0.05^b^ | 0.17±0.01^b^ | 1.17E-03 | 1.56E-03 |

The results are expressed as mean ± SD of 3 samples per group. Statistical comparison was performed using one-way analysis of variance (ANOVA) and post hoc LSD multiple comparison tests at a level of significance P<0.05.

**Table S12. Alignment Score of HMGRs by CLUSTALW.**

|  | PgHMGR1 | PG16235 | PG37498 | PG00233 | PgHMGR2 | PG03840 | PG07131 | PG38245 |
| --- | --- | --- | --- | --- | --- | --- | --- | --- |
| PG16235 | 100.0000 | — | — | — | — | — | — | — |
| PG37498 | 98.6038 | 98.6038 | — | — | — | — | — | — |
| PG00233 | 89.9115 | 89.9115 | 88.8496 | — | — | — | — | — |
| PG15732 | 88.4956 | 88.4956 | 87.7876 | 96.8142 | — | — | — | — |
| PG03840 | — | — | — | — | 99.6633 | — | — | — |
| PG07131 | — | — | — | — | 97.8114 | 98.1481 | — | — |
| PG38245 | — | — | — | — | 87.4363 | 87.7759 | 88.2852 | — |
| PG02251 | — | — | — | — | 88.1154 | 88.4550 | 88.9643 | 98.6418 |

**Table S13. HMGRs location prediction features analyzed by WoLF SPORT.**

|  | E.R. | Chlo | Mito | Chlo-Mito | Nucl | Cyto | Plas | Cysk |
| --- | --- | --- | --- | --- | --- | --- | --- | --- |
| PgHMGR1 | 2 | 2.5 | 2.5 | 3 | 2 | 2 | 1 | 1 |
| PG16235 | 2 | 2.5 | 2.5 | 3 | 2 | 2 | 1 | 1 |
| PG37498 | 7 | — | 1.5 | 1.5 | — | — | 5 | — |
| PG00233 | 3 | 5.5 | 1.5 | 4 | 3 | — | — | — |
| PG15732 | 3 | 5.5 | — | 3.5 | 3 | 1 | — | — |
| PgHMGR2 | — | 5.5 | 5.5 | 6 | 1 | 1 | — | — |
| PG03840 | — | 5.5 | 5.5 | 6 | 1 | 1 | — | — |
| PG07131 | 1 | 6 | 5 | — | 1 | 1 | — | — |
| PG02251 | 1 | 8 | 3 | — | 1 | 1 | — | — |
| PG38245 | 1 | 7 | 3 | — | 1 | 2 | — | — |
| AtHMGR1 | — | 4.5 | 4.5 | 5 | 2 | — | 2 | — |
| AtHMGR2 | 6 | 1 | — | — | 1 | 5 | — | — |

E.R., endoplasmic reticulum; Chlo, chloroplast; Mito, mitochondria; Chlo-Mito, chloroplast- mitochondria; Nucl, nucleus; Cyto, cytolysosome; Plas, plastid; Cysk, cytoskeleton.

|  | **Leaf blade** | **Fruit pedicel** | **Stem** | **Fruit flesh** | **Leaf peduncle** | **Leaflet pedicel** | **Arm root** | **Rhizome** | **Seed** | **CV (%)** | **Extrem deviation** |
| --- | --- | --- | --- | --- | --- | --- | --- | --- | --- | --- | --- |
| **PG37498** | 287.43 | 789.68 | 416.25 | 988.88 | 415.14 | 992.67 | 751.90 | 508.98 | 3002.41 | 1.269 | 2714.98 |
| **PG16235** | 156.30 | 416.31 | 99.46 | 534.52 | 244.59 | 411.30 | 560.10 | 384.52 | 629.47 | 47.93 | 30.015 |
| **PG15732** | 10.33 | 67.59 | 41.56 | 75.88 | 56.81 | 46.96 | 97.21 | 141.13 | 436.13 | 118.67 | 425.80 |
| **PG00233** | 151.75 | 273.23 | 204.79 | 380.14 | 207.08 | 266.20 | 232.95 | 320.14 | 912.18 | 70.00 | 760.43 |
| **PG07131** | 10.43 | 27.26 | 8.90 | 9269.38 | 23.70 | 21.34 | 7.07 | 47.63 | 13983.70 | 201.99 | 13976.63 |
| **PG03840** | 15.64 | 18.17 | 8.90 | 2915.01 | 7.90 | 53.34 | 0.00 | 114.40 | 3079.94 | 189.63 | 3079.94 |
| **PG38245** | 3672.10 | 92.21 | 48.69 | 60.07 | 88.19 | 291.95 | 1538.90 | 290.691 | 41.92 | 154.63 | 363018 |
| **PG02251** | 1969.84 | 270.34 | 212.81 | 72.42 | 479.23 | 299.97 | 1456.79 | 1311.95 | 1311.95 | 104.03 | 1921.63 |

**Table S14. PgHMGR family expression in different tissues.**
