# Supplementary material for: Panax ginseng genome examination for ginsenoside biosynthesis
Source: Gigascience. 2017 Oct 5;6(11):1–15. doi: 10.1093/gigascience/gix093 (PMC5710592; doi:10.1093/gigascience/gix093)
Supplement: Supplement Tables and Figures [file gix093_supp.zip › Supplementary Text R1.docx]

The following is the configuration for SSPACE in the scaffolding process:

Lib1 bowtie /path/to/Ginseng_2000bp.trim.R1.fastq.gz /path/to/Ginseng_2000bp-trim.R2.fastq.gz 5000 0.5 RF

Lib2 bowtie /path/to/Ginseng_5000bp.trim.R1.fastq.gz /path/to/Ginseng_5000bp-trim.R2.fastq.gz 5000 0.5 RF

Lib3 bowtie /path/to/Ginseng_10000bp.trim.R1.fastq.gz /path/to/Ginseng_10000bp-trim.R2.fastq.gz 5000 0.5 RF
