# Supplementary material for: Panax ginseng genome examination for ginsenoside biosynthesis
Source: Gigascience. 2017 Oct 5;6(11):1–15. doi: 10.1093/gigascience/gix093 (PMC5710592; doi:10.1093/gigascience/gix093)
Supplement: Response-to-Reviewer-Comments_Original-Submission.pdf [file gix093_response-to-reviewer-comments_original-submission.pdf]

Author's Response To Reviewer Comments

Dear Dr. Hans Zauner,

We thank you and the reviewers for your detailed comments, which really benefit our manuscript. We have carefully considered all comments and accordingly revised our manuscript. Please find below our point-by-point replies to the comments and detailed explanations of all changes ("V1" refers to the originally submitted version and "R1" is the revised version; all revisions in R1 were tracked). Thank you!

Reviewer #1: The manuscript entitled: "Ginseng genome examination for ginsenoside biosynthesis" by Xu Jiang et al., presents results of sequencing, characterization and annotation of the P. ginseng genome. The authors have further shown the distribution of the ginsenosides molecules in root tissues using mass spectrometry and liquid chromatography, and in order to identify candidate genes possible involved in the ginsenosides biosynthesis pathway an analysis of expression and co-expression networks using RNA-seq data was performed. A set of key genes involved in the ginsenosides biosynthesis were further characterized and interestingly some of them showed tissue specificity and activity during microbial infection. Overall, the experiments and analysis for this study were well conducted. And the results presented in this manuscript are important for the research of its field. However, the manuscript presents major faults that need to be addressed prior publication.
Reply: Thank you for your positive assessment. We have revised our work according to the comments.

Major comments
1. How old were the P. ginseng plants used for genome sequencing?
Reply: The ginseng sample we used for genome sequencing came from a 4-year-old P. ginseng plant. We have added this information in the method section of R1.

2. The Microbial-resistance section needs to be greatly improved since there is a lot of missing information:
Reply: Thank you for pointing out this deficiency. In V1, we used published data (Gao et al. 2016) to recalculate gene expression during fungus infection. Although it is important for ginseng cultivation, this section is still slightly correlated with other parts of the manuscript. Considering the two reviewers' advice, the microbial-resistance section has been removed in R1. Nevertheless, we have attempted to answer the questions below as best as we can. We are truly grateful for the reviewer's comments and suggestions.

a. The statement "Ginsenosides comprise a group of defense metabolites" needs to be supported by a reference.
Reply: Thank you for pointing this out. Indeed, this statement should be supported by some references to increase readers' understanding. Triterpenoid saponins are considered as defense compounds against pathogenic microbes and herbivores (Osbourn, 1996; reviewed by Augustin et al., 2011). A recent study also reveals that unigenes involved in ginsenoside biosynthesis play important roles in the response to Cylindrocarpon destructans infection (Gao et al., 2016).

b. What does the authors mean in the statement: "responses to microbial infection are important for ginsenoside biosynthesis"? Is the ginsenosides biosynthesis pathway lead by defense responses of the plant? Does this mean that if the plant is not under biotic stress the ginsenosides are not produced? What happen with the ginsenosides biosynthesis during C. destructans infection? Does the ginsenosides increase? Please clarify and support the statement above with a scientific reference.
Reply: Thank you for the reviewer's concerns. We obtained this viewpoint from two aspects. First, the ginsenoside biosynthesis pathway may be activated by microbial infection, and ginsenosides reportedly accumulate during C. destructans infection (Chi et al., 2016). Ginsenosides also increase after eliciting SA (Tewari et al., 2011) and MeJA (Lim et al., 2005; Lee et al., 2017). Second, some reports suppose that ginsenosides are phytoalexins and may be the evolution products for microbial resistance (Gao et al., 2016; Hu et al., 2007; Kim et al., 2015). Although we have removed this part in R1, clarifying this statement in future work is still important.

c. The authors mentioned that 1652 resistance genes were annotated base on "plant resistance gene database". Please add the reference for this database.
Reply: Thank you for pointing this out. The Plant Resistance Genes database (PRGdb; http://prgdb.org) is a comprehensive resource of resistance genes (R-genes), a major class of genes in plant genomes that conveys disease resistance against pathogens (Sanseverino et al., 2010).

d. The last paragraph on page 15 starting at "The expression analysis showed that 160 resistance genes …" till the end, it is not supported by any figure or table and it is not clear what expression analysis were performed. (FPKM?, WGCNA?, qPCR?)
Reply: We apologize for the confusion caused by this paragraph. We used a FPKM analysis result here.

e. The first paragraph on page 16 "The transcriptomes of ginseng induced by C. destructants … were recalculated". What does the authors mean by "transcriptomes were recalculated"? Did the authors sequence RNA of infected plants? All this information is missing in the methods section.
Reply: We regret the unclear description. We used the published transcriptome read (Gao et al., 2016) mapping of our genome sequence to recalculate gene expression (FPKM value) during fungus infection. Thus, we did not sequence the RNA of infected plants by ourselves, and information was not described in detail in the method section.

f. In the next statement, starting at "A total of 35008 genes were predicted, of these genes 28481 were expressed … the initial point" has to be supported by evidence. There is not figure or table that shows this analysis. How did the authors get the genes? Did they perform an RNA-seq assembly? If it so, where are the methods and metrics of the RNA-seq assembly?
Reply: We apologize for the confusion. A total of 35 008 genes were identified using time-course RNA-seq data by Gao et al. (2016) As we stated in our reply to Question e, the RNA data were from a published paper (Gao et al., 2016). To avoid misunderstanding, we have deleted this section.

g. The statement "At 0.25 DPI, 104 resistance genes were highly expressed and were possibly

involved in C. destructans recognition" needs to be supported by evidence and a reference. The level of gene expression might not be related with the pathogen recognition at all.
Reply: We completely agree with the reviewer's point. The resistance-gene expression level may be influenced by many factors, including fungus infection.

h. The next paragraph starting at "At 0.25 DPI" till "Figure S10" describes a GO enrichment test described in figure S10. However figure S10 does not show what described in the text.
Reply: We regret the unclear description. Considering the weak correlation between this microbial-resistance section and the other parts of the manuscript, we have removed this section including the GO enrichment test and Fig. S10 in R1.

i. The entire section is not well described or described at all in methods.
Reply: We agree with the reviewer's point. The other reviewer also pointed that this section is weakly related to the other parts of the manuscript. Considering both reviewers' suggestions, we have deleted the entire microbial-resistance section in R1.

1. The methods and tools in methods sections are not properly cited. For example, BLAST, WGCNA, R, etc., lack of reference. Databases such as GO, KEGG, KOG, etc., are not properly cited. The authors should cite the scientific manuscript of the tools and databases and not only the website.
Reply: Thank you for noticing these points that we ignored in V1. We have accordingly corrected these errors and added appropriate references to R1. For example, BLAST (Altschul et al., 1990), WGCNA (Langfelder and Horvath, 2008), GO (Ashburner et al., 2000), KEGG (Kanehisa et al., 2003), and KOG (Koonin et al., 2004) have been cited in R1.

2. Repeat prediction, gene prediction and annotation section in Methods. Page 26. The authors mention that transcripts assembled from RNA-seq data were use for MAKER-P annotation. However there is none information regarding to RNA-seq assembly. What assembler was used? What parameters were used? How many transcripts were obtained? Etc.,The metrics of the transcriptome assembly are not described at all.
Reply: We regret not describing this assembly step in detail. Thank you for pointing this out. We conducted RNA-seq assembly using Trinity software with default parameters and generated 75 878 transcripts, which were used for further gene annotation as RNA evidence. The total length of 75 878 transcripts was 70 273 566 bp, max, min, and the N50 lengths were 12 639, 201, and 1446 bp, respectively.

Minor comments
Page 7. Missing space: "RNA-seqlibraries"
Reply: Thank you for pointing this out. We have revised this fault in line 10, page 7 in R1.

Page 7. Please add the reference for "13 published ginseng RNA-seq data".
Reply: Thank you. The 13 published ginseng RNA-seq data came from two references (Gao et al., 2016; Wang et al., 2015). In R1, we deleted all seven RNA-seq datasets from Gao et al. (2016) and added another two datasets from Wang et al. (2015).

Page 13. Figure 5. Please rename figures in order of citation. (5a, 5d, 5c, 5b -> 5a, 5b, 5c, 5d)

Reply: Thank you for this kind suggestion. We have revised the order of Fig. 5 in R1.

Page 14. The sentence "The expression patterns of different HMGR types differed" does not make sense. Please re-phrase.
Reply: Thank you for pointing out this error. We have replaced this sentence with "The expression patterns of dissimilar HMGR types differed among various organs" in line 13, page 14 in R1.

Page 20. Add the figure number that support this statement: "same PgHMGR subfamily, they exhibited different correlation patterns with other genes"
Reply: This statement was based on the results shown in Figs. 3b (R1) and 6b (V1). However, this sentence that was related to C. destructan infection has been deleted in R1.

Page 22. Genome sequencing and assembly. How old were the plants used in this study?
Reply: We used a 4-year-old P. ginseng plant in this study. The information has been added in the method section in R1.

Page 22. Please add the scientific reference for SOAPdenovo assembler.
Reply: Thank you for your suggestion. We have added the reference for SOAPdenovo2 assembler (Luo et al., 2012) as Reference 56 in R1.

Page 23. Last row of the first paragraph. There is missing information for the cutoff criteria. "BLAStN, a cutoff value of 90%". What is this 90%? Identity?
Reply: We meant an identity cutoff value of 90% and have clarified our meaning as "BLASTN, an identity cutoff value of 90%, and a coverage cutoff value of 90%" in lines 13–14, page 21.

Page 28. Please add the reference for PyMOL.
Reply: Thank you for this suggestion. We have added the reference for PyMOL (Seeliger and Groot, 2010) as Reference 89 in R1.

Figures:
Figure 5b. What does it show? FPKM values? Is it an qPCR? Y axis needs a label. In the figure legend the figure was described as a qPCR analysis however nothing of this is described in methods and primers sequences are not provide.
Reply: Fig. 5b shows the FPKM values. The Y-axis-label has been added to Fig. 5b in R1.

Figure S10. The legend of this figure does not provide enough information of the figure.
Reply: Thank you for pointing this out. As answered previously, we have deleted Fig. S10.

Reviewer #2: Xu Jiang et al., present a work on genome assembly and annotation of Panax ginseng, with subsequent rna-SEQ analysis and metabolite analysis of root tissues to advance the understanding of the ginsenosides' synthesis pathway. All in all this project is interesting and the current work will merit publication once a number of major comments are resolved. The text could also benefit from some re-organisation to remove duplication, and present the information and technical details in a more concise and ordered fashion. I will focus this review mainly on

the sequencing aspects of the paper, with special emphasis on the genome assembly which is my field of expertise.

I feel this paper could gain a lot by tightening up the methods. This will either provide more support for the ginsenoside biosynthesis analysis in which case the focus can highlight that result or if there is no more support for that analysis then the paper can be refocused on a stronger resource description about the genome assembly and differential expression dataset.
Reply: We sincerely appreciate the authoritative and constructive suggestions of Bernardo J. Clavijo and Luis Yanes. We have revised our work according to the comments.

Major comments
Experimental design, data description and availability:

1) Reads submitted to public archives should be raw, except only for demultiplexing. This is a prerequisite for reproducibility and traceability. All data that has been submitted trimmed or otherwise pre-processed must be resubmitted.
Reply: Thank you for pointing this out. We have already uploaded raw reads to NCBI, and here are the SRA accession numbers:
EXPERIMENT: PG10000 (SRX2955138),
EXPERIMENT: PG250 (SRX2955139),
EXPERIMENT: PG500 (SRX2955140),
EXPERIMENT: PG2000 (SRX2955141), and
EXPERIMENT: PG5000 (SRX2955142).

2) There is no description of the protocols used to generate the paired end data and long mate paired data for genome assembly. Specially with the LMP data, the reads seem untrimmed, but they do contain substantial amounts of short-insert contamination which may be better accounted for if the protocol is properly described.
Reply: We have added the information, including the library construction, on how to generate paired end data and long mate paired data in R1. We apologize for the unclear description in V1 about processing the LMP data. We used Skewer (Jiang et al., 2014) for adapter trimming and low-quality base filtering with these parameters. Transposase adapter sequences were used for adapter searching and trimming, and the maximum mismatch rate was set to 10%; trimming reads were from 3′ end until Q>20. After trimming, reads (2–10 kb) with read length <18 bp or average quality <30 were filtered out.

3) There is no justification for the choice of line IR826 over any other line, and no mention whether the independent samples for differential expression are from a single plant or different plants, and if those are from the IR826 line.
Reply: We had two reasons for selecting line IR826. First, the heterozygosity (1‰–5‰) of this line was relative low, which favored genome assembly. Second, we accumulated some horticultural data of IR826 in a previous study. As IR826 was the strain we used for field planting and was still in its test phase, the samples we used for tissue transcription profile were collected from different plants (some of which were not line IR826).

4) In general, including datasets from different studies for differential expression analysis is not

automatically reliable or even comparable. The use of external datasets for every tissue but root needs to be properly supported by analysis that shows statistical validity of equivalent background conditions, plus detailed description of similarities between experimental conditions. Also, the replicate structure of the samples should be the same. While this may be completely true, it is not discussed in the manuscript. If these criteria are not met, there should be explicit description of why this analysis is still considered valuable and its limitations.
Reply: We agree with the reviewer's suggestion. Analyzing new and previous datasets together was faulty. Thus, the transcription analysis of various organs was reformed with new datasets from a single study (Wang et al., 2015). The transcription profile of three different tissues in root was further analyzed for the expression patterns of different HMGR types.

Genome Assembly

5) There is no justification for the choice of assembler and no discussion about parameters (why that particular K, etc). Again, detailed description of the parametrisation for each tool is needed, with some justification when appropriate.
Reply: Thank you for this suggestion. We tested Kmer size using KmerGenie (Chikhi et al., 2014), and it recommended using an 83-mer to assemble. Then, we tried to assemble the Ginseng genome using SOAPdenovo2 and AbySS 1.9, but it ran out of memory after using AbySS 1.9. Consequently, we used 63-, 73-, 83-, and 93-mer to assemble SOAPdenovo2 and obtained the longest N50 when using 83-mer. This information has been added to manuscript R1 (lines 20–21, page 20; lines 1–2, page 21).

6) I analysed the assembly with a KAT spectra-CN plot (using k=31, only the short-insert libraries), with the following result:
This leads to a number of comments:

7.1) The kmer coverage at k=31 is around 20x using both PE libraries, which is lower that would be recommended to construct contigs (around 35x to 100x is probably best practice nowadays). I expect the coverage at the K used for assembly to be even lower.
Reply: Thank you for this technical suggestion. When we started this project, we followed previous genome sequencing projects and decided to sequence 30X depth for every shotgun library. However, after read trimming, the total depth was a bit lower than 30X. We also have noticed some new standards for genome assembly (Clavijo et al., 2017) and have decided to follow these standards in future work.

7.2) There is a loss of single-copy content (represented by the black distribution's peak at x=20) and generation of some content in the assembly not present on the reads (x=0). While this can be explained because of the gap closing and some N inclusion in the assembly, it merits some discussion and validation.
Reply: Thank you for pointing this out. To increase the efficiency of gene prediction, we have filtered fragmental scaffolds <1000 bp long. We performed KAT analysis on scaffolds before and after filtering with the 500 bp library. No significant content loss on unfiltered scaffolds ensued. Thus, we believe that the loss of single-copy content was due to length filtering.

7.3) There is duplication on the main single-copy distribution at x=20. This could explain some of the content loss (again, due to gap closing? analyses before and after gap closing would make this clear). At the same time, SOAPdenovo2's scaffolding introduces a single N between contigs when a negative distance between them is estimated by the scaffolding algorithm. This produces both unnecessary duplication of sequence and some misassembles. There are more than 350K instances of single Ns on the assembly and soap does not output Ns on the contigs, so this should be examined.
Reply: Thank you for pointing this out. We have performed KAT analysis at 7.2, we do not believe that a single Ns led to content loss.

8) The scaffolding method is slightly unorthodox, consisting of contig assembly and scaffolding with SOAPdenovo (I assume SOAPdenovo2, but it is not properly cited and no version is mentioned), then gap closing, then a further round of scaffolding with SSPACE. In general there are better and more modern methods to do this same work. Running an ABySS assembly end-to-end and comparing the results may be a good starting point.
Reply: Thank you for the suggestion. Yes, we used SOAPdenovo2, and the reference (Luo et al., 2012) has been added to R1. We also used ABySS 1.9 but had a large computer node with 1 TB memory only, and the ABySS assembly failed because of insufficient memory.

8.1) Re-scaffolding with the same datasets should not be needed, so I would suggest the authors either just use SOAP's contigs into SSPACE, or do not perform a second round of scaffolding. Performing gap closure before any scaffolding is also not recommended, so in any case this should be the last step in the assembly. As this assembly procedure does not follow best practices, I would have also expected to see a detailed description of the results at every step (i.e. contiguity, N content, etc). Also, the detailed configuration files and commands executed to produce the assemblies should be provided on the supplementary material and referred to in the main text (this is valid for all other analyses).
Reply: Thank you for the suggestion. We have unclearly described our assembly process and have accordingly made corrections. First, we used SOAPdenovo2 to assemble contigs and scaffolds with 250 and 500 bp short-insert libraries and 2000 bp long-mate-pair library. We then used SSPACE for scaffolding with 2K bp, 5K bp, and 10K bp long-mate-pair libraries. Afterwards, we used Gapcloser for gap closing using short libraries and obtain the final scaffolds. We have validated genome assemblies with LMP data, recalculated the mate-pair distance, and speculated that no significant overscaffolding occurred. We have added SSPACE configuration files into the Supplementary Text.

8.2) Any scaffolding such as this needs some validation, at the very least by analysing synteny with related species, and or by producing wet-lab validation of some junctions.
Reply: Yes, thank you for the suggestion. We have validated the junction through alignment with published P. ginseng BAC sequences. We observed good collinearity between the draft genome and BAC sequences as shown in the following figure.

8.3) We ran library fragment size analysis in the LMP libraries by mapping them to the assembly and found significant short-insert (pair-end orientation) contamination. Fragment-size distributions also changed if the assembly was split at N runs. Peaks at exactly the "nominal"

distances, specifically for the 10 Kbp library that was actually 7.5 Kbp, disappeared. This finding indicated some effect of overscaffolding. Performing this kind of analyses at every step of the assembly pipeline helped evaluate where biases were introduced.

8.4) The 10Kbp library is in fact 7.5Kbp so all references to that size should be updated. If 10Kbp has been used as size in software that does not correct for it automatically it may need to be re-run.
Reply: Thank you for pointing this out. We have run a validation test on size distribution on contigs and scaffolds. We mapped each LMP library read to contigs and scaffolds. We obtained a similar size distribution of each library between contigs and scaffolds. We speculated that small-insert-size fragments were easier to cyclize when constructing an LMP library. Similar size-distribution results showed no significant overscaffolding in scaffolds.

9) Transcript assembly mappability is shown as a metric of assembly reliability, but it is unclear that any de novo transcript assembly was performed. If that is the case, the de novo transcript assembly must be properly described and discussed.
Reply: We conducted RNA-seq assembly by using Trinity software with default parameters through one RNA-seq library and generated 75 878 transcripts (N50 length, 1446 bp; average sequence length, 926 bp), which were used for further gene annotation as RNA evidence. We have added these information to the manuscript.

10) The mapping of the 500bp library to assess non-bias is ad-hoc and I am not aware of methods that would support this (it also introduces mappability bias, etc, etc). For completeness I would prefer either metrics based on how well the original reads map back to the assembly or simply kmer spectra completeness (Disclaimer: I am the senior author of the KAT publication).
Reply: We have mapped 250 and 500 bp libraries to the draft genome using BWA mem with default parameters, which had 99.77% and 99.95% reads mapped. We also performed KAT analysis, and the result was 7.2. We found no significant content loss for unfiltered scaffolds.

RNA-seq and differential expression

11) The whole method of the transcript assembly, mapping support and numbers does not add up easily. A diagram of the annotation pipeline would clarify this, with numbers of transcripts, support for them, etc. This is a KEY point if this manuscript wants to present a resource for the community.
Reply: Thank you for pointing out this deficiency. Transcripts were de novo assembled with Trinity. Specifically, raw reads generated by RNA-seq of different parts of ginseng root were trimmed and quality controlled by skewers with the following parameters: 10% of the maximum mismatch rate, trimming reads from the 3′ end until Q>20, and trimmed reads with read length <100 bp or average quality <30 were filtered out. Thereafter, Trinity software with default parameters were used for de novo assembly. A total of 75 878 transcripts (N50 length, 1446bp; average sequence length, 926 bp) were assembled and used for further gene annotation as RNA evidence. The clean RNA-seq reads were aligned to the draft genome in the orientation mode through TopHat, and gene expression levels were calculated using FPKM value.

12) As mentioned in (4) the usage of dataset from previous studies needs more justification and support.
Reply: As we stated in in (4), analyzing new and previous datasets together was defective. Thus, the transcription analysis of various organs was reformed with new datasets from a single study (Wang et al., 2015). The transcription profile of three different tissues in root was analyzed in terms of the expression patterns of various HMGR types.

13) Figure 3e is unclear and probably also belongs in the supplementary.
Reply: Thank you for the suggestion. We have moved this figure to the supplementary.

14) Figure 4a shows "a possible pathway" but gives little extra detail or justification and it is not clear from the main text neither. I think this should be expanded and properly described in the main text.
Reply: Thank you for the suggestion. Fig. 4 shows that ginsenosides were mainly biosynthesized through the themevalonic acid (MVA) pathway utilizing the precursor IPP in the cytosol. This pathway is an ancestral metabolic route in all organisms, and eukaryotes have conserved enzymes from MVA to IPP synthesis. Given that the end product is IPP, this pathway is also accepted as the upstream pathway of triterpenoid saponins, most of which have been studied well. Thus, in the main text, we showed only our new discovery, i.e., that homologous enzymes were found in P. ginseng by BLAST and motif searching. If more detailed information is needed, Reference 52 (Kim et al., 2015) would be helpful.

15) Figure 4b shows an extremely different expression levels between root and all the other tissues. While this is expected and could be supportive of other claims in the manuscript, this also shows the division of new vs. pre-existent datasets. I think differential expression between the new datasets on this manuscript should become the main focus of this figure and the relevant section. If RNA-seq data for the other tissues can be regenerated and/or shown to be perfectly comparable for this purpose, then I would suggest using differential expression patterns to test the robustness of the proposed pathway membership among the proposed genes, and even maybe trying to find "missing members" of the pathway.
Reply: Thank you for the suggestion. As explained in Q4, we replotted the heatmaps using previous datasets and new datasets independently.

16) Figure 5b uses bars and SD to show 3-point datasets. This hides the real data and leads to a feeling of over-confidence in what is effectively a small-n case. Replace with a plot showing every point. The mean can be indicated within this plots with a line, but SD is mostly meaningless with N=3.
Reply: We appreciate the reviewer's advice. Showing small sample size data using a plot was appropriate. Thus, we have redrawn Fig. 5c, replacing the bars and SD with plots showing every point.

17) I can't finish to understand Figure 7, and I am conscious about the rna-seq dataset origin is playing a role here too.
Reply: We regret the unclear description in Fig. 7, especially Fig. 7b. We have added some information to Fig. 7 (V1)/Fig. 6 (R1) to make it clearer. The upper part of Fig. 6b (R1) is a

sketch of a UGT73 cluster; the line shows the cluster length and the arrows represent gene length and direction. The bottom part is the expression level of these genes, and we wanted to show that although these genes were in the same gene cluster and belonged to the same gene family, their expression patterns differed. Further study of these genes may benefit the biosynthesis analysis of ginsenosides. Thank you!

Pathway analysis

18) The first paragraph of this section is difficult to read, rewrite for clarity, please.
Reply: The MVA pathway is a conserved pathway for sterols or terpenoids synthesis. We have shortened the description of this pathway, which may have decreased the clarity of this paragraph. Accordingly, we have added a couple of references (References 12, 14, and 17) to this part and to increase understanding.

19) Copy number assessment of genes based on a WGS fragmented assembly as this one should at the very least be validated by using kmer coverage and/or read mapping depth. Moreover since there is a mention to putting together some of the genes manually because they were fragmented on the original assembly.
Reply: We have extracted the alignment information (.bam file) of the scaffold that contained the UGT gene cluster from full alignment (mapping 500 bp library reads to draft genome using BWA mem). We then calculated the mapping depth of this scaffold. Except for the gap, repeat region, or high-GC-content area, the mapping depth was around 20X.

Microbial resistance

20) I am not very sure how this section relates to the rest of the manuscript.
Reply: Thank you for the suggestion. Although it is important for ginseng cultivation, this section was still slightly correlated with other parts of the manuscript. Considering the two reviewers' advice, the microbial-resistance section has been removed in R1. We are truly grateful for the reviewer's comments and suggestions.

21) The seven-time-points experiment is a whole analysis on its own, same need for experimental design explanation and rationale as in previous sections apply.
Reply: We apologize for the confusion. These data were from a previously published paper (Gao et al., 2016) and contained limited explanation and rationale in V1.

22) The last sentence is too strong on its claim for the evidence presented and seems to have no experimental validation.
Reply: We apologize for including the statement without adequate evidence. This section has been accordingly deleted in R1.

Discussion

23) The claim for LTR content being significantly higher than previously thought is a huge one, and should be supported better starting by a proper statement of the methods used in both

studies. De novo methods for LTR content estimation exist that can give a figure without the bias of the assembly. If this claim is well supported it should feature more prominently, as it will probably change the understanding of the genome's history and evolution. Also, further dating of LTRs and extra analyses could help elucidate why the results are so different to previous reports.
Reply: Thank you for the professional suggestion. We think it is because of the major reason that we use the whole genome analysis rather than the BAC sequences. However, this comparison is a little unfairly as whole genome possess more information naturally. We are doing more analysis about ginseng repeats. We hope we can get more interesting information from our data.

Minor comments

1) Figure 1a should be a table, Figures 1b and 1c needs either more explanation over new findings or important confirmation or may just be moved to supplementary. I personally find it impossible to extract any information from figures like 1c.
Reply: Considering the limit in number of figures/tables and that Fig. 1a seems to be insufficiently vivid as an individual table, this figure was thus integrated into Fig. 1. We also believed that rebuilding the phylogentic tree can show the possible speciation history of different species and give feedback information for genome-assembly quality. Given that phylogenetic and Venn diagram analyses are conventional methods (Guan et al., 2016; Wei et al., 2016), we decided to retain keep these information in the original manuscript.

2) The abstract should be adjusted to get rid of unnecessary nitty-gritty details, and provide a more focused description of what the manuscript is aiming for (assembly+rnaseq or biosynthesis analysis, but tighter and more conclusive). Conclusions should not be along the lines of "this will help further work" but at the very least along the lines of "this supports this, this, and this types of analyses/developments".
Reply: Thank you for your suggestion. We have rewritten the Abstract and deleted some information.
Background: Ginseng, which contains ginsenosides characterized as bioactive compounds, has been regarded as an important traditional medicine for several millennia. However, the genetic background of ginseng remains poorly understood partly because of the plant's large and complex genome composition.
Results: We report the entire genome sequence of Panax ginseng using next-generation sequencing. The 3.5 Gb nucleotide sequence contained more than 60% repeats and encoded 42 006 genes. Twenty-two transcriptome datasets and mass spectrometry images of ginseng roots were adopted to precisely quantify the functional genes. Thirty-one genes were identified to be involved in the mevalonic acid pathway. Eight of these genes were annotated as 3-hydroxy-3-methylglutaryl-CoA reductases, which displayed diverse structures and expression characteristics. A total of 225 UDP-glycosyltransferase (UGTs) were identified, and these UGTs accounted for one of the largest gene families of ginseng. Tandem repeats contributed to the duplication and divergence of UGTs. Molecular modeling of UGTs in 71, 74, and 94 families revealed a regiospecific conserved motif located at the N-terminus. Molecular docking predicted that this motif captured ginsenoside precursors.
Conclusions: The panorama of ginseng genome is a valuable resource for understanding and improving the breeding, cultivation, and synthesis biology of this key herb.

3) The organisation of the text could be much clearer (i.e. have all the data about experimental design and sequencing for genome assembly in one place in the methods, the same for differential expression, etc).
Reply: Thank you for this suggestion, some adjustments have been made to increase text clarity in R1.

4) There is no justification on the plant organisms selected for comparison.
Reply: Thank you for pointing this out. Giseng is a traditional herb and has a long cultivation history in East Asia. We selected the plant organisms based on the physiological characteristics of P. ginseng during its cultivation. We also referred to some references regarding organism selection. We selected tissues based on the mass-spectrometry imaging results of ginseng root. Our future studies will include more detailed comparison works.

5) Figure 3b and 3c are plotted in such a way that some points are "hidden" behind other points.
Reply: Thank you for pointing this out. We have modified the Fig. 3b and 3c in R1 to show all sample points.

6) Figure 5 is the first point where "4-year-old" is mentioned. This belongs in methods and needs some explanation.
Reply: Thank you for pointing out this issue. We described the sample information in the method section in R1.

7) Figures 6b and 6c provide no information. Should be removed and/or a section (possibly in the supplementary) describing their results and importance should be explicitly written.
Reply: Thank you for the suggestion. We have deleted them in R1 considering that the microbial-resistance section has been removed.

8) The sections for phylogenetic analysis and gene family identification are vague and/or add little information for the rest of the manuscript's analyses. They can either be moved to supplementary or their links to the rest of the manuscript stressed and the analyses strengthened.
Reply: Thank you for the kind suggestion. We have highlighted the reason why we chose to keep this part in minor comment 1. Furthermore, we have strengthened our findings by highlighting the importance of phylogenetic analysis and introducing some necessary citations in R1.

9) In general the discussion has some claims that do not seem completely supported from the current analyses, but I feel a plant biologist will be better suited to judge that.
Reply: Thank you for your comment. These suggestions are indeed very valuable to our work.

Best regards,
Bernardo J. Clavijo
NOTE: review produced with extensive help from Luis Yanes from my research team.
