# Supplementary material for: Panax ginseng genome examination for ginsenoside biosynthesis
Source: Gigascience. 2017 Oct 5;6(11):1–15. doi: 10.1093/gigascience/gix093 (PMC5710592; doi:10.1093/gigascience/gix093)
Supplement: Response-to-Reviewer-Comments_Revision-1.pdf [file gix093_response-to-reviewer-comments_revision-1.pdf]

Author's Response To Reviewer Comments

Dear Dr. Hans Zauner,

We appreciate you and the reviewers for your precious comments. We have carefully considered all comments for our last version and accordingly revised our manuscript. Please find below our point-by-point replies to the comments and detailed explanations of all changes (“R1” refers to the submitted revised version and “R2” is the new revised version; all revisions in R2 were tracked). All the changes were highlighted in the revised manuscript. Thank you!

Reviewer #1: The manuscript entitled: "Ginseng genome examination for ginsenoside biosynthesis" by Xu Jiang et al. was previously revised and some major considerations were made. I would like to thank the authors for answering point-by-point all my inquiries. I'm satisfied with the answers and don't have any further questions. I'm okay with this version of the manuscript for its publication.

Thank you for reviewer #1’s positive comments and thank you for permitting our work. We acknowledge the useful suggestion of reviewer #1. Thank you very much.

Reviewer #2: The manuscript has substantially improved, there is still some points that I would like to see considered (minor) and fixed (major) before publication:

Many thanks for reviewer #2’s useful suggestion. Followings are our point-by-point replies, please check them.

Minor points partially addressed:
* The single-N issue with soap scaffolding still stands. It can be overlooked, although I would recommend it mentioned for transparency. In fairness I have failed to mention it on my own manuscripts on occasion out of a lack of knowledge about the issue, but it could help further understand assembly characteristics if needed. This can affect things like read mapping and gene annotation.
Thank you for pointing out this problem. We have added the information of single-Ns at the note of Table 1 Statistical analysis of the P. ginseng draft genome.
* The justification for the use of line IR826 needs to be written into the main manuscript, as soon as this line is mentioned.
Thank you for pointing out this problem. We have added the content in Page6, Line 16.
* The fact that some tissues come from another line needs to be written too and its possible implications for the analyses discussed (i.e. do the samples cluster by line?).
Thank you for pointing out this problem. We have added the cultivar name in Page 22 Line 1. We didn’t find obvious difference among samples.
* I am not sure if the UTG analysis never included the extra expression datasets (in which case it is ok) or if it did why it is not changing.
We are sorry we didn’t find any interesting information from the analysis of expression dataset in all UGTs, so we only put the expression analysis of a UGT73 gene cluster in this manuscript. We hope in future work we can get more useful information.

* The answer to my question about the ginsenosides' pathway should be included in the main text for clarity.
The pathway introduction was included in the introduction. We have highlighted in Page 4 Line 22.
* The copy number assessment justification could be included in the text for extra support (even if only in supplementary), for all relevant genes.
Thank you for this suggestion. We have added the justification at the supplementary(Supplementary Figure 10).
* Figure 1a should be a table. A table is a table, it sounds tautological but it is still true. Images do not allow automated data analysis by things like paper-crawlers and such.
Thank you for pointing out this problem. We agree with the reviewer's suggestion. We have split the table as Table 1.

Major points still standing:
* The library of nominal size 10Kbp is still referred as the "10Kbp" library in the manuscript without explanation of its actual 7.5Kbp fragment size mode. The effect of using this library as 10Kbp is noticeable both in the fragment size analysis the authors did and in the one I did. While this does not in my view invalidate the results of the assembly (most likely effect is to have some N runs of incorrect length here and there), the description of the library needs to be updated.
Thank you for pointing out this problem. We have added a column "Estimated insert size(bp)" at Supplementary Table S1. The estimated insert size was calculated using reads alignment.
* There is still no description of which lab protocol was used to produce LMP data. Mentions to transposase on the processing seem to indicate Nextera, we could not find content of nextera adaptors, so it would be good for reusability of this data to describe the protocol.
We are sorry for this negligence. Except the 2kb mate-paired library, all the libraries were constructed using the commercial library prep kits (Vazyme Biotech). The 2kb mate-paired library was constructed using 454 method(Cre/loxp recombination system), the linkage adapter was 5'-CGTAATAACTTCGTATAGCATACATTATACGAAGTTATACGA-3'. We have added the instruction in Page 20 Line 15. The check results of adapters were listed as following:

2kb

5kb

10kb

* The filtering of scaffolds <1000bp, which originates some sequence loss, is not mentioned in the manuscript. While this may be the right choice, it needs to be written and its effect of "possibly loosing some content" at the very least mentioned.
Thank you for mentioning this problem. We have noticed it in Page 21 Line 11, please check it.
* While the Figure in 5c was updated, the description was not. This could be expanded to show the main finding of this particular figure's analysis, which is unclear to me given that most of the patters are not clear now that the points are being represented.
We apologize for this negligence. The description in previous manuscript has been updated, but without labeling. Thereby, we just added the label of Fig 5c in the manuscript R2 at Page 14 Line

18. Thank you for your chariness suggestion.
* The estimation of LTR at 1.5x what was previously thought needs to be either put in context or presented with extreme care to highlight methodological differences.
Thank you for pointing out this problem. We have added the comments for the LTR change in Page 17 Line 5. We agreed with the reviewer that it should be highlighted here for the difference of BAC and WGS.
