# Supplementary material for: Panax ginseng genome examination for ginsenoside biosynthesis
Source: Gigascience. 2017 Oct 5;6(11):1–15. doi: 10.1093/gigascience/gix093 (PMC5710592; doi:10.1093/gigascience/gix093)
Supplement: Response-to-Reviewer-Comments_Revision-2.pdf [file gix093_response-to-reviewer-comments_revision-2.pdf]

Author's Response To Reviewer Comments

Dear Dr. Hans Zauner,

Thank you for your information, and thanks for your and the reviewers’ hard work. We have added a description of 10 kb library in the section Method (L18-21, P20: After assembly, the average estimated span distance of the 10 kb library was about 7.5 kb, we speculated that the shrinkage owing to the molecule disruption during library preparation (Supplementary Table S1)). We hope this description can help readers to use our data. We also have corrected some language problems. The new version of our manuscript is named R3, without any highlighting/tracking of changes, please check it. Thank you!

Best wishes,

Xu Jiang
