# Supplementary material for: Panax ginseng genome examination for ginsenoside biosynthesis
Source: Gigascience. 2017 Oct 5;6(11):1–15. doi: 10.1093/gigascience/gix093 (PMC5710592; doi:10.1093/gigascience/gix093)
Supplement: Reviewer-1-Report-(Original-Submission).pdf [file gix093_reviewer-1-report-(original-submission).pdf]

Reviewer Report

Title: Ginseng genome examination for ginsenoside biosynthesis

Version: Original Submission **Date:** 3/22/2017

Reviewer name: Elsa Gongora

Reviewer Comments to Author:

The manuscript entitled: "Ginseng genome examination for ginsenoside biosynthesis" by Xu Jiang et al. presents results of sequencing, characterization and annotation of the P. ginseng genome. The authors have further shown the distribution of the ginsenosides molecules in root tissues using mass spectrometry and liquid chromatography, and in order to identify candidate genes possible involved in the ginsenosides biosynthesis pathway an analysis of expression and co-expression networks using RNA-seq data was performed. A set of key genes involved in the ginsenosides biosynthesis were further characterized and interestingly some of them showed tissue specificity and activity during microbial infection. Overall, the experiments and analysis for this study were well conducted. And the results presented in this manuscript are important for the research of its field. However, the manuscript presents major faults that need to be addressed prior publication.

Major comments
1. How old were the P. ginseng plants used for genome sequencing?
2. The Microbial resistance section needs to be greatly improved since there is a lot of missing information:
a. The statement "Ginsenosides comprise a group of defense metabolites" needs to be supported by a reference.
b. What does the authors mean in the statement: "responses to microbial infection are important for ginsenoside biosynthesis"? Is the ginsenosides biosynthesis pathway lead by defense responses of the plant? Does this mean that if the plant is not under biotic stress the ginsenosides are not produced? What happen with the ginsenosides biosynthesis during C. destructans infection? Does the ginsenosides increase? Please clarify and support the statement above with a scientific reference.
c. The authors mentioned that 1652 resistance genes were annotated base on "plant resistance gene database". Please add the reference for this database.
d. The last paragraph on page 15 starting at "The expression analysis showed that 160 resistance genes …" till the end, it is not supported by any figure or table and it is not clear what expression analysis were performed. (FPKM?, WGCNA?, qPCR?)
e. The first paragraph on page 16 "The transcriptomes of ginseng induced by C. destructants … were recalculated". What does the authors mean by "transcriptomes were recalculated"? Did the authors sequence RNA of infected plants? All this information is missing in the methods section.
f. In the next statement, starting at "A total of 35008 genes were predicted, of these genes 28481 were expressed … the initial point" has to be supported by evidence. There is not figure or table that shows this analysis. How did the authors get the genes? Did they perform an RNA-seq assembly? If it so, where

are the methods and metrics of the RNA-seq assembly?
g. The statement "At 0.25 DPI, 104 resistance genes were highly expressed and were possibly involved in C. destructans recognition" needs to be supported by evidence and a reference. The level of gene expression might not be related with the pathogen recognition at all.
h. The next paragraph starting at "At 0.25 DPI" till "Figure S10" describes a GO enrichment test described in figure S10. However figure S10 does not show what described in the text.
i. The entire section is not well described or described at all in methods.

1. The methods and tools in methods sections are not properly cited. For example, BLAST, WGCNA, R, etc., lack of reference. Databases such as GO, KEGG, KOG, etc., are not properly cited. The authors should cite the scientific manuscript of the tools and databases and not only the website.
2. Repeat prediction, gene prediction and annotation section in Methods. Page 26. The authors mention that transcripts assembled from RNA-seq data were use for MAKER-P annotation. However there is none information regarding to RNA-seq assembly. What assembler was used? What parameters were used? How many transcripts were obtained? Etc.,The metrics of the transcriptome assembly are not described at all.

Minor comments
Page 7. Missing space: "RNA-seqlibraries"
Page 7. Please add the reference for "13 published ginseng RNA-seq data".
Page 13. Figure 5. Please rename figures in order of citation. (5a, 5d, 5c, 5b -> 5a, 5b, 5c, 5d)
Page 14. The sentence "The expression patterns of different HMGR types differed" does not make sense. Please re-phrase.
Page 20. Add the figure number that support this statement: "same PgHMGR subfamily, they exhibited different correlation patterns with other genes"
Page 22. Genome sequencing and assembly. How old were the plants used in this study?
Page 22. Please add the scientific reference for SOAPdenovo assembler.
Page 23. Last row of the first paragraph. There is missing information for the cutoff criteria. "BLAStN, a cutoff value of 90%". What is this 90%? Identity?
Page 28. Please add the reference for PyMOL.

Figures:
Figure 5b. What does it show? FPKM values? Is it an qPCR? Y axis needs a label. In the figure legend the figure was described as a qPCR analysis however nothing of this is described in methods and primers sequences are not provide.
Figure S10. The legend of this figure does not provide enough information of the figure.

Methods

Are the methods appropriate to the aims of the study, are they well described, and are necessary controls included? No

Conclusions

Are the conclusions adequately supported by the data shown? No

Reporting Standards

Does the manuscript adhere to the journal's guidelines on [minimum standards of reporting?]() YesChoose an item.

Statistics

Are you able to assess all statistics in the manuscript, including the appropriateness of statistical tests used? There are no statistics in the manuscript.

Quality of Written English

Please indicate the quality of language in the manuscript: Acceptable

Declaration of Competing Interests

Please complete a declaration of competing interests, considering the following questions:

- Have you in the past five years received reimbursements, fees, funding, or salary from an organisation that may in any way gain or lose financially from the publication of this manuscript, either now or in the future?
- Do you hold any stocks or shares in an organisation that may in any way gain or lose financially from the publication of this manuscript, either now or in the future?
- Do you hold or are you currently applying for any patents relating to the content of the manuscript?
- Have you received reimbursements, fees, funding, or salary from an organization that holds or has applied for patents relating to the content of the manuscript?
- Do you have any other financial competing interests?
- Do you have any non-financial competing interests in relation to this paper?

If you can answer no to all of the above, write 'I declare that I have no competing interests' below. If your reply is yes to any, please give details below.

I declare that I have no competing interests

I agree to the open peer review policy of the journal. I understand that my name will be included on my report to the authors and, if the manuscript is accepted for publication, my named report including any attachments I upload will be posted on the website along with the authors' responses. I agree for my report to be made available under an Open Access Creative Commons CC-BY license (http://creativecommons.org/licenses/by/4.0/). I understand that any comments which I do not wish to be included in my named report can be included as confidential comments to the editors, which will not be published.

I agree to the open peer review policy of the journal

To further support our reviewers, we have joined with Publons, where you can gain additional credit to further highlight your hard work (see: https://publons.com/journal/530/gigascience). On publication of this paper, your review will be automatically added to Publons, you can then choose whether or not to claim your Publons credit. I understand this statement.

Yes
