# Supplementary material for: Panax ginseng genome examination for ginsenoside biosynthesis
Source: Gigascience. 2017 Oct 5;6(11):1–15. doi: 10.1093/gigascience/gix093 (PMC5710592; doi:10.1093/gigascience/gix093)
Supplement: Reviewer-2-Report-(Revision-1).pdf [file gix093_reviewer-2-report-(revision-1).pdf]

Reviewer Report

Title: Ginseng genome examination for ginsenoside biosynthesis

Version: Revision 1 **Date:** 8/9/2017

Reviewer name: Bernardo Clavijo

Reviewer Comments to Author:

The manuscript has substantially improved, there is still some points that I would like to see considered (minor) and fixed (major) before publication:

Minor points partially addressed:
* The single-N issue with soap scaffolding still stands. It can be overlooked, although I would recommend it mentioned for transparency. In fairness I have failed to mention it on my own manuscripts on occasion out of a lack of knowledge about the issue, but it could help further understand assembly characteristics if needed. This can affect things like read mapping and gene annotation.
* The justification for the use of line IR826 needs to be written into the main manuscript, as soon as this line is mentioned.
* The fact that some tissues come from another line needs to be written too and its possible implications for the analyses discussed (i.e. do the samples cluster by line?).
* I am not sure if the UTG analysis never included the extra expression datasets (in which case it is ok) or if it did why it is not changing.
* The answer to my question about the ginsenosides' pathway should be included in the main text for clarity.
* The copy number assessment justification could be included in the text for extra support (even if only in supplementary), for all relevant genes.
* Figure 1a should be a table. A table is a table, it sounds tautological but it is still true. Images do not allow automated data analysis by things like paper-crawlers and such.

Major points still standing:
* The library of nominal size 10Kbp is still referred as the "10Kbp" library in the manuscript without explanation of its actual 7.5Kbp fragment size mode. The effect of using this library as 10Kbp is noticeable both in the fragment size analysis the authors did and in the one I did. While this does not in my view invalidate the results of the assembly (most likely effect is to have some N runs of incorrect length here and there), the description of the library needs to be updated.
* There is still no description of which lab protocol was used to produce LMP data. Mentions to transposase on the processing seem to indicate Nextera, we could not find content of nextera adaptors, so it would be good for reusability of this data to describe the protocol.
* The filtering of scaffolds <1000bp, which originates some sequence loss, is not mentioned in the manuscript. While this may be the right choice, it needs to be written and its effect of "possibly loosing some content" at the very least mentioned.

* While the Figure in 5c was updated, the description was not. This could be expanded to show the main finding of this particular figure's analysis, which is unclear to me given that most of the patters are not clear now that the points are being represented.
* The estimation of LTR at 1.5x what was previously thought needs to be either put in context or presented with extreme care to highlight methodological differences.

With my best wishes,

Bernardo J. Clavijo

Methods

Are the methods appropriate to the aims of the study, are they well described, and are necessary controls included? Yes

Conclusions

Are the conclusions adequately supported by the data shown? Yes

Reporting Standards

Does the manuscript adhere to the journal's guidelines on minimum standards of reporting? YesChoose an item.

Statistics

Are you able to assess all statistics in the manuscript, including the appropriateness of statistical tests used? Yes, and I have assessed the statistics in my report.

Quality of Written English

Please indicate the quality of language in the manuscript: Acceptable

Declaration of Competing Interests

Please complete a declaration of competing interests, considering the following questions:

- Have you in the past five years received reimbursements, fees, funding, or salary from an organisation that may in any way gain or lose financially from the publication of this manuscript, either now or in the future?
- Do you hold any stocks or shares in an organisation that may in any way gain or lose financially from the publication of this manuscript, either now or in the future?

- Do you hold or are you currently applying for any patents relating to the content of the manuscript?
- Have you received reimbursements, fees, funding, or salary from an organization that holds or has applied for patents relating to the content of the manuscript?
- Do you have any other financial competing interests?
- Do you have any non-financial competing interests in relation to this paper?

If you can answer no to all of the above, write 'I declare that I have no competing interests' below. If your reply is yes to any, please give details below.

I declare that I have no competing interests

I agree to the open peer review policy of the journal. I understand that my name will be included on my report to the authors and, if the manuscript is accepted for publication, my named report including any attachments I upload will be posted on the website along with the authors' responses. I agree for my report to be made available under an Open Access Creative Commons CC-BY license (http://creativecommons.org/licenses/by/4.0/). I understand that any comments which I do not wish to be included in my named report can be included as confidential comments to the editors, which will not be published.

I agree to the open peer review policy of the journal

To further support our reviewers, we have joined with Publons, where you can gain additional credit to further highlight your hard work (see: https://publons.com/journal/530/gigascience). On publication of this paper, your review will be automatically added to Publons, you can then choose whether or not to claim your Publons credit. I understand this statement.

Yes
